# Pyridine‐Fused Bis(azacorrole)s: Easily Accessible NIR III Absorbing Cation Radicals and Biradicaloids of Antiaromatic Ground State

**DOI:** 10.1002/advs.202416223

**Published:** 2025-03-28

**Authors:** Sha Li, Shaowei Zhang, Xiaofang Li, Oskar Smaga, Kinga Szydełko, Miłosz Pawlicki, Piotr J. Chmielewski

**Affiliations:** ^1^ Key Laboratory of Theoretical Organic Chemistry and Functional Molecules Ministry of Education School of Chemistry and Chemical Engineering, Hunan University of Science and Technology, Xiangtan, Hunan 411201, China; ^2^ Department of Chemistry University of Wrocław 14 F. Joliot‐Curie Wrocław 50383 Poland; ^3^ Faculty of Chemistry Jagiellonian University Gronostajowa Kraków 230387 Poland

**Keywords:** antiaromaticity, aromaticity, biradicaloids, porphyrinoids, radicals

## Abstract

A family of pyridine‐fused bis(porphyrinoids) is obtained, including constitutionally isomeric bis(azacorrole)s, azacorrole‐oxacorrole, as well as azacorrole‐norcorrole heterodimers by two distinct synthetic approaches. Spectroscopic characteristics, corroborated by Density Functional Theory (DFT) calculations, indicate aromaticity of the bis(azacorrole) as well as azacorrole‐oxacorrole products, while for the azacorrole‐norcorrole heterodimers, the presence of both dia‐ and paratropic currents is detected. Electrochemical analyses indicate facile chemical access to cation radicals and dicationic species that have been characterized by electronic and electron spin resonance spectroscopy as well as by DFT calculations. Monocations give rise to the relatively strong absorption bands in the near infra red (NIR) region between 2400 and 3200 nm, while dications are characterized by a series of absorptions between 1000 and 2200 nm. Electron spin resonance (ESR) experiments indicate the presence of singlet‐triplet spin equilibria for the dications. For the dication of bis(azacorrole) of the most planar structure, the singlet ground state is established, and low temperature nuclear magnetic resonance (NMR) as well as gauge‐independent atomic orbital NMR calculations indicate its antiaromatic character.

## Graphical abstract

A series of pyridine‐fused hetero‐ and homodimers containing azacorrole moiety is obtained. The spectral, redox, and magnetic properties of the bis(azacorrole)s reflect an electron‐donating character of these systems and interaction between the subunits which depends on the substitution pattern. An extremely narrow energy gap between highest‐occupied and lowest unoccupied molecular orbitals in the radicals and redox‐switching between aromatic and antiaromatic properties have been found.

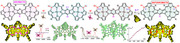

## 1 Introduction

**Figure 1:**
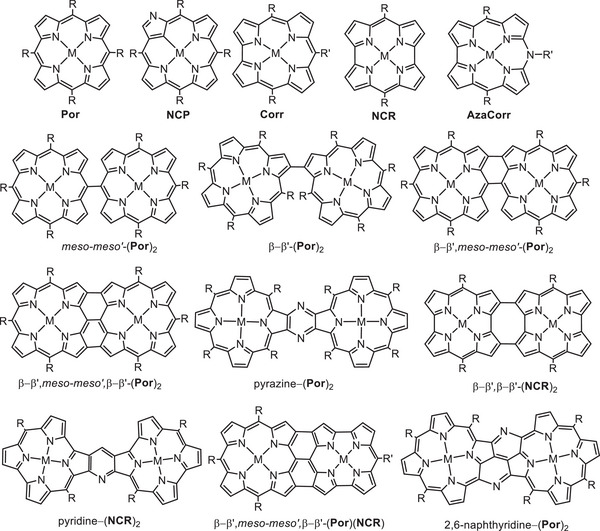
Schematic structures of metal complexes of tetrapyrrolic porphyrinoids and selected bis(porphyrinoids).

Porphyrins and porphyrinoids constitute a class of macrocycles comprising conjugated π‐electrons which can result in aromatic, non‐aromatic, or antiaromatic systems. Aromaticity and many other structural and electronic properties of porphyrinoids depend on the number of delocalized electron pairs, the size of the ring, atoms connectivity within the molecular skeleton, involvement of heteroatoms inside or on the ring periphery, the presence and type of metal ion within macrocycle, as well as overall 3D shape of the molecules.^[^ ^1^, ^2^, ^3^, ^4^, ^5^, ^6^, ^7^, ^8^, ^9^, ^10^, ^11^, ^12^, ^13^, ^14^, ^15^, ^16^ ^]^ Apart from the variety of tetrapyrrolic monomers, such as porphyrins (**Por**), N‐confused porphyrins (**NCP**), corroles (**Corr**), norcorroles (**NCR**), or azacorroles (**AzaCorr**) there are many structurally different oligomers with a direct bonding between the porphyrinoid subunits (**Figure** **1**).^[^ ^17^, ^18^, ^19^, ^20^, ^21^, ^22^, ^23^ ^]^ Among the plethora of porphyrinoid‐containing oligomeric systems, the strongest interaction between the subunits is observed or expected for the systems where macrocyclic rings are fused, either directly with β,β' and *meso*‐*meso'* bonds or sharing two or more atoms with the aromatic junctures.^[^ ^24^, ^25^, ^26^, ^27^, ^28^, ^29^ ^]^

**Scheme 1:**
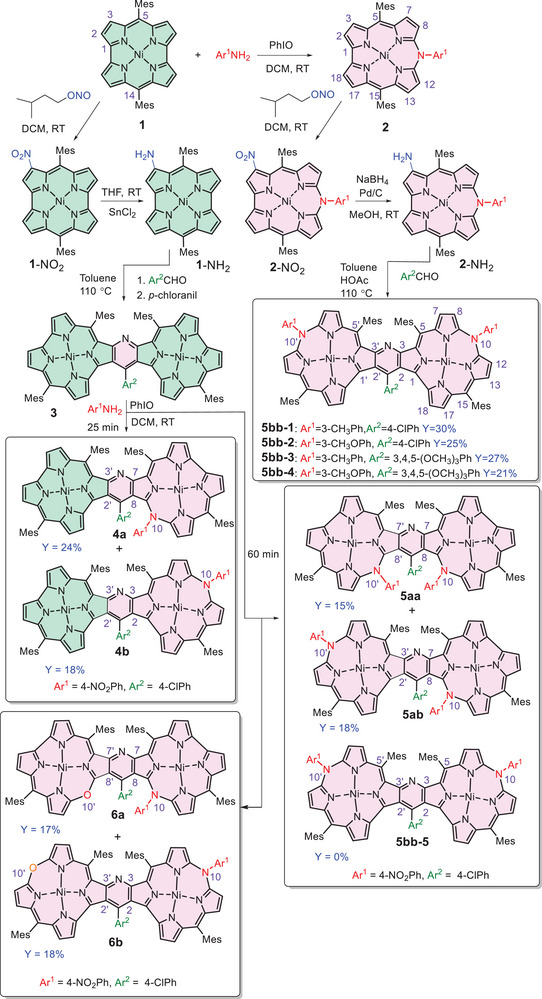
Synthetic paths toward pyridine‐fused bis(azacorrole)s and azacorrole‐containing heterodimers.

Fused porphyrin oligomers have been extensively developed due to intriguing optical and electronic properties that arise from their conjugated networks over the more or less coplanar molecular frameworks. The synthetic method leading to pyridine‐ or pyrazine‐fused oligoporphyrinoids involves the oxidative coupling of  NH_2_‐substituted monomeric or dimeric precursors.^[^ ^30^, ^31^, ^32^ ^]^ An analogous approach has been applied by Li and coworkers for a synthesis of pyridine‐fused norcorrolatonickel(II) dimer consisting of fused antiaromatic subunits.^[^ ^33^ ^]^ Recently, we have shown that the antiaromatic nickel(II) complex of norcorrole **1** can be effectively transformed into aromatic *N*‐substituted 10‐azacorrolatonickel(II) **2** by oxidative insertion of aromatic or aliphatic amine to the macrocyclic ring (**Scheme** **1**), and such a ring expansion can be performed for various norcorrole derivatives.^[^ ^34^, ^35^, ^36^ ^]^ We thus decided to employ such a reactivity to the pyridine‐fused bis(norcorrole) **3** in order to explore structural, optical, magnetic, and redox properties of the novel systems consisting of both antiaromatic and aromatic macrocyclic subunits. On the other hand, pyridine‐fused bis(azacorrole)s that can be obtained in this way offer a platform for the exploration of pyridine‐mediated interactions between the aromatic macrocyclic subunits in different oxidation states.

Herein, we reported the synthesis and characterization of novel pyridine‐fused systems comprising azacorroles. We focused on the aromaticity/antiaromaticity of the fused systems and the influence of the conjugation extension on spectroscopic and redox properties of the structurally diverse dimers with the same linking mode.

## 2 Results and Discussion

### 2.1 Synthesis and Structure

Two alternative synthetic paths toward bis(azacorroles) were applied. The first approach involved iodosobenzene‐supported oxidative insertion of aromatic amine^[^ ^34^ ^]^ into the predefined antiaromatic dimer **3**,^[^ ^33^ ^]^ whilst in the second method, a Hantzsch‐type pyridine cyclization was applied for the aromatic 3‐NH_2_‐substituted azacorroles (**2**‐NH_2_),^[^ ^35^ ^]^ mimicking that employed in the synthesis of **3** (Scheme 1).^[^ ^33^ ^]^ The first method resulted in the formation of several products that comprised either one or two azacorrole subunits. As expected, a shorter reaction period (25 min) gave rise to isomeric mono‐arylaza heterodimers **4a** and **4b** consisting of one antiaromatic norcorrole and one aromatic azacorrole ring fused with pyridine (yields were 24% and 18%, respectively, and 35% of **3** remained unreacted), while 60 min of reaction was sufficient for insertion of arylaza bridges into both subunits. Under such conditions, a certain amount (jointly, up to 35%) of **3** was converted into isomeric “oxa‐aza” heterodimers **6a** and **6b** comprising 10‐arylaza‐ and 10′‐oxa‐corrole subunits fused with pyridine (Scheme 1). Surprisingly, formation of only two isomeric pyridine‐fused bis(azacorroles) **5aa** and **5ab** was observed, and the third possible isomer, i.e., **5bb‐5** (Ar^1^ = 4‐NO_2_Ph, Ar^2^ = 4‐ClPh) with C2, C3, C2', C3' pyrrole carbons involved in the fusion, was absent in the reaction mixture. The absence of this isomer, for which density functional theory (DFT) calculations revealed the lowest energy among isomers of **5** (by 33.8 and 18.7 kcal mol^−1^, comparing with **5aa** and **5ab**, respectively; see Table S2 and Figure S121, Supporting Information) may be related to a mechanism of the arylamine insertion, involving a splitting of the α–α‘ bond of the bipyrrole moiety upon oxidation with iodosobenzene.^[^ ^34^ ^]^ Such a ring opening apparently facilitates OH^−^ or water molecule attack instead of the amine at the well‐exposed 9′,10′‐bipyrrole unit in **4b**, leading to the oxa‐aza product **6b** rather than to the symmetric homodimer **5bb‐5**. Thus, the first method gives rise to many products but is devoid of that which seemed to be the most likely to form.

Conversely, the second synthetic method was completely regioselective, solely giving rise to the formation of **5bb** with 21–30% yields. The selectivity in this case was likely due to the steric factors introduced by Ar^1^ and Ar^2^ as well as to the predefined positions of NH_2_ relative to that of the aza‐aryl substituent in the monomeric **2**‐NH_2_.

**Figure 2:**
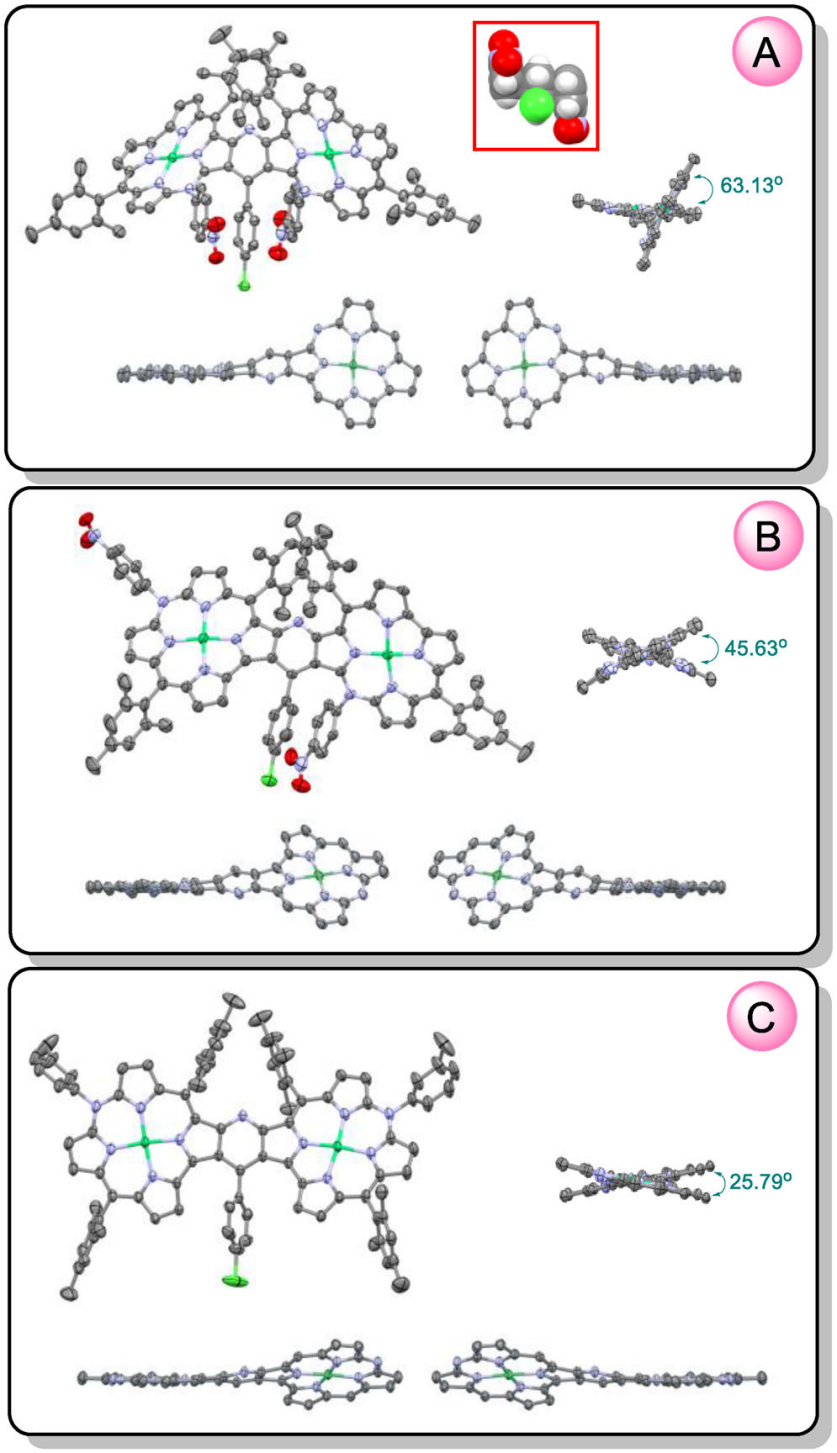
Molecular structures of **5aa** (A), **5ab** (B), and **5bb‐1** (C) based on single crystal XRD, drawn with 50% probability ellipsoids. The right‐hand side views, present dihedral angles between mean planes of the macrocyclic rings, and the bottom side views show enantiomers of each of the dimers. Hydrogen atoms, and in the side‐view plots, all aryl substituents are omitted.  The red box in panel A comprises a space‐filling representation of the crowded region of the aryl rings at the positions C10, C_γ_, and C10'.

**Figure 3:**
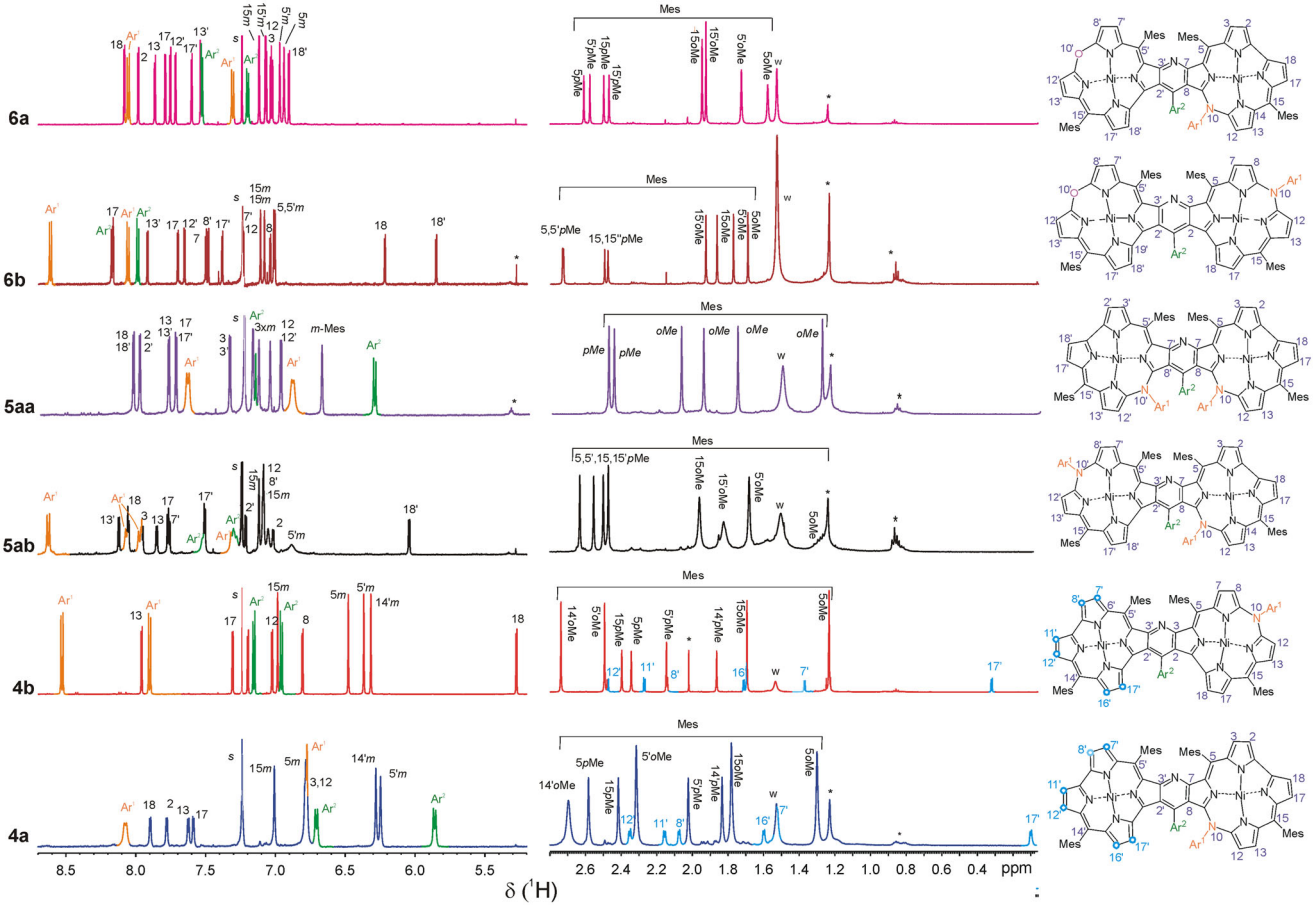
^1^H NMR spectra (600 MHz, 300 K, CDCl_3_) and signal assignments for the specified pyridine‐fused azacorrole systems. The residual CHCl_3_, impurities, and dissolved water signals are marked with s, *, and w, respectively. For all the compounds Ar^1^ = 4‐NO_2_Ph and Ar^2^ = 4‐ClPh.

All new compounds were characterized by high‐resolution mass spectrometry (HRMS), nuclear magnetic resonance (NMR), and ultra violet‐visible‐near infra red (UV–vis–NIR) spectroscopy (for complete synthetic and analytical details, see the Experimental Section in Supporting Information). For the representative products, i.e., **5aa**, **5ab**, and **5bb‐1**, single crystal X‐ray diffraction (XRD) analyses were performed (**Figure** **2**). For **4a**, due to a poor crystal quality only a preliminary solution was possible (Figure S182, Supporting Information). The HRMS confirmed the composition of the products, while ^1^H NMR indicated their symmetry, aromatic character of **5** and **6**, and mixed aromatic‐antiaromatic properties of **4** (**Figure** **3**). The full assignments of the signals by means of the 2D correlation experiments, allowed the identification of the isomers. Thus, the spectra of **5aa** and **5bb** each consisted of only one set of six signals of the pyrrole β‐protons resonating in the low‐field region, likewise for monomeric azacorroles (δ 6.2–8.3 ppm). The non‐symmetric substitution pattern in **5ab** resulted in two sets of the pyrrole proton signals in the same low‐field region with a unique proton 18‐H resonating at δ 6.0 ppm owing to a screening by aromatic ring current of Ar^2^. Similar dissymmetry was observed for heterodimers **6** represented by twelve β‐pyrrole proton doublets in the region δ 6.9–8.1 ppm for **6a** and δ 5.8–8.2 for **6b** indicating the presence of the aromatic ring current in both fused macrocycles similar to the monomeric aza‐ or oxacorroles.^[^ ^34^, ^35^, ^37^ ^]^ Conversely, the ^1^H NMR spectra of the heterodimers **4** consisted of six pyrrole doublets in the low‐field and six such signals in the high‐field regions (δ 6.8–7.9 ppm and 0.1–2.3 for **4a**; δ 5.3–8.0 ppm and 0.32–4.7 ppm for  **4b**), in line with the aromatic character of the azacorrole ring and antiaromatic properties of the fused azacorrole moiety. Unlike recently reported triply‐linked norcorrole‐porphyrin hybrid β,β'‐*meso*‐*meso*'‐β,β'‐(**Por**)(**NCR**) (Figure 1), for which a low‐lying triplet excited state contribution has been shown,^[^ ^38^ ^]^ the heterodimers **4** were in the closed‐shell singlet ground state and no temperature dependence of their ^1^H NMR was observed.

Single crystal X‐ray diffraction analyses revealed essentially planar structures of the azacorrole subunits, except those 7,8‐fused in **5aa** and **5ab** where ≈0.7 Å total out‐of‐plane deviation with predominantly ruffling distortion of the azacorrole rings were observed. The molecules were twisted, and the dihedral angles between mean planes defined by 24 heavy atoms of macrocyclic ring including central nickel, were 63.1°, 45.6°, and 25.8° for **5aa**, **5ab**, and **5bb‐1**, respectively. Also, the mean plane of the bridging pyridine ring was not coplanar with macrocyclic rings, and dihedral angles to the mean planes of the azacorrole subunits were 39.1° and 32.3° for **5aa**, 17.2° and 29.1° for **5ab**, and 13.7° for **5bb‐1**. These skews were likely related to the attenuating in this order steric repulsion of the aryl rings at N10 and that at the γ‐carbon of the pyridine bridge. The distances between centroids of the 4‐nitrophenyl substituents at N10 or N10' and 4‐chlorophenyl at C_γ_ of the pyridine bridge were 3.517 or 3.527 Å for **5aa** and 3.356 Å for **5ab**. The shortest interatomic distance 3.061(4) or 3.073(4) Å and 3.020(6) Å, respectively involved *ipso* carbons of these rings. XRD or density functional theory (DFT) models of norcorrole‐azacorrole heterodimers **4a** and **4b** also indicated lack of coplanarity of the macrocyclic rings with dihedral angle of 39.5° and 20.0°, respectively. No such twists have been observed for the pyridine‐fused bis(norcorrole) **3** for which a ruffled structure of the dimer arises from bowl‐like distortion of the subunits.^[^ ^33^ ^]^ On the other hand, in the highly twisted pyrazine‐fused bis(porphyrins), the subunits are roughly perpendicular to each other and strongly ruffled.^[^ ^30^ ^]^ Owing to their non‐planarity, the azacorrole‐containing systems were chiral, though they crystalized in centrosymmetric space groups as racemates.

### 2.2 Aromaticity and Antiaromaticity

**Figure 4:**
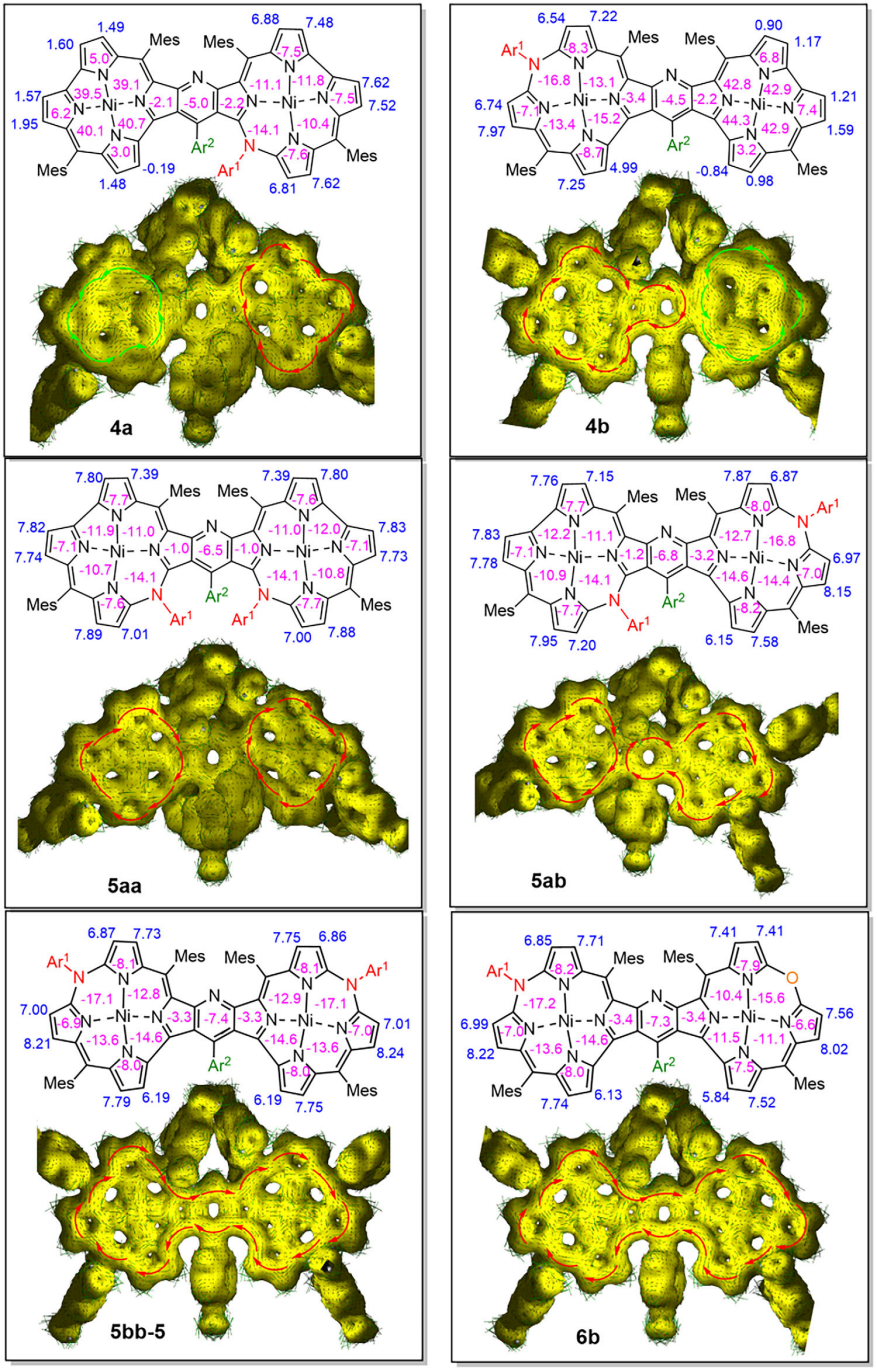
The GIAO‐calculated NICS(0) indices (purple numbers), ^1^H NMR chemical shifts of pyrrole protons (blue numbers), and AICD plots of the specified pyridine‐fused dimers containing azacorrole rings. Each of the NICS was calculated in the geometric midpoint of the five‐ or six‐membered ring. In AICD plots (0.025 isosurface), the diatropic (clockwise) induced current was depicted with red arrows, and the paratropic (counterclockwise) current with cyan arrows. In all systems Ar^1^ = 4‐nitrophenyl and Ar^2^ = 4‐chlorophenyl.

Theoretical analysis by the gauge‐independent atomic orbitals density functional theory (GIAO DFT) approach corroborated experimental assignments of the proton signals and conclusions regarding the presence of the diatropic and paratropic currents in the fused systems. The nucleus‐independent chemical shifts, NICS(*n*) indices, where *n* = –1, 0, or 1, calculated for all isomers of **5** were negative for both macrocyclic units in line with the aromatic character of the compounds (**Figure** **4**; Tables S5–S8, Supporting Information). The indices were somewhat less negative for the 7,8‐fused units in **5aa** and **5ab** than those for monomeric azacorrole **2** (Table S9, Supporting Information). In heterodimer **6b**, the NICS indices were significantly less negative for the oxacorrole subunit than those calculated for the azacorrole moiety of this compound, suggesting a stronger diatropic current in the latter (Table S8, Supporting Information). For the heterodimers **4**, the indices calculated for azacorrole moiety were negative but for the norcorrole part, they were positive reaching higher values of NICS(0) in **4b** (Figure 4, Tables S3 and S4, Supporting Information) than those of the monomeric norcorrole **1** ^[^ ^37^ ^]^ but close to the values calculated for the pyridine‐fused bis(norcorrole) **3**.^[^ ^33^ ^]^ The anisotropy of the induced current density (AICD) plots indicated exclusively an induced current of clockwise direction in **5** and **6**, and both dia‐ and paratropic currents of opposite directions in **4** (Figure 4). In **5ab**, **5bb**, and **6b**, the induced current was displaced onto the whole perimeter of the dimer skeleton, including bridging pyridine. No such a current appeared for azacorrole‐norcorrole hybrids **4a** and **4b**, in line with the observed and calculated shieldings for these systems strongly distinguishing chemical shifts of the protons belonging to the different subunits. Also in **5aa**, the diatropic ring current was confined to each of the macrocyclic subunits reflecting the severly twisted structure of the dimer.

### 2.3 Chirality and Dynamics of the Molecular Skeleton

Although **5bb‐1** and **5ab**, as well as **4a** were chiral in the solid state owing to a non‐planarity of these dimers, the ^1^H NMR indicated their configurational instability in solution reflected by the absence of diastereotopic differentiation of the dimer faces.  Conversely, despite a two‐fold symmetry of **5aa**, its spectrum comprised four distinct signals of *ortho*‐methyls as well as four signals of the mesityl *meta*‐protons, indicating diastereotopicity of the molecule faces and a slow chemical exchange between the stereoisomers. Also, time dependent density functional theory (TD‐DFT) calculation predicted relatively strong Cotton effects in the circular dichroic spectra of isomeric species **5** (Figure S139, Supporting Information). An attempt to separate the enantiomers using chiral stationary phase high‐performance liquid chromatography (HPLC) allowed an in situ observation of a weak optical activity of the fractions of **5aa** at 420 nm. However, the Cotton effect intensity diminished to zero within 1 min with *t*_1/2_ ∼ 13 s which prevented the recording of the full circular dichroic (CD) spectra of the enantiomers (Figures S140 and S141, Supporting Information). Thus, despite the spatial overcrowding present in **5aa**, the energy barrier for the configuration change appeared to be low, indicating the flexibility of the fused bis(azacorrole) systems and its transient planarity in solution. Consequently, in solution the effects of cooperation of the azacorrole rings related to delocalization of the π‐electron density may be stronger than those predicted on the basis of the single‐conformation calculations or the solid‐state structures.

### 2.4 Electronic Spectroscopy

**Figure 5:**
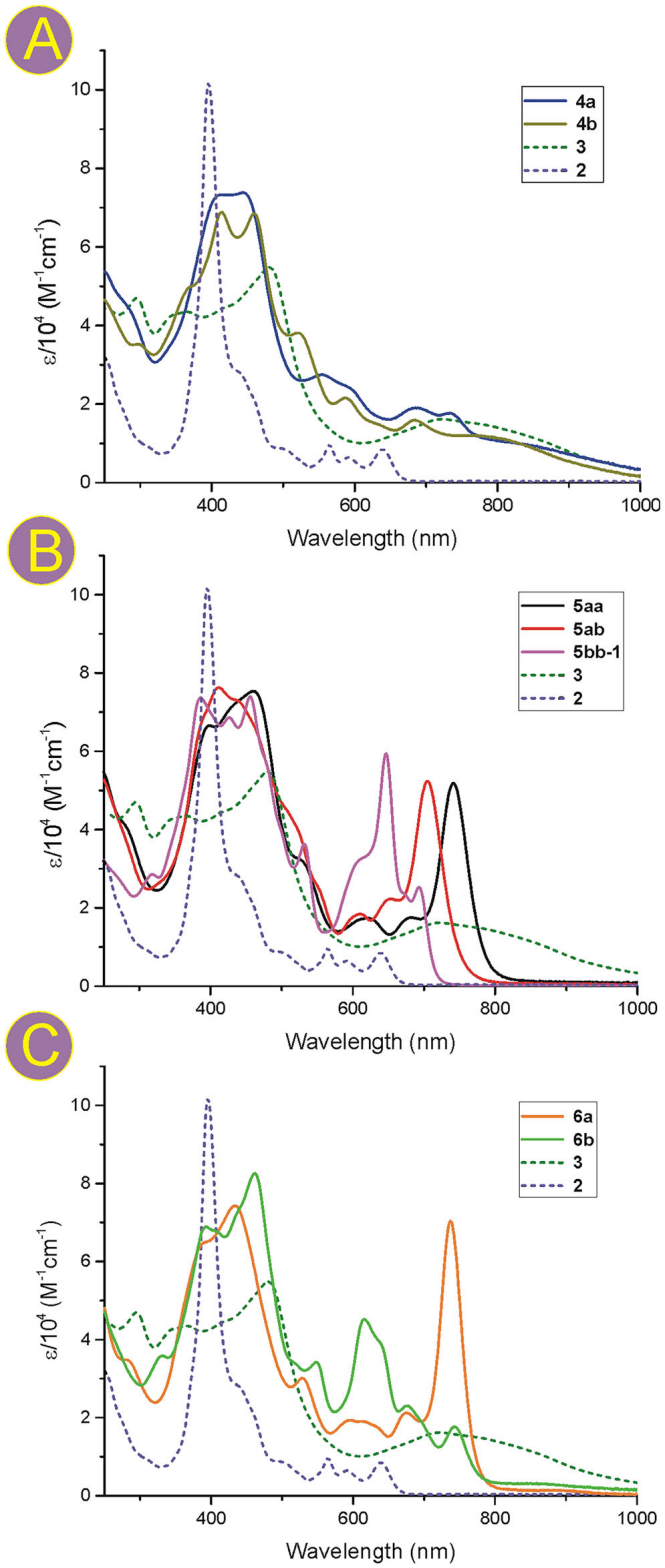
Electronic spectra (DCM, 298 K) of the specified pyridine‐fused azacorrole‐containing homo‐ and heterodimers: (A) azacorrole‐norcorroles; (B) bis(azacorrole)s; (C) azacorrole‐oxacorrole. For comparison, the spectra of monomeric 10‐(*p*‐nitrophenyl)‐azacorrole **2** and pyridine‐fused bis(norcorrole) **3** are also presented in each of the panels (dashed lines).

The electronic spectra of all pyridine‐fused bis(azacorroles) **5** differed considerably from those of the monomeric predecessors **2** and were characterized by multiply‐split Soret‐type bands in a region of 380–470 nm and strong absorptions in the Q‐region with most pronounced bands at 741, 705, and 647 nm for **5aa**, **5ab**, and **5bb‐1**, respectively (**Figure** **5**B). Similar spectroscopic characteristics were observed for oxa‐aza heterodimers **6a** and **6b** with split Soret bands and strong Q‐bands in the 600–800 nm region (Figure 5C). Also, these spectra differed considerably from those reported for the monomeric 10‐oxacorrole^[^ ^37^ ^]^ whose UV–vis characteristics are similar to that of azacorroles. Conversely, the spectra of the heterodimers **4a** and **4b**, apart from the Soret‐type bands typical of the “all aromatic” fused dimers **5**, and **6**, comprised broad and weak low‐energy absorptions, tailing up to ≈2000 nm similar to that observed for the pyridine‐fused bis(norcorrole) **3** (Figure 4A; Figure S53, Supporting Information). Evidently, the effect of the fusion on the optical properties of azacorroles appeared to be much stronger than that of multiple substitutions such as those reported previously.^[^ ^35^, ^36^ ^]^

### 2.5 Electrochemistry

**Figure 6:**
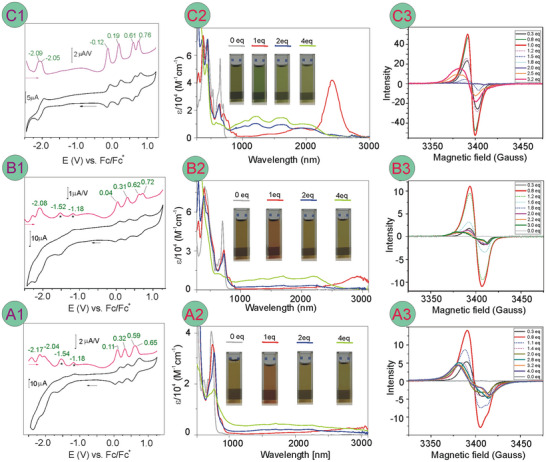
Left column: Cyclic (CV, black traces) and differential pulse (DPV, purple traces) voltammograms (DCM, glassy carbon electrode, [Bu_4_N]PF_6_ supporting electrolyte) of **5aa** (A1), **5ab** (B1), and **5bb‐2** (C1); green numbers at DPV are electrode potentials in volts. In DPV of **5aa** and **5ab**, the irreversible reduction peaks of NO_2_ groups were marked with asterisks. Mid column: Selected electronic spectra recorded upon titration of **5aa** (A2), **5ab** (B2), and **5bb‐2** (C2) with BAHA: 0 equiv. (gray traces), 1 equiv. (red traces), 2 equiv. (blue traces), and 4 equiv. (green traces). The spectra were modified by cutting off the uncompensated solvent IR overtone bands between 2695 and 2805 nm and replacing them with extrapolated straight lines. Right column: Selected frozen solution ESR spectra (DCM, 140 K) taken upon titration of **5aa** (A3), **5ab** (B3), and **5bb‐2** (C3) with BAHA.

The redox properties of the fused systems were studied by cyclic (CV) and differential pulse (DPV) voltammetry in dichloromethane (DCM) solutions with tetrabutylammonium hexafluorophosphate ([Bu_4_N]PF_6_) as a supporting electrolyte (**Figure** **6** A1‐C1; Figures S108–S120, Supporting Information, and **Table** **1**). For almost all systems, four reversible oxidation events were observed below 1 V with the first two processes occurring in a region from −0.12 to 0.34 V (all potentials were referenced with Fc^0/+^ couple as internal standard), indicating relative stability of mono‐ and dicationic forms of the fused systems. On the other hand, the reductions of the pyridine‐fused aromatic rings occurring near −2 V for **5bb**’s were strongly cathodic shifted compared to those of the antiaromatic bis(norcorroles) **3** observed at ≈−0.9 V.^[^ ^33^ ^]^ Significantly, for the heterodimers **4a** and **4b**, the first reduction appeared at ≈−1 V in line with the presence of electron‐accepting antiaromatic subunit. Unlike for the β‐nitrated derivatives of antiaromatic norcorrole or aromatic azacorrole, for which the first reduction takes place at the macrocyclic ring,^[^ ^34^, ^39^ ^]^ for **5aa**, **5ab**, **6a**, and **6b** irreversible reductions of *p*‐NO_2_ group at 10‐aryl substituents were observed at the potentials higher than *E*_red_ of azacorrole or oxacorrole ring.^[^ ^34^ ^]^ From the analysis of the redox potentials, it can be inferred that the pyridine‐fused bis(azacorroles) are stronger electron donors but much poorer electron acceptors, comparing to the analogues comprising one or two norcorrole subunits. The splitting of the oxidations and reductions waves of symmetric systems **5aa** and **5bb** indicated interactions between the subunits, as for the monomeric azacorroles single oxidation and reduction events have been observed over the comparable potential ranges.^[^ ^34^, ^35^, ^36^ ^]^

**Table 1:** Selected electrode potentials from electrochemical measurements for azacorrole‐containing systems in DCM solutions.

System	*E_red_*_1_ [V]	*E_red_*_2_ [V]	*E_red_*_3_ [V]	*E_ox_*_1_ [V]	*E_ox_*_2_ [V]	*E_ox_*_3_ [V]	*E_ox_*_4_ [V]	Δ*E*_HL_ [eV]
**5aa**	(−1.18, −1.54) ^a)^−2.04^b)^	−2.17	−2.41	0.11	0.32	0.58	0.64	
**5ab**	(−1.18, −1.52) ^a)^ −2.08 ^b)^	−2.27^b)^		0.04	0.31	0.62	0.72	
**5bb‐1**	−2.02	−2.07		−0.10	0.22	0.68	0.83	1.92
**5bb‐2**	−2.00	−2.06		−0.09	0.22	0.64	0.80	1.91
**5bb‐3**	−2.08	−2.02		−0.11	0.21	0.63	0.78	1.97
**5bb‐4**	−2.09	−2.05		−0.12	0.19	0.61	0.76	1.97
**4a**	−0.99	−1.63 (−1.54) ^a)^	−2.18	0.04	0.33	0.67	0.89^b)^	1.03
**4b**	−1.00	−1.63 (−1.47) ^a)^	−2.19	0.02	0.32	0.71	0.87^b^ ^)^	1.02
**3** ^[^ ^c^ ^]^	−0.87	−1.12	−1.66	0.03	0.28	0.74	0.88	0.90
**6a**	(−1.48) ^a)^ −1.81	−2.08	−2.19 ^b)^	0.13	0.34	0.63	0.75	
**6b**	(−1.49) ^a)^ −1.73	−2.09	−2.30 ^b)^	0.03	0.32	0.72	0.88	
**2**‐NO_2_ (*m*‐Tol)^d)^	−1.57	−1.99	−2.15	0.41	0.90	1.06		
**2**‐NO_2_ (*m*‐Anis) ^d)^	−1.59	−2.00		0.38	0.86	1.04		
**2**‐NH_2_ (*m*‐Tol) ^d)^	−2.15			−0.13	0.46			2.02
**2** (*p*‐NO_2_Ph)^d)^ ^e)^	(−1.11, −1.47) ^a)^ −2.15			0.21	0.68			
**2** (*m*‐Tol) ^d)^ ^e)^	−2.09			0.16	0.68			2.25
**2** (*m*‐Anis)^d)^ ^e)^	−2.10			0.15	0.66			2.25

^a)^ Irreversible waves of the NO_2_ group reductions;

^b)^ Irreversible wave;

^c)^ Data from ref. [33];

^d)^ Substituent Ar^1^ at N10.;

^e)^ Data from ref. [34].

### 2.6 Characterization of the Oxidation Products

The low oxidation potentials suggested facile multistep chemical oxidation of the fused bis(azacorrole) systems **5** which we studied by UV‐vis‐NIR and ESR spectroscopies.

#### 2.6.1 Radicals

Spectrophotometric titration with tris(*p‐*bromophenyl)ammoniumyl hexachloroantimonate (BAHA, Magic Blue) allowed, apart from the changes in the UV–vis region, observation of extremely low‐energy transitions for the monoxidized species (Figure 6 A2‐C2) with bands maxima reaching 3100 and 2930 nm for [**5aa**]^+•^ and  [**5ab**]^+•^, with low and moderate molar extinction coefficients *ε* = 3.0^.^10^3^ and 1.2^.^10^4^ m ^−1^cm^−1^, respectively, while a strong band was observed at ≈2420 nm (*ε* = 4.2^.^10^4^ m ^−1^cm^−1^) for [**5bb‐2**]^+•^ (Figure 6A2‐C2) and all other **5bb** cation radicals. A similar spectral pattern was observed for heterodimeric radical cation [**6a**]^+•^ with a relatively weak and broad absorbance in the region between 2200 and 3200 nm (Figure S154, Supporting Information). Significantly, no such strong bathochromic shift has been observed for monomeric 10‐azacorroles upon either electrochemical or chemical one‐electron oxidation with the longest wavelength band appearing below 800 nm.^[^ ^34^ ^]^ The one‐electron oxidation of heterodimer **4b** resulted in an ill‐defined broad band at ≈1150 nm tailing up to 2000 nm (Figures S148 and S149, Supporting Information). Apparently, the cation radical formed upon the addition of one equivalent of BAHA, required an extended π‐electron system of fused aromatic rings to give rise to the transitions of the energies as low as 0.4‐0.5 eV. Intensities of the lowest‐energy bands clearly increased in the order [**5aa**]^+•^ < [**5ab**]^+•^ < [**5bb**]^+•^ that may reflect the increasing planarity of the systems. The one‐electron oxidized species were also obtained using iodine solution (Figures S145–S147, Supporting Information) allowing exclusive observation of the monocations due to relatively low potential of the oxidant (≈0.2 V in DCM) with respect to the second oxidation potentials *E_ox_*_2_.

Liquid solution electron spin resonance (ESR) spectra of monocations consisted of unresolved signals at *g*_0_ = 2.0109, 2.0112, and 2.0122 for [**5aa**]^+•^, [**5ab**]^+•^, and [**5bb‐2**]^+•^, respectively, and such low values of the isotropic *g*_0_ are typical for nickel(II) radicals (Figures S125 and S131, Supporting Information)^[^ ^34^, ^40^ ^]^ rather than nickel(III) with innocent porphyrinoid ligands.^[^ ^41^, ^42^, ^43^, ^44^ ^]^ The frozen DCM solution ESR spectra recorded at 140 K consisted of an orthorhombic signal of *g_x_* = 2.0186, *g_y_* = 2.0123, *g_z_* = 2.0039 for [**5aa**]^+•^ and unresolved signals centered at *g* = 2.0113 for [**5ab**]^+•^ and at *g* = 2.0120 for [**5bb‐2**]^+•^ (Figure 6A3‐C3, red traces and Figures S124, S127, S131, and S134, Supporting Information). These spectra are similar to those observed previously for the one‐electron oxidized monomeric azacorrolatonickel(II) systems.^[^ ^34^ ^]^

**Figure 7:**
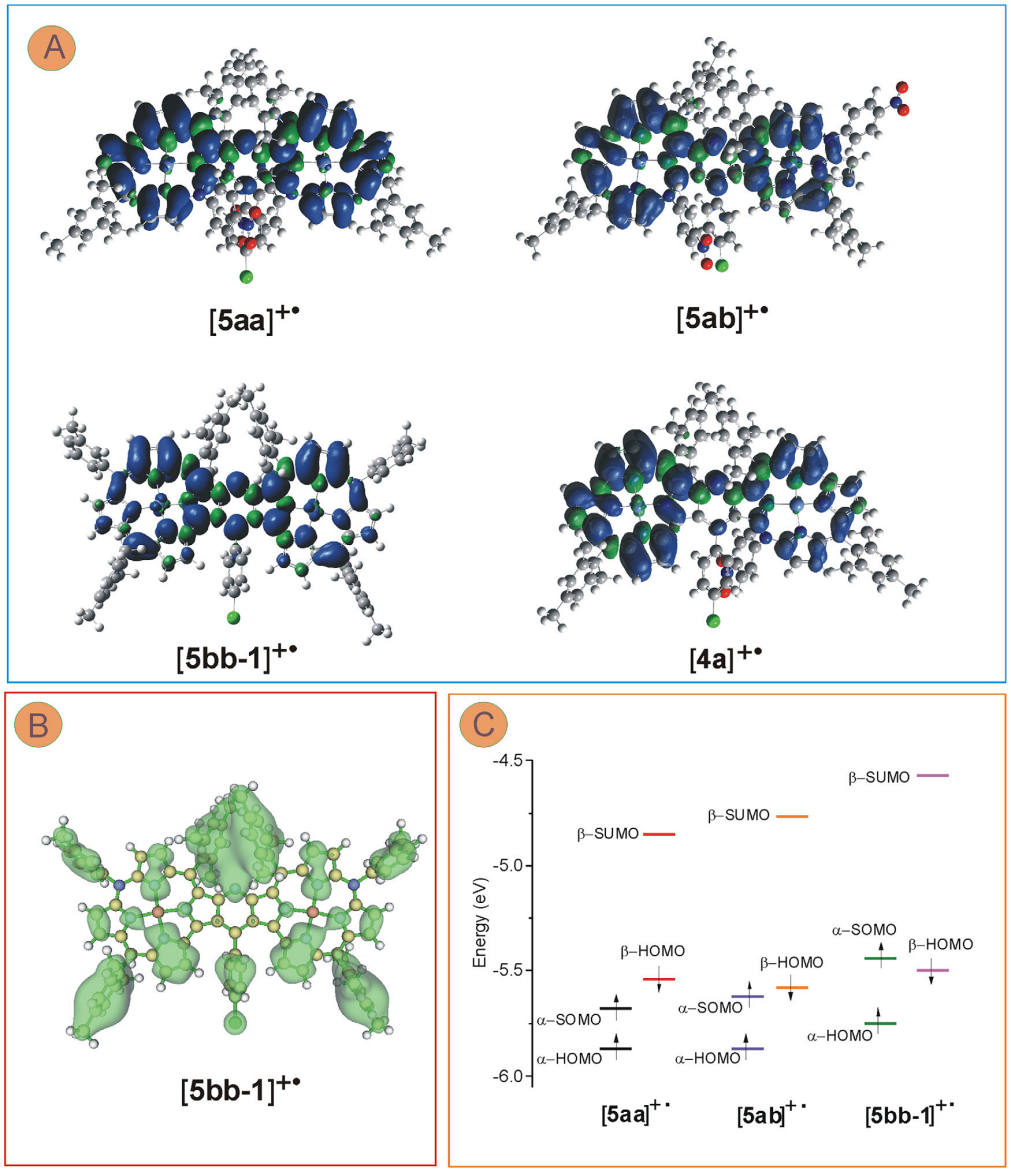
A) Calculated spin density distribution (isovalue 0.0004) in the one‐electron oxidation products of **5aa**, **5ab**, **5bb**, and **4a**. B) The positive charge distribution (green surface, isovalue 0.15) over the [**5bb‐1**]^+•^ skeleton derived from the NBO approach applied to DFT‐optimized structure. C) Calculated energies of the frontier spin orbitals of the cation radicals.

Time‐dependent DFT calculations of the electronic transitions corroborated the observations of the low‐energy absorption bands and assigned them to the β‐highest occupied molecular orbital → β‐lowest unoccupied molecular orbital (β‐HOMO → β‐LUMO) transitions at 2904, 2435, and 2038 nm for [**5aa**]^+•^, [**5ab**]^+•^, and [**5bb‐1**]^+•^, respectively (see Tables S18–S20 and Figures S168–S171, Supporting Information) with oscillator strengths values *f* = 0.299, 0.336, and 0.373, respectively, clearly indicating their highly allowed character. The calculations indicated ligand‐centered first oxidation with the spin density relatively evenly distributed among macrocyclic rings and bridging pyridine with a negligible contribution of nickel orbitals to the singly‐occupied molecular orbital (SOMO) (**Figure** **7**A; Figures S183–S185, Supporting Information). Thus, the mono‐oxidized forms were cation π‐radicals, and despite the considerable deviation from planarity in [**5aa**]^+•^ or [**5ab**]^+•^, communication between the subunits appeared to be feasible owing to the delocalized π‐electrons as well as the flexibility of the fused systems. The calculated orbital energies allowed interpretation of the observed and calculated tendency for the lowest‐energy, i.e., β‐HOMO → β‐LUMO electronic transitions for the radicals, that increased in the order [**5aa**]^+•^ < [**5ab**]^+•^ < [**5bb‐1**]^+•^ (observed: 0.39, 0.42, 0.51 eV; calculated: 0.43, 0.51, 0.61 eV, respectively; see also Figure S171 and Tables S18–S20, Supporting Information). Apparently, the transition energy was affected mostly by the increasing energy of the lowest empty orbital (β‐SUMO in Figure 7C), and that in turn may be due to relatively stronger destabilization of the LUMO orbitals in the more planar systems. Unlike the spin density in [**5bb‐1**]^+•^ that was delocalized virtually on all the atoms involved in the π‐electron framework of the dimer (Figure 7A), distribution of the positive charge in the monocation was calculated to be quite different with most of the positive charge resided at the peripheral aryl substituents and some pyrrole moieties, but not at the 10‐ or 10′‐N (Figure 7B). Since the first electron abstraction from the neutral azacorrole has been anticipated to take place from a lone pair of one the *meso*‐N,^[^ ^34^ ^]^ such a distribution appeared to be somehow unexpected. On the other hand, the subunits conjugation in the dimer may support electronegativity‐controlled distribution of the electron density, allowing its shift towards nitrogens.

The DFT‐optimized models of the radicals indicated virtually no structural changes upon oxidation. The oxidation, however, seemed to reduce the flexibility of the systems. Significantly, for [**5aa**]^+•^ comprising pyridine‐fused bis(azacorrole) of strongest deviation from the planarity, separations of enantiomers and stopped‐flow kinetics indicated considerably slower racemization compared with starting **5aa** with an increase of *t*_1/2_ from ≈13 s to more than 240 s. Such an increase in the system rigidity allowed an in situ recording of the circular dichroic spectra for the separated enantiomers of [**5aa**]^+•^ (Figures S142–S144, Supporting Information).

#### 2.6.2 Dications

Further addition of BAHA up to 2 equiv. resulted in a gradual decrease of the low‐energy near infra red (NIR) bands diagnostic for radicals, accompanied by less pronounced changes in the visible region (Figure 6A2–C2; Figures S151–S153 and S156–S158, Supporting Information). Concurrently, four bands with the maxima appeared between 1000 and 2200 nm and gained their intensities with saturation upon the addition of four equivalents of BAHA. Apparently, the spectra of di‐ and tricationic species strongly overlapped in this region and were hardly distinguishable. Tetracations were not expected to be accessible with the applied oxidant as the reduction potential of BAHA was too low (0.7 V).^[^ ^45^ ^]^ Similar results were obtained using solution of AgBF_4_ in DCM as an oxidant (Figures S149,S150,S155, Supporting Information).

The TD DFT calculations for t‐[**5aa**]^2+^, i.e., dicationic species in the triplet state, revealed transitions at 1688 and 1549 nm with oscillator strengths *f* = 0.0621 and 0.0085, respectively, while for the dication of the singlet ground state, s‐[**5aa**]^2+^, about an order of magnitude stronger transitions at 1945 and 1206 nm with *f* = 0.5418 and 0.1006, respectively were predicted by the calculations. The same tendency for weak absorption of triplets and strong absorption of singlets were occurred for the doubly oxidized **5ab** and **5bb‐1** with the transitions at 2046 nm (*f* = 0.0417) and 1608 nm (*f* = 0.0292) for t‐[**5ab**]^2+^, 1790 nm (*f* = 0.4845) and 1500 nm (*f* = 0.0146) for s‐[**5ab**]^2+^, 2230 nm (*f* = 0.0503) and 1940 (*f* = 0.0075) for t‐[**5bb**]^2+^, and 1440 nm (*f* = 1.1763) for s‐[**5bb**]^2+^ (see Figures S172–S180 and Tables S21–S26, Supporting Information). The relatively weak absorbance observed in this region for **5aa** and **5ab** upon the addition of two equivalents of BAHA, suggested domination of triplet states in solution at room temperature, in line with the prediction of low molar extinction coefficient for t‐[**5aa**]^2+^ or t‐[**5ab**]^2+^. On the other hand, a relatively stronger absorption observed in the NIR region of [**5bb**]^2+^ may reflect a higher contribution of the singlet for this species of more planar structure. However, a detailed analysis of the ground state of the dication based on the NIR absorption would be unjustified due to possible equilibria with mono‐ and trication, all absorbing in this region. Nevertheless, it can be concluded that the experimental spectra of the dications consisted of the bands derived from the species in both singlet and triplet spin states giving rise to a non‐zero absorption reaching beyond the NIR III window.

The formation of higher oxidized species derived from bis(azacorroles) was studied also by the ESR‐monitored titrations of **5** with the oxidant in both liquid and frozen solutions. Increase of the BAHA concentration in solution over 1 equiv., led to a decrease in the amplitude of the radical isotropic spectrum and a gradual signal formation at a higher value of *g*_0_ (Figures S126 and S130, Supporting Information). Similar conclusions could be inferred from the ESR of the oxidation products in frozen solution (DCM, 140 K, Figure 6A3‐C3; Figures S124–S135, Supporting Information). The initial increase in the radical signal evolved to its almost complete disappearance for **5bb‐2** and decrease of the intensity for **5aa** and **5ab** after adding two BAHA equivalents. These results may suggest the singlet state contribution for all the dicationic species. However, only for **5bb** almost fully diamagnetic solution can be obtained at 140 or 77 K in line with the “most planar” structure of the dimer allowing effective interaction between the oxidized subunits.  However, for neither of the dicationic systems the frozen solution ESR spectra displayed spectral features typical of *S* = 1 systems.^[^ ^46^ ^]^ Instead, the anisotropic ESR spectrum of the [**5aa**]^2+^ can be reproduced by simulation as a doublet spectrum of the orthorhombic Zeeman tensor with principal components *g*_*x*_ = 2.0228, *g*_*y*_ = 2.0153, and *g*_*z*_ = 2.0044. The addition of more than 2 equivalents of the oxidant resulted in the reappearance of an ESR signal for **5bb** and changes in the spectral parameters for the other systems which could be attributed to the formation of the tricationic species. The shape of the frozen‐solution spectra indicated a doublet rather than quartet, i.e., radical and not a tris(radical)^[^ ^47^ ^]^ nature of the trications at 140 K with the spin Hamiltonian parameters *g*_*x*_ = 2.0231, *g*_*y*_ = 2.0138, *g*_*z*_ = 2.0029 for [**5aa**]^3+^,  * g*_*x*_ = 2.0240, *g*_*y*_ = 2.0130, *g*_*z*_ = 2.0039 for [**5ab**]^3+^, and *g*_*x*_ = 2.0216, *g*_*y*_ = 2.0118, *g*_*z*_ = 2.0080 for [**5bb‐2**]^3+^.

#### 2.6.3 Stability

Although the oxidized species were reasonably stable at room temperature, lifespans of cation radicals and dications were limited in the presence of air in the DCM solution. For instance, in the case of [**5aa**]^+•^, the absorbance of the most characteristic band at ≈3000 nm diminished to the half of its intensity after 8 h when obtained by addition of 2 equiv. of iodine. The same effect was observed for [**5ab**]^+•^ after 16 h. For both systems, the UV–vis spectra of the decomposition products were different than those of the starting compounds indicating consecutive rather than reverse decomposition reactions of radicals. On the other hand, no such irreversible decompositions were observed for the compounds of **5bb** series, although in the presence of less than 1 equiv. of I_2_, the oxidation was reversed after 6 h at room temperature. Our attempt of structural characterization of the radical [**5bb‐1**]^+•^ obtained by oxidation of **5bb‐1** with 2 equiv. of iodine under control of the vis‐NIR spectroscopy (Figure S147, Supporting Information), resulted in an apparently unaltered molecular structure ([**5bb‐1**]I_2_) of the starting compound with I_2_ present in the crystal lattice, though badly disordered among several crystallographic sites. Seemingly, I^−^ was able to reduce the radical under the condition of crystallization (DCM/hexane, room temperature) even in the presence of an excess of the I_2_ oxidant. Apparently, the **5bb** dimers were stable also in the higher oxidation states. Addition of heterogeneous reductant (solid KO_2_) to the sample which was previously oxidized with 4 equiv. of BAHA and kept for several hours at room temperature, resulted in almost quantitative reverse of the oxidation and restoration of the spectrum of the starting species (Figure S157, Supporting Information). Similarly, the sample of **5bb‐3** oxidized with 3.5 equiv. of BAHA was kept in solution for ≈16 h at room temperature without absorbance decrease in the region of 1000–2500 nm (Figure S159, Supporting Information).

#### 2.6.4 Ground State of the Dications

**Figure 8:**
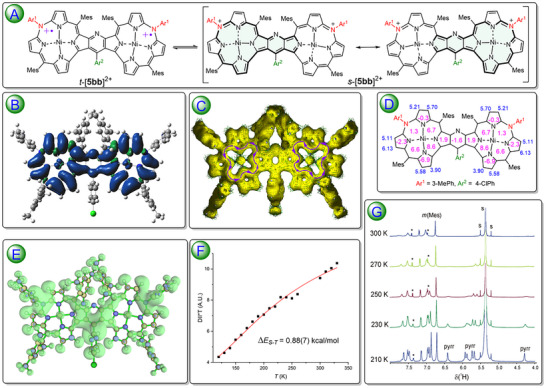
A) Schematic Lewis representation of the spin equilibrium of s‐[**5bb**]^2+^ and t‐[**5bb**]^2+^. The 24πe delocalization paths in the canonical structures of s‐[**5bb**]^2+^ are marked with bold lines. B) Calculated spin distribution within t‐[**5bb‐1**]^2+^ (isovalue 0.0004). C) AICD plot (0.025 isovalue) for s‐[**5bb‐1**]^2+^ with purple curved arrows indicating the counterclockwise direction of the induced current. D) GIAO‐calculated chemical shifts (blue numbers) and NICS(0) values (rose numbers) for s‐[**5bb‐1**]^2+^. E) Positive charge distribution (green surface, isovalue 0.15) over the s‐[**5bb‐1**]^2+^ skeleton derived from the NBO approach applied to DFT‐optimized structure. F) Plot of the temperature dependence of the product of temperature and doubly integrated intensity of ESR signals (T*DII) for the solid [**5bb‐2**]^2+^ (black squares) and best fit to the Bleaney‐Bowers equation^[^ ^48^, ^49^, ^50^ ^]^ (red line). G) Low‐field region of the ^1^H NMR spectra recorded for the solution of [**5bb‐2**]^2+^ in CD_2_Cl_2_ at specified temperatures. The signals of residual tris(bromophenyl)amine, i.e., reduced form of BAHA are marked with asterisks; s, CHDCl_2_ signal.

The DFT calculations revealed triplet ground states for [**5aa**]^2+^ and [**5ab**]^2+^ with T‐S energy gap of 3.3 and 2.4 kcal mol^−1^, respectively in line with ESR signal observation in the frozen solution at low temperatures. Conversely, the calculated energy of s‐[**5bb‐1**]^2+^ was 0.2 kcal mol^−1^ lower than that of t‐[**5bb‐1**]^2+^ (**Figure** **8**A). However, due to the limited accuracy of predicting spin‐state energetics, such a small calculated Δ*E*_S‐T_ gap can only be used as qualitative indicators of near‐degeneracy rather than quantitative estimates. The diradical index of [**5bb‐1**]^2+^ at UB3LYP/gen(6‐31G(d,p)/LANL2DZ level was calculated to be 0.00, indicating the negligible contribution of the paramagnetic open shell resonance structures for the singlet dication. The solid‐state VT ESR for [**5bb‐2**]^2+^ revealed an increase of the DII*T value along with temperature, and the Bleaney‐Bowers equation fitting gave an S‐T energy gap of 0.88±0.07 kcal mol^−1^ (Figure 8F). For the estimated energy gap and the singlet ground state, the Boltzmann distribution predicted less than 0.3% of the [**5bb‐2**]^2+^ population in the paramagnetic state at 77 K and ≈4% at 140 K, in line with the observed disappearance of the ESR signal in the frozen solutions of [**5bb‐2**]^2+^. The ^1^H NMR spectrum of [**5bb‐2**]^2+^ in CD_2_Cl_2_ solution at room temperature (Figure 8G; Figure S138, Supporting Information) indicated the presence of several broadened signals of protons belonging to the *meso‐*substituents, in line with low spin densities at these positions predicted by calculations for the dication in the triplet state (Figure 8B). Conversely, no pyrrole β‐proton signals were observed at 300 K due to relatively high electron spin density at pyrrole carbons. Lowering the solution temperature down to ≈250 K gave rise to the appearance of the pyrrole signals that became reasonably sharp at 210 K, allowing observation of the correlations in a 2D COSY experiment. Thus, under such conditions, the dications of **5bb** in the singlet state dominated (89% according to the Boltzmann distribution) and the chemical exchange between s‐[**5bb‐2**]^2+^ and t‐[**5bb‐2**]^2+^ was slow at the proton NMR timescale.

Analysis of the magnetic properties strongly suggested paratropic character of the macrocyclic subunits in the singlet ground state of these dications. The ^1^H NMR chemical shifts of the pyrrole protons, both experimental (from δ 4.6 to 6.4 ppm) and GIAO‐calculated (δ 3.9–6.1 ppm) suggested weak antiaromaticity, while the NICS(0) indices calculated inside the porphyrinoid rings were all positive, implying the presence of a paratropic current on the bis(porphyrinoid) subunits (Figure 8D). Significantly, the NICS(0) at the center of bridging pyridine (−1.6 ppm) suggested lack of a local diatropic current on this part of the molecule. Moreover, the AICD analysis revealed the presence of the induced current and its counter‐clockwise direction (Figure 8C) proving the paratropic character of s‐[**5bb**]^2+^. The paratropic current seemed to be weak when compared to the norcorrole systems and was confined separately to each of the subunits, not involving the whole fused bis(porphyrinoid) or the bridging pyridine. Two equivalent resonance Lewis structures that are isoelectronic with 44πe system of the dication diradical t‐[**5bb**]^2+^ (Figure 8A) can be drawn to account for the observed weak antiaromaticity of s‐[**5bb**]^2+^. Both include 24πe in the conjugation paths obeying the 4*n* antiaromatic rule, though displaced on the complementary regions of the skeleton while the rest constitutes a 20πe system with a broken conjugation. Thus, both the spin pairing as well as antiaromaticity required conjugation reaching beyond the single azacorrole subunit. We also analyzed the charge distribution on s‐[**5bb**]^2+^ by natural bond orbital (NBO) approach finding out that the net positive excesses were placed near the *meso*‐N10(10′) environments in both subunits which likely were the oxidations sites (Figure 8E).^[^ ^34^ ^]^ However, a comparable excess of the positive charge appeared on the bridging pyridine, fused pyrroles and *meso*‐C5(5′) suggesting conjugation within the macrocyclic subunits rather than disjunction occurring at the oxidation sites due to lack of the electron lone pairs at N10 and N10'. Almost identical positive charge displacement were obtained for t‐[**5bb**]^2+^ (Figure S123, Supporting Information) suggesting that the electron density distributions were the same in this system regardless of the spin ground state.

## 3 Conclusion

Extensions of the π‐electron system of the aromatic azacorroles alters considerably the electronic structure of the macrocyclic subunits leading to the changes in the spectroscopic properties of the neutral, mono‐, and dicationic forms. The substitution pattern, reflecting the applied method of synthesis of the pyridine‐fused bis(azacorrole) strongly affects the structure of the dimers determining deviation from planarity. These observations are in line with the delocalization of the electron density onto the whole dimer. Conversely, in the heterodimers consisting of pyridine‐fused aromatic azacorrole and antiaromatic norcorrole, the macrocyclic subunits retain most of their individual characteristics and electron density delocalization is confined separately to each of the macrocycles. The isomeric pyridine‐fused bis(mesoaryl‐azacorrolatonickel(II)) systems **5aa** and **5ab** can be obtained by the iodosobenzene‐stimulated oxidative insertion of aniline derivative into the fused bis(norcorrolatonickel(II)). Apart from the mixture of isomers, the reaction gives rise to norcorrole‐azacorrole and azacorrole‐oxacorrole heterodimers. Conversely, the highly regio‐ and chemoselective Hantzsch‐type pyridine annulation of 3‐amino‐10‐arylazacorrolatonickel(II) with arylaldehyde results in the symmetric bis(azacorrole)s **5bb**. The redox studies indicate strong electron‐donating properties of the azacorrole dimers. Mono‐, di‐ and tris‐oxidized forms of the homodimers are characterized by electronic transitions of extremely low energy, giving rise to the absorption bands in the NIR region beyond 2000 nm. The spectroscopic and magnetic properties of the oxidized species depend on the geometry of the dimers controlling the interaction of the subunits. A singlet ground state for doubly‐oxidized homodimers of **5bb** series allows observation of paratropic current in the diamagnetic dicationic species. The two‐electron oxidation has been shown to be sufficient to convert two pyridine‐fused aromatic macrocycles comprising jointly 46 π‐electrons into the 44πe system of two antiaromatic azacorrole subunits. Such an aromatic‐antiaromatic switching or remarkably narrow HOMO‐LUMO gaps in the oxidized forms has not been observed for the monomeric azacorrole or bis(norcorrole) systems reported previously.^[^ ^33^, ^34^ ^]^ Thus, the current systems constitute a new class of the porphyrinoid oligomers that are qualitatively different from their predecessors. A broad scope of substituents that can be introduced to the system, allows a fine‐tuning of the spectral and redox properties of the dimers or their further extension that may be exploited in the construction of new NIR‐absorbing materials of high spin and high charge.

## Conflict of Interest

The authors declare no conflict of interest.

## Supporting information



Supporting Information

## Data Availability

The data that support the findings of this study are available in the supplementary material of this article.
