# Supplementary material for: Pyridine‐Fused Bis(azacorrole)s: Easily Accessible NIR III Absorbing Cation Radicals and Biradicaloids of Antiaromatic Ground State
Source: Adv Sci (Weinh). 2025 Mar 28;12(21):2416223. doi: 10.1002/advs.202416223 (PMC12140311; doi:10.1002/advs.202416223)
Supplement: Supplementary file 1 — Supporting Information [file ADVS-12-2416223-s001.pdf]

ADVANCED SCIENCE Open Access

Supporting Information

for *Adv. Sci.*, DOI 10.1002/advs.202416223

Pyridine-Fused Bis(azacorrole)s: Easily Accessible NIR III Absorbing Cation Radicals and Biradicaloids of Antiaromatic Ground State

Sha Li, Shaowei Zhang, Xiaofang Li, Oskar Smaga, Kinga Szydełko, Miłosz Pawlicki and Piotr J. Chmielewski**

Table of Contents

1. General Methods and Instrumentation
2. Synthesis and Characterization of Azacorrole-Norcorrole Derivatives **4**
3. Synthesis and Characterization of pyridine-fused dimers **5aa**, **5ab**, **6a**, and **6b**
4. NMR Spectra for **5aa**, **5ab**, **4a**, **4b**, **6a**, and **6b**
5. Mass Spectra for **5aa**, **5ab**, **4a**, **4b**, **6a**, and **6b**
6. Optical Spectra for **5aa**, **5ab**, **4a**, **4b**, **6a**, and **6b**
7. Synthesis of NO_2-azacorrole: **2-NO$_2$**(3-MePh) and **2-NO2**(3-MeOPh)
8. Synthesis of NH_2-azacorrole **2-NH$_2$**(3-MePh) and **2-NH$_2$**(3-MeOPh)
9. General Procedure of Synthesis and Characterization of Pyridine-fused Bis(azacorrole) from 3-Amino-10-azacorrole
10. NMR Spectra of **5bb** and Precursors
11. Mass Spectra for **5bb** and Precursors
12. UV-vis Spectra for **5bb** and Precursors
13. Electrochemical Data
14. Calculation Results, Part 1
15. ESR Spectra of the Oxidized Species and VT Experiments
16. Chirality of **5aa** and [**5aa**]$^{•+}$
17. Spectrophotometric Titrations of Dimers with Oxidants
18. DFT and TD DFT Calculation Results, Part 2
19. Frontier Orbitals of Cation Radicals [**5aa**], [**5ab**], and [**5bb-1**]
20. Crystallographic Data
21. References

1. General Methods and Instrumentation

Commercial reagents were used without further purification. Solvents were freshly distilled from the appropriate drying agents or purified under nitrogen with the mBraun MBSPS-800 before use. The analytical TLCs were performed with silica gel 60 F254 plates. Column chromatography was performed by using silica gel 60 (200-300 mesh ASTM). The NMR spectra were recorded on a Bruker Avance II or Jeol JNM-ECZR 500 MHz spectrometers, operating at 500 MHz for ^{1}H and 125 MHz for ^{13}C, and Bruker Avance II spectrometer operating at 600 MHz for ^{1}H and 150 MHz for ^{13}C. TMS was used as an internal reference for ^{1}H and ^{13}C chemical shifts and $CDCl_3$ was used as solvent. Standard pulse programs from the Bruker or Jeol libraries were used for 2D experiments. Mass spectrometry measurements were conducted by using the electrospray ionization technique on a Shimadzu IT-TOF LCMS or Finnigan LCQ Advantage MAX mass spectrometer. Absorption UV/Vis/NIR spectra were recorded by using a Varian Cary 50 Bio and Jasco V-770 spectrophotometers. EPR spectra (X-band) were recorded on a Bruker ELEXSYS E500 spectrometer and were simulated using WinEPR Simfonia v.1.25 (shareware version) by Bruker. The applied dichloromethane was freshly distilled over calcium hydride. HPLC separations were carried out by means of Chirex 3014 analytical column (25 cm length, 4.6 mm i.d.) packed with 5 μm silica gel coated with covalently bound (*S*)-valine and (*R*)-1-(α-naphthyl)ethylamine using Shimadzu chromatographic system or Merck-Hitachi LaChrom series connected to a flow-cell mounted on Jasco J-1500 spectropolarimeter. HPLC-grade hexane was used. Circular dichroism spectra were obtained directly in the flow cell by means of pseudo stopped-flow technique using a Jasco J-1500 spectropolarimeter. Electrochemical measurements were performed by means of Autolab (Metrohm) potentiostat/galvanostat system for dichloromethane solutions with a glassy carbon, a platinum wire, and Ag/AgCl as the working, auxiliary, and reference electrodes, respectively. Tetrabutylammonium hexafluorophosphate was used as a supporting electrolyte. The potentials were referenced with the ferrocene/ferrocenium couple used as internal standard.

Crystals were obtained by slow diffusion of the benzene solution into hexane (**5aa**) and by slow diffusion of the dichloromethane solution into hexane (**5ab**, **5bb-1**, **[5bb-1]**I2). Crystal data for the crystals reported in this paper were collected at low temperatures (100 K) using XtaLAB Synergy R, HyPix diffractometer with Cu *Kα* radiation (λ = 1.54184 Å). Data reduction and analysis were carried out with the SAINT[1] and CrysAlisPRO programs[2]. The structure was solved by using the SHELXT[3] and refined by the full-matrix least-squares method on all F^2 data using the SHELXL[4]. All hydrogen atoms, including those located in the difference density map, were placed in calculated positions and refined as the riding model. See Table S28-S31 for detailed data. The full structure and diffraction data are available in Cambridge Crystallographic Data Center under the CCDC numbers 2369186 (**5aa**), 2369275 (**5ab**), 2369438 (**5bb-1**) and 2369429 ([**5bb-1**]I2).

Computational methods

Density functional theory (DFT) calculations were performed by using the Gaussian 16 program[5]. DFT geometry optimizations were carried out in the unconstrained C_1 symmetry by using the X-ray structures, molecular mechanics, or semiempirical models as starting geometries. DFT geometries were refined to meet standard convergence criteria, and the existence of a local minimum was verified by a normal mode frequency calculation. DFT calculations were performed by using the hybrid B3LYP functional,[6-8] and the 6-31G(d,p) basis set with a LAN2DZ pseudopotential applied for nickel(II) center. The electronic spectra were simulated by means of time-dependent density functional theory (TD-DFT) using the Tamm-Dancoff approximation for 25 states. For TD calculations, the polarizable continuum model of solvation was used (PCM, standard chloroform parametrization). The electronic transitions and UV/Vis/NIR spectra were analyzed by means of the GaussSum program.[9] The transitions were convoluted by Gaussian curves with 2000 cm^{-1} half line width. ^{1}H and ^{13}C NMR chemical shifts as well as nucleus-independent chemical shifts (NICS(–1,0,1)) were

calculated by means of GIAO/B3LYP/6-31G(d,p)/LANL2DZ procedure included in the Gaussian program. The AICD plots were generated from the calculations performed with G16 software and CSGT approach with the IOP(10/93=1) parametrization. Obtained results were post-calculation edited with the AICD-2.0.0 software package provided by the Authors.[10]

The diradical/diradicaloid character of [**5bb-1**]$^{2+}$ at UB3LYP/gen(6-31G(d,p) (C H N Cl) and LANL2DZ (Ni)) with a guess=(mix,always) approach was calculated to be 0.00, indicating the negligible contribution of open shell resonance structures for the dication. Spin density and electrostatic potential maps were generated with Multiwfn.[11-13]

2. Synthesis and Characterization of Azacorrole-Norcorrole Derivatives **4**

Synthesis of precursors. Starting complexes **1**, **1**-NO_2, and dimer **3** were obtained as described previously.[14-16]

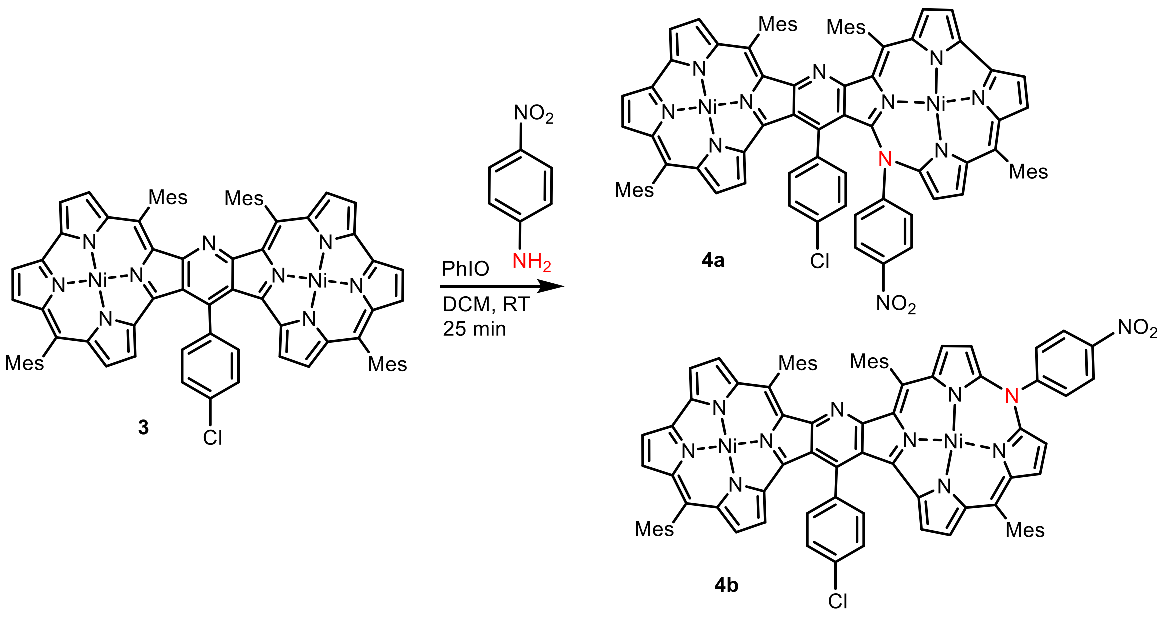

A solution of **3** (22 mg, 0.017 mmol) and 4-nitroaniline (3.5 mg, 0.026 mmol) in 5 ml CH_2Cl_2 was stirred at room temperature for 25 min in the presence of PhIO (15 mg, 0.068 mmol). The reaction mixture was then passed through a short silica gel column with CH_2Cl_2 as eluent to collect all the moveable fraction, and then the solvent was evaporated under vacuum. The residue was purified by silica preparative thin layer plate (1 mm, 20 x 20 cm) with petroleum ether/CH_2Cl_2 (*v*/*v* = 3:1) as eluent to afford recovered **3** (5.7 mg), **4a** (4.3 mg, 24% yield (based on consumed **3**) and **4b** (3.4 mg, 19% yield, based on consumed **3**).

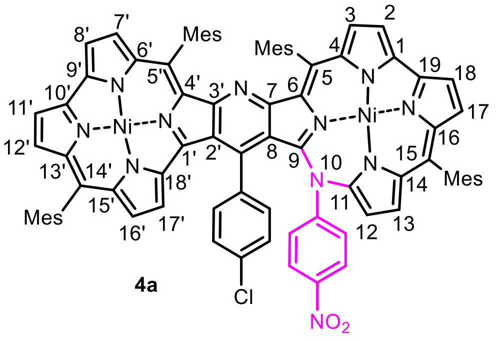

Selected data for **4a**: ^{1}H NMR (600 MHz, $CDCl_3$, 300 K): δ = 8.07 (m(br), 2H, 4-NO_2Ph), 7.89 (d, ^3J = 4.3 Hz, 1H, 18-pyrr), 7.78 (d, ^3J = 4.5 Hz, 1H, 2-pyrr), 7.62 (d, ^3J = 4.9 Hz, 1H, 13-pyrr), 7.59 (d, ^3J = 4.3 Hz, 1H, 17-pyrr), 7.00 (s, 2H, 15-*m*-Mes), 6.78 (m, 6H, 3-pyrr, 12-pyrr, 5-*m*-Mes, 4-NO_2Ph), 6.71 (m, 2H, 4-ClPh), 6.28 (s, 2H, 14'-*m*-Mes), 6.25 (s, 2H, 5'-*m*-Mes),

5.86 (m, 2H, 4-ClPh), 2.70 (s, 6H, 14'-*o*-Me), 2.58 (s, 3H, 5-*p*-Me), 2.42 (s, 3H, 15-*p*-Me), 2.35 (d, ^{3}J = 4.7 Hz, 1H, 12'-pyrr), 2.32 (s, 6H, 5'-*o*-Me), 2.15 (d, ^{3}J = 4.7 Hz, 1H, 11'-pyrr), 2.07 (d, ^{3}J = 3.9 Hz, 1H, 8'-pyrr), 2.02 (s, 3H, 5'-*p*-Me), 1.83 (s, 3H, 14'-*p*-Me), 1.78 (s, 6H, 15-*o*-Me), 1.60 (d, ^{3}J = 4.1 Hz, 1H, 16'-pyrr), 1.52 (d, ^{3}J = 3.9 Hz, 1H, 7'-pyrr), 1.30 (s, 6H, 5-*o*-Me), 0.11 (d, ^{3}J = 4.1 Hz, 1H, 17'-pyrr). ^{13}C NMR (150 MHz, $CDCl_3$, 300 K) δ_C = 177.6, 170.2, 157.7, 157.0, 156.8, 153.9, 152.7, 149.11, 149.05, 148.8, 147.3, 147.1, 147.0, 146.2, 143.8, 142.9, 140.2, 138.8, 138.0, 137.7, 136.9, 136.2, 135.8, 135.5, 135.3, 134.9, 134.8, 134.7, 134.4, 134.2, 133.8, 133.3, 132.8, 132.1, 131.1, 130.7, 130.4, 129.0, 128.6, 128.0, 127.7, 127.5, 127.4, 126.73, 126.70, 126.1, 125.5, 125.4, 123.5, 121.6, 121.5, 119.5, 118.5, 117.8, 117.3, 116.6, 114.9, 114.3, 112.5, 29.7, 22.7, 21.9, 21.2, 21.1, 20.6, 20.1, 18.2, 17.8. UV-vis (CH_2Cl_2) λ_{max}/nm (logε): 284 (4.65), 388 (sh), 410 (4.86), 446 (4.87), 554 (4.44), 590 (4.39), 687 (4.28), 733 (4.25), 810 (sh). ESI-HRMS calc. for [$C_{85}H_{64}ClN_{11}Ni_2O_2$] $(M)^+$: 1423.3590, Found 1423.3711; for [$C_{85}H_{65}ClN_{11}Ni_2O_2$] $(M+H)^+$: 1424.3668, Found 1424.3734.

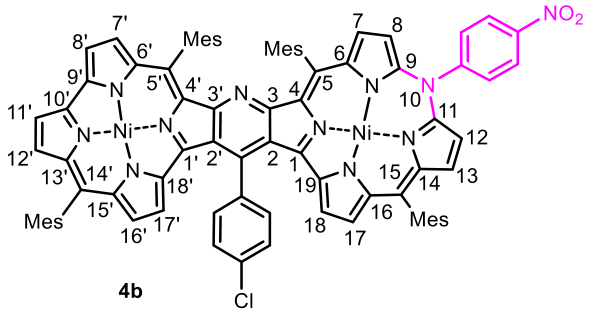

4b

Selected data for **4b**: ^{1}H NMR (600 MHz, $CDCl_3$, 300 K): δ = 8.53 (m, 2H, 4-NO_2Ph), 7.96 (d, ^{3}J = 4.8 Hz, 1H, 13-pyrr), 7.90 (m, 2H, 4-NO_2Ph), 7.31 (d, ^{3}J = 4.6 Hz, 1H, 17-pyrr), 7.20 (d, ^{3}J = 4.9 Hz, 1H, 7-pyrr), 7.16 (m, 2H, 4-ClPh), 7.02 (d, ^{3}J = 4.8 Hz, 1H, 12-pyrr), 6.99 (s, 2H, 15-*m*-Mes), 6.96 (m, 2H, 4-ClPh), 6.81 (d, ^{3}J = 4.9 Hz, 1H, 8-pyrr), 6.48 (s, 2H, 5-*m*-Mes), 6.37 (s, 2H, 5'-*m*-Mes), 6.32 (s, 2H, 14'-*m*-Mes), 5.28 (d, ^{3}J = 4.6 Hz, 1H, 18-pyrr), 2.74 (s, 6H, 14'-*o*-Me), 2.50 (s, 6H, 5'-*o*-Me), 2.48 (d, ^{3}J = 4.7 Hz, 1H, 12'-pyrr), 2.40 (s, 3H, 15-*p*-Me), 2.34 (s, 3H, 5-*p*-Me), 2.27 (d, ^{3}J = 4.7 Hz, 1H, 11'-pyrr), 2.15 (m, 4H, 5'-*p*-Me + 8'-pyrr), 1.87 (s, 3H, 14'-*p*-Me), 1.71 (d, ^{3}J = 4.0 Hz, 1H, 16'-pyrr), 1.70 (s, 6H, 15-*o*-Me), 1.37 (d, ^{3}J = 3.9 Hz, 1H, 7'-pyrr), 1.23 (s, 6H, 5-*o*-Me), 0.32 (d, ^{3}J = 4.0 Hz, 1H, 17'-pyrr). ^{13}C NMR (150 MHz, $CDCl_3$, 300 K) δ_C = 177.2, 170.5, 155.5, 155.4, 153.7, 151.5, 151.2, 149.4, 148.72, 148.69, 148.6, 147.0, 144.5, 143.1, 142.2, 141.3, 139.8, 139.0, 138.1, 137.7, 136.8, 136.6, 136.3, 135.2, 135.04, 135.00, 134.8, 134.4, 134.2, 134.1, 134.0, 133.58, 133.56, 133.3, 132.6, 131.2, 131.0, 130.9, 130.4, 129.92, 129.89, 128.8, 128.7, 128.5, 128.0, 127.7, 127.62, 127.58, 126.9, 126.8, 126.4, 126.4, 124.6, 124.3, 124.0, 121.6, 121.2, 120.3, 120.2, 118.7, 115.7, 114.7, 113.1, 111.1, 77.2, 77.0, 76.8, 68.2, 21.6, 21.3, 21.2, 20.7, 20.6, 20.2, 18.3. UV-vis (CH_2Cl_2) λ_{max}/nm (logε): 298 (4.55), 365 (4.69), 414 (4.84), 459 (4.84), 520 (4.58), 587 (4.34), 684 (4.20), 800 (sh). ESI-HRMS calc. for [$C_{85}H_{64}ClN_{11}Ni_2O_2$] $(M)^+$: 1423.3590, Found 1423.3711; for [$C_{85}H_{65}ClN_{11}Ni_2O_2$] $(M+H)^+$: 1424.3668, Found 1424.3802.

3. Synthesis and Characterization of pyridine-fused dimers **5aa**, **5ab**, **6a**, and **6b**

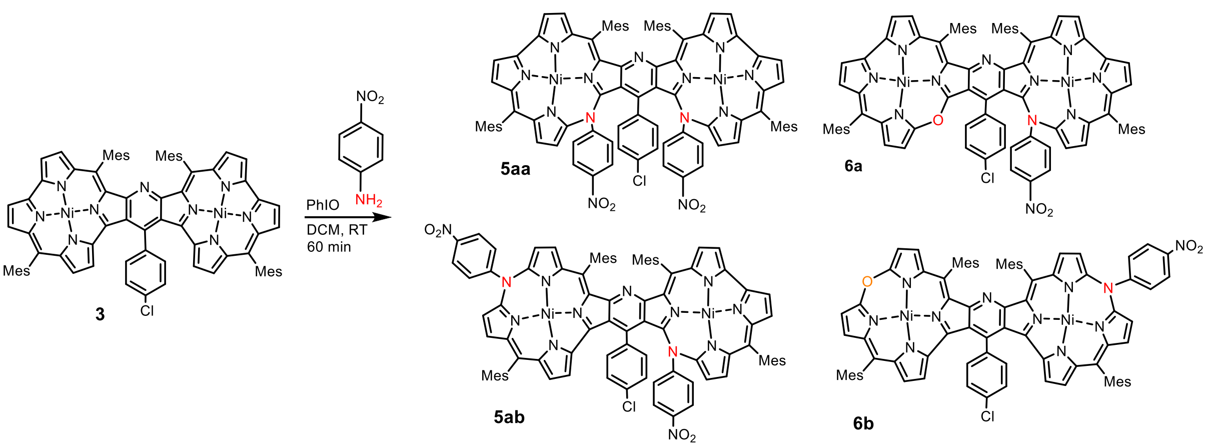

A solution of **3** (22 mg, 0.017 mmol) and 4-nitroaniline (9.3 mg, 0.068 mmol) in 5 ml CH_2Cl_2 was stirred at room temperature for 60 min in the presence of PhIO (15 mg, 0.068 mmol). After that, the reaction mixture was passed through a short silica gel column with CH_2Cl_2 as eluent to collect all the moveable fraction, then the solvent was evaporated under vacuum. The residue was purified by silica preparative thin layer plate (1 mm, 20 x 20 cm) with petroleum ether/CH_2Cl_2 (*v*/*v* = 2:1) as eluent to afford **6a** (4.4 mg, 18% yield), **6b** (4.2 mg, 17% yield), **5aa** (4.0 mg, 15% yield), and **5bb** (5.5 mg, 21% yield).

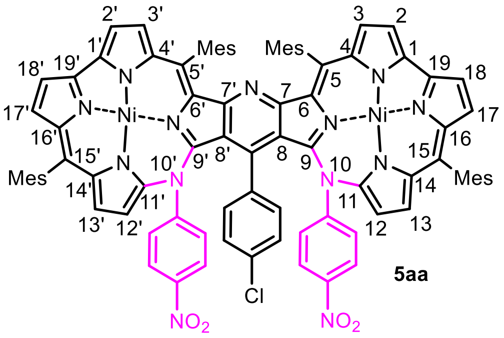

Selected data for **5aa**: ^{1}H NMR (600 MHz, $CDCl_3$, 300 K): δ = 8.04 (d, ^3J = 4.4 Hz, 2H, 18,18'-pyrr), 7.99 (d, ^3J = 4.1 Hz, 2H, 3,3'-pyrr), 7.79 (d, ^3J = 4.6 Hz, 2H, 13,13'-pyrr), 7.73 (d, ^3J = 4.4 Hz, 2H, 17,17'-pyrr), 7.65 (m(br), 4H, 4-NO_2Ph), 7.35 (d, ^3J = 4.1 Hz, 2H, 2,2'-pyrr), 7.19 (s, 2H, *m*-Mes), 7.17 (m, 2H, 4-ClPh), 7.14 (s, 2H, *m*-Mes), 7.06 (s, 2H, *m*-Mes), 6.98 (d, ^3J = 4.6 Hz, 2H, 12,12'-pyrr), 7.17 (m(br), 4H, 4-NO_2Ph), 6.69 (s, 2H, *m*-Mes), 6.31 (m, 2H, 4-ClPh), 2.48 (s, 6H, *p*-Me), 2.46 (s, 6H, *p*-Me), 2.08 (s, 6H, *o*-Me), 1.96 (s, 6H, *o*-Me), 1.76 (s, 6H, *o*-Me), 1.29 (s, 6H, *o*-Me). ^{13}C NMR (150 MHz, $CDCl_3$, 300 K) δ_C = 154.5, 146.6, 145.4, 144.4, 141.9, 140.4, 139.1, 136.9, 136.7, 136.6, 136.5, 135.2, 135.1, 134.5, 133.7, 133.4, 133.1, 132.5,132.0, 131.7, 130.5, 129.8, 129.7, 129.2, 127.8, 127.7, 126.5, 126.3, 125.9, 125.35, 123.0, 122.9, 121.6, 116.3, 115.1, 111.7, 110.3, 20.4, 20.4, 20.1, 19.6, 19.1, 18.4. UV-vis (CH_2Cl_2) λ_{max}/nm (logε): 398 (4.83), 463 (4.88), 526 (4.51), 620 (4.24), 681 (4.24), 742 (4.72). ESI-HRMS calc. for [$C_{91}H_{68}ClN_{13}Ni_2O_4Na$] $(M+Na)^+$: 1583.3793, Found 1583.3700.

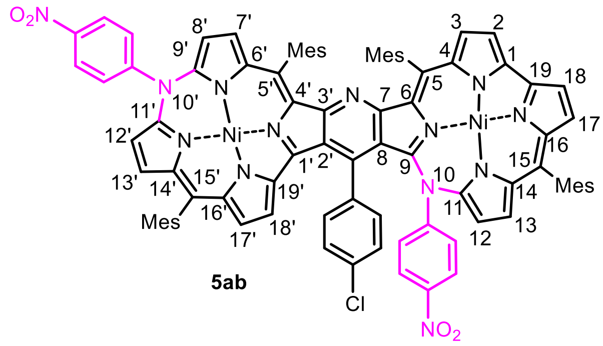

Selected data for **5ab**: ^{1}H NMR (600 MHz, $CDCl_3$, 300 K): δ = 8.63 (m(br), 2H, 4-ClPh), 8.12 (d, ^3J = 4.7 Hz, 1H, 17-pyrr), 8.07 (br, 2H, 4-ClPh), 8.05 (d, ^3J = 4.3 Hz, 1H, 18'-pyrr), 7.97 (m(br), 2H, 10'-(4-NO_2Ph)), 7.95 (d, ^3J = 4.1 Hz, 1H, 3-pyrr), 7.85 (d, ^3J = 4.7 Hz, 1H, 13'-pyrr), 7.77 (d, ^3J = 4.4 Hz, 1H, 17'-pyrr), 7.76 (d, ^3J = 4.6 Hz, 1H, 7'-pyrr), 7.52 (br, 2H, 10-(4-NO_2Ph)), 7.51 (d, ^3J = 4.4 Hz, 1H, 13-pyrr), 7.30 (m(br), 4H, 10-(4-NO_2-Ph)+10'-(4-NO_2-Ph), 7.21 (d, ^3J = 4.7 Hz, 1H, 18-pyrr), 7.12 (s, 2H, *m*-Mes), 7.09 (br, 4H, 12'-pyrr, *m*-Mes, 8'-pyrr), 7.05 (br, 2H, *m*-Mes), 7.02 (d, ^3J = 4.1 Hz, 1H, 2-pyrr), 6.88 (br, 2H, *m*-Mes), 7.51 (d, ^3J = 4.4 Hz, 1H, 12-pyrr), 2.63 (s, 3H, *p*-Me), 2.56 (s, 3H, *p*-Me), 2.50 (s, 3H, *p*-Me), 2.47 (s, 3H, *p*-Me), 1.96 (s, 6H, *o*-Me), 1.82 (br, 6H, *o*-Me), 1.68 (s, 6H, *o*-Me), 1.52 (s, 6H, *o*-Me). ^{13}C NMR (150 MHz, $CDCl_3$, 300 K) δ_C = 155.4, 155.2, 149.0, 148.1, 147.5, 146.5, 143.3, 142.5, 142.3, 141.6, 140.8, 138.8, 138.6, 138.5, 138.0, 138.0, 137.4, 136.5, 136.3, 135.7, 135.2, 135.0, 134.92, 134.88, 134.2, 133.9, 133.8, 133.3, 133.0, 131.4, 131.0, 130.6, 130.5, 128.0, 127.5, 127.4, 126.8, 126.5, 126.3, 125.6, 124.6, 124.2, 124.0, 123.8, 120.7, 120.6, 117.5, 116.1, 115.5, 113.9, 112.3, 110.9, 22.2, 22.1, 21.6, 21.6, 21.4, 21.2, 21.0, 20.9. UV-vis (CH_2Cl_2) λ_{max}/nm (logε): 388 (sh), 410 (4.88), 441 (4.85), 464 (sh), 521 (4.60), 551 (4.40), 609 (4.26), 653 (4.34), 705 (4.71). ESI-HRMS calc. for [$C_{91}H_{68}ClN_{13}Ni_2O_4Na$] $(M+Na)^+$: 1583.3793, Found 1583.3700.

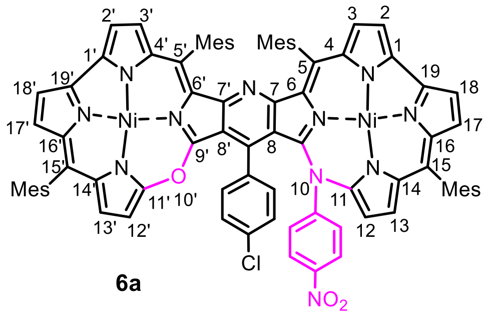

Selected data for **6a**: ^{1}H NMR (600 MHz, $CDCl_3$, 300 K): δ = 8.08 (d, ^3J = 4.4 Hz, 1H, 18-pyrr), 8.05 (m, 2H, 4-NO_2Ph), 7.98 (d, ^3J = 4.2 Hz, 1H, 2-pyrr), 7.86 (d, ^3J = 4.7 Hz, 1H, 13-pyrr), 7.79 (d, ^3J = 4.4 Hz, 1H, 17-pyrr), 7.75 (d, ^3J = 4.3 Hz, 1H, 18'-pyrr), 7.72 (d, ^3J = 4.1 Hz, 1H, 2'-pyrr), 7.60 (d, ^3J = 4.4 Hz, 1H, 13'-pyrr), 7.54 (d, ^3J = 4.2 Hz, 1H, 17'-pyrr), 7.53 (m, 2H, 4-ClPh), 7.31 (m, 2H, 4-NO_2Ph), 7.20 (m, 2H, 4-ClPh), 7.11 (s, 2H, 15-*m*-Mes), 7.072 (s, 2H, 15'-*m*-Mes), 7.066 (d, ^3J = 4.1 Hz, 1H, 7'-pyrr), 7.03 (d, ^3J = 4.2 Hz, 1H, 3-pyrr), 7.02 (d, ^3J = 4.6 Hz, 1H, 12-pyrr), 6.97 (s, 2H, 5'-*m*-Mes), 6.94 (s, 2H, 5-*m*-Mes), 6.90 (d, ^3J = 4.4 Hz, 1H, 12'-pyrr), 2.61 (s, 3H, 5-*p*-Me), 2.58 (s, 3H, 5'-*p*-Me), 2.50 (s, 3H, 15-*p*-Me), 2.47 (s, 3H, 15'-*p*-Me), 1.95 (s, 6H,

15-*o*-Me), 1.92 (s, 6H, 15'-*o*-Me), 1.73 (s, 6H, 5'-*o*-Me), 1.58 (s, 6H, 5-*o*-Me). ^{13}C NMR (150 MHz, $CDCl_3$, 300 K) δ_C = 157.5, 156.3, 149.9, 149.4, 149.3, 147.8, 147.5, 146.6, 143.6, 142.9, 142.5, 140.2, 138.2, 137.9, 137.8, 137.8, 137.0, 136.9, 136.7, 136.3, 136.1, 135.8, 135.7, 134.9, 134.5, 134.4, 134.3, 134.1, 134.0, 133.4, 133.2, 132.93, 132.90, 132.6, 130.7, 130.54, 130.45, 130.15, 130.09, 128.8, 128.4, 128.00, 127.96, 127.8, 127.3, 127.2, 125.4, 125.0, 124.9, 123.6, 123.4, 117.5, 116.8, 116.4, 115.8, 115.4, 112.2, 111.7, 105.8, 21.9, 21.8, 21.35, 21.28, 20.9, 20.7, 20.6, 20.5. UV-vis (CH_2Cl_2) λ_{max}/nm (logε): 281 (4.54), 388 (4.81), 433 (4.87), 528 (4.48), 594 (4.29), 615 (4.28), 635 (4.25), 674 (4.33), 737 (4.85). ESI-HRMS calc. for [$C_{85}H_{65}ClN_{11}Ni_2O_3$] $(M+H)^+$: 1438.3662, Found 1438.3671.

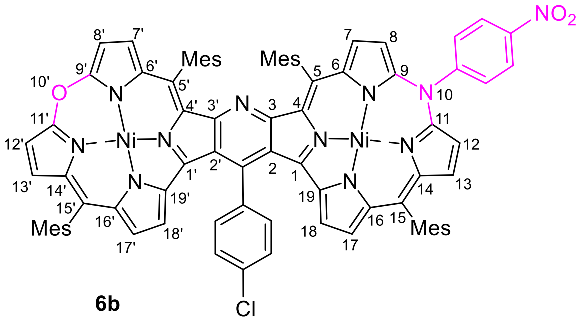

6b

Selected data for 6b: ^{1}H NMR (600 MHz, $CDCl_3$, 300 K): δ = 8.62 (m, 2H, 4-NO_2Ph), 8.18 (m, 2H, 4-ClPh), 8.17 (d, ^3J = 4.6 Hz, 1H, 13-pyrr), 8.06 (m, 2H, 4-NO_2Ph), 7.99 (m, 2H, 4-ClPh), 7.92 (d, ^3J = 4.5 Hz, 1H, 13'-pyrr), 7.70 (d, ^3J = 4.5 Hz, 1H, 17-pyrr), 7.66 (d, ^3J = 4.5 Hz, 1H, 12'-pyrr), 7.50 (d, ^3J = 4.6 Hz, 1H, 7-pyrr), 7.49 (d, ^3J = 4.4 Hz, 1H, 8'-pyrr), 7.39 (d, ^3J = 4.3 Hz, 1H, 17'-pyrr), 7.24 (d, 1H, 12-pyrr), 7.23 (d, ^3J = 4.4 Hz, 1H, 7'-pyrr), 7.11 (s, 2H, 15-*m*-Mes), 7.08 (s, 2H, 15'-*m*-Mes), 7.04 (d, ^3J = 4.6 Hz, 1H, 8-pyrr), 7.01 (s, 2H, 5'-*m*-Mes), 7.01 (s, 2H, 5-*m*-Mes), 6.22 (d, ^3J = 4.5 Hz, 1H, 18-pyrr), 5.85 (d, ^3J = 4.3 Hz, 1H, 18'-pyrr), 2.732 (s, 3H, 5-*p*-Me), 2.727 (s, 3H, 5'-*p*-Me), 2.50 (s, 3H, 15-*p*-Me), 2.48 (s, 3H, 15'-*p*-Me), 1.93 (s, 6H, 15-*o*-Me), 1.87 (s, 6H, 15'-*o*-Me), 1.77 (s, 6H, 5'-*o*-Me), 1.69 (s, 6H, 5-*o*-Me). ^{13}C NMR (150 MHz, $CDCl_3$, 300 K) δ_C = 138.7, 138.4, 138.00, 137.99, 137.9, 137.72, 137.70, 135.7, 135.0, 134.7, 133.8, 133.6, 132.6, 131.2, 130.8, 130.4, 130.3, 130.1, 127.8, 127.7, 127.6, 125.6, 125.2, 124.6, 124.2, 119.8, 119.2, 113.4, 110.7, 109.9, 105.6, 22.03, 22.00, 21.32, 21.29, 20.9, 20.72, 20.68, 20.5. UV-vis (CH_2Cl_2) λ_{max}/nm (logε): 330 (4.32), 392 (4.61), 462 (4.69), 548 (4.30), 615 (4.29), 615 (4.42), 639 (4.36), 677 (4.13), 742 (4.02). ESI-HRMS calc. for [$C_{85}H_{65}ClN_{11}Ni_2O_3$] $(M+1)^+$: 1438.3662, Found 1438.3666.

4. NMR spectra for **5aa**, **5ab**, **4a**, **4b**, **6a**, and **6b**

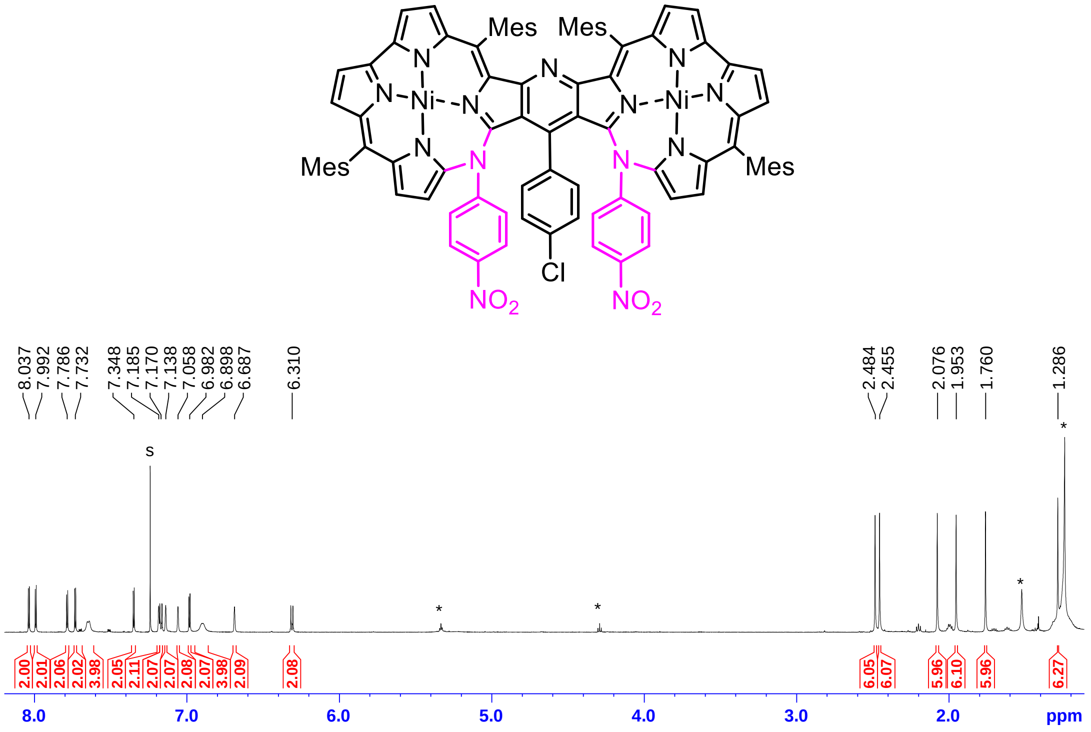

Figure S1. ^{1}H NMR spectrum (600 MHz, $CDCl_3$, 300 K) of **5aa**.

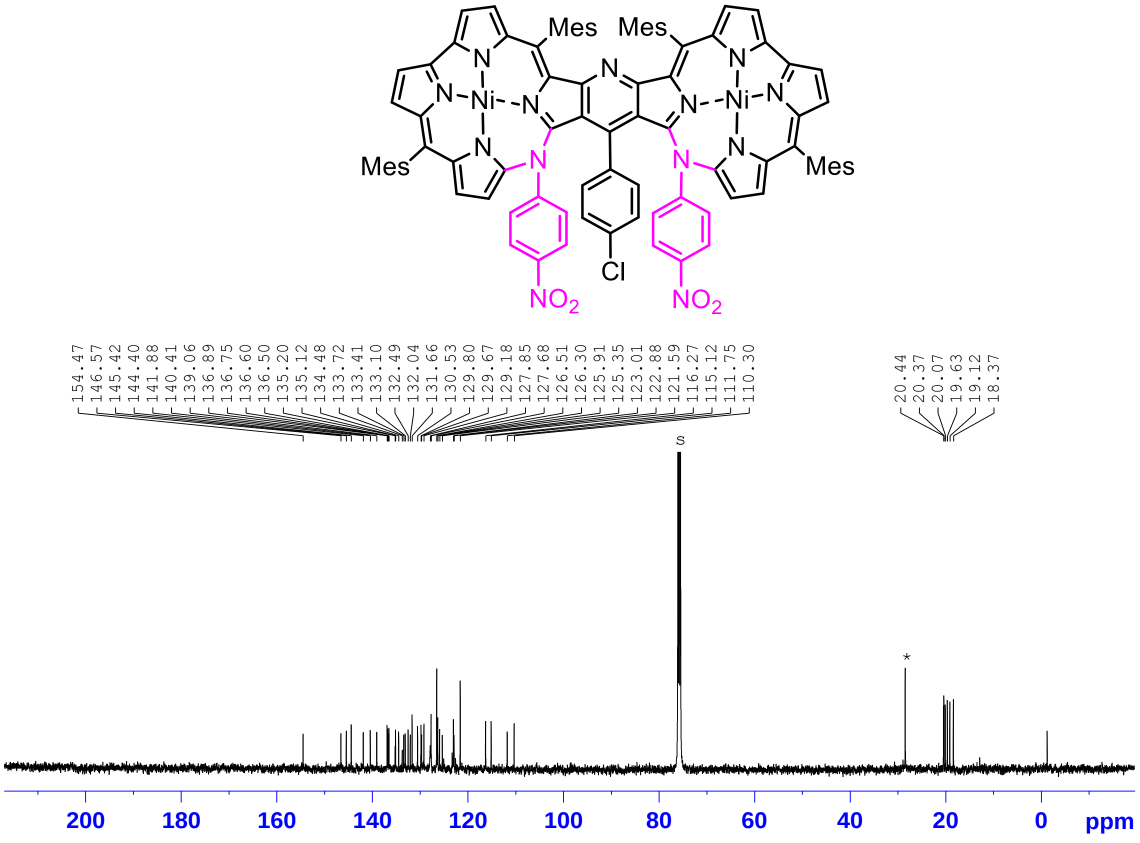

Figure S2. ^{13}C NMR spectrum (150 MHz, $CDCl_3$, 300 K) of **5aa**.

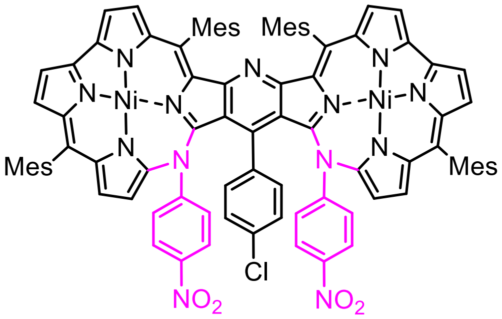

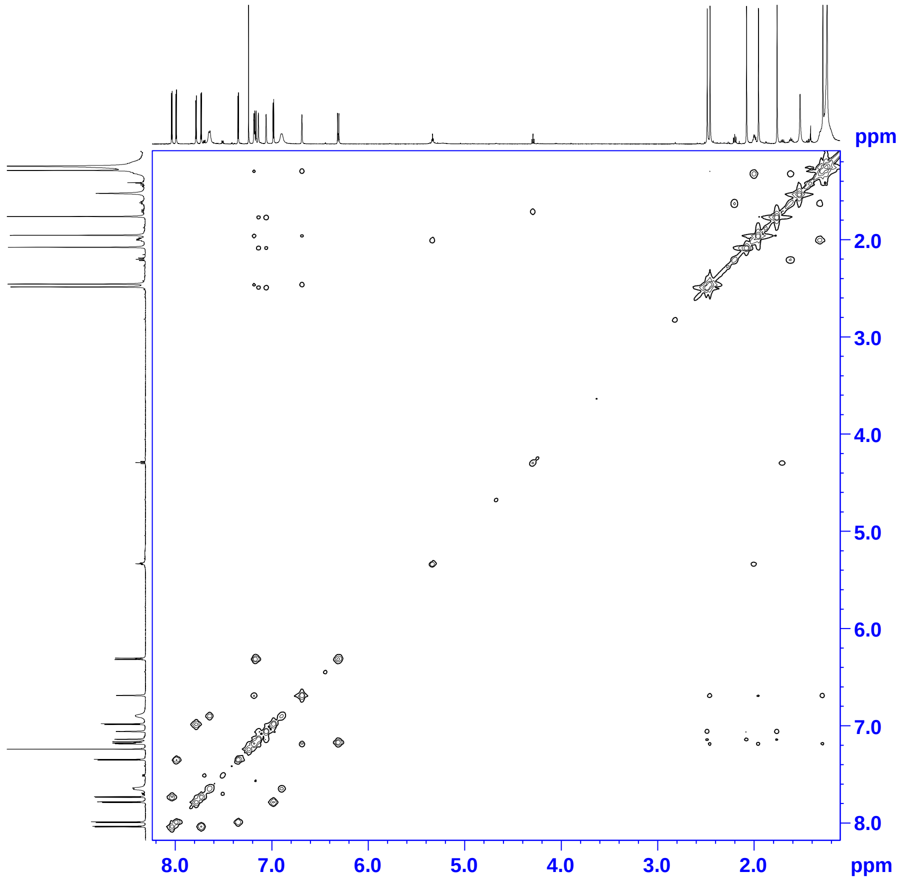

Figure S3. $^{1}H,^{1}H$ COSY spectrum (600 MHz, $CDCl_3$, 300 K) of **5aa**.

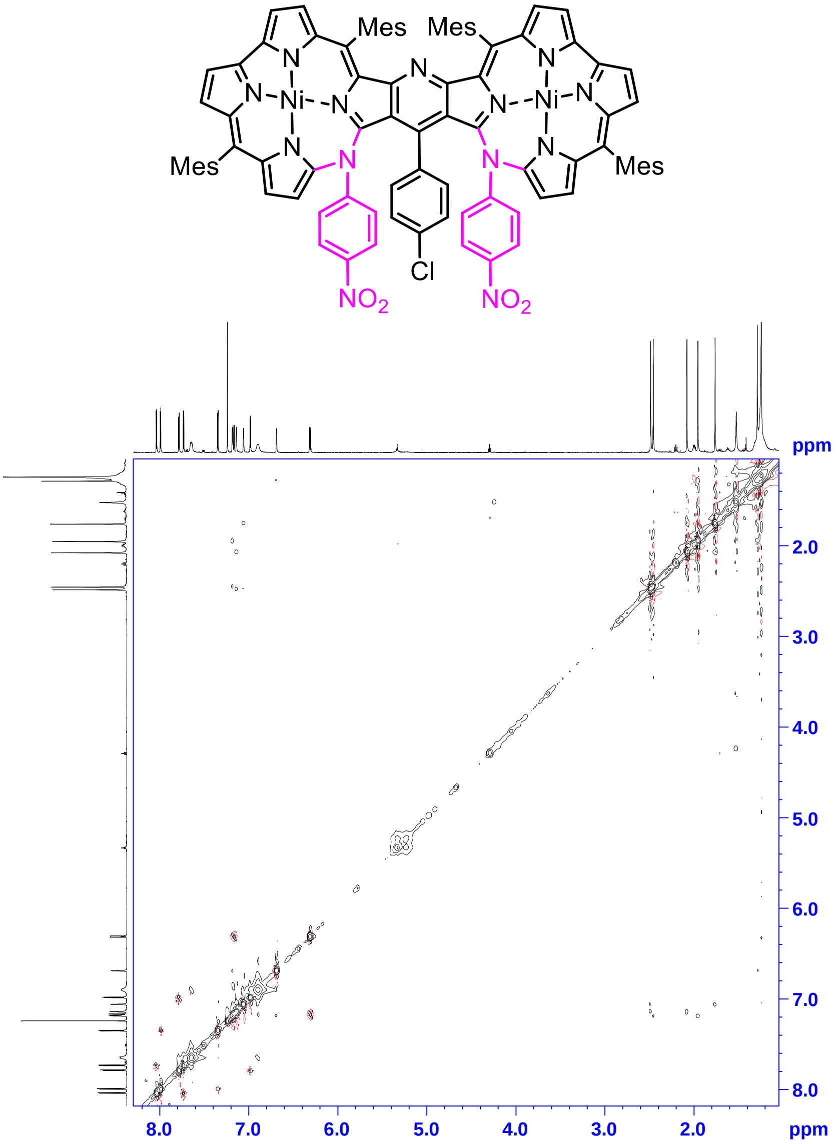

Figure S4. ^{1}H,^{1}H NOESY spectrum (600 MHz, $CDCl_3$, 300 K) of **5aa**.

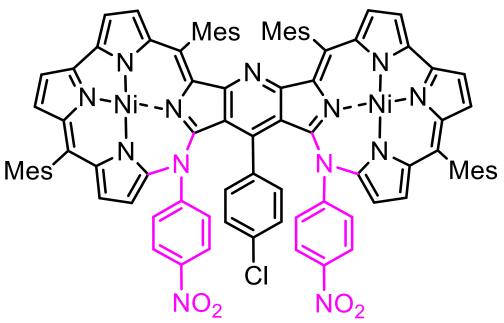

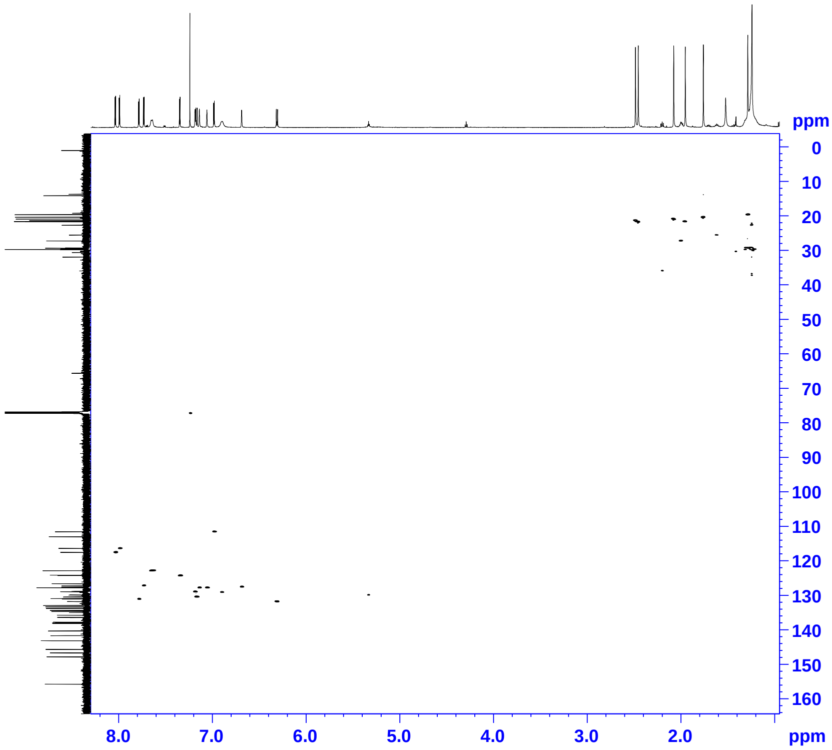

Figure S5. $^{1}H,^{13}C$ HSQC spectrum (600/150 MHz, $CDCl_3$, 300 K) of **5aa**.

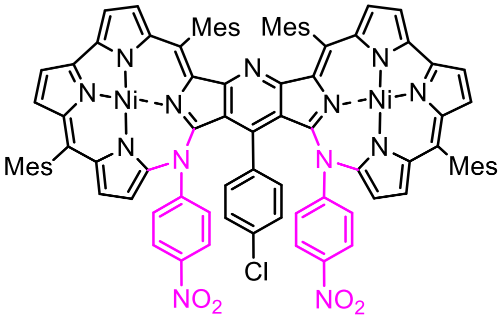

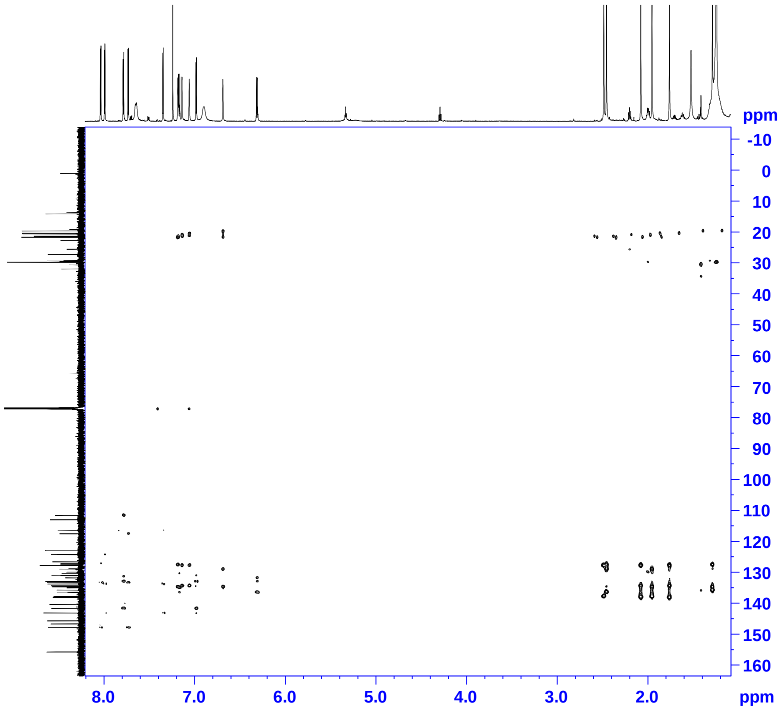

Figure S6. $^{1}H,^{13}C$ HMBC spectrum (600/150 MHz, $CDCl_3$, 300 K) of **5aa**.

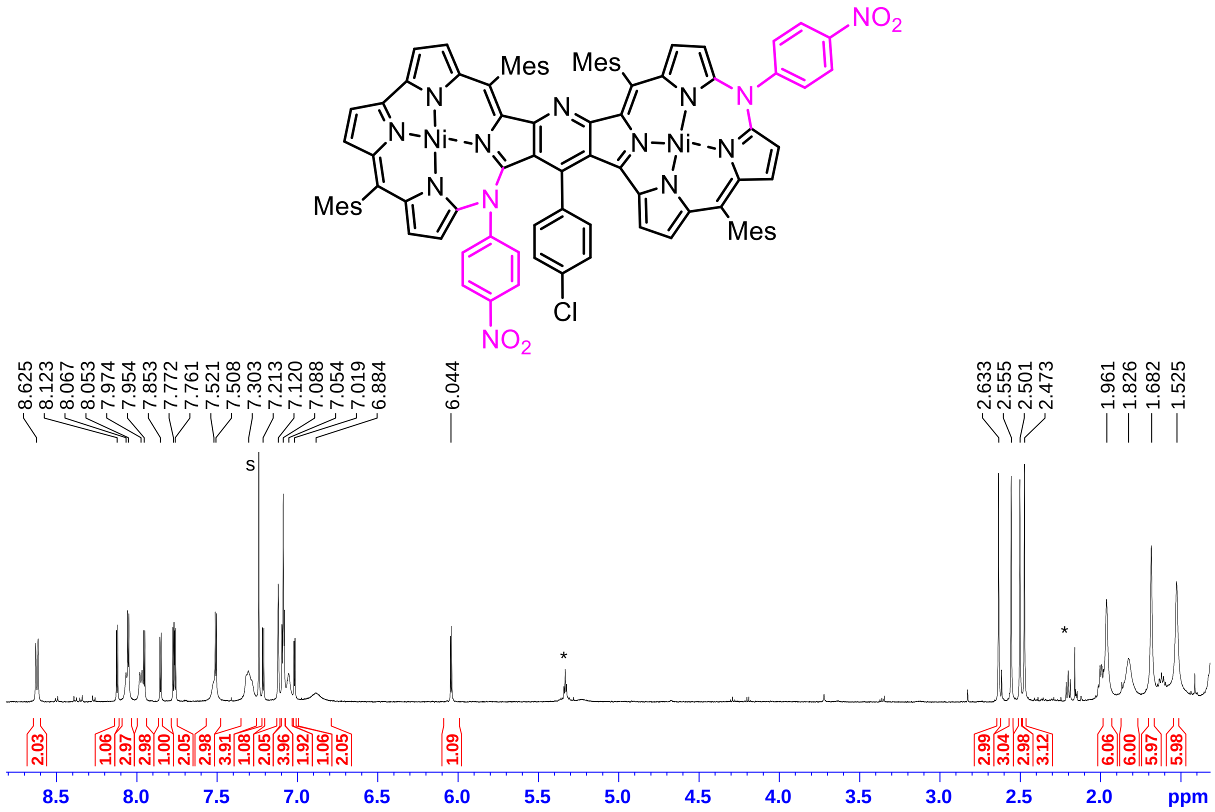

Figure S7. ^{1}H NMR spectrum (600 MHz, $CDCl_3$, 300 K) of **5ab**.

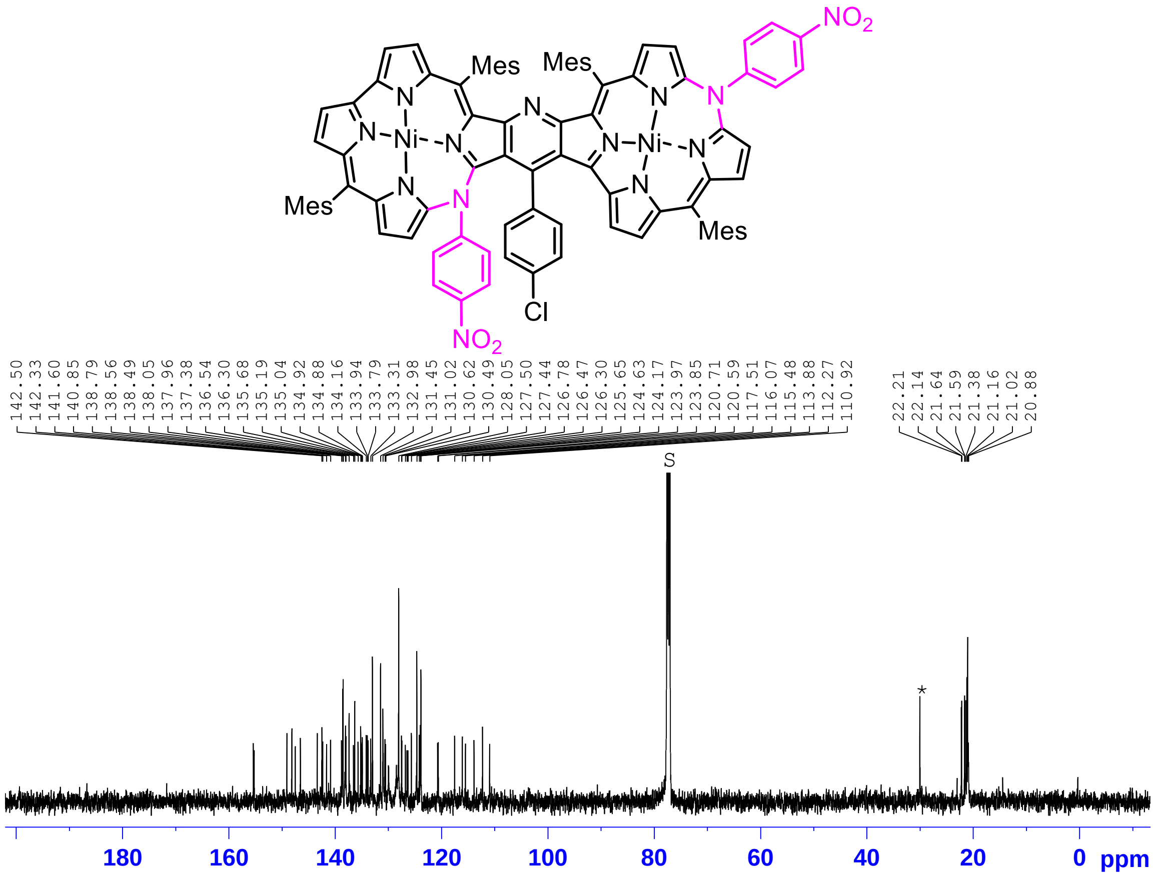

Figure S8. ^{13}C NMR spectrum (150 MHz, $CDCl_3$, 300 K) of **5ab**.

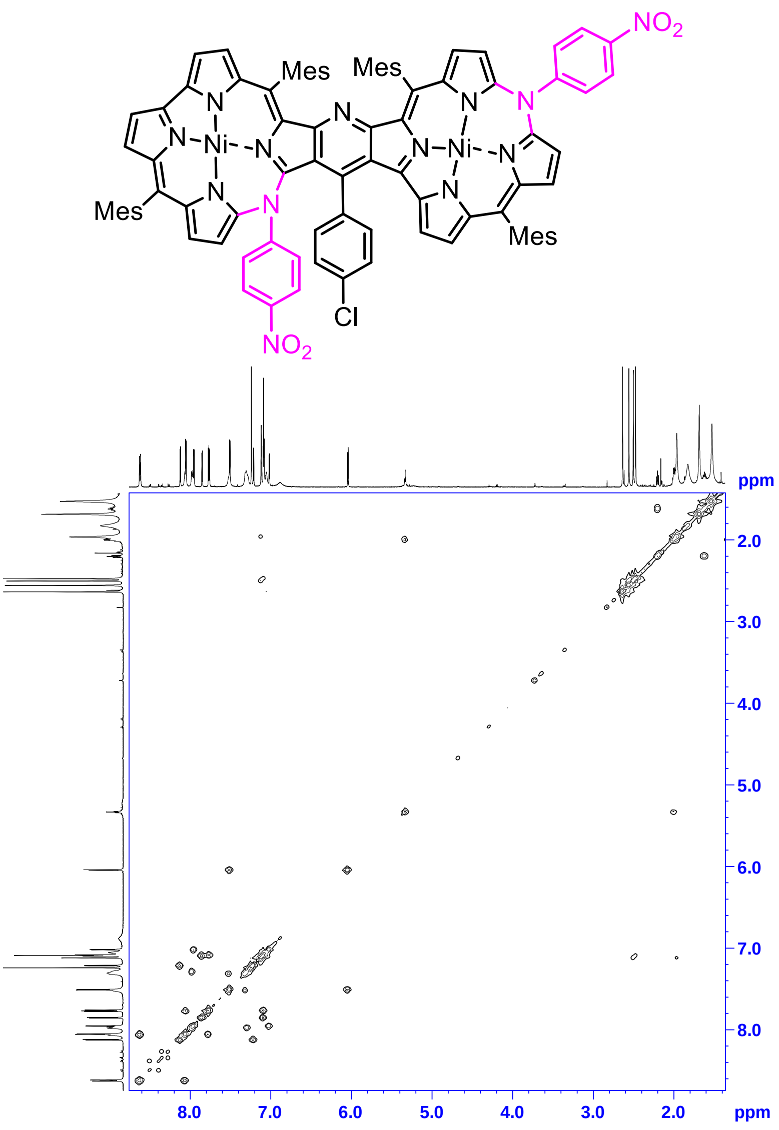

Figure S9. $^{1}H,^{1}H$ COSY spectrum (600 MHz, $CDCl_3$, 300 K) of **5ab**.

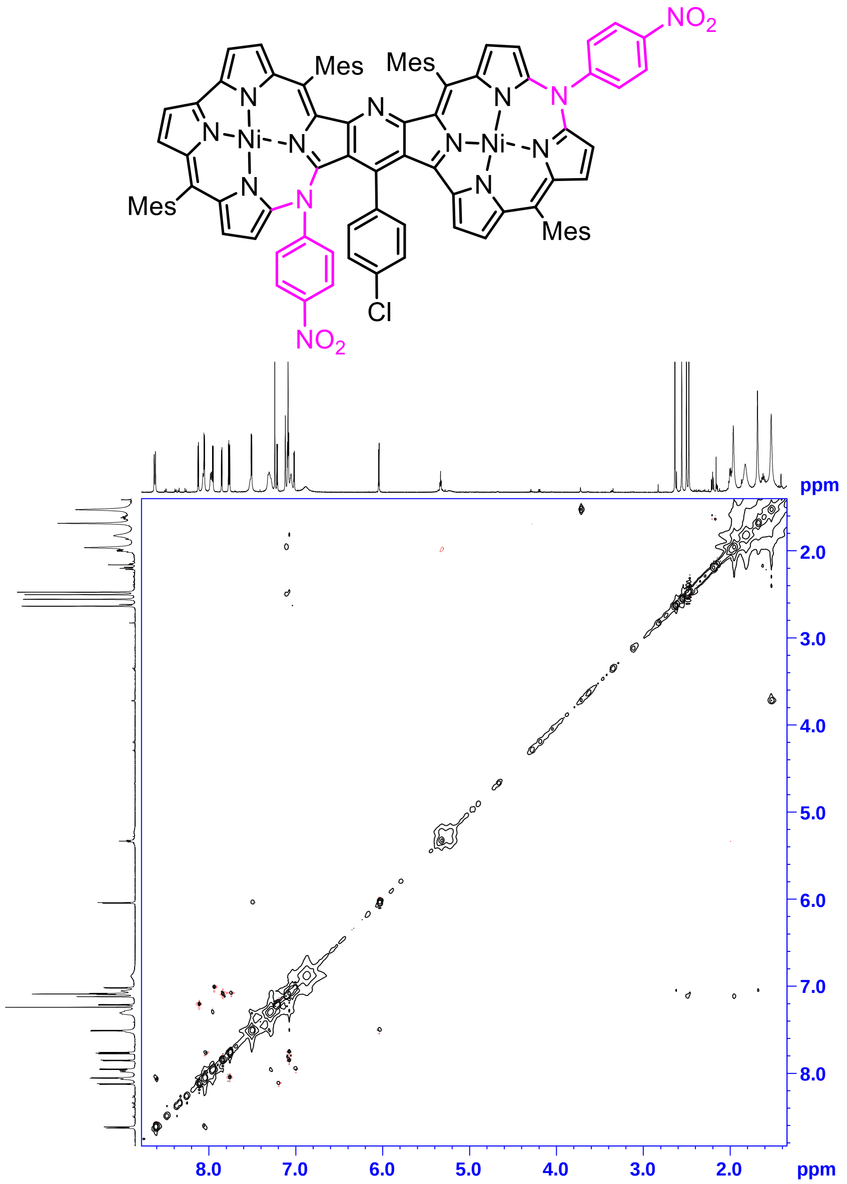

Figure S10. $^{1}H,^{1}H$ NOESY spectrum (600 MHz, $CDCl_3$, 300 K) of **5ab**.

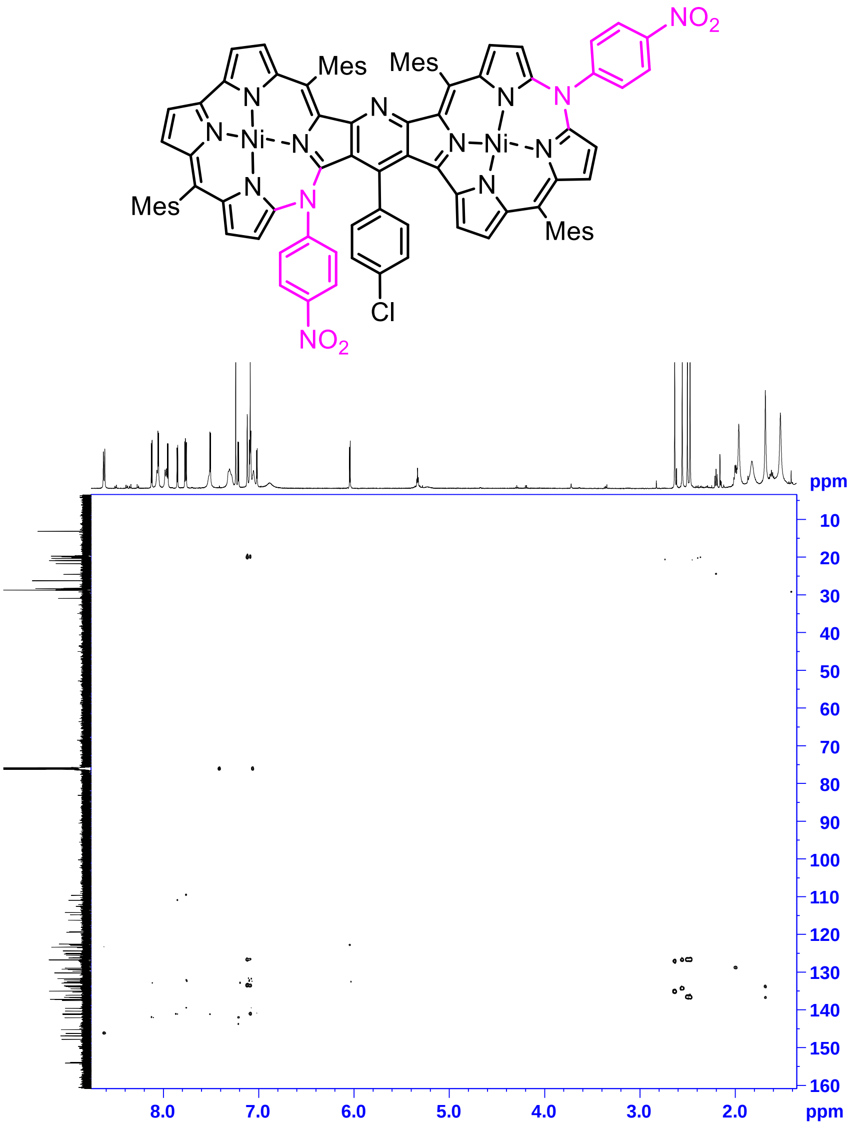

Figure S11. $^{1}H,^{13}C$ HSQC spectrum (600/150 MHz, $CDCl_3$, 300 K) of **5ab**.

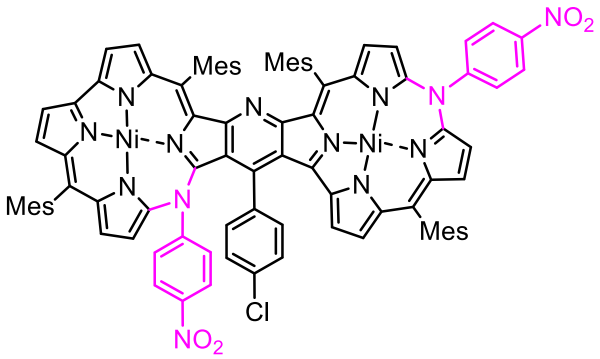

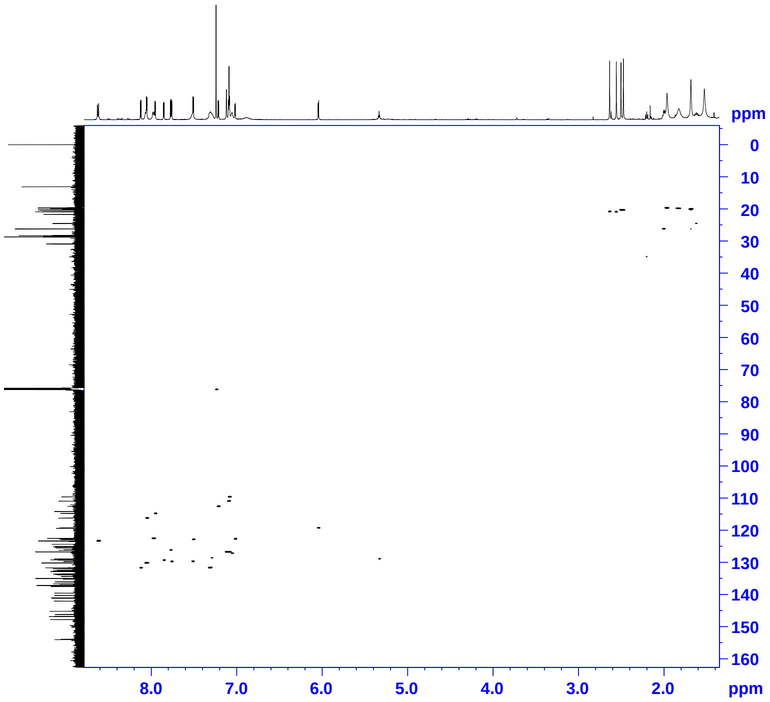

Figure S12. $^1H,^{13}C$ HMBC spectrum (600/150 MHz, $CDCl_3$, 300 K) of **5ab**.

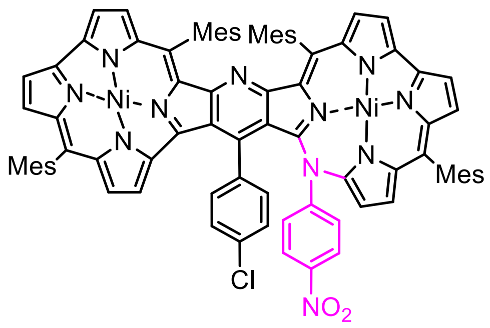

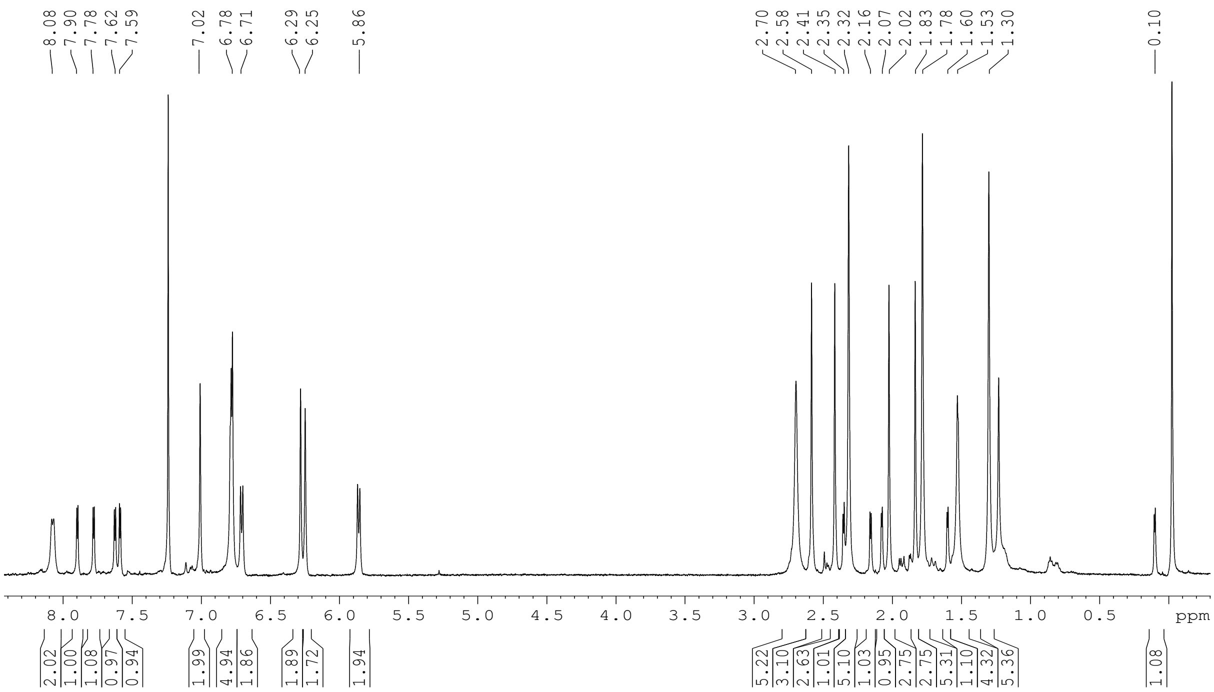

Figure S13. ^1H NMR spectrum (600 MHz, $CDCl_3$, 300 K) of **4a**.

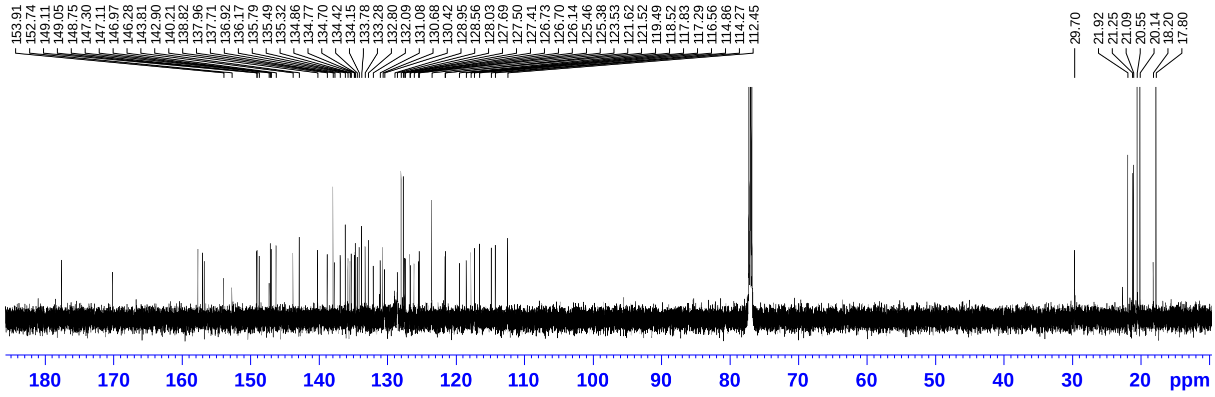

Figure S14. ^{13}C NMR spectrum (150 MHz, $CDCl_3$, 300 K) of **4a**.

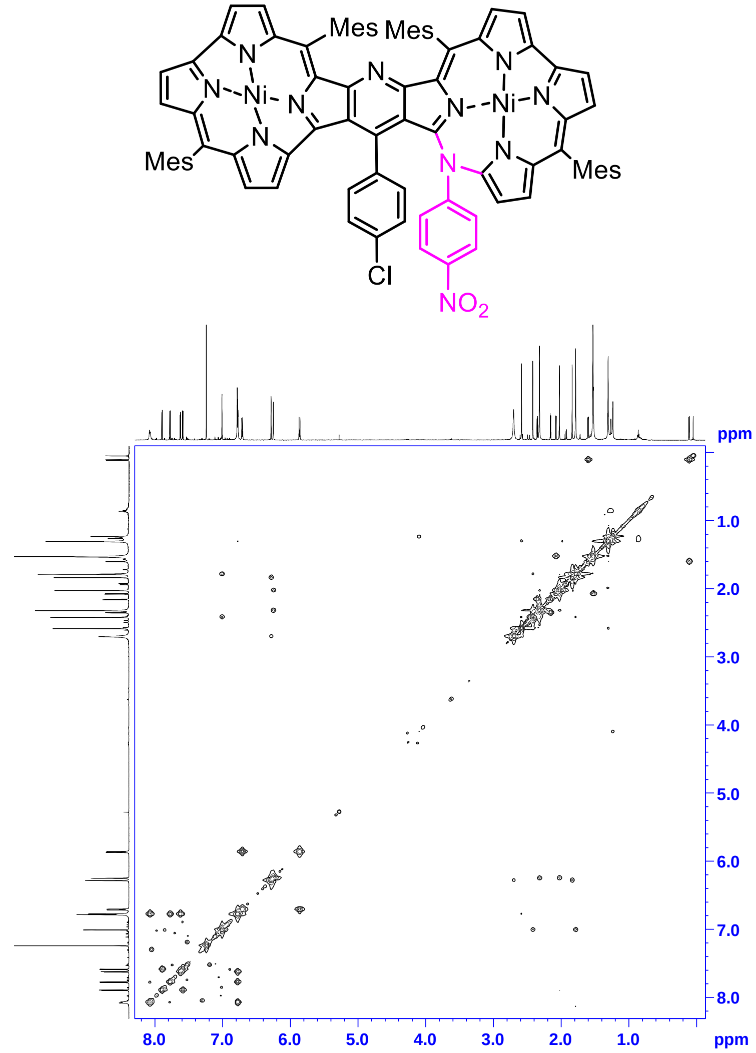

Figure S15. $^1H,^1H$ COSY spectrum (600 MHz, $CDCl_3$, 300 K) of **4a**.

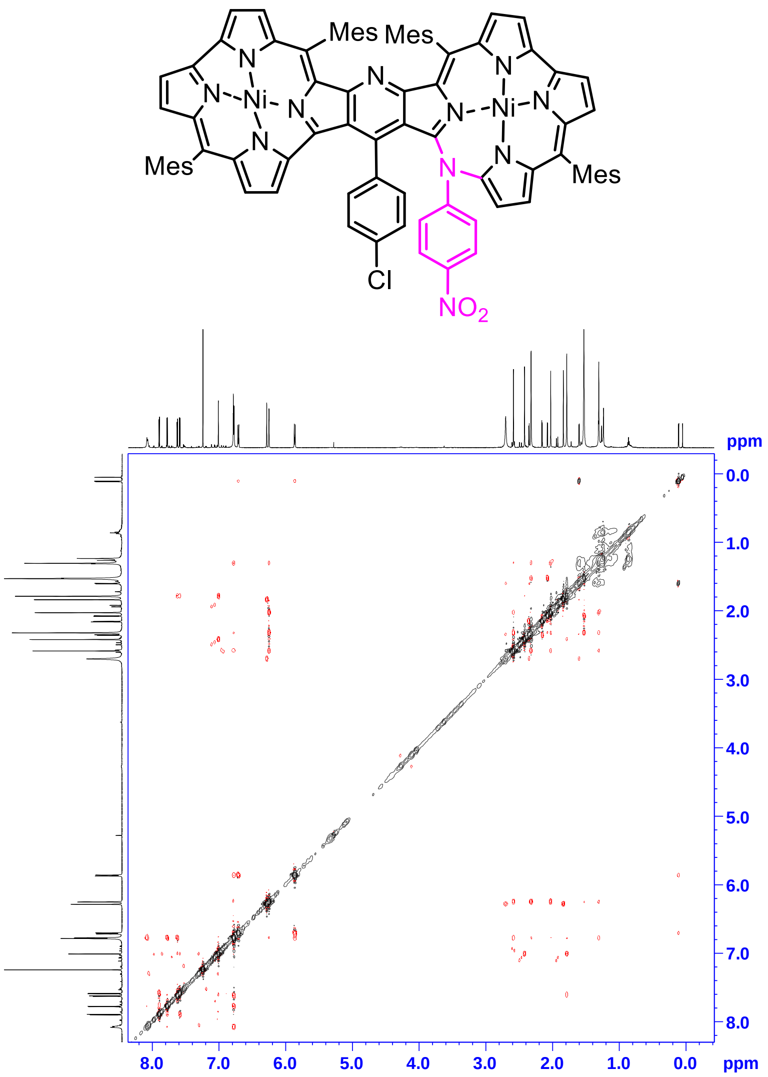

Figure S16. $^{1}H,^{1}H$ ROESY spectrum (600 MHz, $CDCl_3$, 300 K) of **4a**.

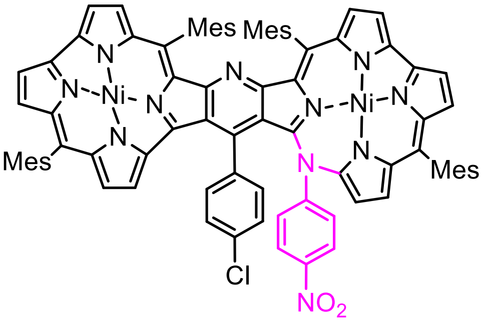

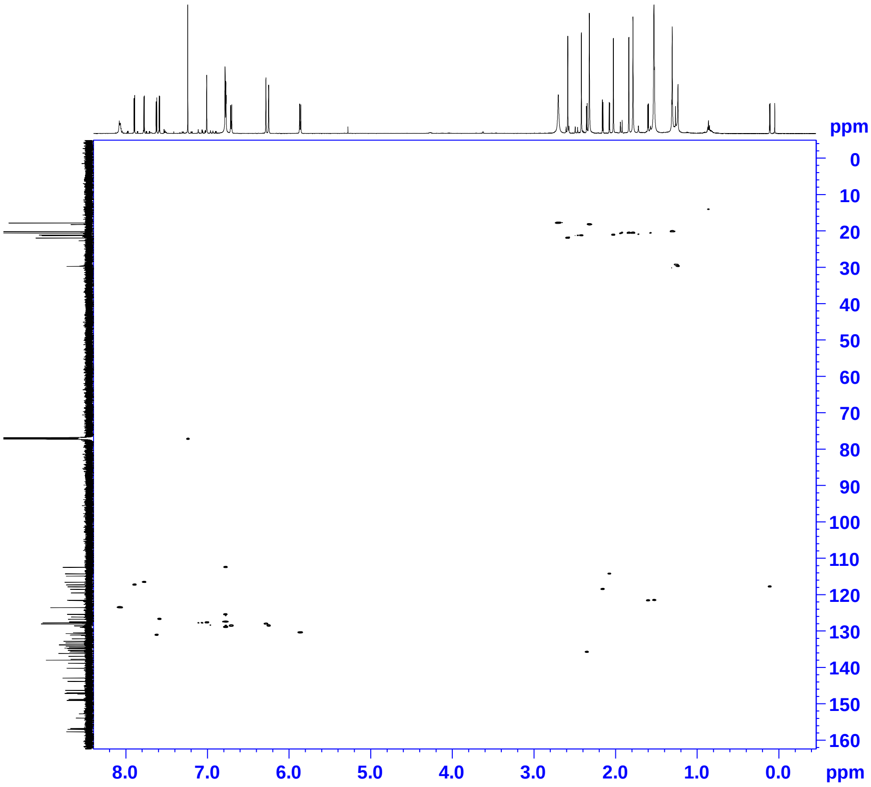

Figure S17. $^{1}H,^{13}C$ HSQC spectrum (600/150 MHz, $CDCl_3$, 300 K) of **4a**.

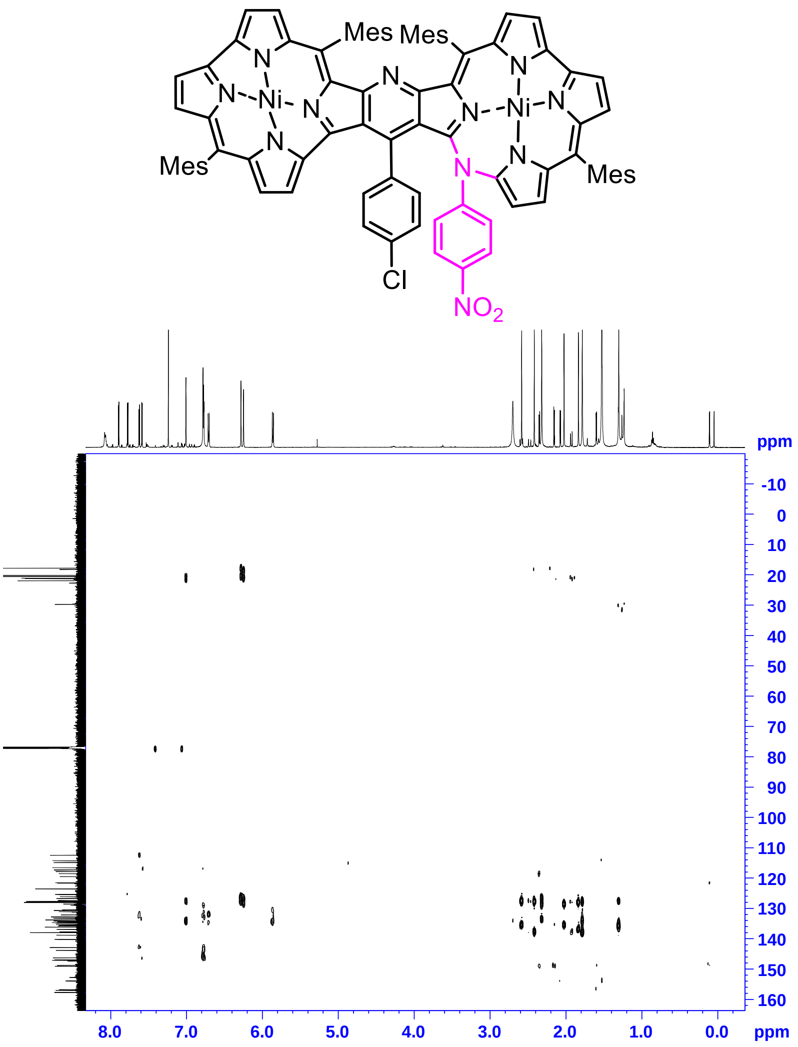

Figure S18. $^{1}H,^{13}C$ HMBC spectrum (600/150 MHz, $CDCl_3$, 300 K) of **4a**.

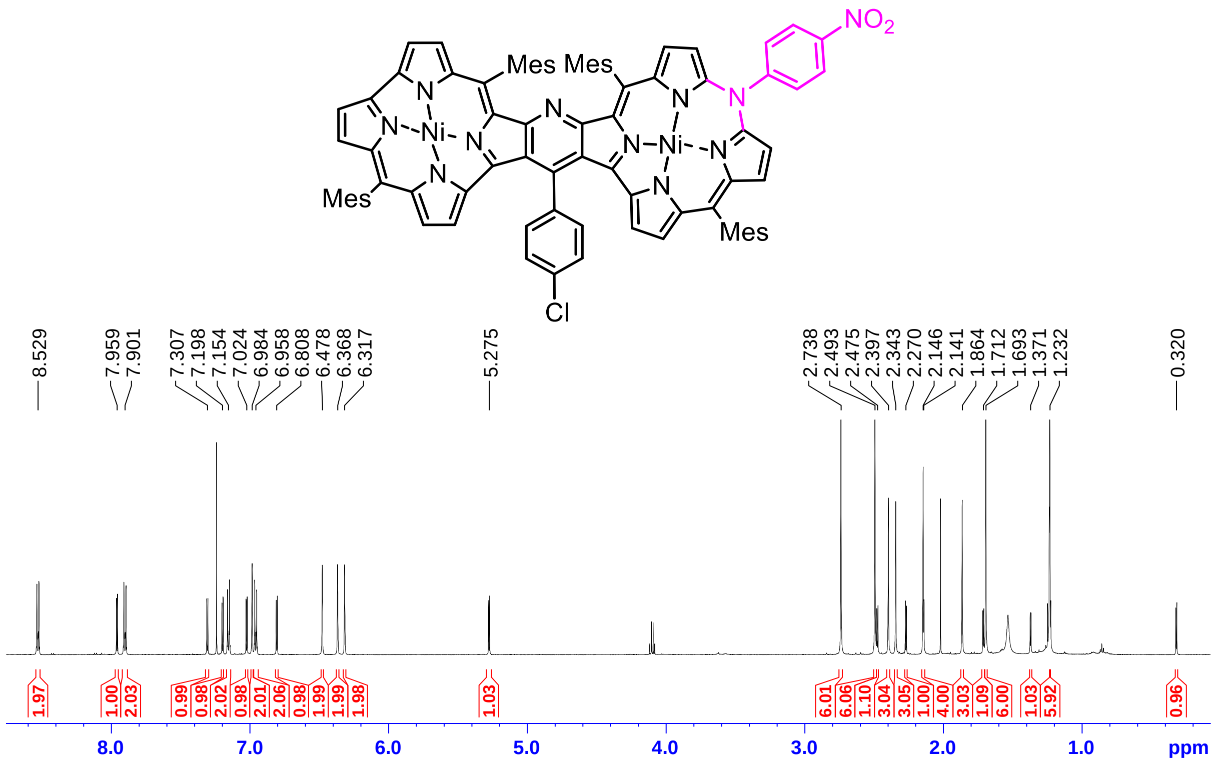

Figure S19. ^{1}H NMR spectrum (600 MHz, $CDCl_3$, 300 K) of **4b**.

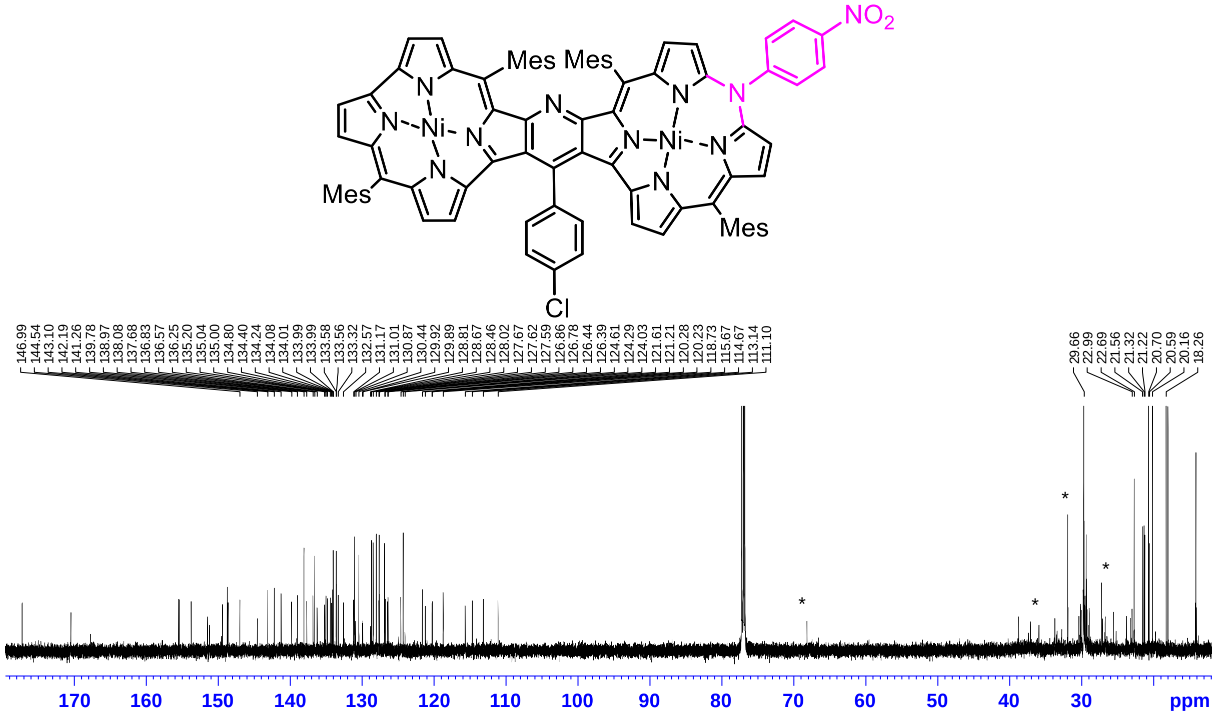

Figure S20. ^{13}C NMR spectrum (150 MHz, $CDCl_3$, 300 K) of **4b**.

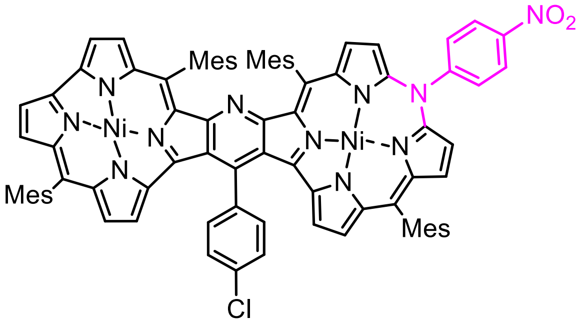

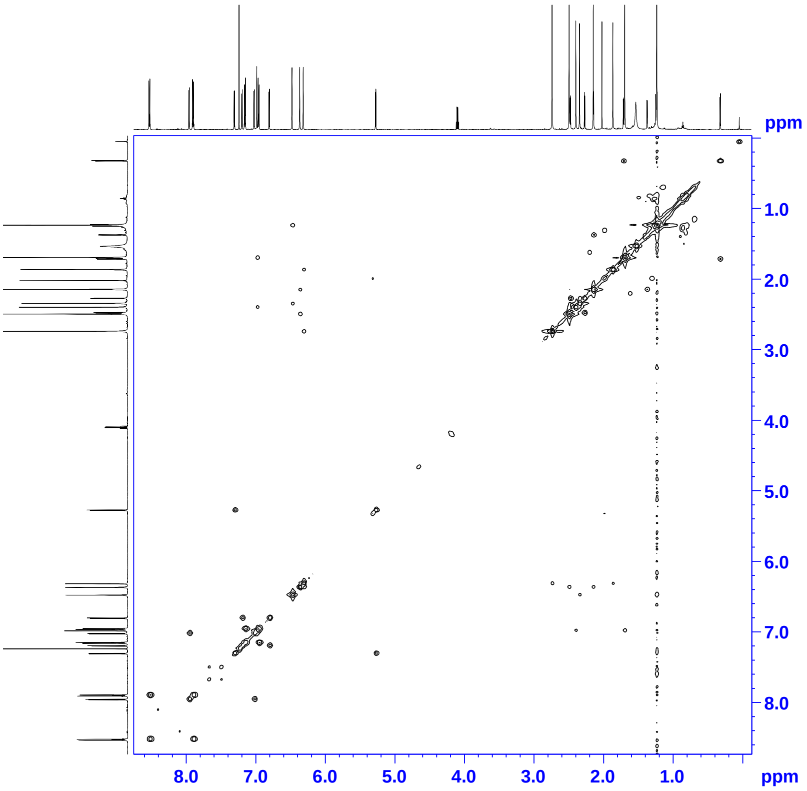

Figure S21. $^{1}H,^{1}H$ COSY spectrum (600 MHz, $CDCl_3$, 300 K) of **4b**.

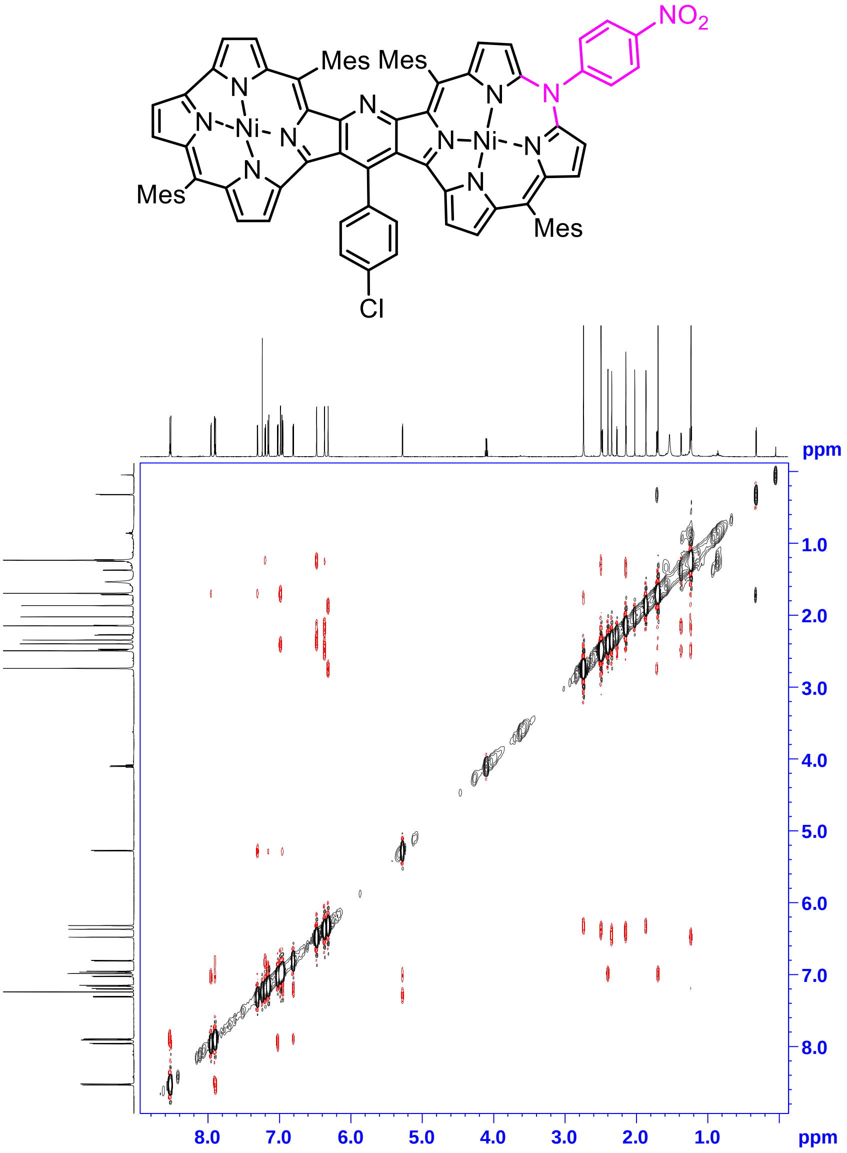

Figure S22. ^{1}H,^{1}H ROESY spectrum (600 MHz, $CDCl_3$, 300 K) of **4b**.

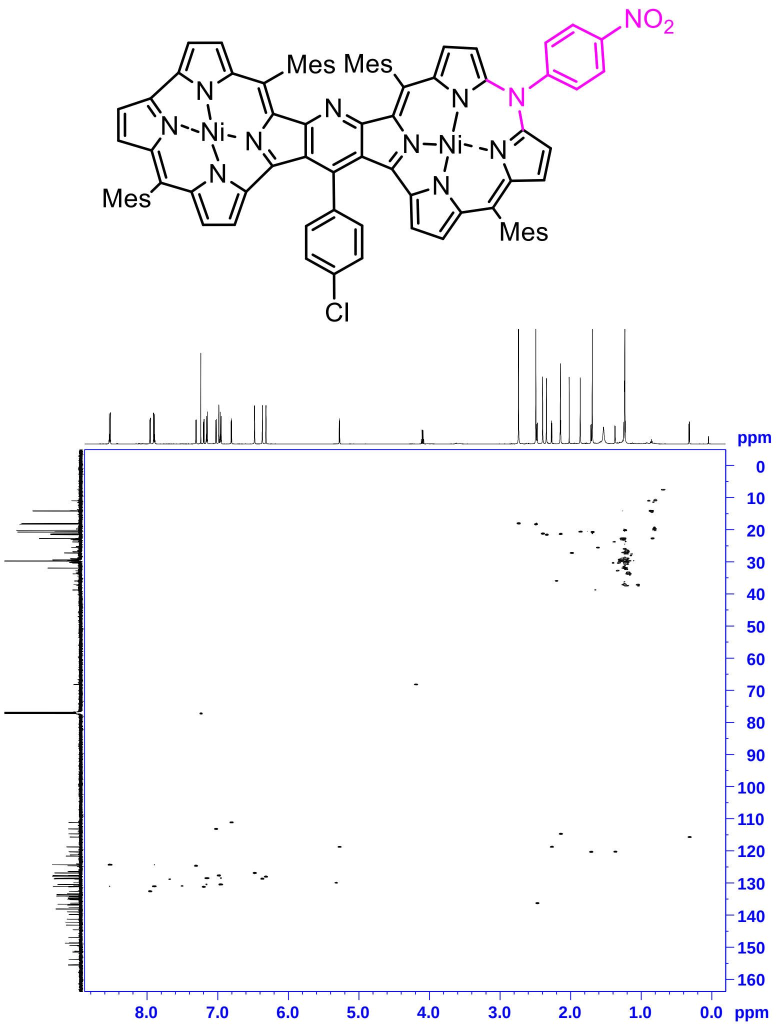

Figure S23. $^{1}H,^{13}C$ HSQC spectrum (600/150 MHz, $CDCl_3$, 300 K) of **4b**.

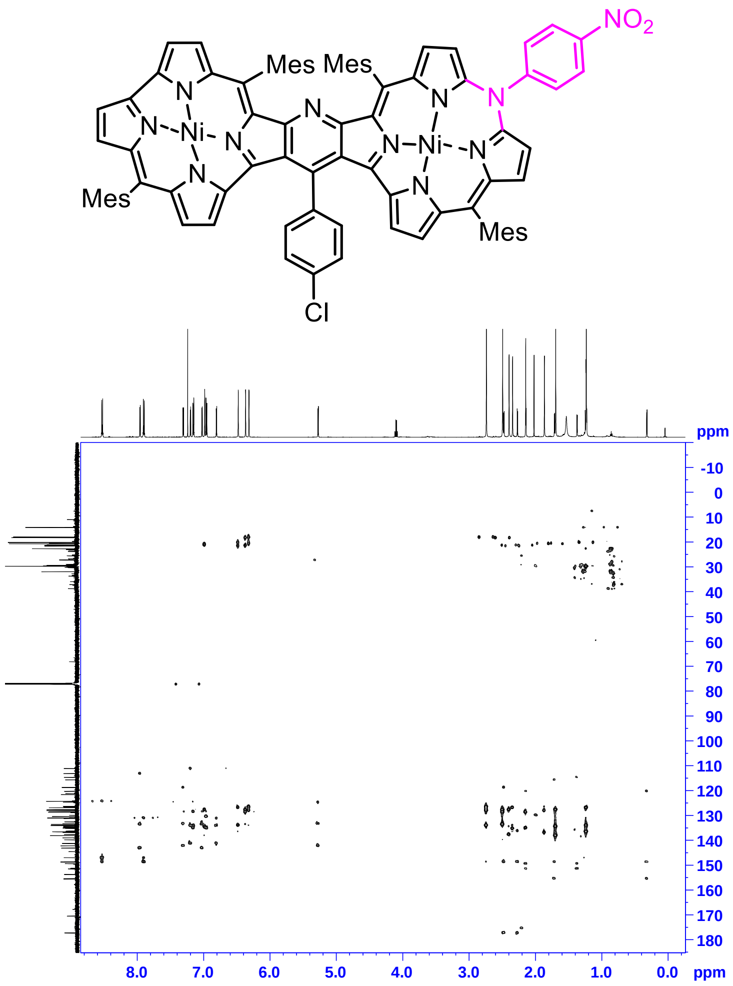

Figure S24. $^{1}H,^{13}C$ HMBC spectrum (600/150 MHz, $CDCl_3$, 300 K) of **4b**.

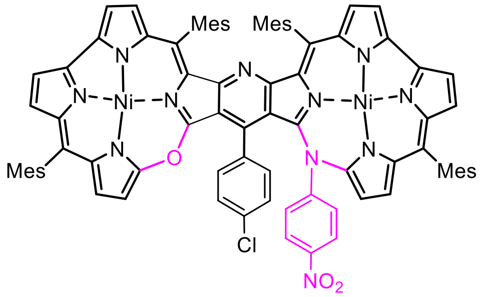

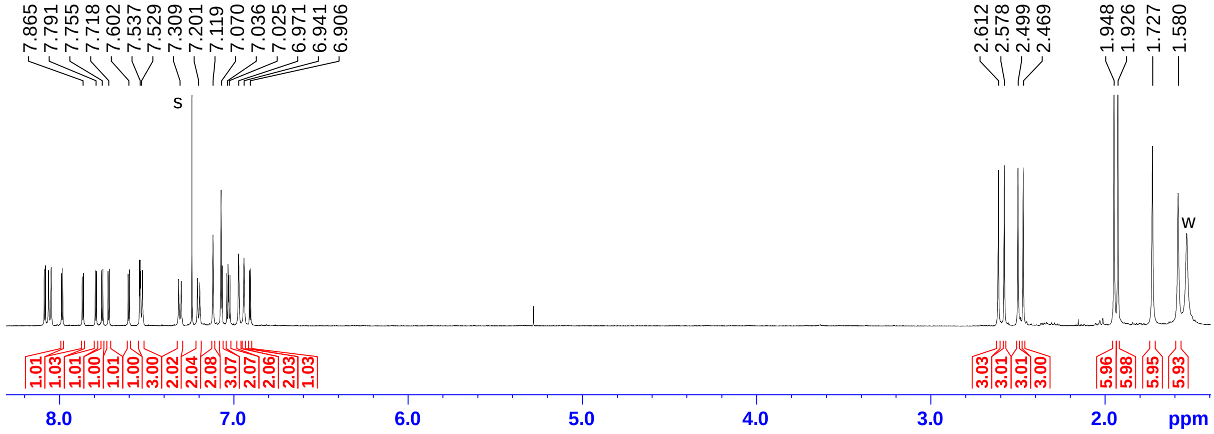

Figure S25. ^{1}H NMR spectrum (600 MHz, $CDCl_3$, 300 K) of **6a**.

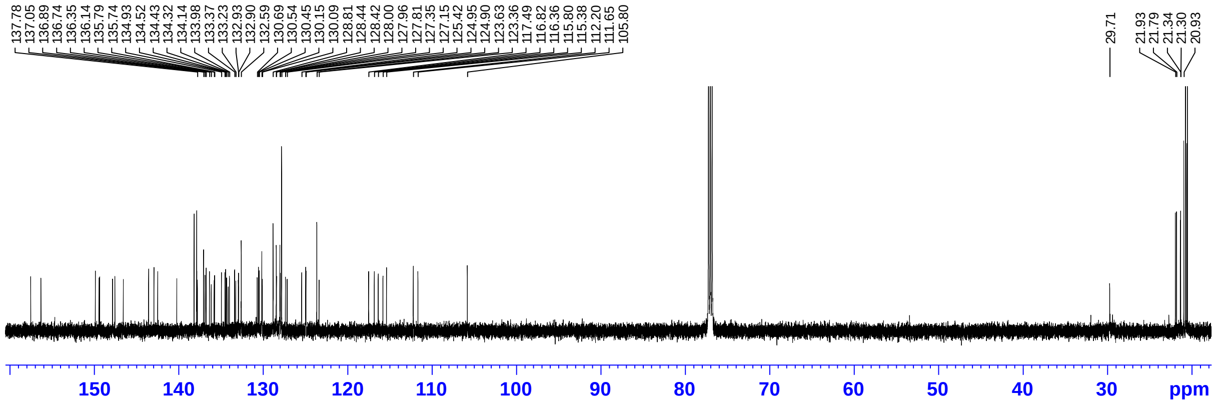

Figure S26. ^{13}C NMR spectrum (150 MHz, $CDCl_3$, 300 K) of **6a**.

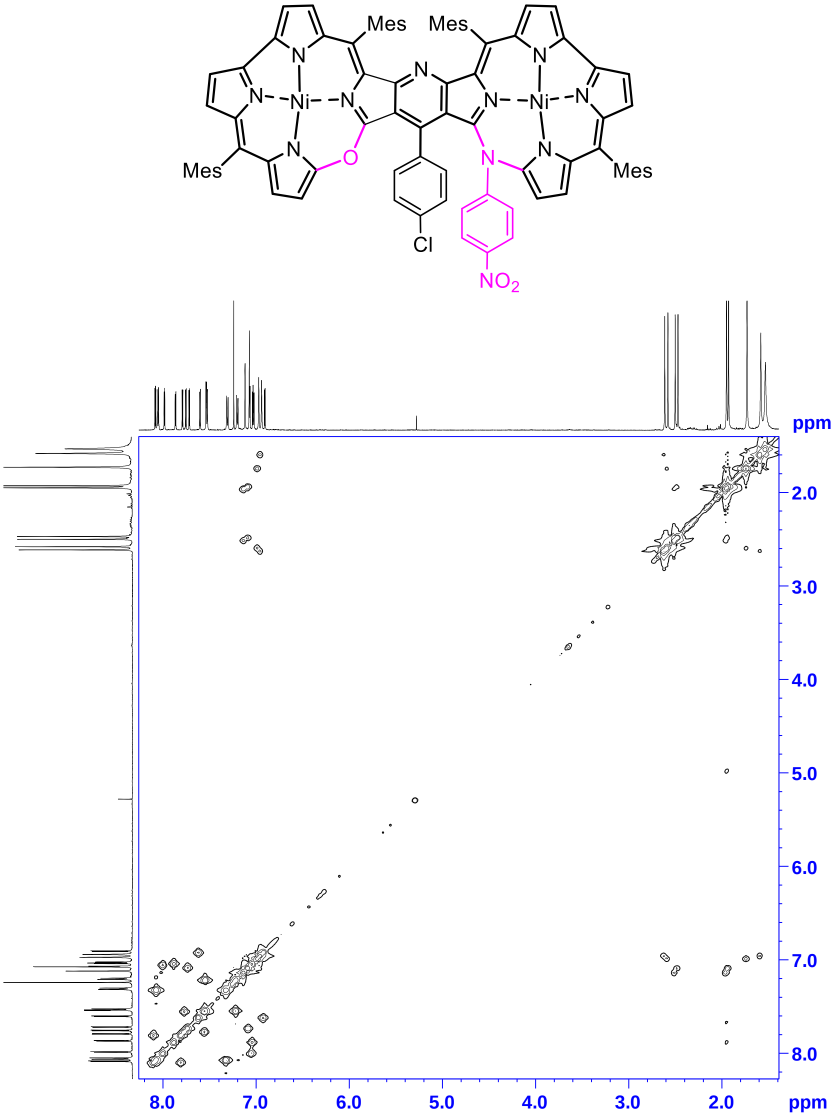

Figure S27. $^{1}H,^{1}H$ COSY spectrum (600 MHz, $CDCl_3$, 300 K) of **6a**.

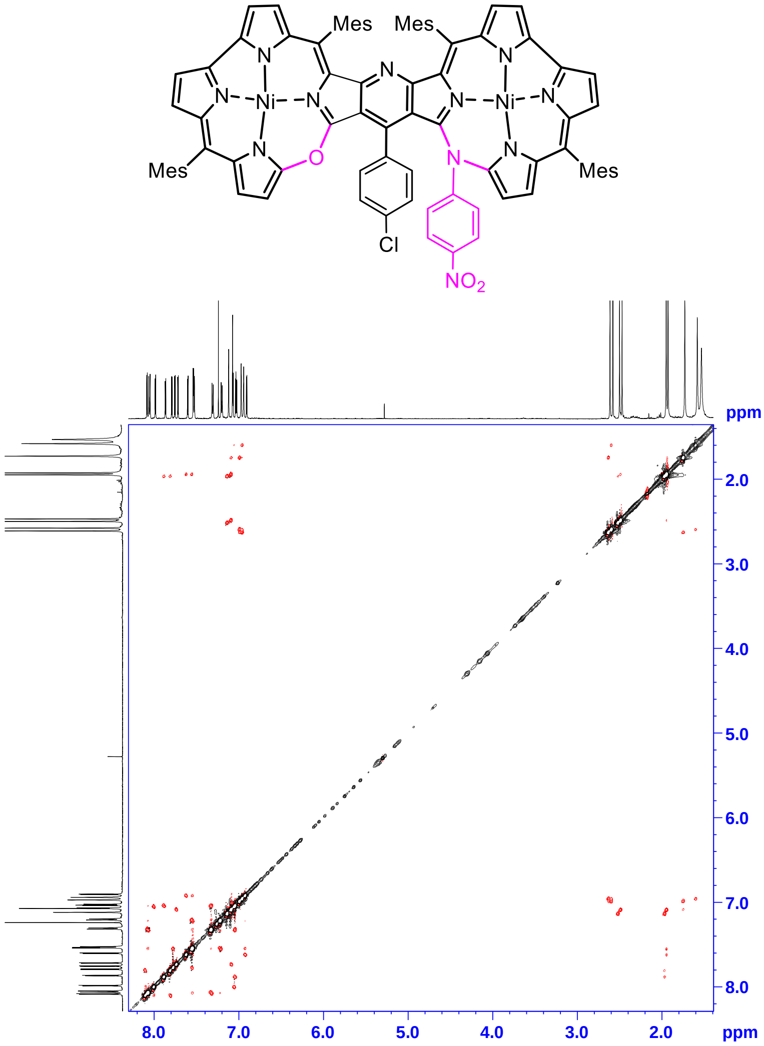

Figure S28. $^{1}H,^{1}H$ ROESY spectrum (600 MHz, $CDCl_3$, 300 K) of **6a**.

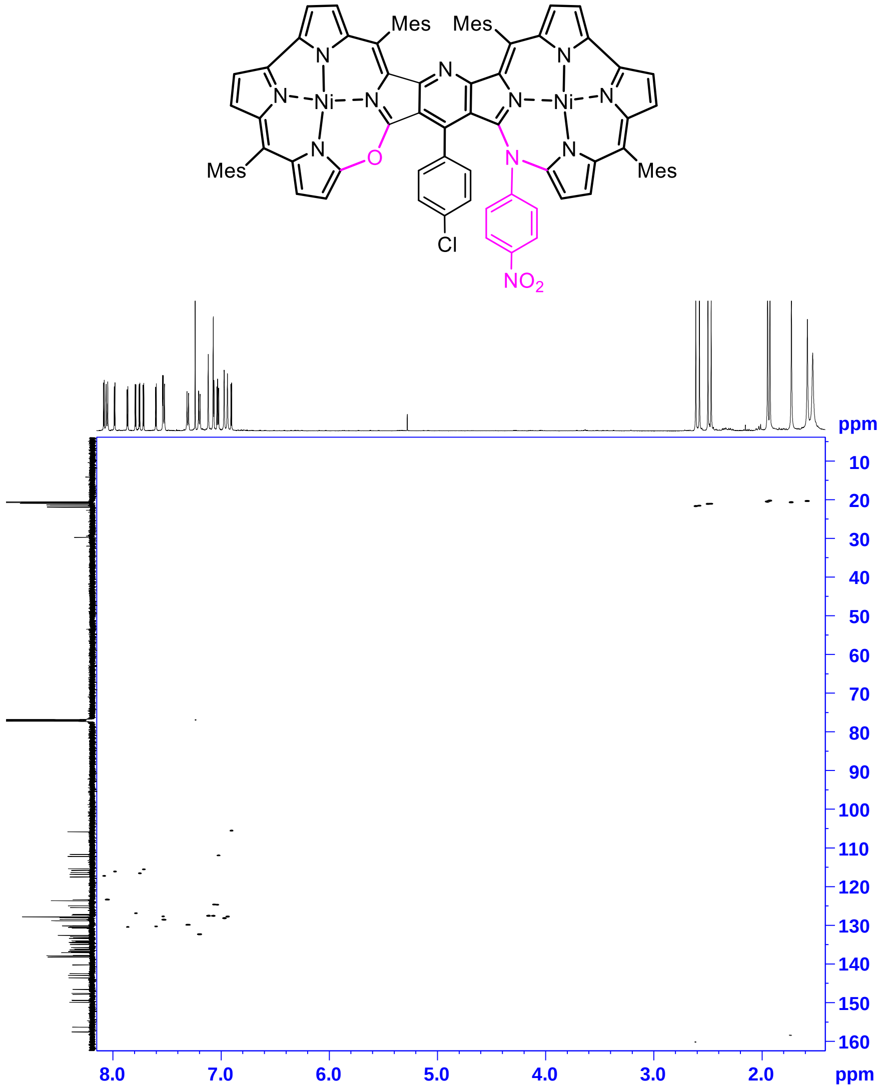

Figure S29. $^{1}H,^{13}C$ HSQC spectrum (600/150 MHz, $CDCl_3$, 300 K) of **6a**.

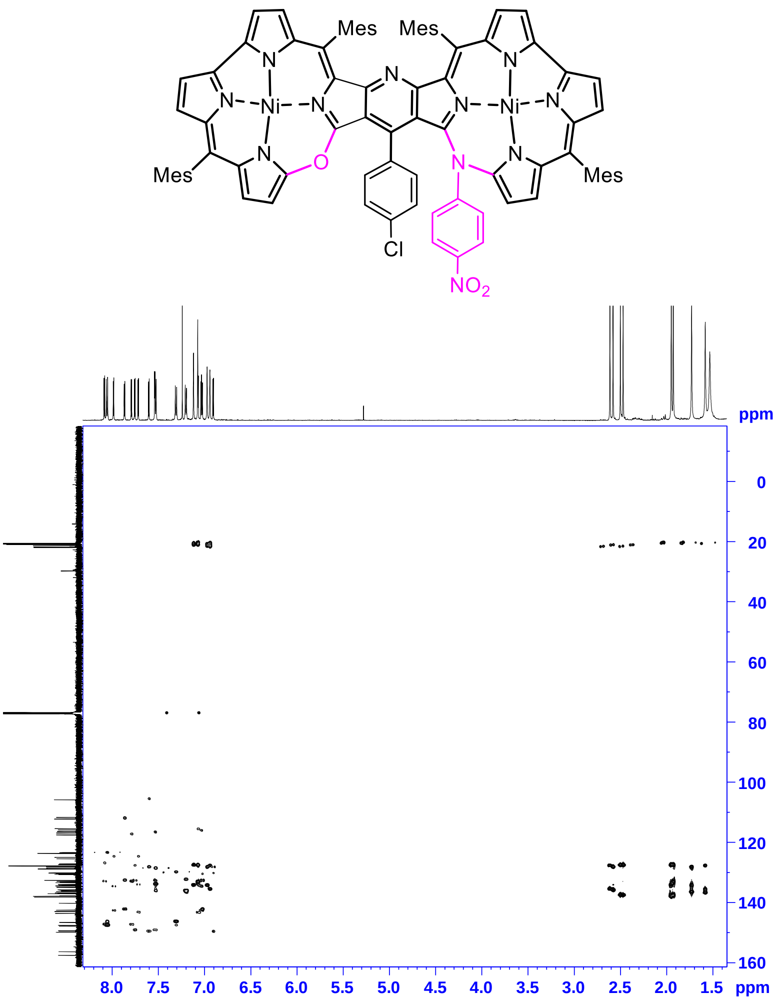

Figure S30. $^{1}H,^{13}C$ HMBC spectrum (600/150 MHz, $CDCl_3$, 300 K) of **6a**.

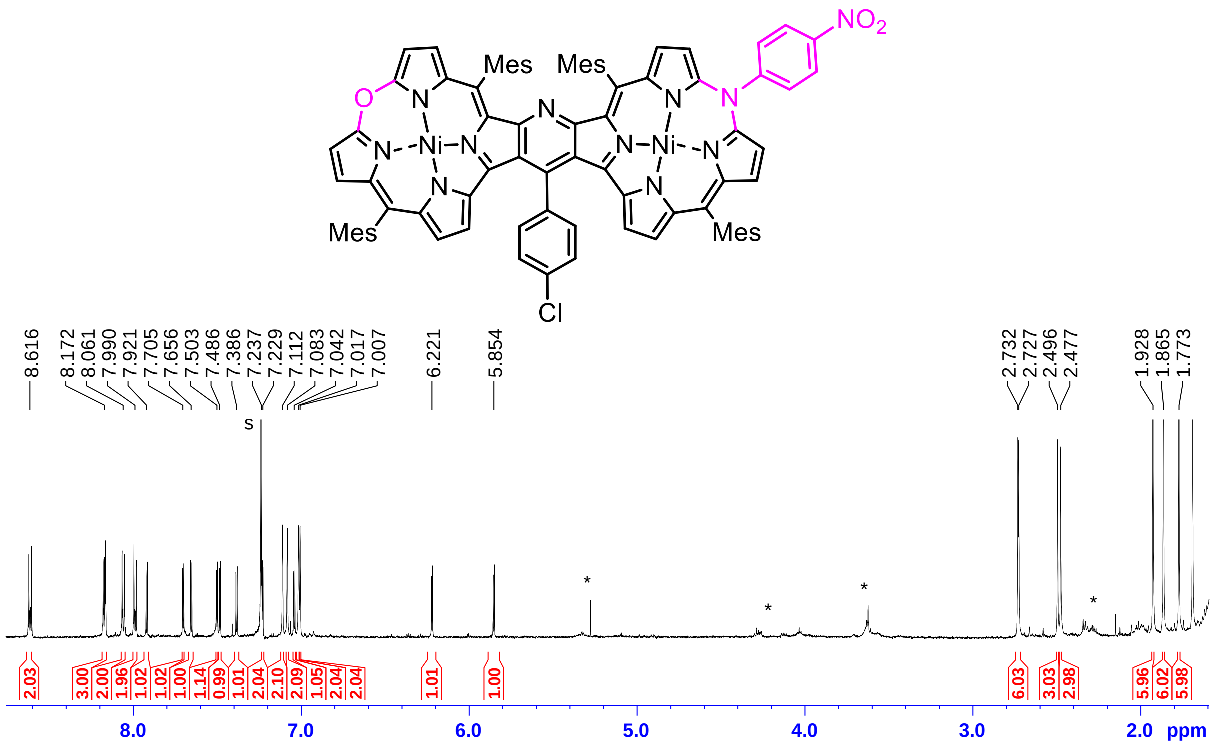

Figure S31. ^{1}H NMR spectrum (600 MHz, $CDCl_3$, 300 K) of **6b**.

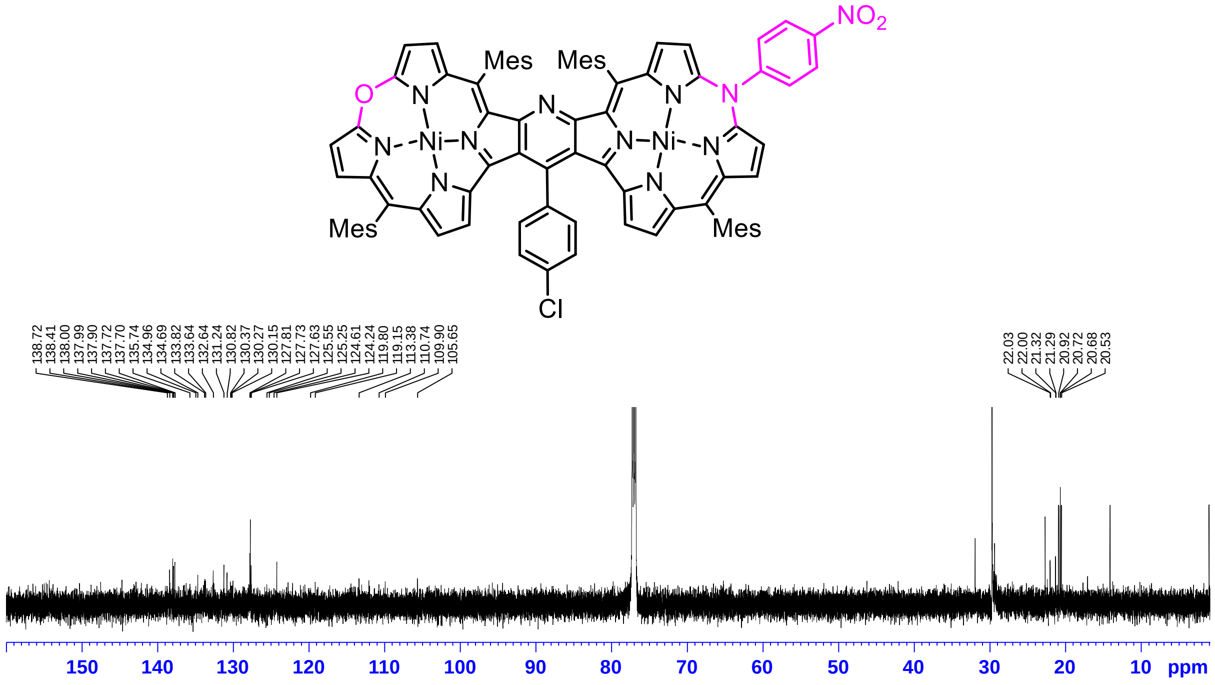

Figure S32. ^{13}C NMR spectrum (150 MHz, $CDCl_3$, 300 K) of **6b**.

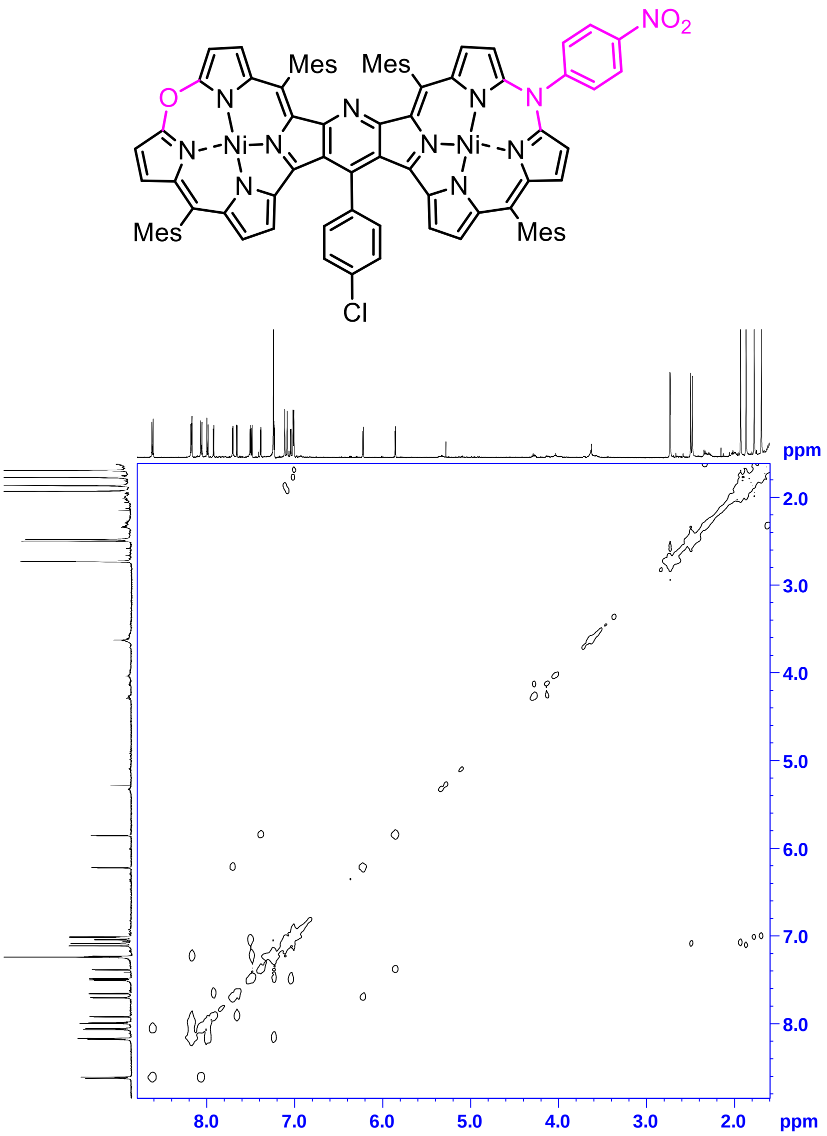

Figure S33. $^{1}H,^{1}H$ COSY spectrum (600 MHz, $CDCl_3$, 300 K) of **6b**.

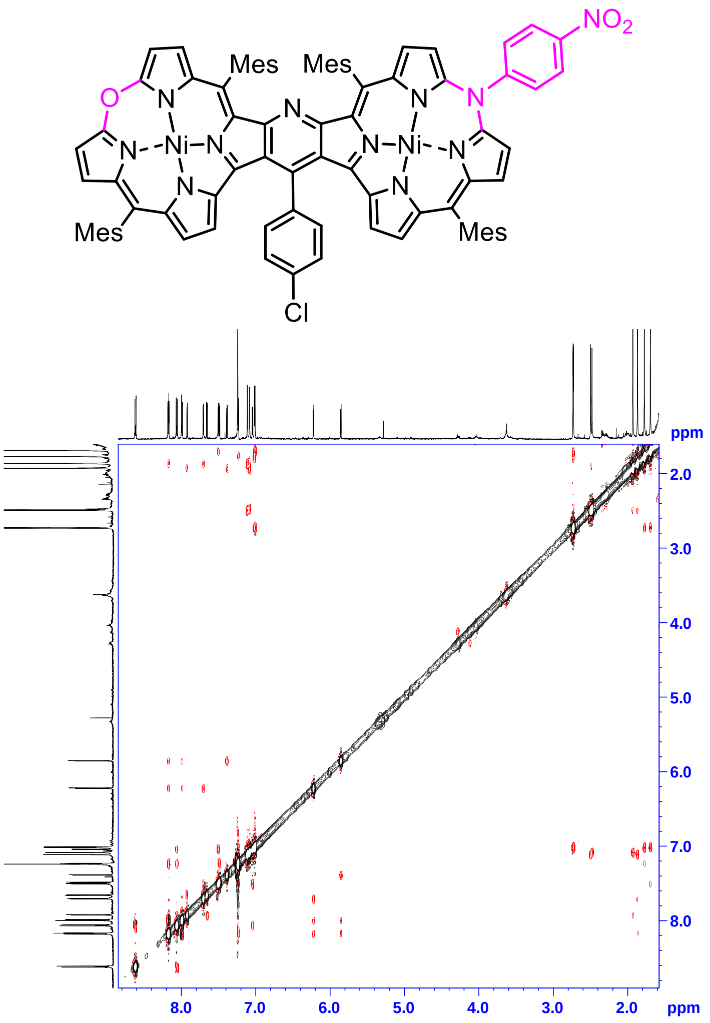

Figure S34. $^{1}H,^{1}H$ NOESY spectrum (600 MHz, $CDCl_3$, 300 K) of **6b**.

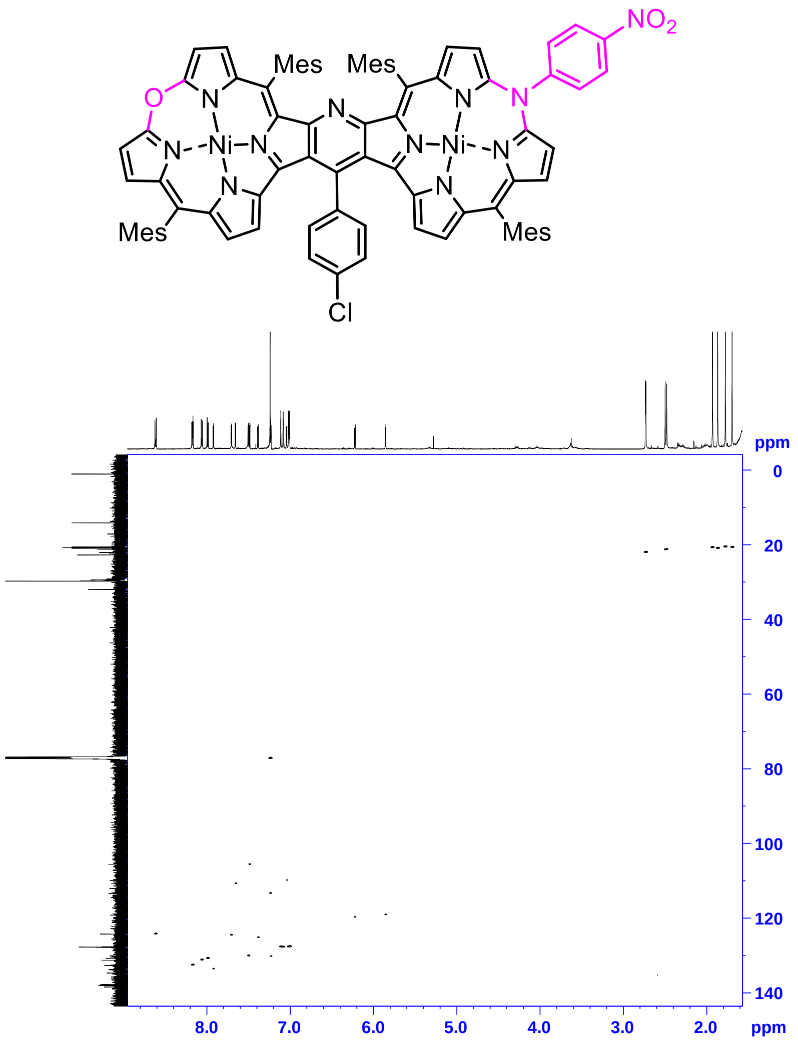

Figure S35. $^{1}H,^{13}C$ HSQC spectrum (600/150 MHz, $CDCl_3$, 300 K) of **6b**.

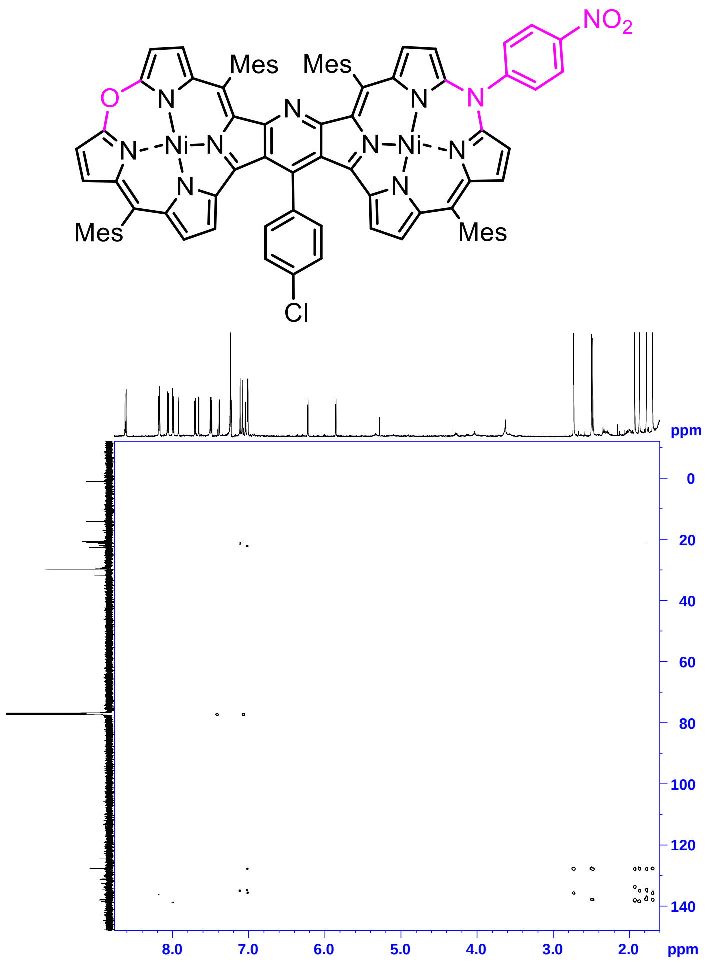

Figure S36. ^{1}H,^{13}C HMBC spectrum (600/150 MHz, $CDCl_3$, 300 K) of **6b**.

5. Mass spectra for **5aa**, **5ab**, **4a**, **4b**, **6a**, and **6b**

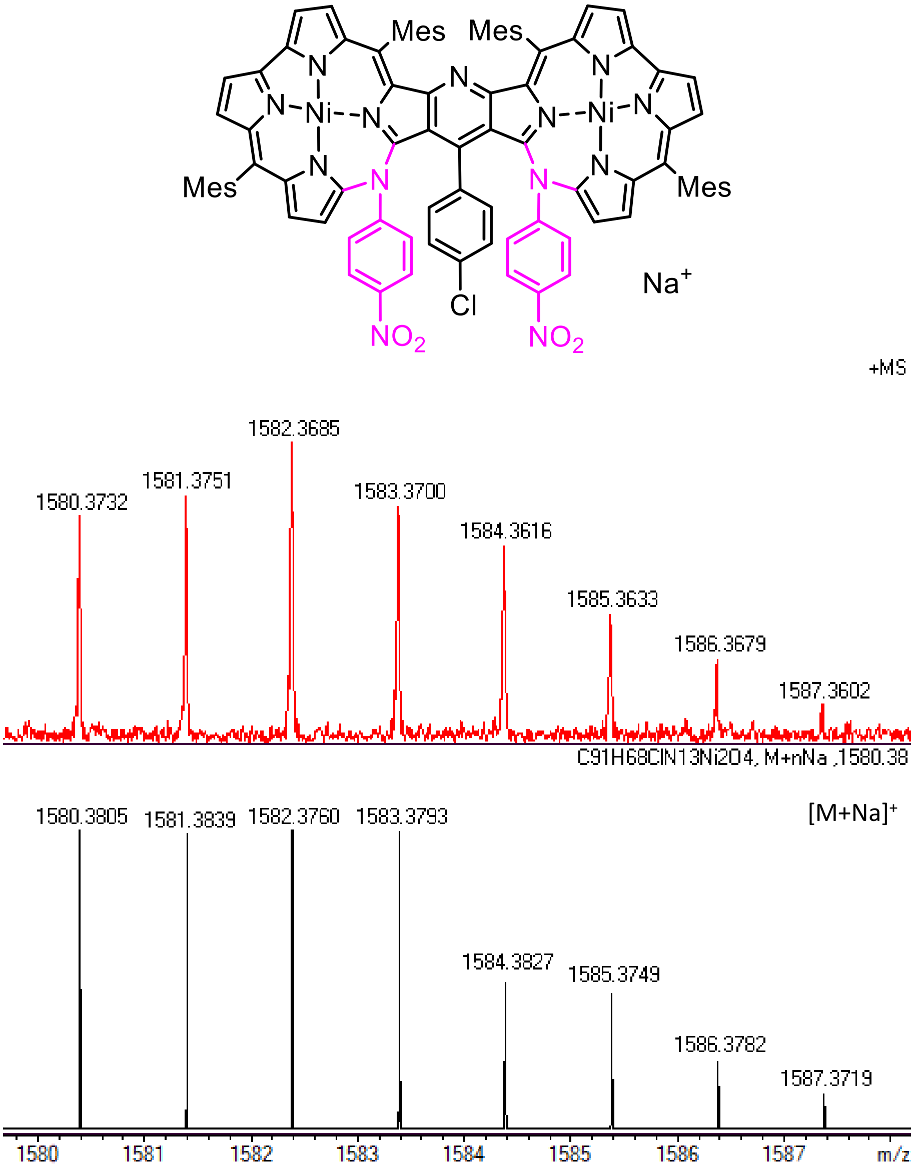

Figure S37. ESI(+) HRMS spectrum of **5aa** (experimental: red, upper trace; simulated: black, bottom trace).

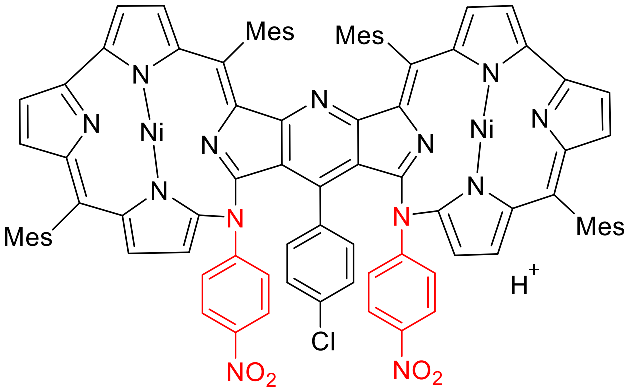

Chemical Formula: $C_{91}H_{69}ClN_{13}Ni_2O_4$
Exact Mass: 1558.3985

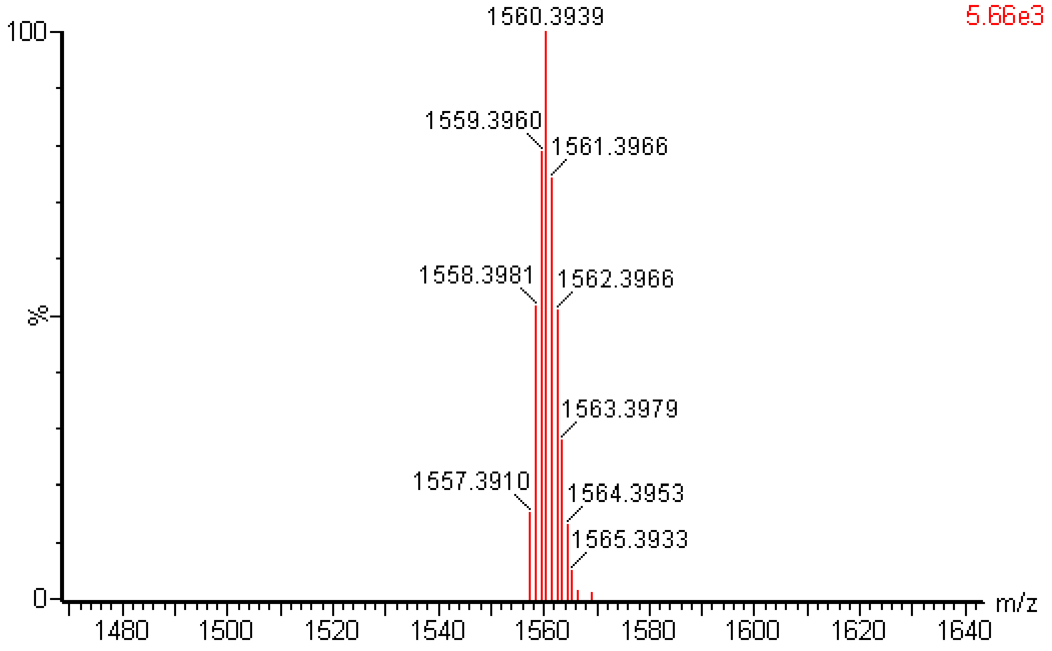

Figure S38. ESI(+) HRMS spectrum of **5aa**

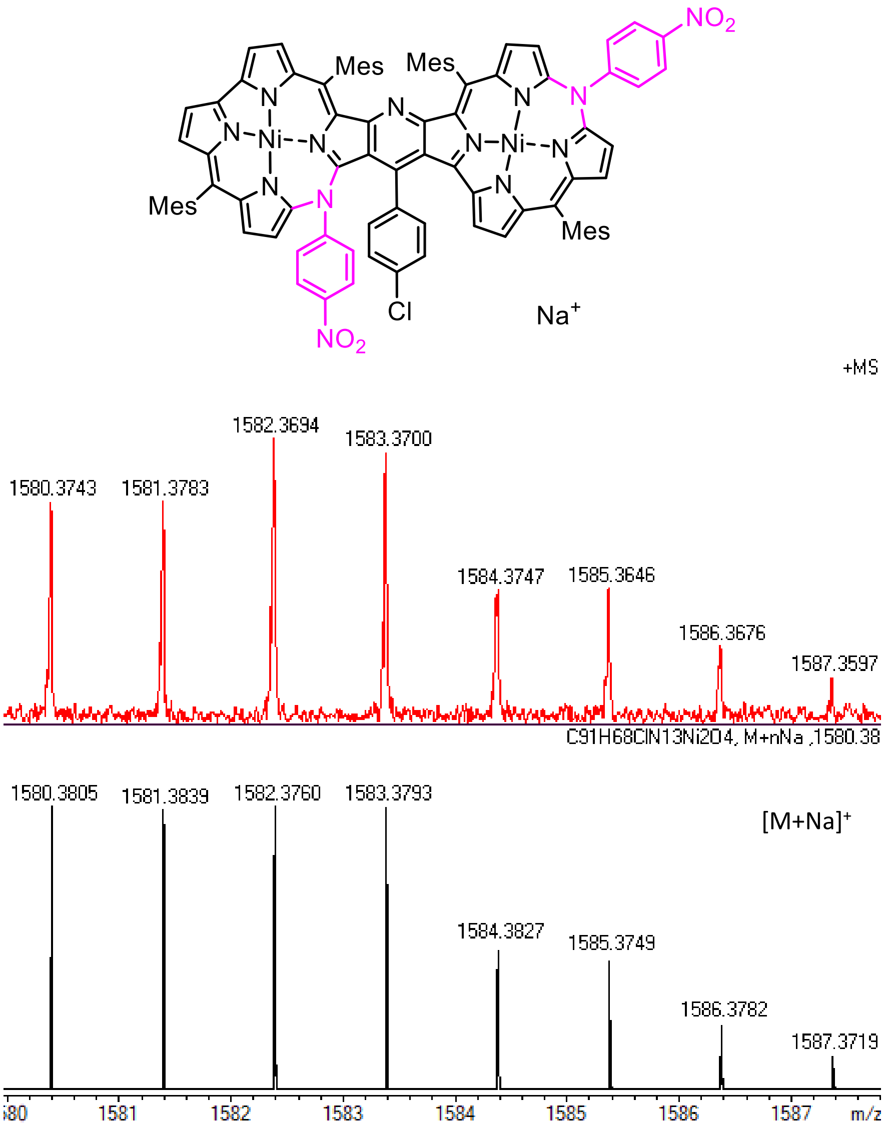

Figure S39. ESI(+) HRMS spectrum of **5ab** (experimental: red, upper trace; simulated: black, bottom trace).

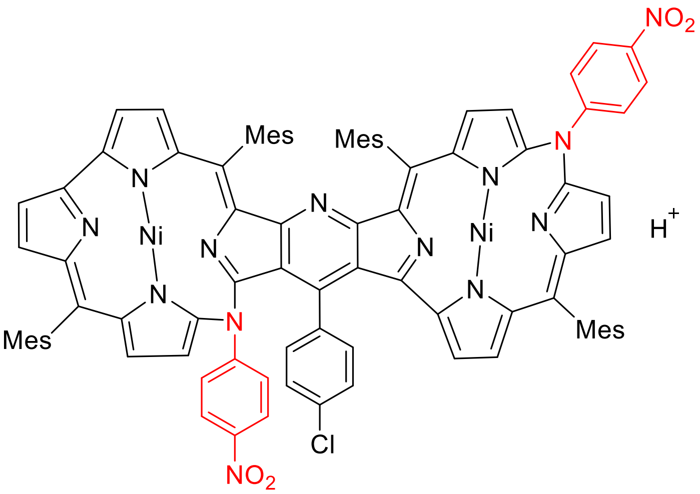

Chemical Formula: $C_{91}H_{69}ClN_{13}Ni_2O_4$
Exact Mass: 1558.3985

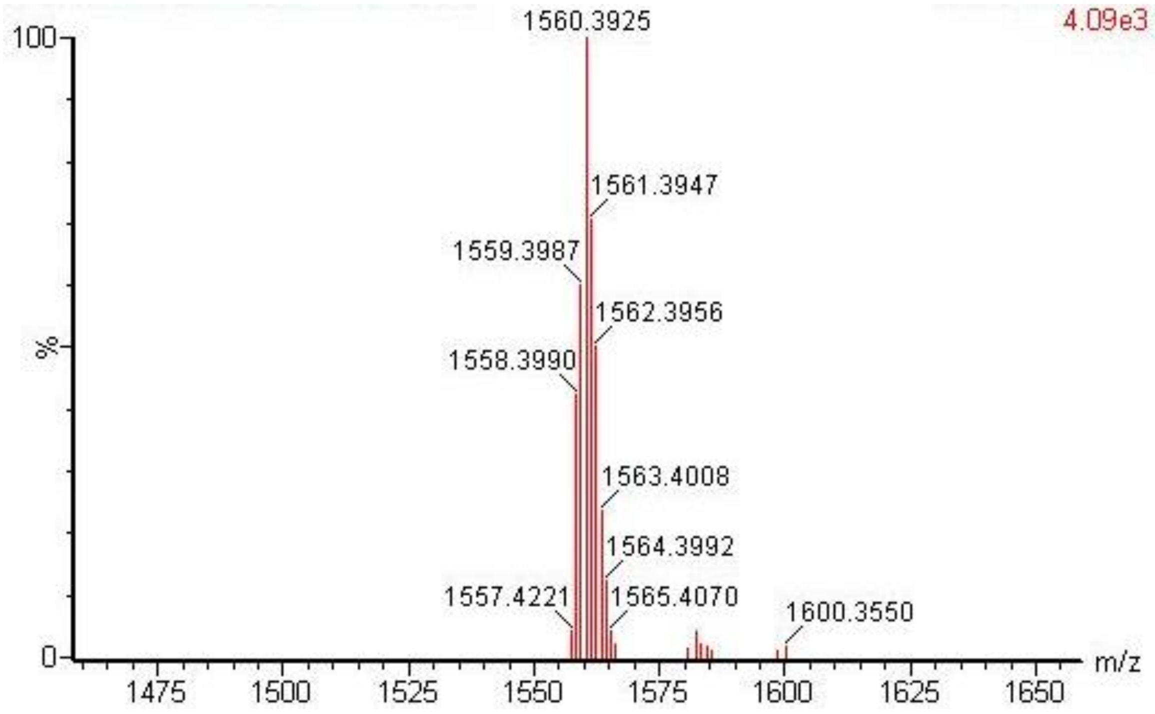

Figure S40. ESI(+) HRMS spectrum of **5ab**.

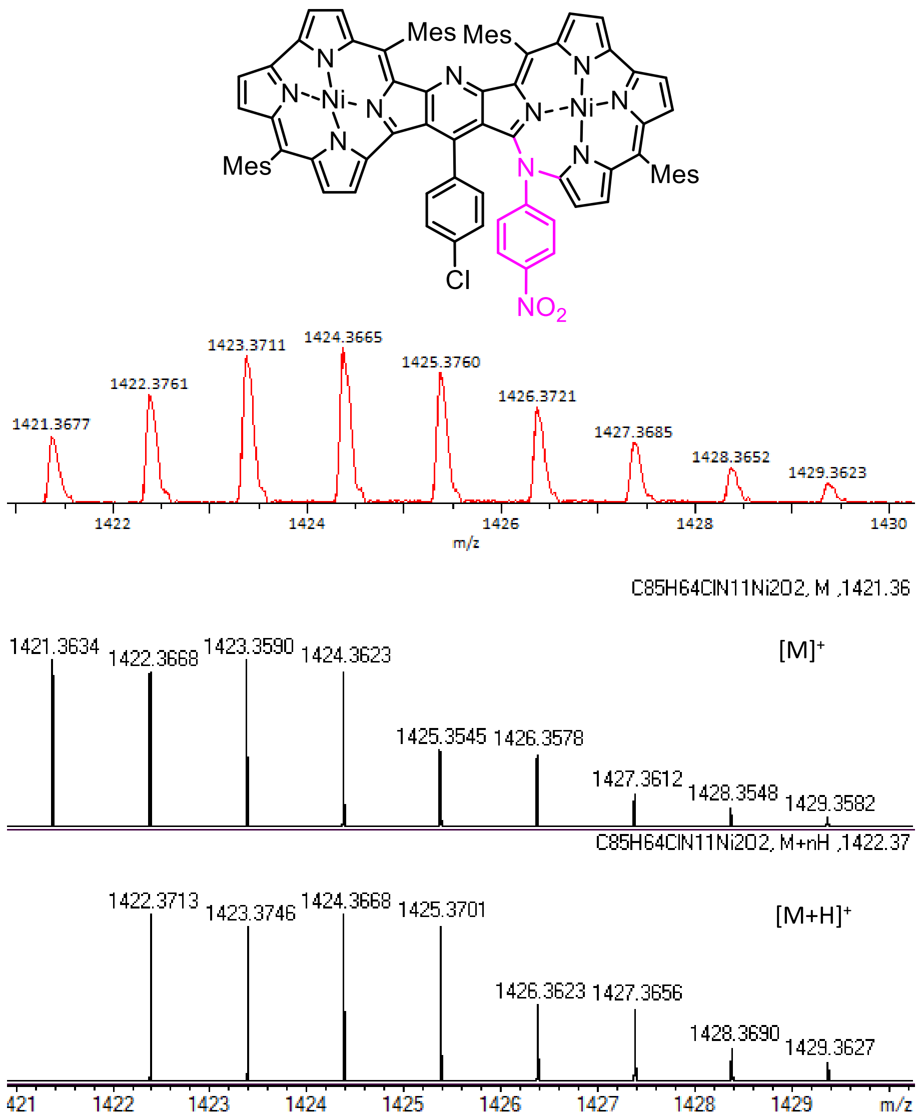

Figure S41. ESI(+) HRMS spectrum of **4a** (experimental: red, upper trace; simulated: black, bottom traces).

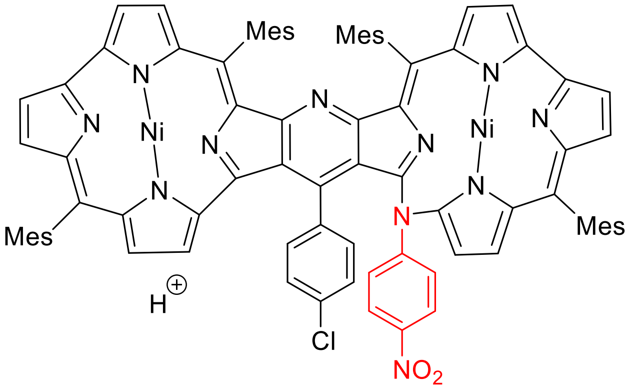

Chemical Formula: $C_{85}H_{65}ClN_{11}Ni_2O_2^+$
Exact Mass: 1422.3713

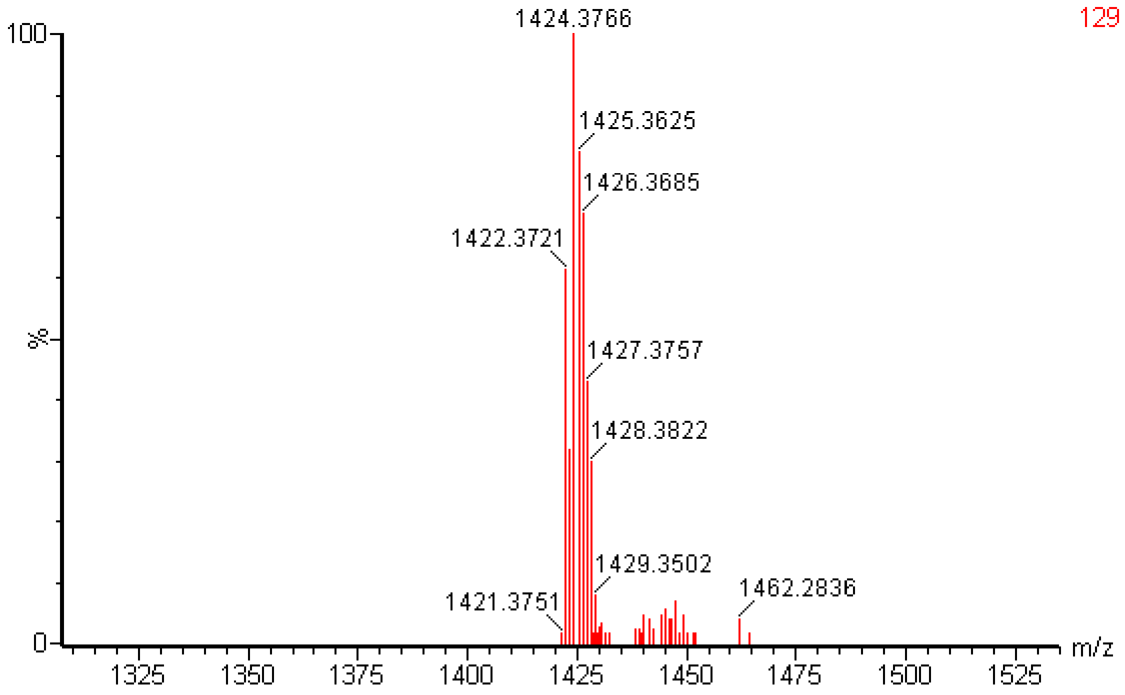

Figure S42. ESI(+) HRMS spectrum of **4a**

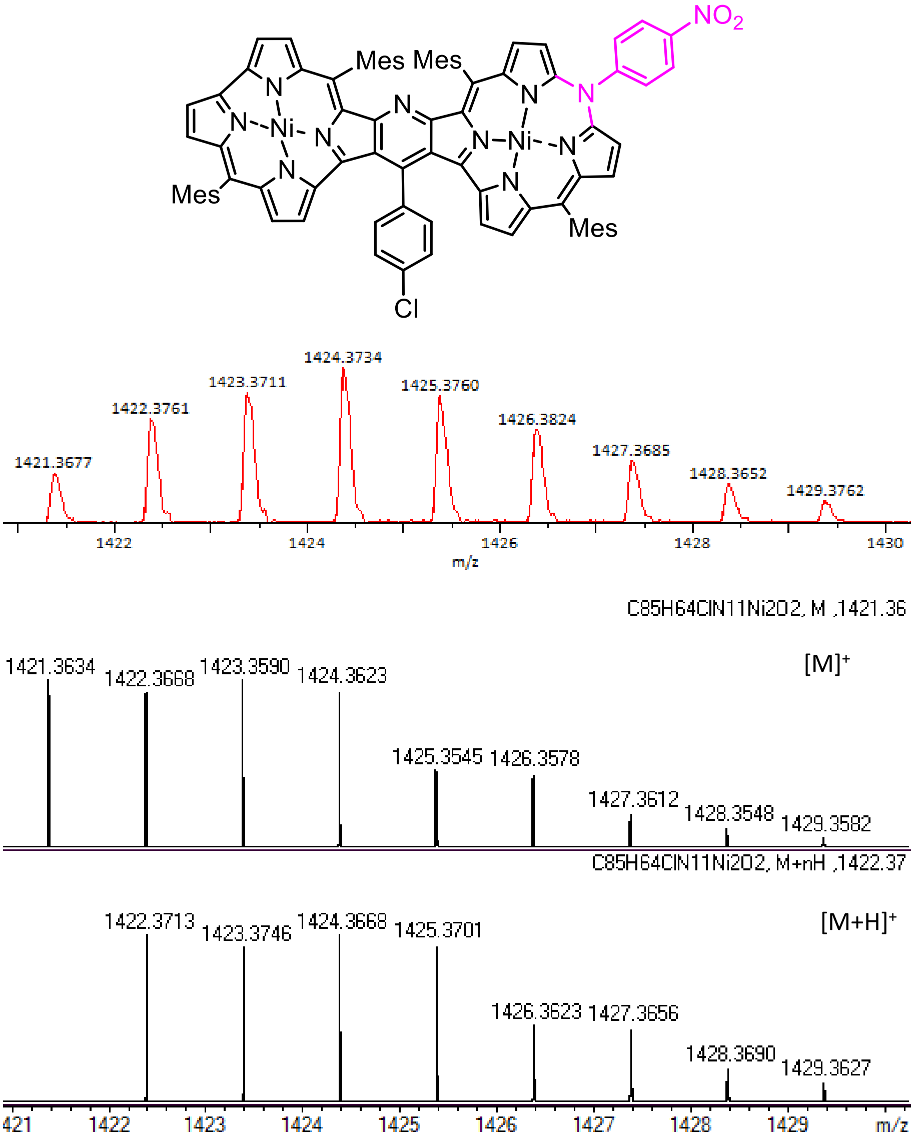

Figure S43. ESI(+) HRMS spectrum of **4b** (experimental: red, upper trace; simulated: black, bottom traces).

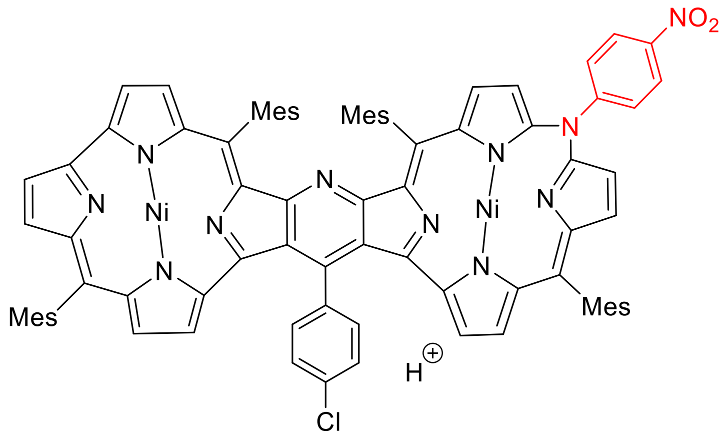

Chemical Formula: $C_{85}H_{65}ClN_{11}Ni_2O_2^+$
Exact Mass: 1422.3713

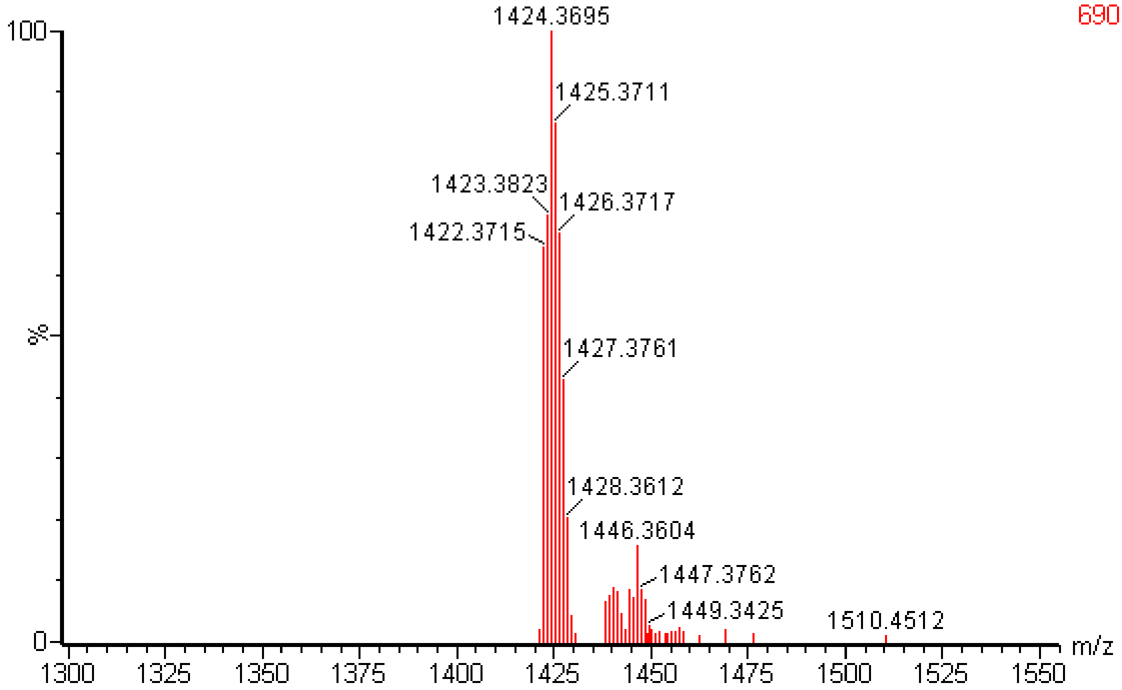

Figure S44. ESI(+) HRMS spectrum of **4b**

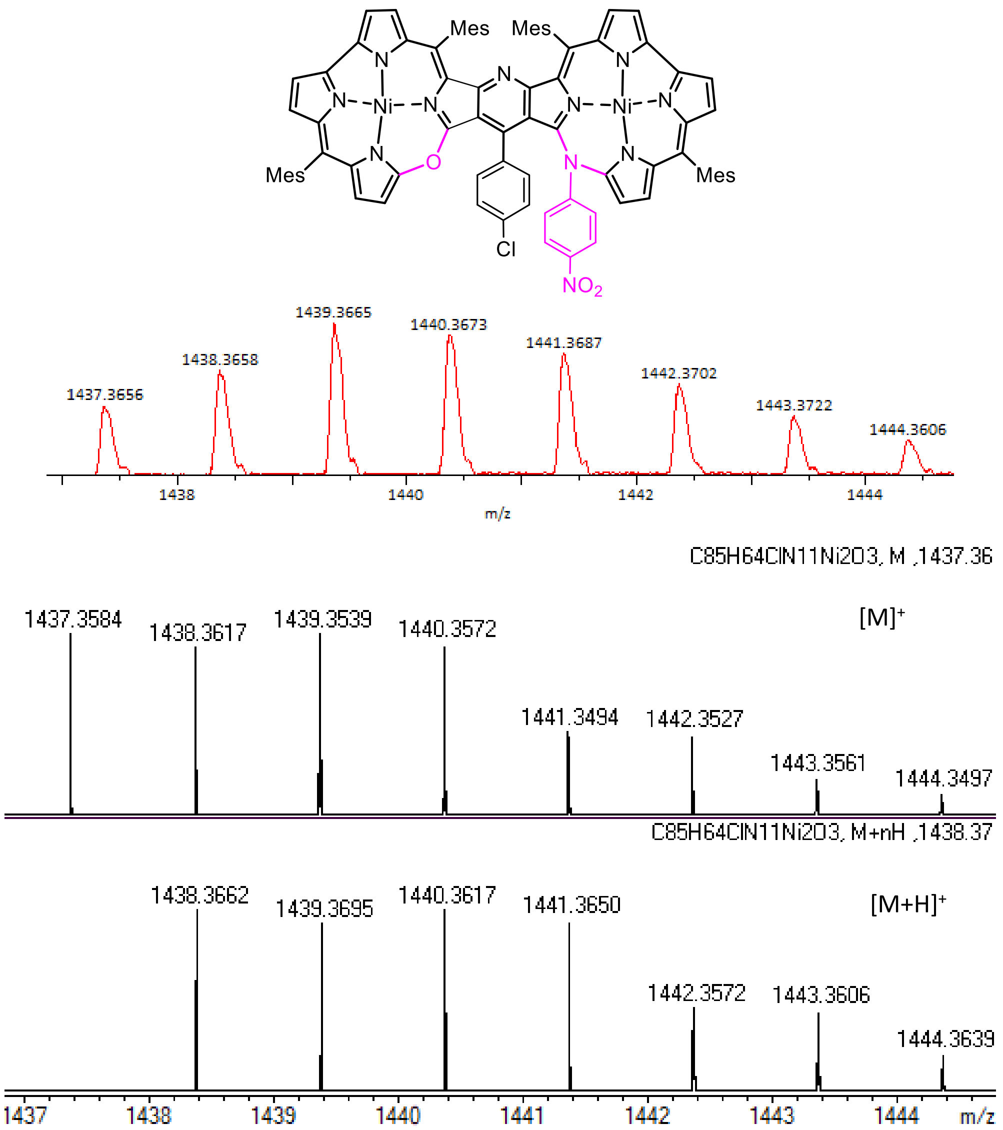

Figure S45. ESI(+) HRMS spectrum of **6a** (experimental: red, upper trace; simulated: black, bottom traces).

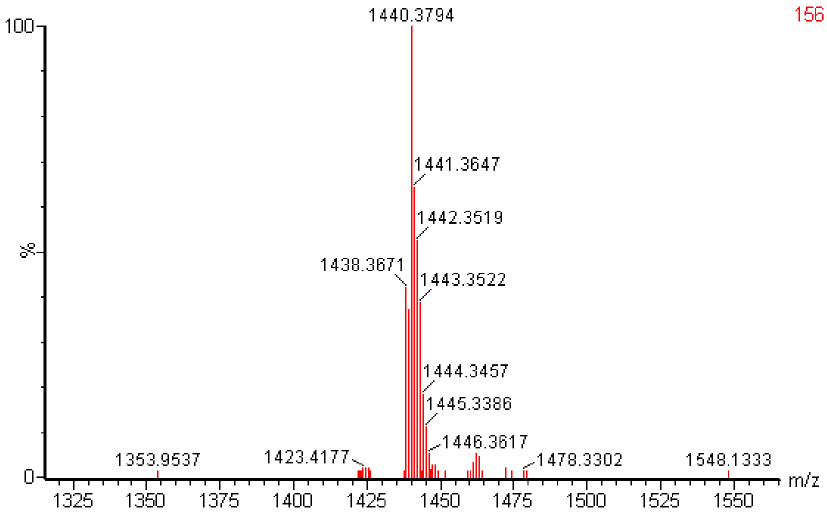

Figure S46. ESI(+) HRMS spectrum of **6a**

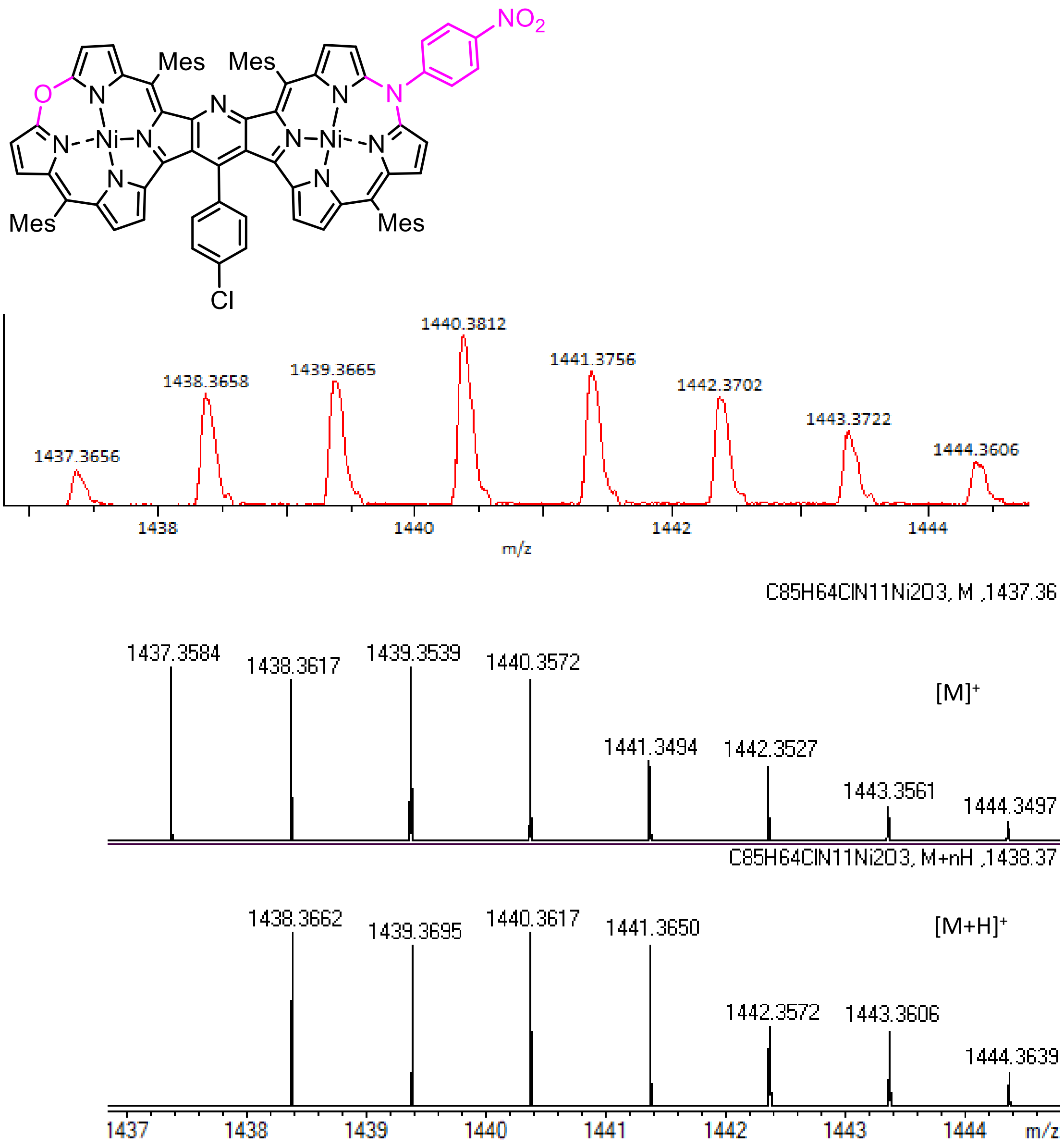

Figure S47. ESI(+) HRMS spectrum of **6b** (experimental: red, upper trace; simulated: black, bottom traces).

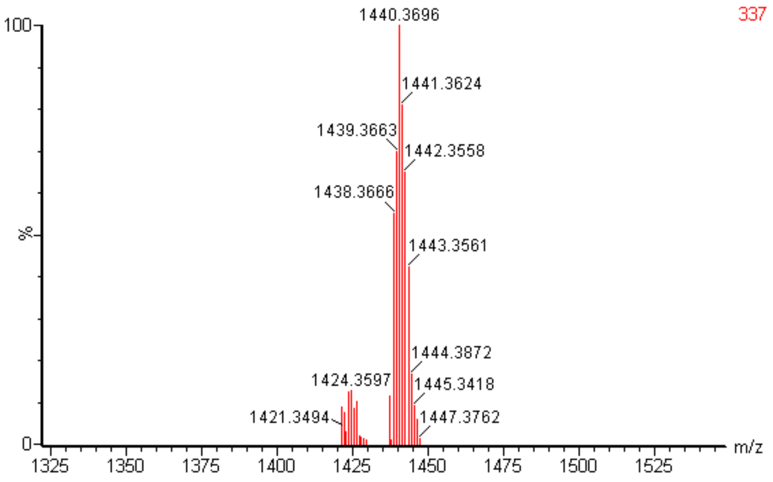

Figure S48. ESI(+) HRMS spectrum of **6b**.

6. Optical spectra for **5aa**, **5ab**, **4a**, **4b**, **6a**, and **6b**

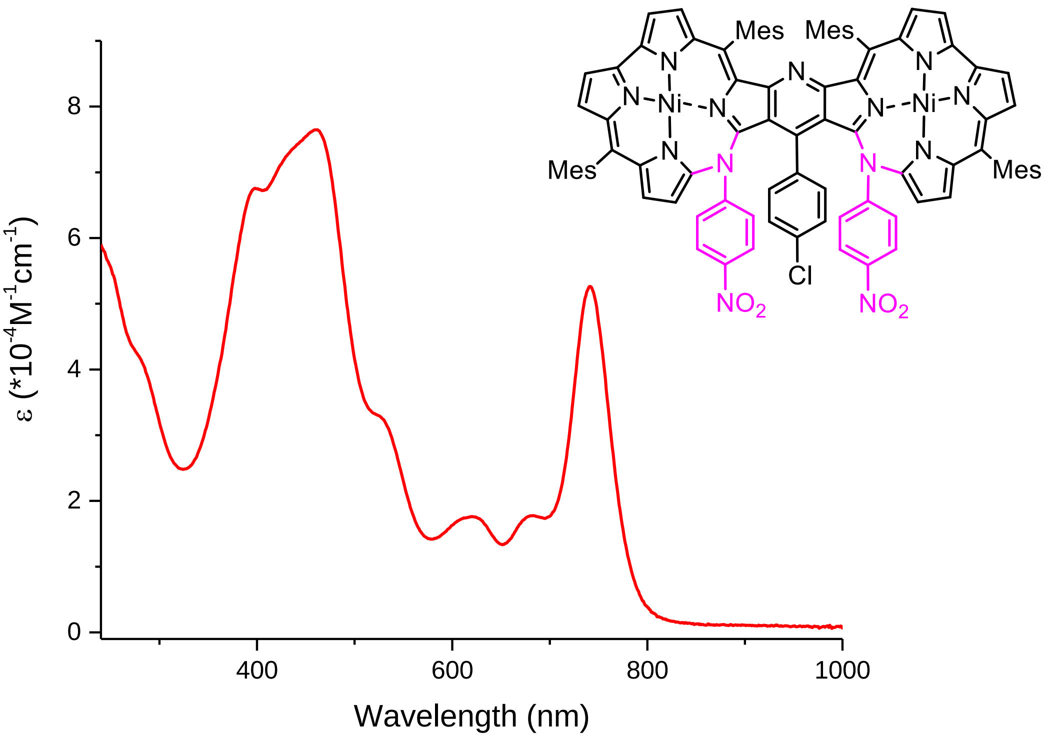

Figure S49. UV-vis-NIR spectrum of **5aa** in dichloromethane.

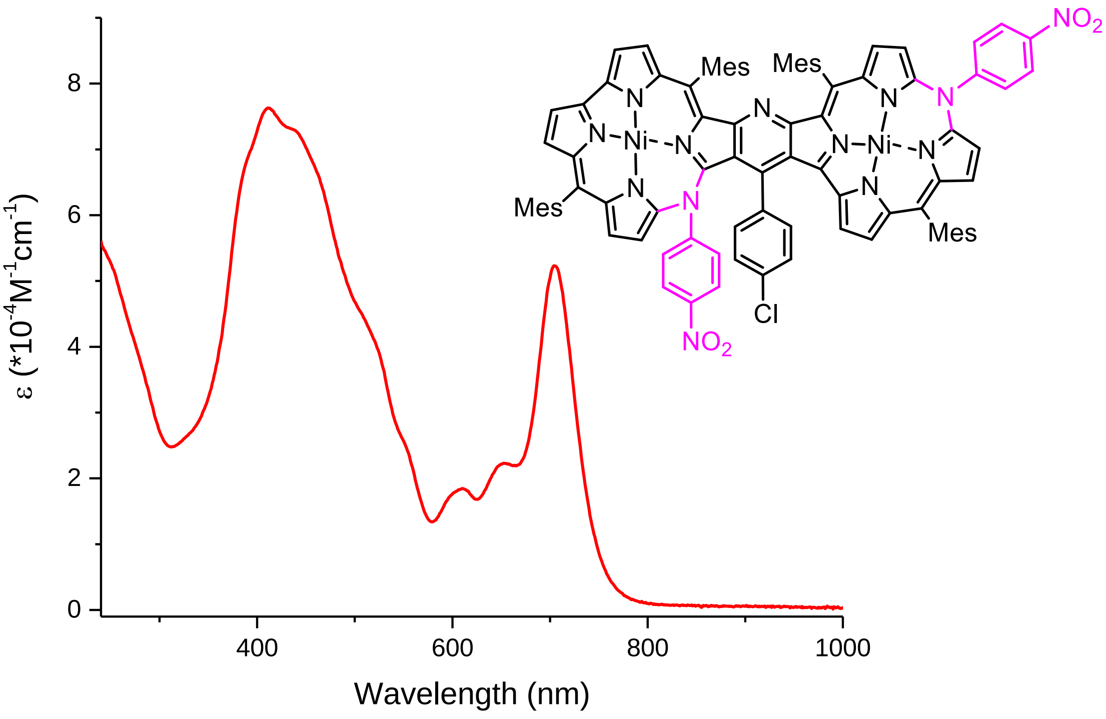

Figure S50. UV-vis spectrum of **5b** in dichloromethane.

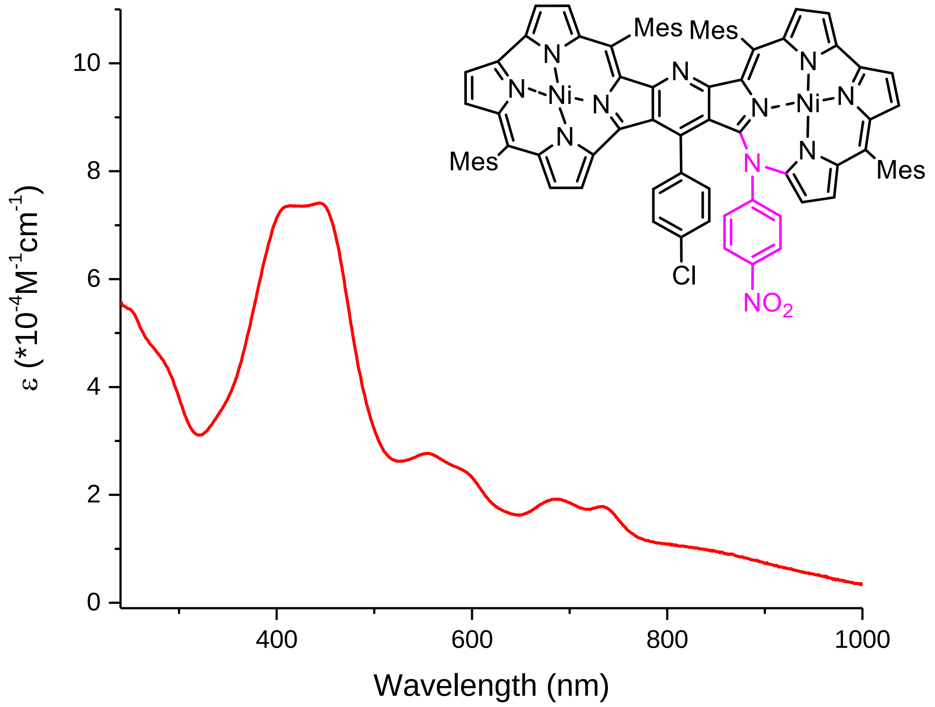

Figure S51. UV-vis spectrum of **4a** in dichloromethane.

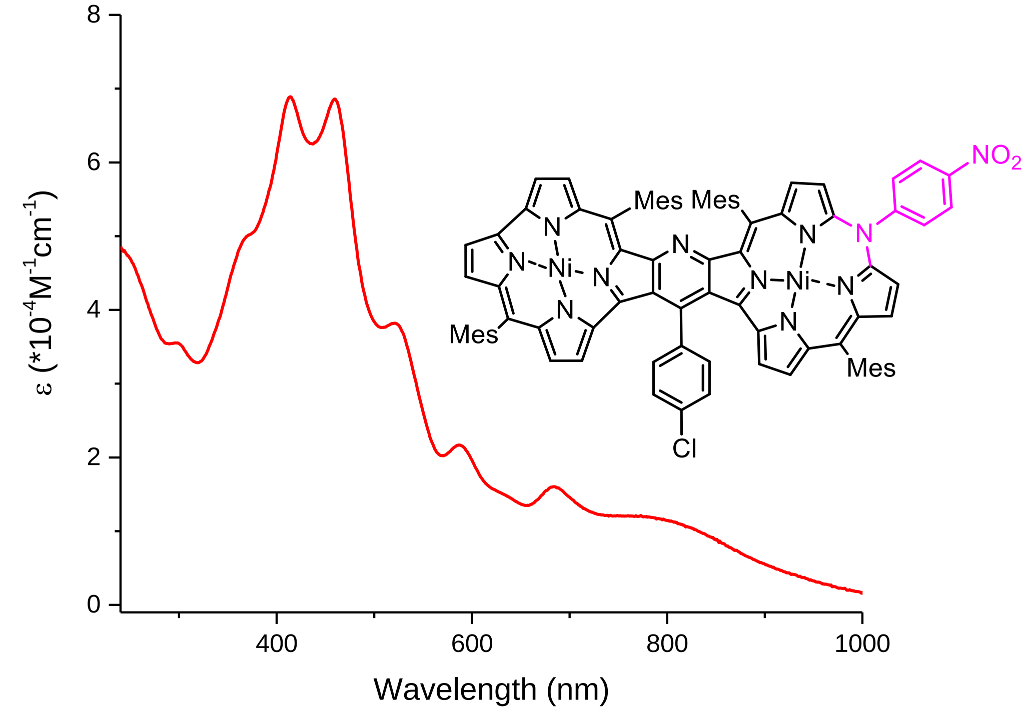

Figure S52. UV-vis spectrum of **4b** in dichloromethane.

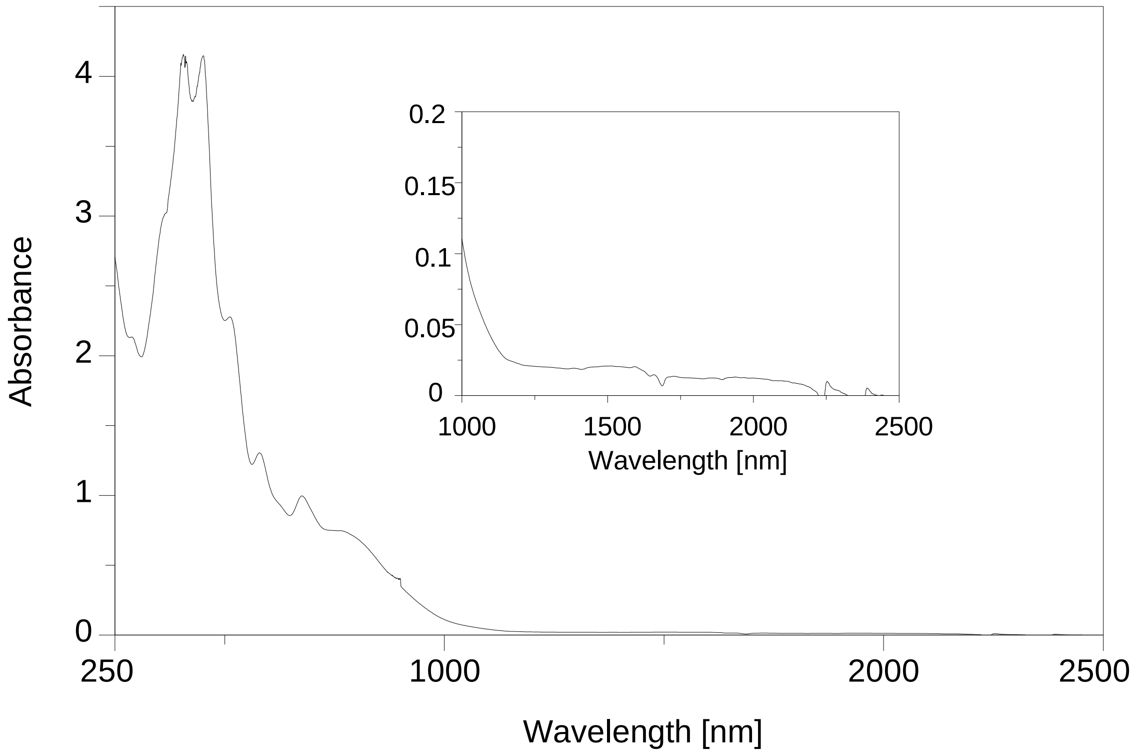

Figure S53. UV-vis-NIR spectrum of **4b** in dichloromethane.

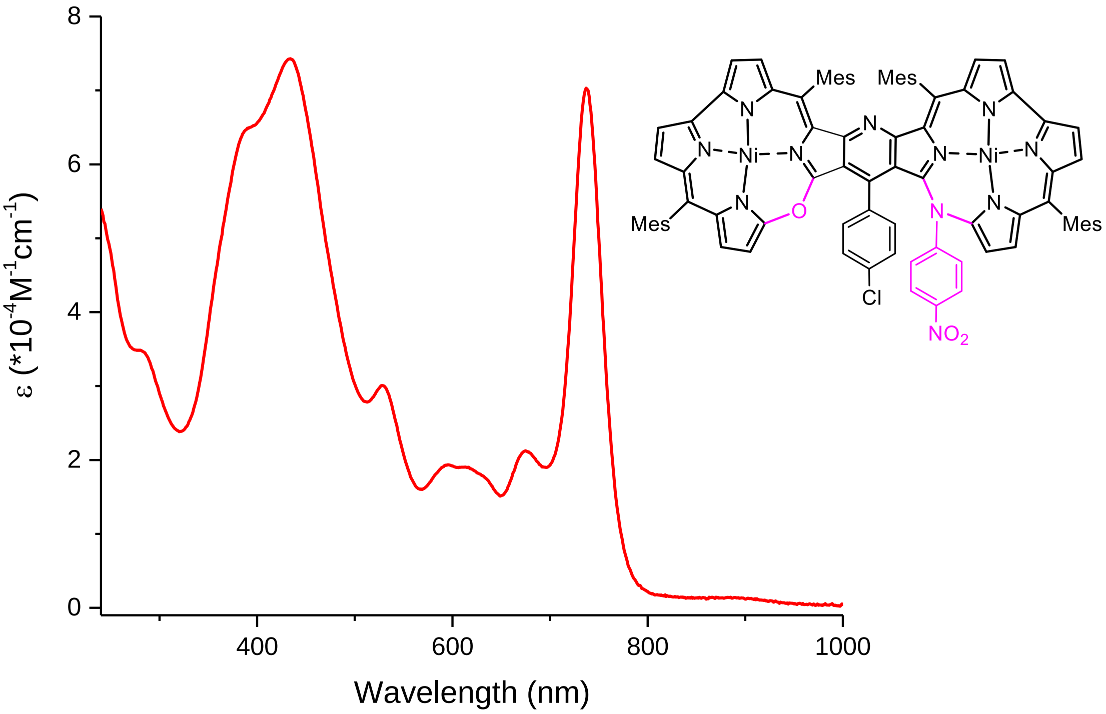

Figure S54. UV-vis spectrum of **6a** in dichloromethane.

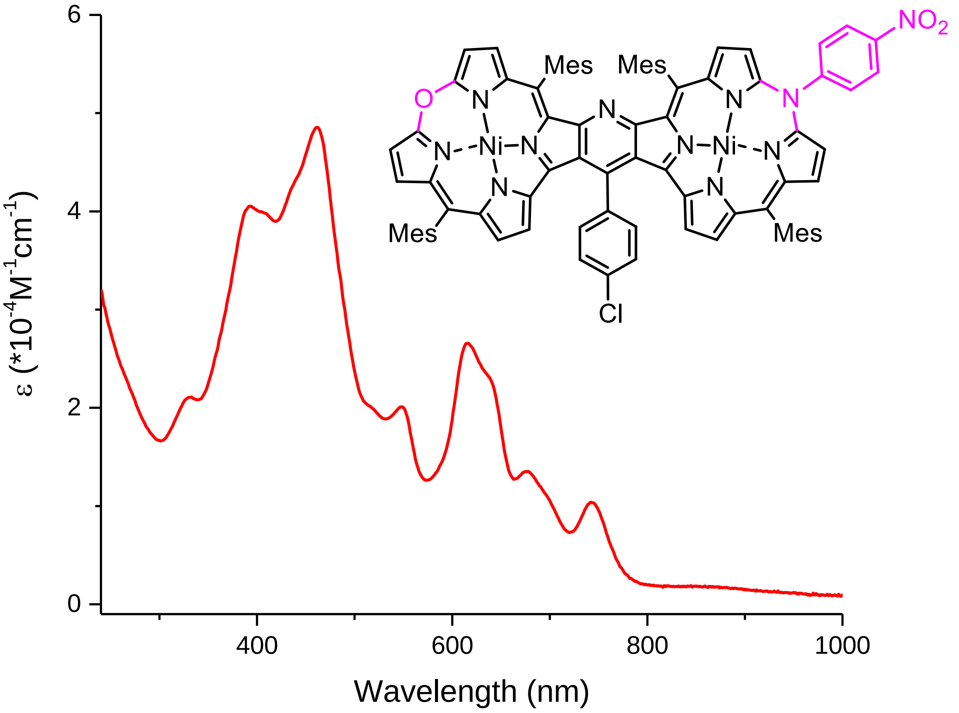

Figure S55. UV-vis spectrum of **6b** in dichloromethane.

7. Synthesis of NO_2-azacorrole: **2-NO$_2$**(3-MePh) and **2-NO$_2$**(3-MeOPh)

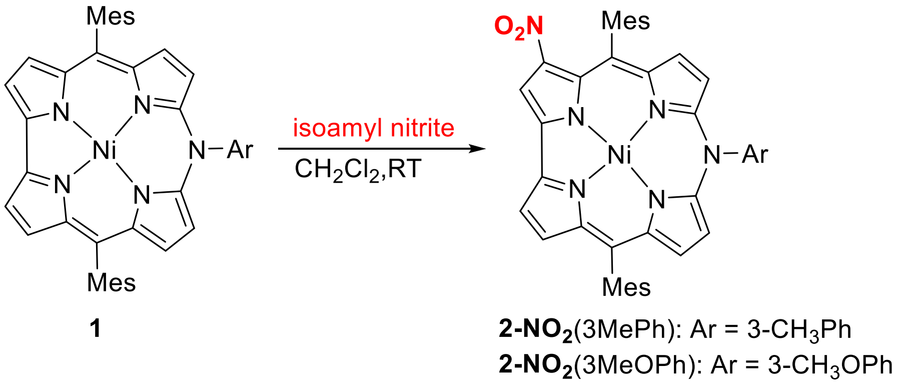

A solution of azacorrole **1** (0.089 mmol) and Isoamyl nitrite (100 uL, 0.74 mmol) in 10 ml CH_2Cl_2 was stirred at room temperature for 3 h, then the solvent was evaporated under vacuum. The residue was subjected to silica-gel column chromatography with petroleum ether/dichloromethane (*v*/*v* = 1:1) as eluent. The first brown fractions were collected and concentrated to obtain **2-NO$_2$**(3-MePh) (Yield: 60 mg, 91%), **2-NO$_2$**(3-MeOPh) (Yield: 53 mg, 80%) .

Selected data for **2-NO₂**(3'-MePh): ^{1}H NMR (500 MHz, 300 K, $CDCl_3$) δ 8.72 (s, 1H), 8.16 (d, *J* = 4.3 Hz, 1H), 7.97 (d, *J* = 2.4 Hz, 1H), 7.95 (d, *J* = 2.1 Hz, 1H), 7.81 (d, *J* = 4.3 Hz, 1H), 7.62 (dt, *J* = 7.5, 3.9 Hz, 4H), 7.22 (d, *J* = 4.9 Hz, 1H), 7.13 (m, 3H), 7.07 (s, 2H), 2.56 (s, 3H), 2.50 (s, 3H), 2.47 (s, 3H), 1.94 (s, 3H), 1.93 (s, 3H), 1.90 (s, 3H), 1.89 (s, 3H). UV-vis (CH_2Cl_2) λ_{max}/nm (logε): 407 (4.69), 449 (4.37), 471 (4.39), 579 (3.94), 644 (3.70). ESI-HRMS calc. for $C_{43}H_{36}N_6NiO_2^+$ $[M]^+$: 726.2248, Found: 726.2248.

Selected data for **2-NO₂**(3-MeOPh ^{1}H NMR (500 MHz, 300 K, $CDCl_3$) δ 8.71 (s, 1H), 8.15 (d, *J* = 4.2 Hz, 1H), 7.96 (d, *J* = 4.2 Hz, 2H), 7.82 (d, *J* = 4.3 Hz, 1H), 7.63 (t, *J* = 8.1 Hz, 1H), 7.43 (ddd, *J* = 7.7, 1.9, 0.9 Hz, 1H), 7.37 (t, *J* = 2.3 Hz, 1H), 7.34 (m, 1H), 7.24 (d, *J* = 4.2 Hz, 1H), 7.17 (d, *J* = 4.7 Hz, 1H), 7.12 (s, 2H), 7.07 (s, 2H), 3.91 (s, 3H), 2.50 (s, 3H), 2.47 (s, 3H), 1.93 (s, 3H), 1.93 (s, 3H), 1.90 (s, 3H), 1.89 (s, 3H). ^{13}C NMR (126 MHz, 300 K, $CDCl_3$) δ 160.2, 146.9, 146.1, 144.4, 143.3, 142.5, 138.2, 138.2, 137.9, 137.7, 137.6, 136.2, 134.9, 134.4, 133.7, 133.0, 132.3, 131.5, 130.0, 127.9, 127.8, 127.8, 126.2, 125.5, 122.1, 117.3, 116.8, 116.5, 115.6, 114.1, 113.6, 55.9, 21.5, 21.4, 21.1, 20.8. UV-vis (CH2Cl2) λmax/nm (logε): 407 (4.76), 444 (4.43), 471 (4.46), 578 (4.01), 644 (3.76). ESI-HRMS calc. for $C_{43}H_{36}N_6NiO3^+$ $[M]^+$: 742.2197, Found: 742.2195.

8. Synthesis of NH_2-azacorrole **2-NH₂**(3-MePh) and **2-NH₂**(3-MeOPh) **(**based on reference[17])

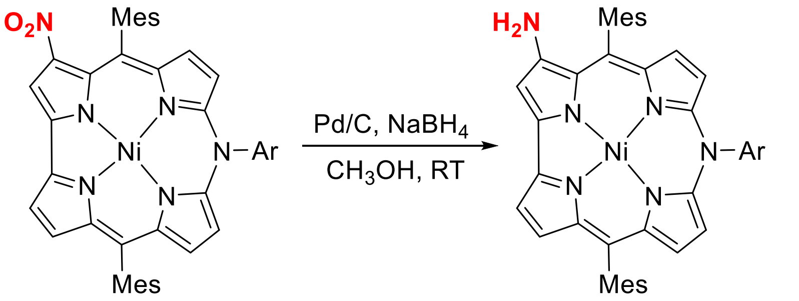

2-NO₂(3MePh)
2-NO₂(3MeOPh)

2-NH₂(3MePh): Ar=3CH_3Ph)
2-NH₂(3MeOPh): Ar=3CH_3OPh)

A mixture of NO_2-azacorrole (0.08 mmol) and Pd/C (10%, 60 mg) in CH_2Cl_2 (24 ml) and MeOH (6 ml) was purged with N_2 for 10 min. Sodium borohydride (130 mg, 3.4 mmol) was added and the solution was stirred under nitrogen at room temperature for 10 min. After complete consumption of the starting material (TLC), the mixture was passed through a short pad of silica using CH_2Cl_2 as eluent. All the brown fractions were collected together and concentrated to obtain the product NH_2-azacorrole **2-NH₂**(3-MePh) (Yield: 40 mg, 71%), **2-NH₂**(3-MeOPh) (Yield:36 mg, 63%).

Selected data for **2-NH₂**(3'-MePh): ^{1}H NMR (500 MHz, 300 K, CDCl3) δ 8.20 (d, J = 4.2 Hz, 1H), 8.05 (d, J = 4.9 Hz, 1H), 7.81 (m, 1H), 7.65 (m, 2H), 7.58 (m, 3H), 7.24 (d, J = 4.7 Hz, 1H), 7.19 (s, 2H), 7.13 (m, 2H), 7.08 (m, 1H), 2.51 (m, 12H), 1.96 (m, 6H), 1.91 (m, 6H). ^{13}C NMR (126

MHz, 300 K, CDCl3) δ 144.49, 142.67, 139.12, 138.46, 138.30, 138.22, 137.36, 137.25, 135.64, 134.47, 134.29, 133.65, 132.83, 132.10, 131.93, 130.64, 128.91, 128.77, 128.69, 127.66, 127.55, 127.17, 123.28, 116.92, 114.18, 110.11, 21.47, 21.42, 21.04, 20.67. UV-vis (CH_2Cl_2) λ_{max}/nm (logε): 396 (5.01), 456 (4.47), 577 (3.78), 679 (3.66). ESI-HRMS calc. for $C_{43}H_{38}N_6Ni^+$ $[M]^+$: 696.2506, Found: 696.2508.

Selected data for **2-NH₂**(3'-MeOPh): ^{1}H NMR (500 MHz, 300K, $CDCl_3$) δ 8.19 (d, *J* = 4.2 Hz, 1H), 8.05 (d, *J* = 4.8 Hz, 1H), 7.80 (d, *J* = 4.1 Hz, 1H), 7.59 (m, 2H), 7.46 (m, 1H), 7.40 (m, 1H), 7.29 (m, 5H), 7.19 (s, 3H), 7.13 (m, 2H), 3.89 (s, 3H), 2.51 (s, 6H), 1.96 (s, 6H), 1.92 (s, 6H). ^{13}C NMR (126 MHz, 300 K, $CDCl_3$) δ 160.0, 138.2, 138.2, 137.4, 134.3, 132.2, 129.5, 128.8, 127.7, 127.6, 122.6, 116.9, 116.0, 115.8, 114.1, 55.8, 21.5, 21.4, 21.0, 20.7.UV-vis (CH2Cl2) λmax/nm (logε): 396 (4.97), 456 (4.43), 577 (3.74), 679 (3.63). ESI-HRMS calc. for C43H38N6NiO$^+$ $[M]^+$: 712.2455, Found: 712.2455.

9. General procedure of synthesis and characterization of pyridine-fused bis(azacorrole) from 3-amino-10-azacorrole

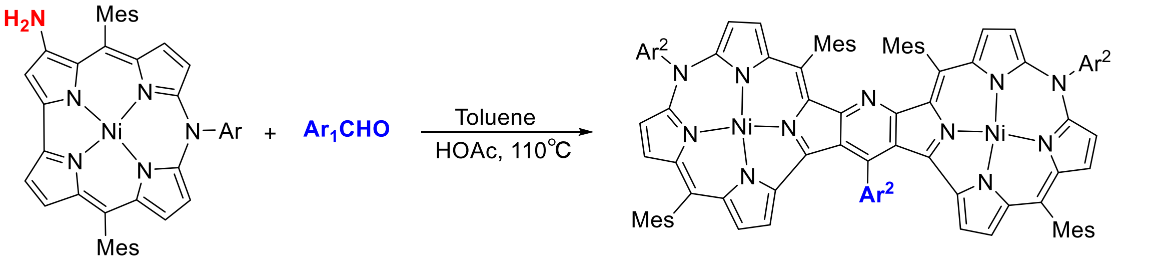

2-NH₂(3MePh): Ar = 3-CH_3Ph
2-NH₂(3MeOPh): Ar = 3-CH_3OPh

5bb-1: Ar^1 = 3-CH_3Ph, Ar^2 = 4-ClPh
5bb-2: Ar^1 = 3-CH_3OPh, Ar^2 = 4-ClPh
5bb-3: Ar^1 = 3-CH_3Ph, Ar^2 = 3,4,5-$(OCH_3)_3$Ph
5bb-4: Ar^1 = 3-CH_3OPh, Ar^2 = 3,4,5-$(OCH_3)_3$Ph

A solution of NH_2-azacorrole (0.014 mmol), and aromatic aldehyde (0.07 mmol, 5 eq.) in 3 mL toluene in the presence of HOAc (80 μL) was refluxed at 110°C for 60 min. The solvent was then evaporated under vacuum. The residue was washed with CH_3OH to remove excess of aromatic aldehyde, then separated by preparative TLC with dichloromethane as eluent to afford the desired product **5bb-1** (Yield: 3.1 mg, 30%), **5bb-2** (Yield:2.6 mg, 25%), **5bb-3** (Yield: 2.9 mg, 27%), and **5bb-4** (Yield:2.2 mg, 21%).

Selected data for **5bb-1**: ^{1}H NMR (500 MHz, 300 K, $CDCl_3$) δ 8.32 – 8.27 (m, 2H), 8.16 (d, *J* = 4.6 Hz, 2H), 8.06 (d, *J* = 7.8 Hz, 2H), 7.73 (d, *J* = 4.4 Hz, 2H), 7.72 – 7.66 (m, 4H), 7.65 – 7.57 (m, 4H), 7.52 (d, *J* = 4.5 Hz, 2H), 7.38 (d, *J* = 4.7 Hz, 2H), 7.19 (d, *J* = 4.5 Hz, 2H), 7.13 (s, 4H), 7.03 (s, 4H), 6.33 (d, *J* = 4.7 Hz, 2H), 2.75 (s, 6H), 2.56 (s, 6H), 2.52 (s, 6H), 1.90 (s, 6H), 1.89 (s, 6H), 1.74 (s, 12H). ^{13}C NMR (126 MHz, 300, $CDCl_3$) δ 153.6, 143.8, 142.5, 142.1, 141.0, 139.7, 139.5, 139.1, 138.6, 138.2, 137.5, 136.2, 136.1, 135.4, 135.4, 134.0, 133.5, 133.0, 132.0, 131.0, 130.8, 130.6, 129.7, 128.7, 127.7, 127.6, 127.5, 127.1, 125.1, 124.6, 123.9, 122.4, 119.5, 114.7, 111.0, 22.8, 22.1, 21.4, 21.1, 20.8. UV-vis (CH_2Cl_2) λ_{max}/nm (logε): 392 (4.86),

434 (4.83), 462 (4.86), 538 (4.56), 652 (4.77), 700 (4.41). API-HRMS calc. for $C_{93}H_{75}ClN_{11}Ni_2^+$ $[M+H]^+$: 1496.4597, Found: 1496.4597.

Selected data for **5bb-2**: ^1H NMR (500 MHz, 300 K, $CDCl_3$) δ 8.30 (d, J = 7.9 Hz, 2H), 8.16 (d, J = 4.6 Hz, 2H), 8.06 (d, J = 7.9 Hz, 2H), 7.73 (d, J = 4.4 Hz, 2H), 7.62 (t, J = 8.1 Hz, 2H), 7.52 (d, J = 4.8 Hz, 2H), 7.51 – 7.47 (m, 2H), 7.44 (t, J = 2.2 Hz, 2H), 7.42 (d, J = 4.6 Hz, 2H), 7.33 (dd, J = 8.6, 2.5 Hz, 2H), 7.23 (d, J = 4.7 Hz, 2H), 7.13 (s, 4H), 7.04 (s, 4H), 6.33 (d, J = 4.4 Hz, 2H), 3.90 (s, 6H), 2.75 (s, 6H), 2.52 (s, 6H), 1.90 (s, 12H), 1.74 (s, 12H). ^{13}C NMR (126 MHz, 300 K, $CDCl_3$) δ 159.9, 153.6, 143.7, 143.6, 142.2, 140.8, 139.8, 139.5, 138.6, 138.6, 138.1, 137.5, 136.2, 136.1, 135.4, 134.7, 134.0, 134.0, 133.6, 133.0, 132.1, 131.0, 129.8, 129.5, 127.7, 127.7, 127.5, 125.3, 124.7, 123.9, 122.5, 122.4, 119.5, 116.3, 115.7, 114.6, 110.9, 55.8, 22.1, 21.4, 21.1, 20.8. UV-vis (CH_2Cl_2) λ_{max}/nm (logε): 392 (4.82), 434 (4.78), 462 (4.79), 538 (4.47), 654 (4.67), 700 (4.35). API-HRMS calc. for $C_{93}H_{75}ClN_{11}Ni_2O_2^+$ $[M+H]^+$: 1528.4495, Found: 1528.4496.

Selected data for **5bb-3**: ^1H NMR (500 MHz, 300K, $CDCl_3$) δ 8.16 (d, J = 4.7 Hz, 2H), 7.73 (d, J = 4.0 Hz, 2H), 7.68 (m, 4H), 7.60 (m, 4H), 7.57 (s, 2H), 7.52 (s, 2H), 7.38 (d, J = 4.3 Hz, 2H), 7.19 (s, 2H), 7.15 (s, 4H), 7.04 (s, 4H), 6.63 (d, J = 4.4 Hz, 2H), 4.31 (s, 3H), 3.81 (s, 6H), 2.75 (s, 6H), 2.56 (s, 6H), 2.53 (s, 6H), 1.91 (s, 6H), 1.90 (s, 6H), 1.75 (s, 6H), 1.74 (s, 6H). ^{13}C NMR (126 MHz, 300K, $CDCl_3$) δ 155.4, 143.7, 142.5, 140.3, 139.1, 138.7, 138.2, 137.5, 136.3, 135.4, 133.9, 133.5, 131.9, 130.8, 130.6, 129.6, 128.7, 127.7, 127.6, 127.6, 127.6, 127.1, 123.9, 120.3, 114.5, 110.9, 108.8, 61.9, 56.8, 22.1, 21.4, 21.4, 21.1, 20.9. UV-vis (CH_2Cl_2) λ_{max}/nm (logε): 392 (4.84), 434 (4.82), 462 (4.86), 538 (4.58), 654 (4.76), 700 (4.40). API-HRMS calc. for $C_{96}H_{81}N_{11}Ni_2O_3^+$ $[M]^+$: 1551.5225, Found: 1551.5227.

Selected data for **5bb-4**: ^1H NMR (500 MHz, 300 K, $CDCl_3$) δ 8.15 (d, J = 4.7 Hz, 2H), 7.72 (d, J = 4.4 Hz, 2H), 7.61 (t, J = 8.1 Hz, 2H), 7.56 (s, 2H), 7.51 (d, J = 4.5 Hz, 2H), 7.49 (d, J = 7.8 Hz, 2H), 7.43 (s, 2H), 7.41 (d, J = 4.7 Hz, 2H), 7.33 (dd, J = 8.6, 2.5 Hz, 2H), 7.14 (s, 4H), 7.04 (s, 6H), 6.61 (d, J = 4.4 Hz, 2H), 4.31 (s, 3H), 3.91 (s, 6H), 3.81 (m, 6H), 2.75 (s, 6H), 2.53 (s, 6H), 1.91 (s, 12H), 1.76 (s, 3H), 1.75 (s, 3H), 1.75 (s, 3H), 1.74 (s, 3H). ^{13}C NMR (126 MHz, 300 K, $CDCl_3$) δ 159.9, 155.4, 143.6, 140.3, 138.6, 138.1, 137.5, 136.2, 135.4, 133.9, 133.5, 132.0, 129.5, 127.7, 127.6, 123.9, 122.5, 120.3, 116.2, 115.7, 114.5, 110.8, 108.8, 61.9, 56.8, 55.8, 22.1, 21.4, 21.1, 20.8. UV-vis (CH_2Cl_2) λ_{max}/nm (logε): 392 (4.90), 434 (4.89), 462 (4.93), 538 (4.64), 654 (4.82), 700 (4.47). API-HRMS calc. for $C_{96}H_{81}N_{11}Ni_2O_5^+$ $[M]^+$: 1583.5124, Found: 1583.5125.

10. NMR spectra of **5bb** and precursors

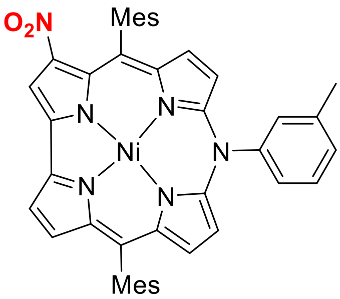

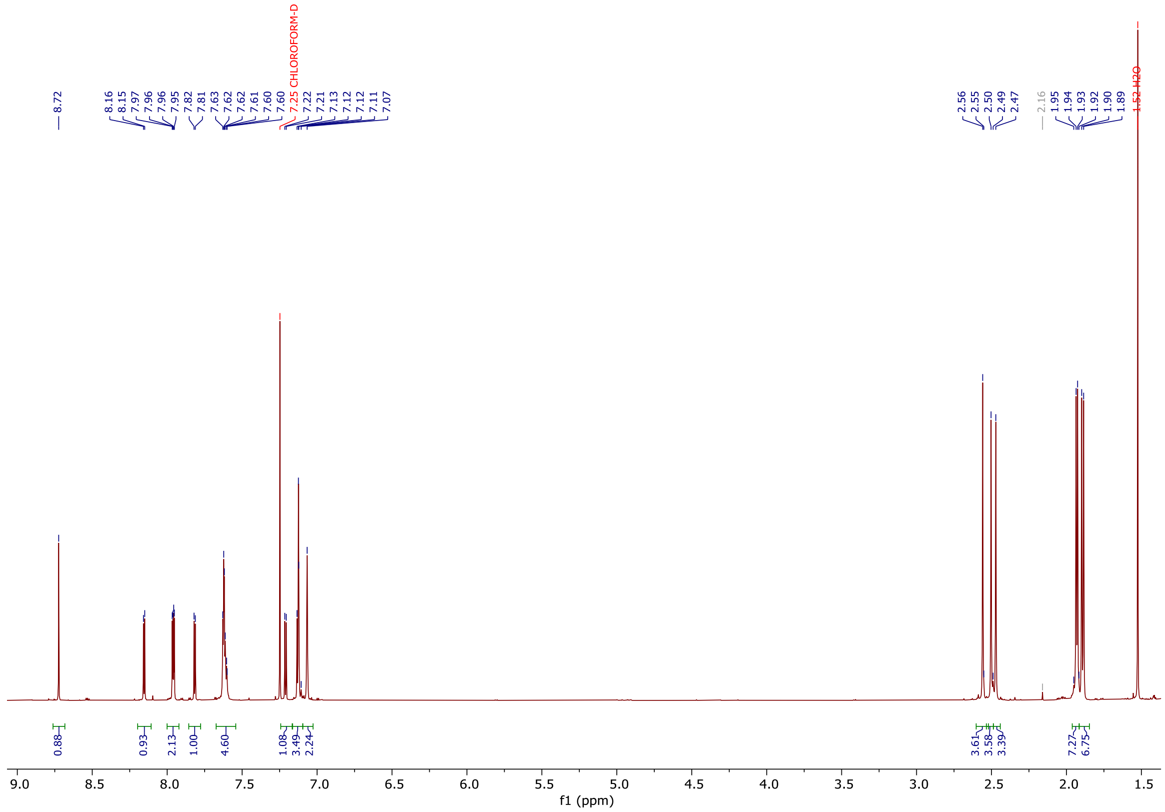

Figure S56. ^{1}H NMR spectrum (500 MHz, $CDCl_3$, 300 K) of **2**-NO_2(3-MePh).

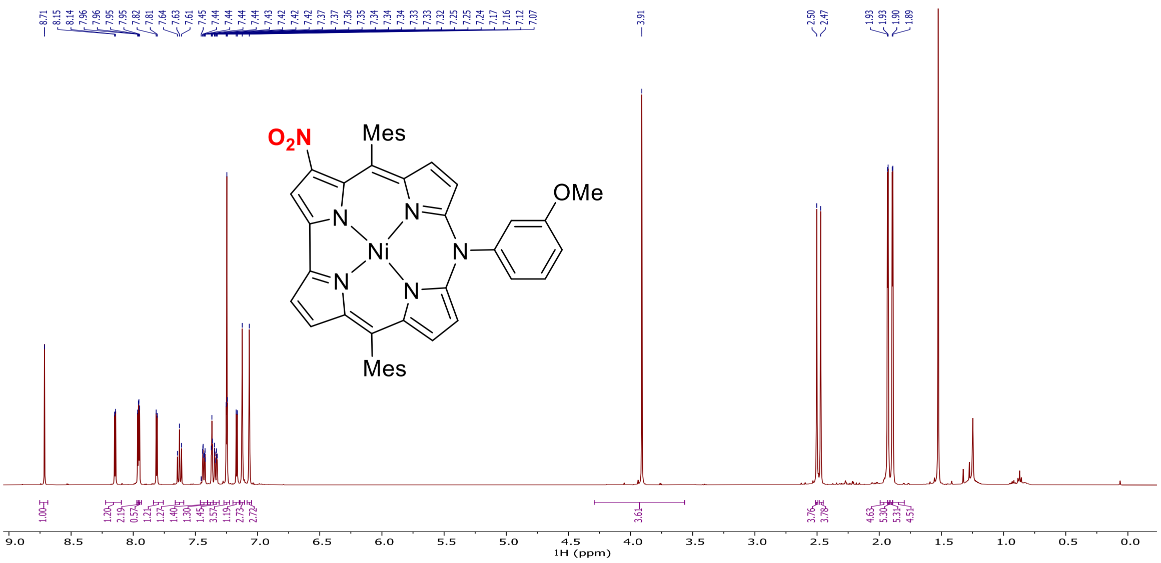

Figure S57. ^{1}H NMR spectrum (500 MHz, $CDCl_3$, 300 K) of **2**-NO_2(3-MeOPh).

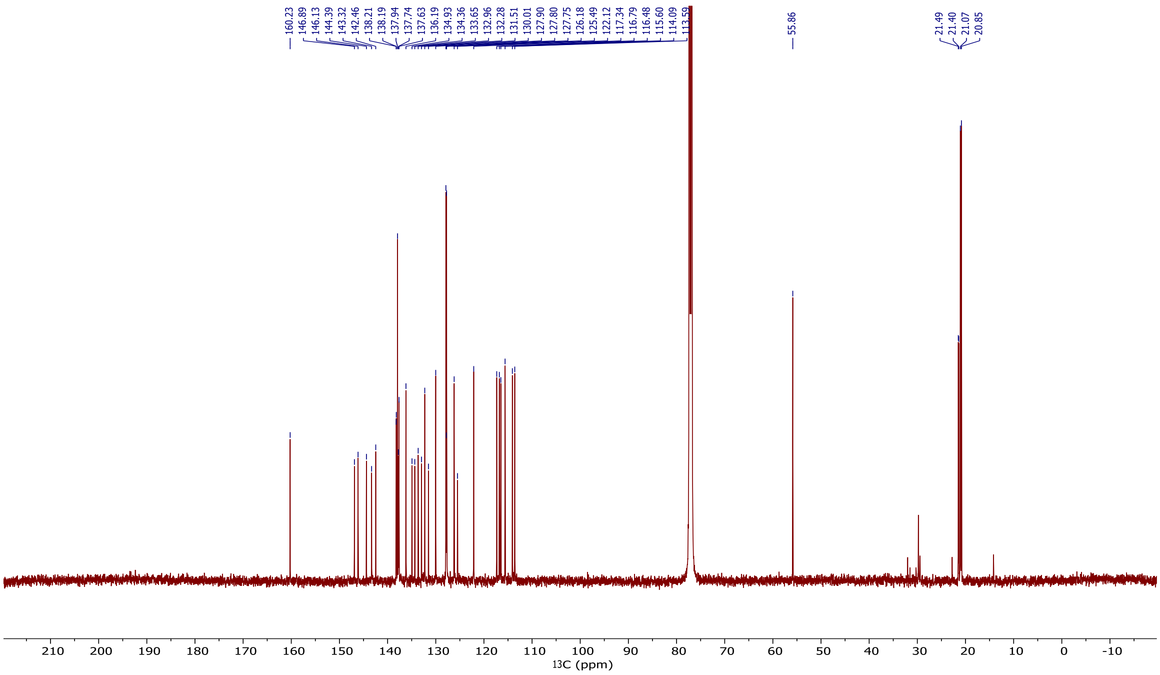

Figure S58. ^{13}C NMR spectrum (126 MHz, $CDCl_3$, 300 K) of **2**-NO_2(3-MeOPh).

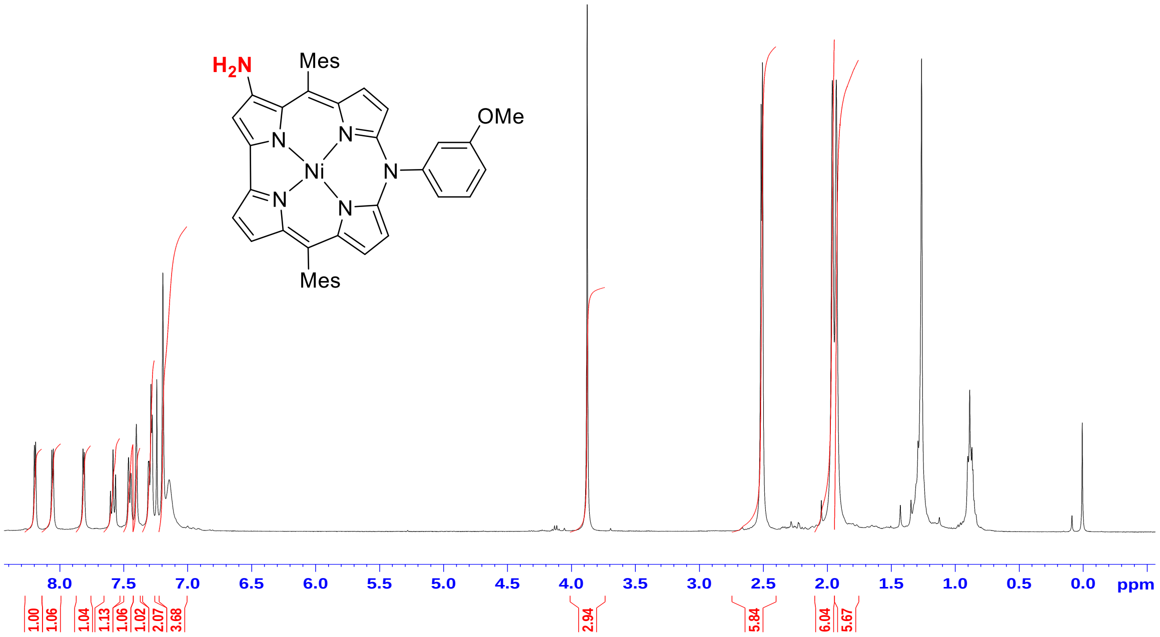

Figure S59. ^{1}H NMR spectrum (500 MHz, $CDCl_3$, 300 K) of **2**-NH_2(3-MeOPh).

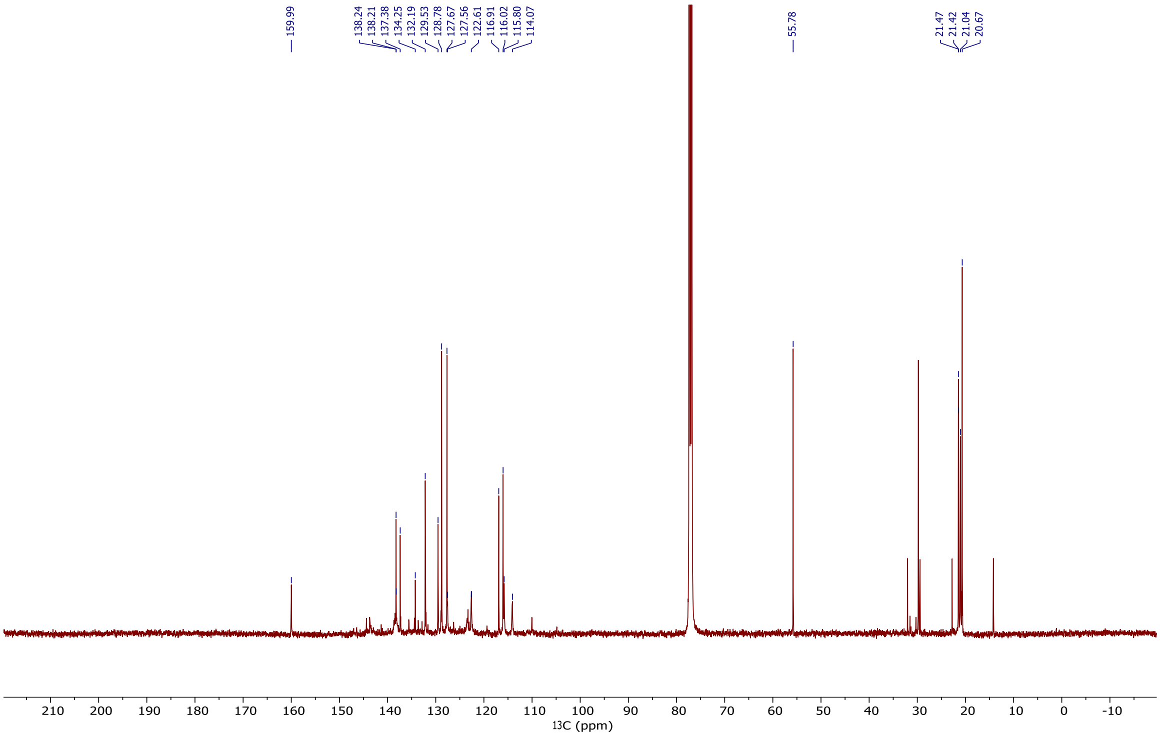

Figure S60. ^{13}C NMR spectrum (126 MHz, $CDCl_3$, 300 K) of **2**-NH_2(3-MeOPh).

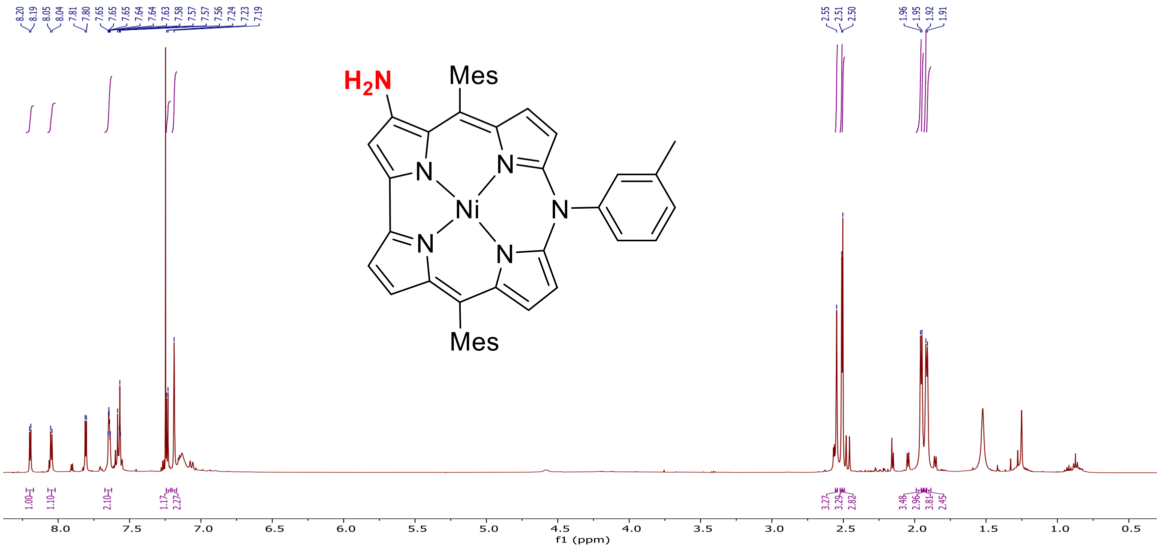

Figure S61. ^{1}H NMR spectrum (500 MHz, $CDCl_3$, 300 K) of **2**-NH_2(3-MePh)

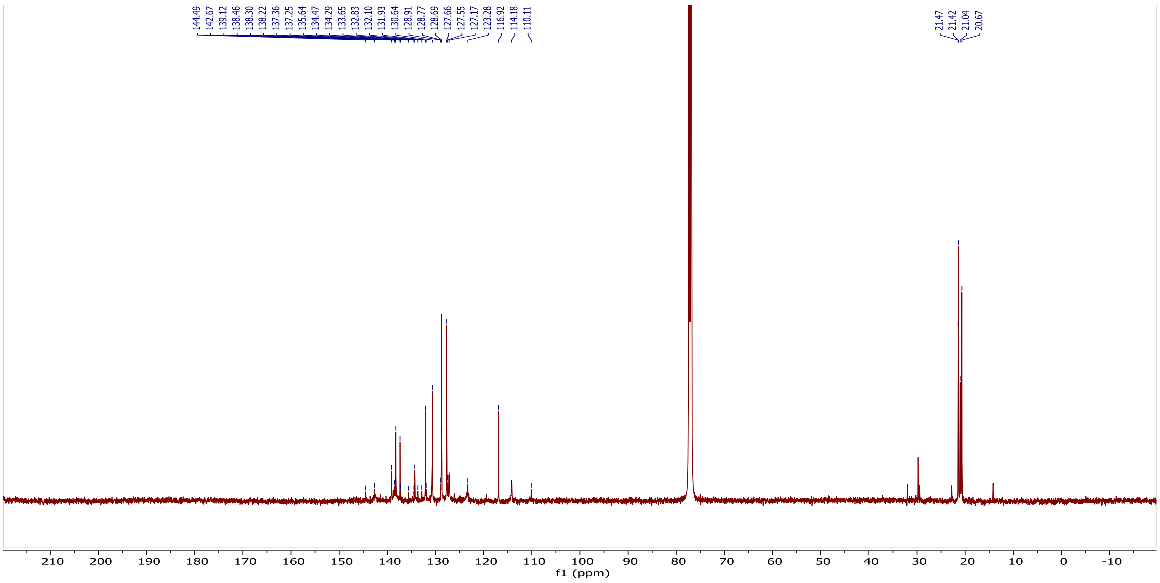

Figure S62. ^{13}C NMR spectrum (126 MHz, $CDCl_3$, 300 K) of **2**-NH_2(3-MePh).

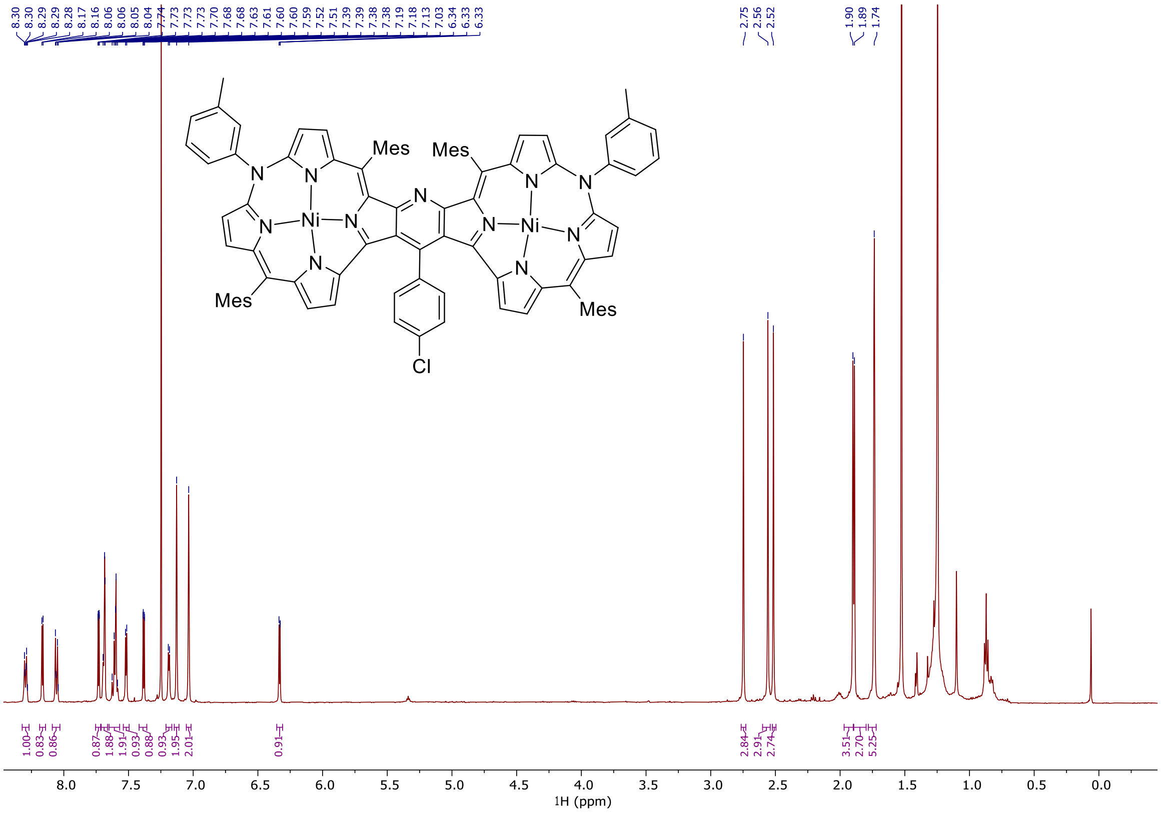

Figure S63. ^{1}H NMR spectrum (500 MHz, $CDCl_3$, 300 K) of **5bb-1**.

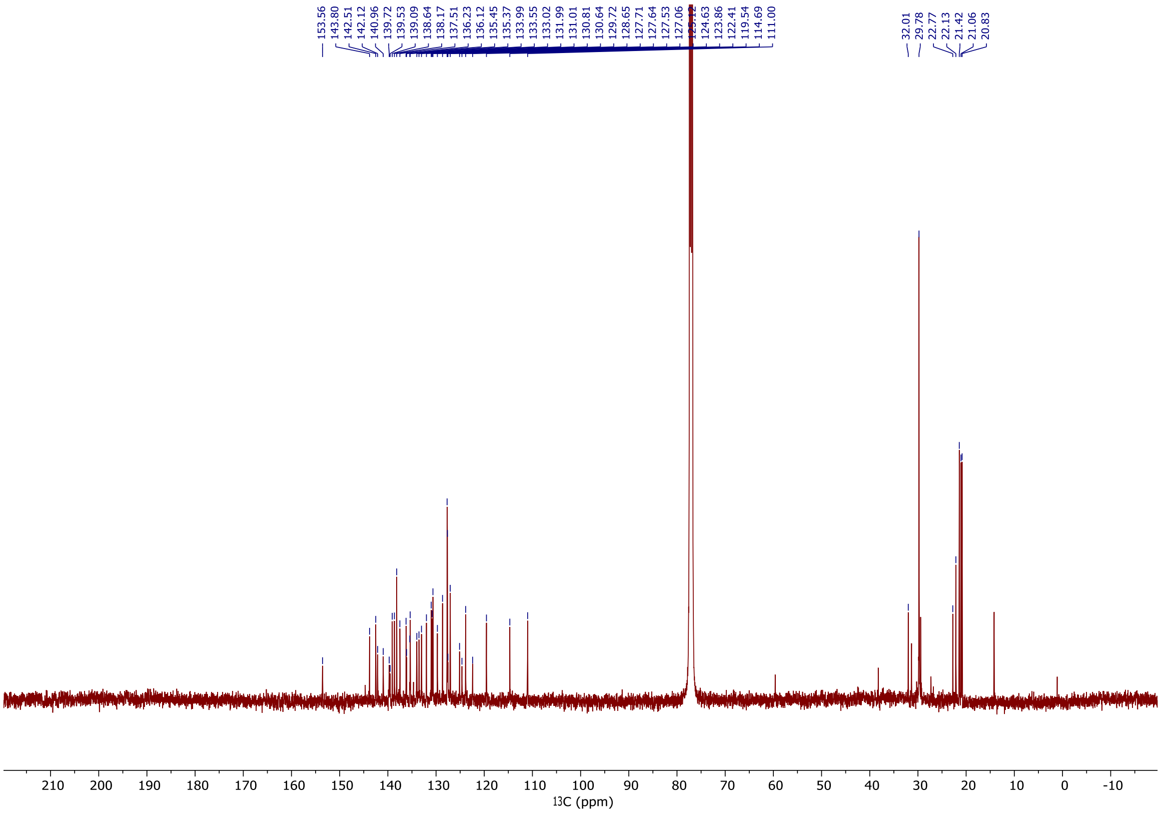

Figure S64. ^{13}C NMR spectrum (126 MHz, $CDCl_3$, 300 K) of **5bb-1**.

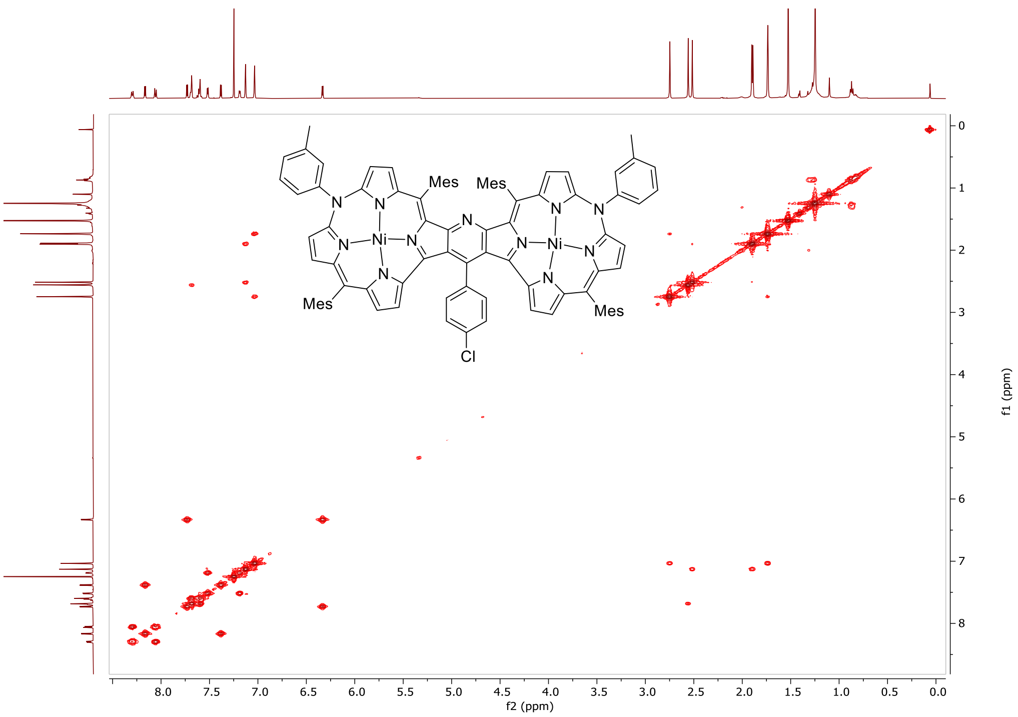

Figure S65. $^{1}H,^{1}H$ COSY spectrum (500 MHz, $CDCl_3$, 300 K) of **5bb-1**.

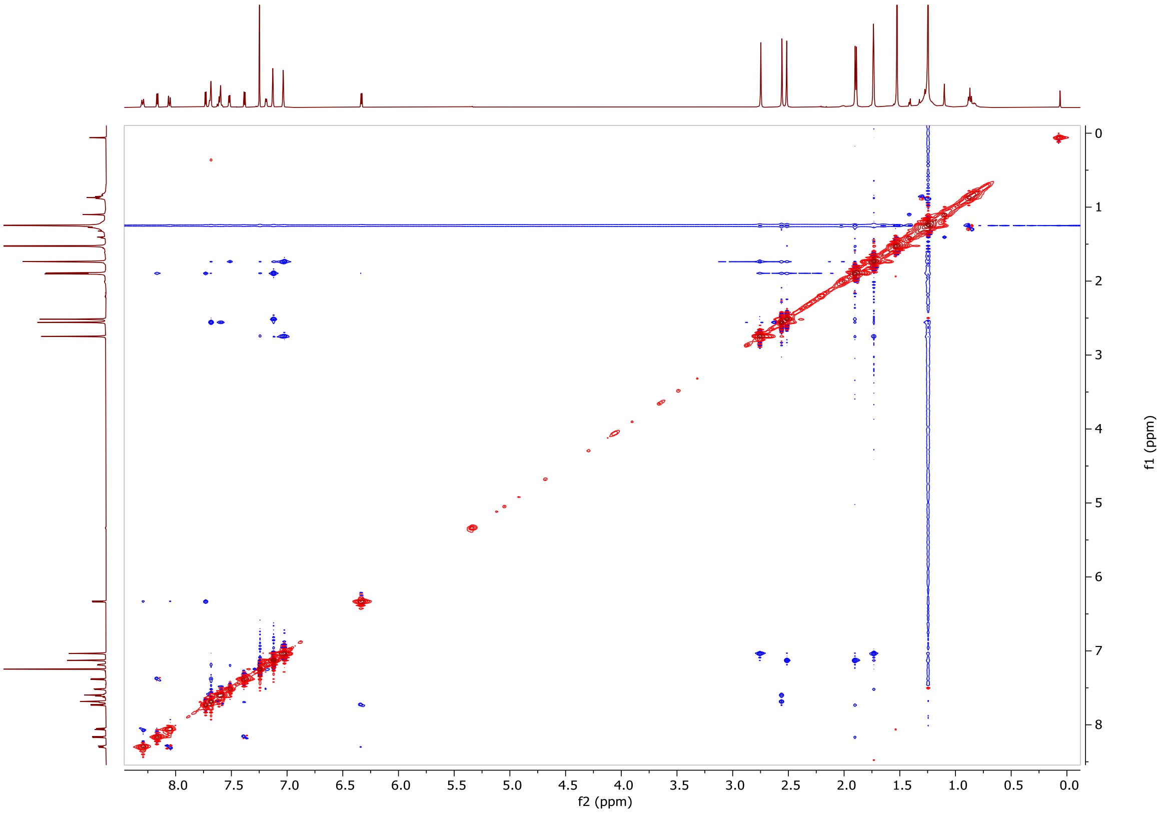

Figure S66. $^{1}H,^{1}H$ ROESY spectrum (500 MHz, $CDCl_3$, 300 K) of **5bb-1**.

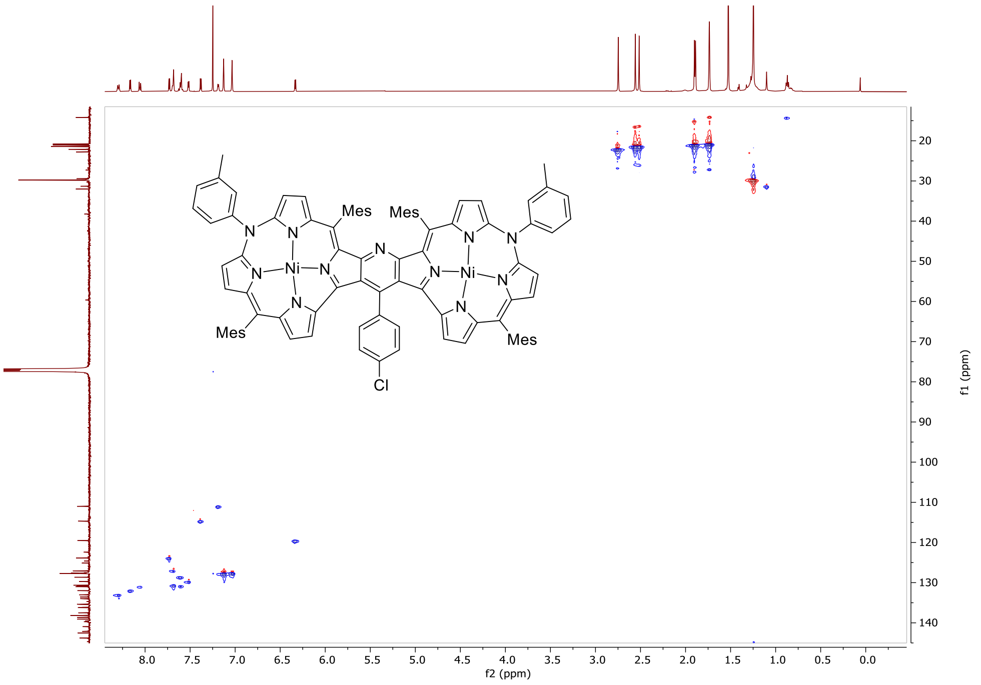

Figure S67. $^{1}H,^{13}C$ HSQC spectrum (500/126 MHz, $CDCl_3$, 300 K) of **5bb-1**.

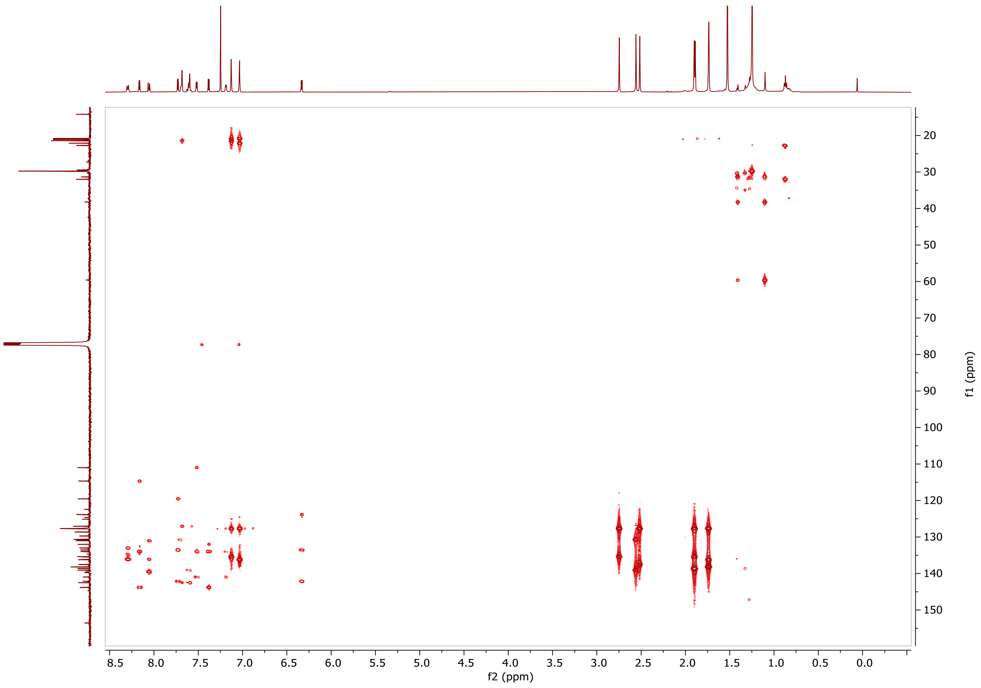

Figure S68. $^{1}H,^{13}C$ HMBC spectrum (500/126 MHz, $CDCl_3$, 300 K) of **5bb-1**.

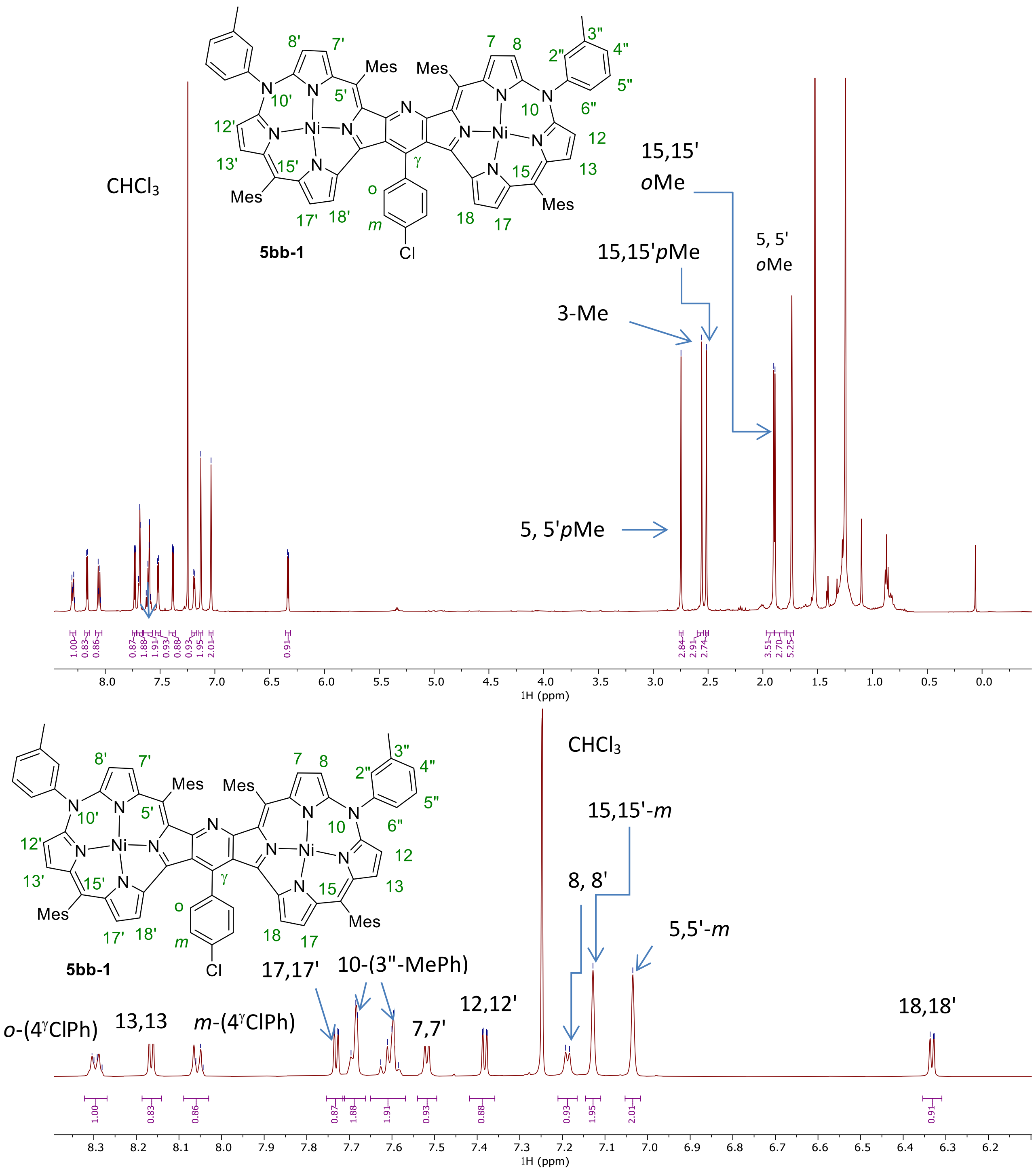

Figure S69. ^{1}H NMR signal assignments (500 MHz, $CDCl_3$, 300 K) for **5bb-1**.

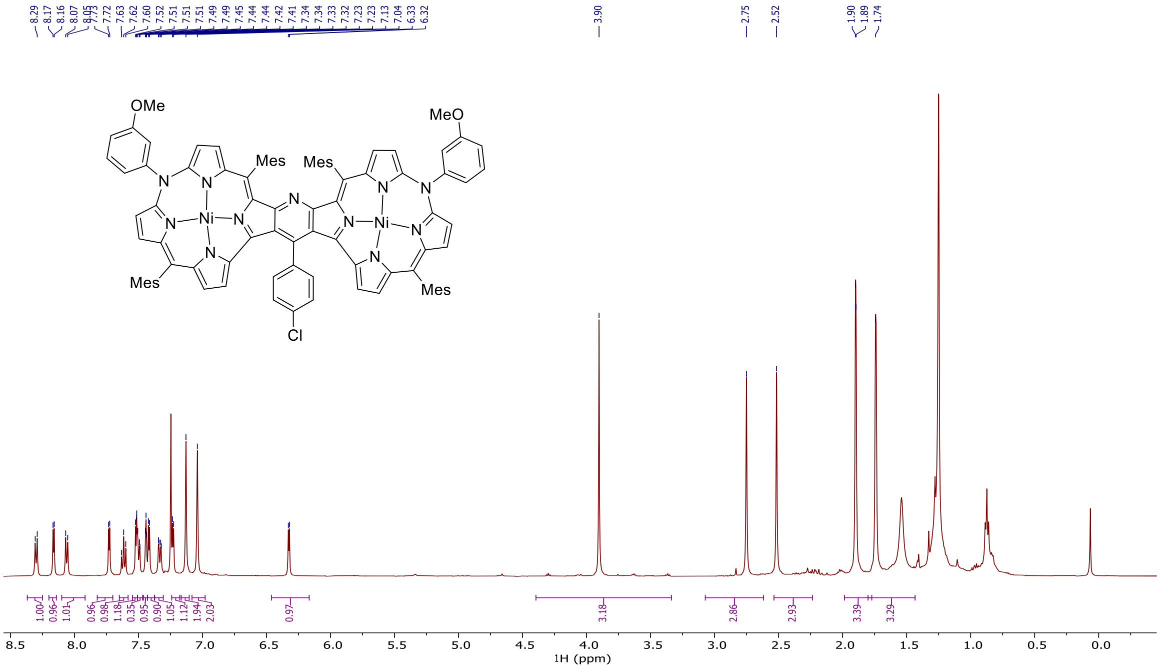

Figure S70. ^{1}H NMR spectrum (500 MHz, $CDCl_3$, 300 K) of **5bb-2**.

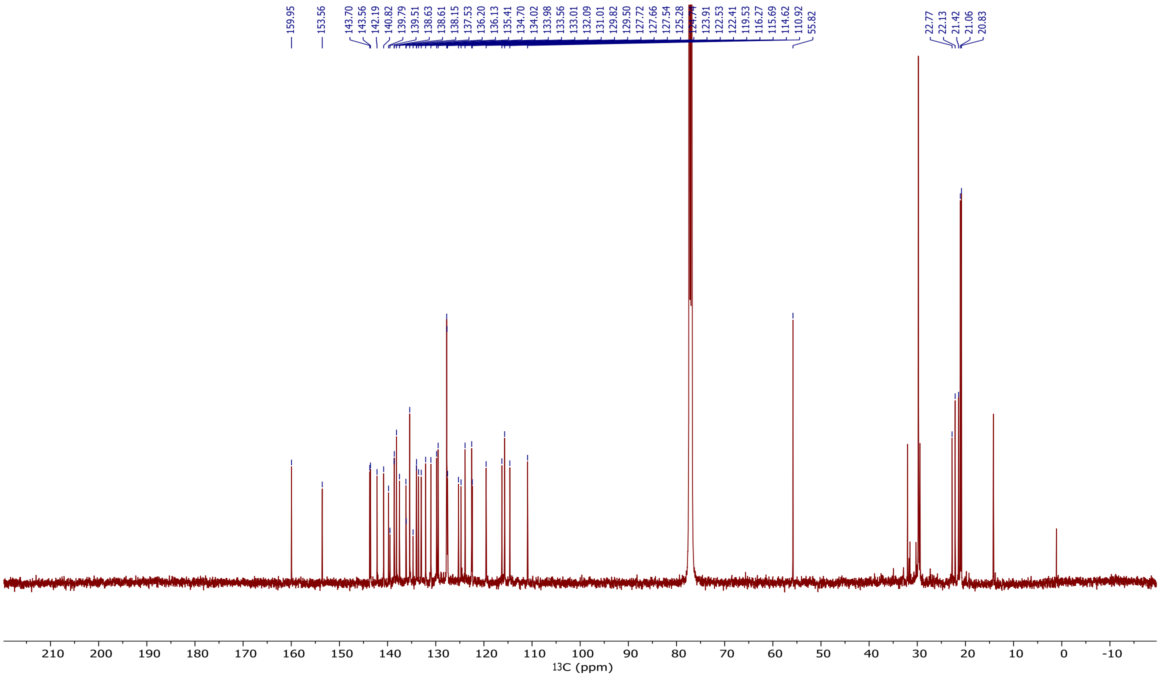

Figure S71. ^{13}C NMR spectrum (126 MHz, $CDCl_3$, 300 K) of **5bb-2**.

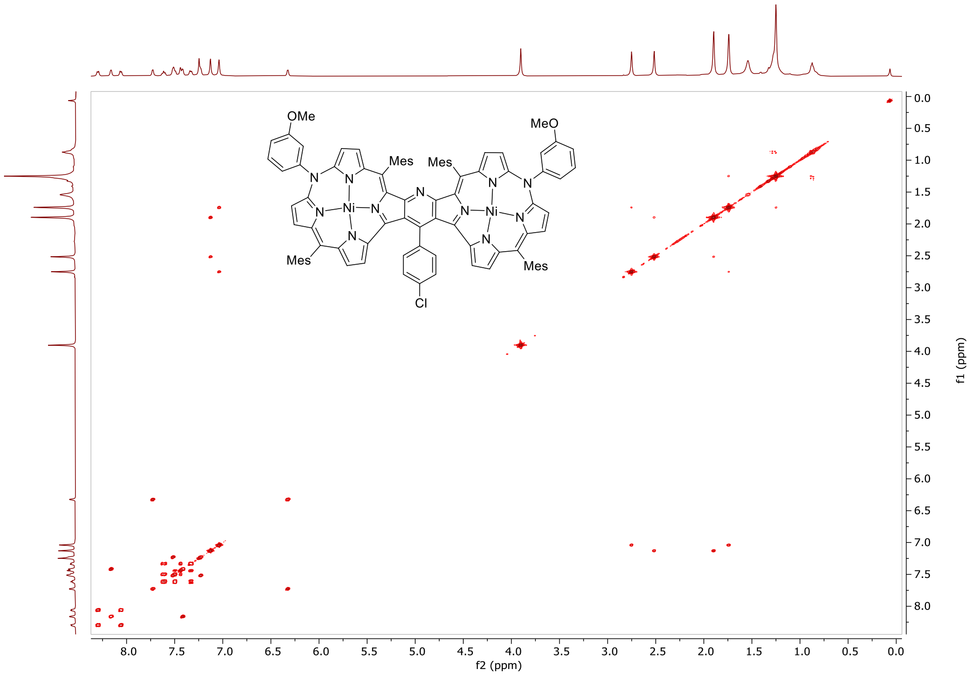

Figure S72. ^{1}H,^{1}H COSY spectrum (500 MHz, $CDCl_3$, 300 K) of **5bb-2**.

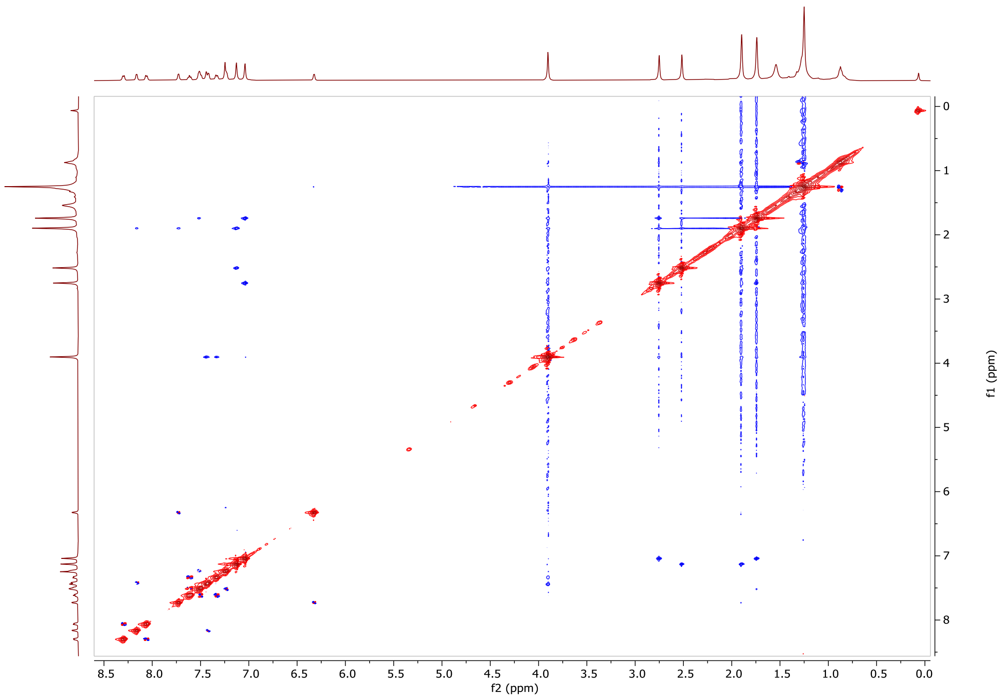

Figure S73. ^{1}H,^{1}H ROESY spectrum (500 MHz, $CDCl_3$, 300 K) of **5bb-2**.

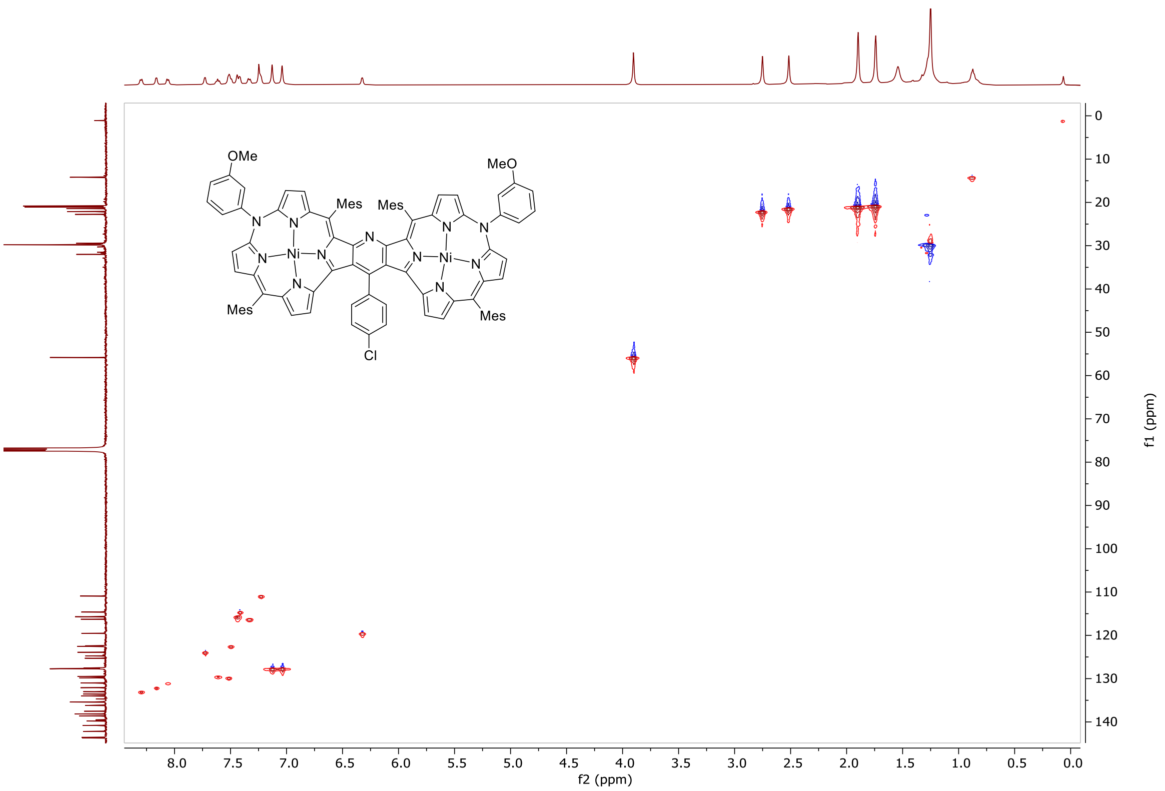

Figure S74. ^{1}H,^{13}C HSQC spectrum (500/126 MHz, $CDCl_3$, 300 K) of **5bb-2**.

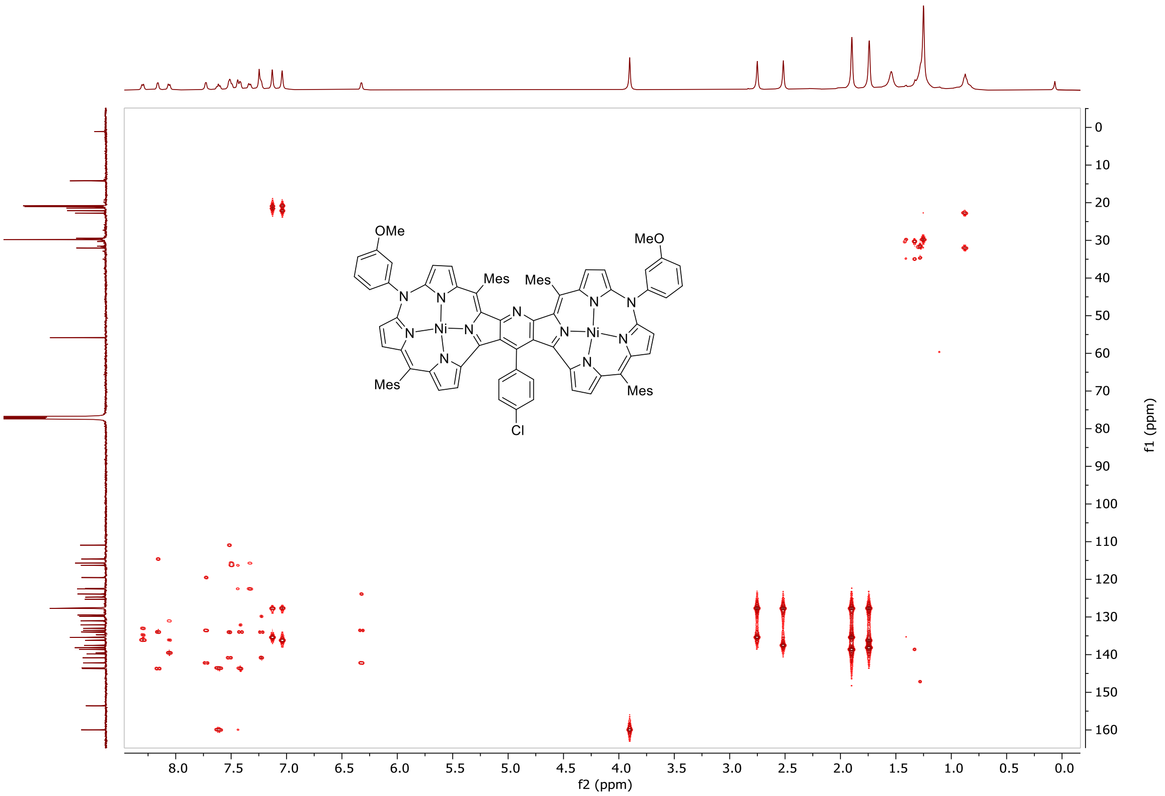

Figure S75. ^{1}H,^{13}C HMBC spectrum (500/126 MHz, $CDCl_3$, 300 K) of **5bb-2**.

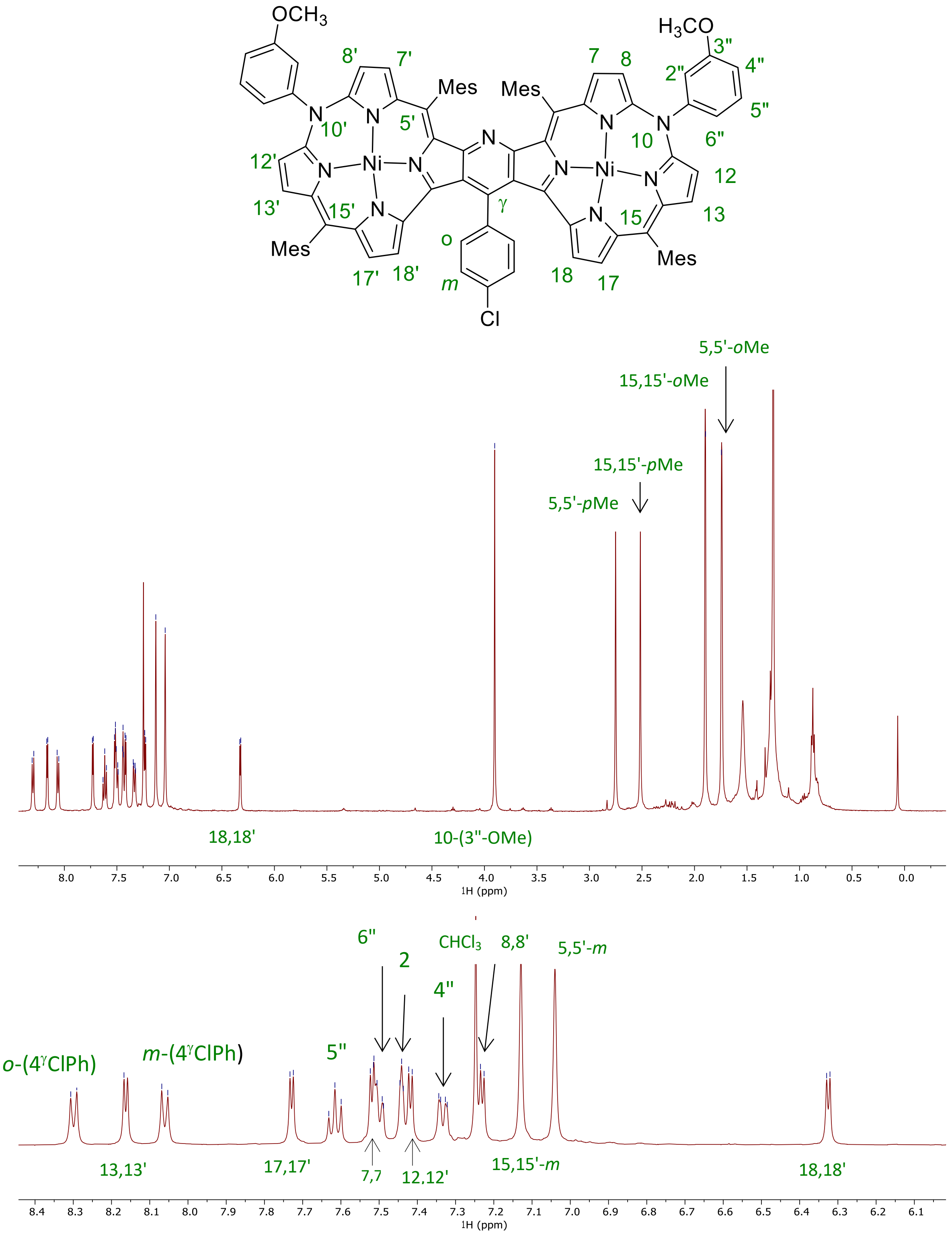

Figure S76. ^{1}H NMR signal assignments (500 MHz, $CDCl_3$, 300 K) for **5bb-2**.

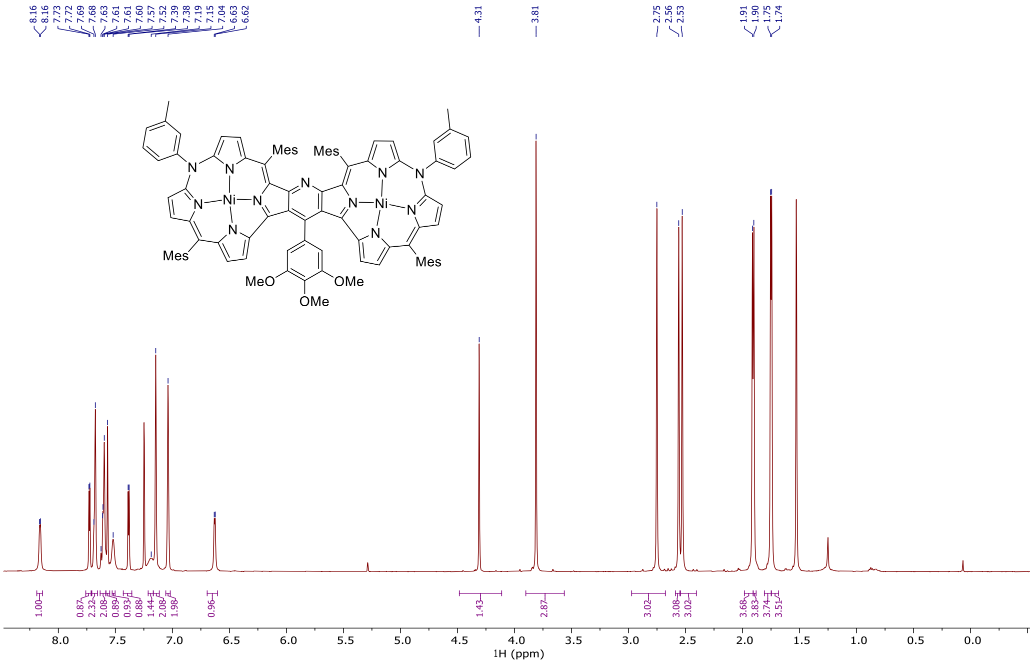

Figure S77. ^{1}H NMR spectrum (500 MHz, $CDCl_3$, 300 K) of **5bb-3**.

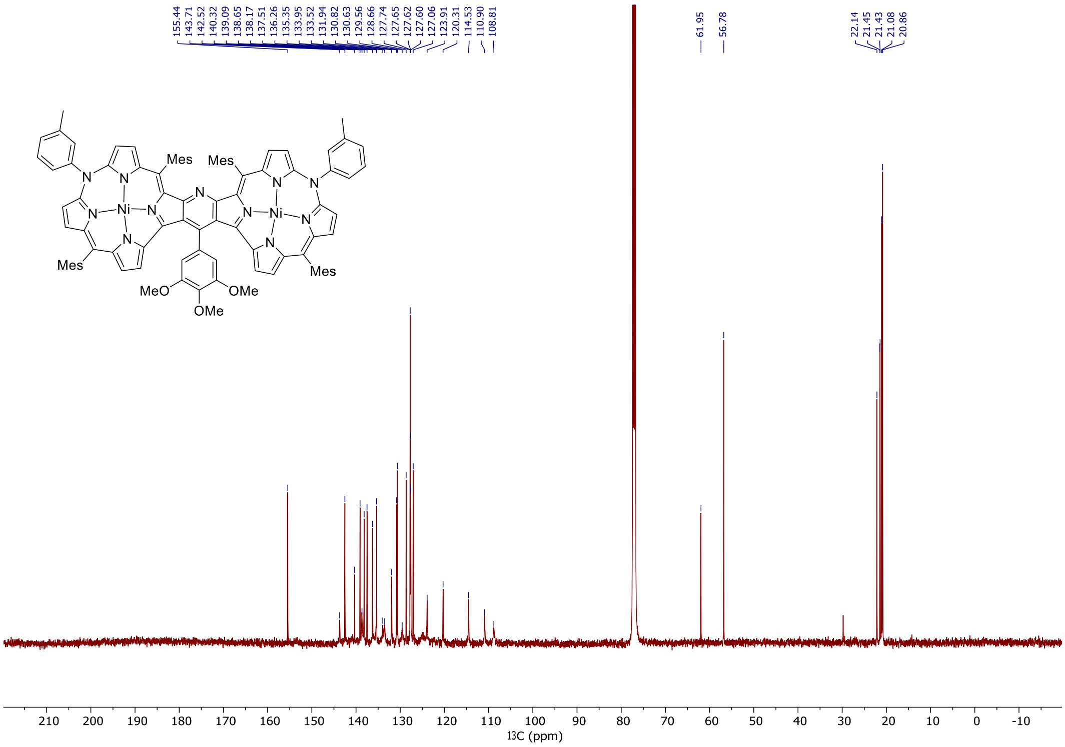

Figure S78. ^{13}C NMR spectrum (126 MHz, $CDCl_3$, 300 K) of **5bb-3**.

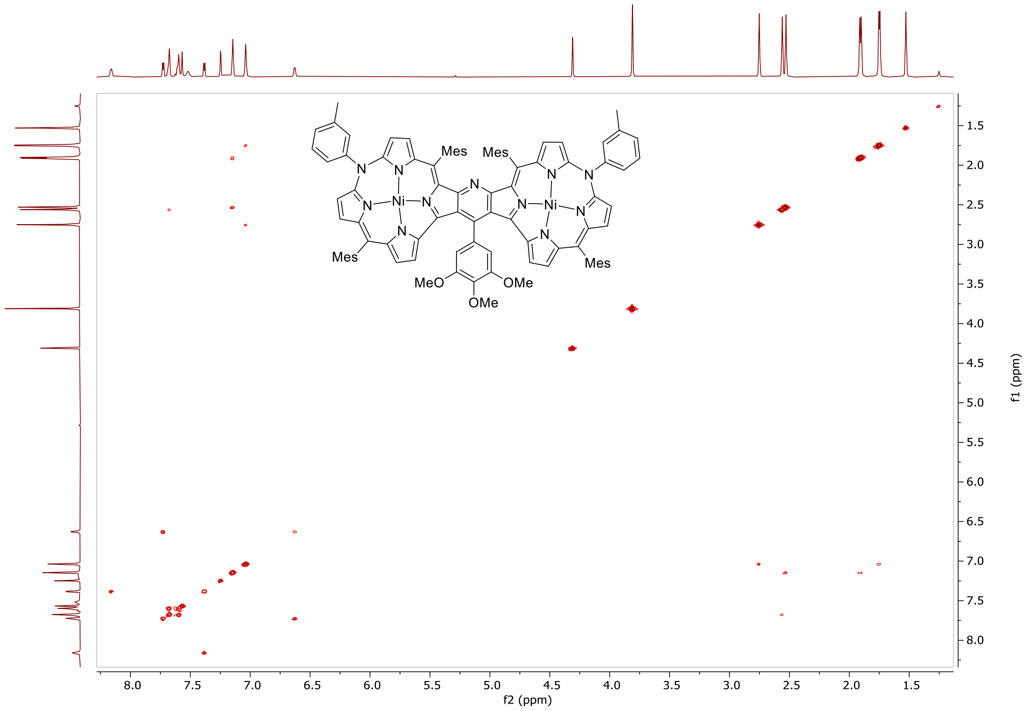

Figure S79. $^{1}H,^{1}H$ COSY spectrum (500 MHz, $CDCl_3$, 300 K) of **5bb-3**.

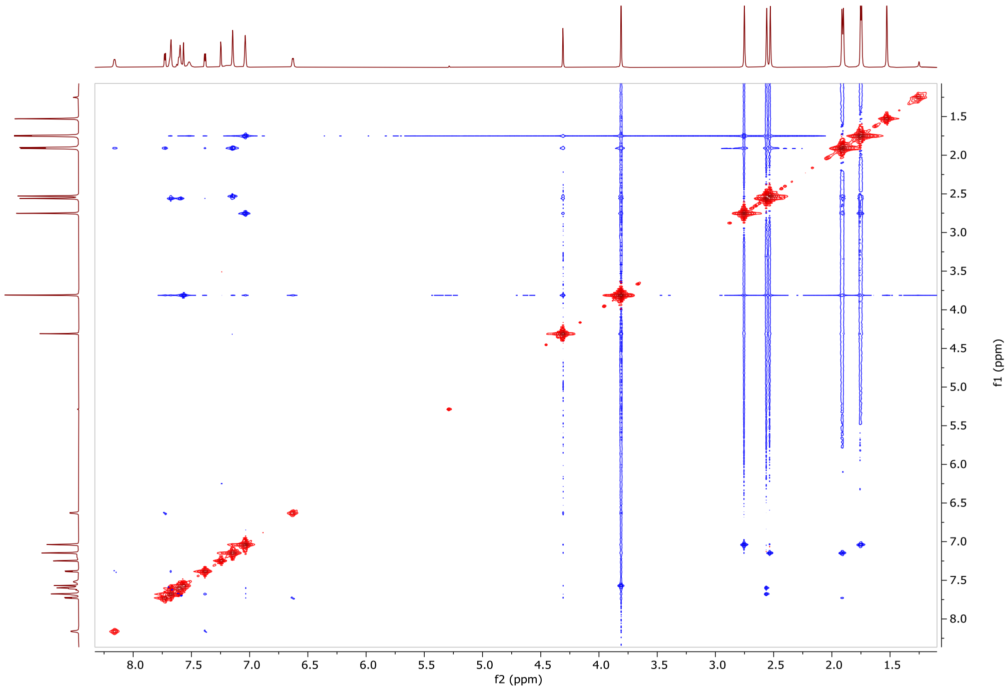

Figure S80. $^{1}H,^{1}H$ ROESY spectrum (500 MHz, $CDCl_3$, 300 K) of **5bb-3**.

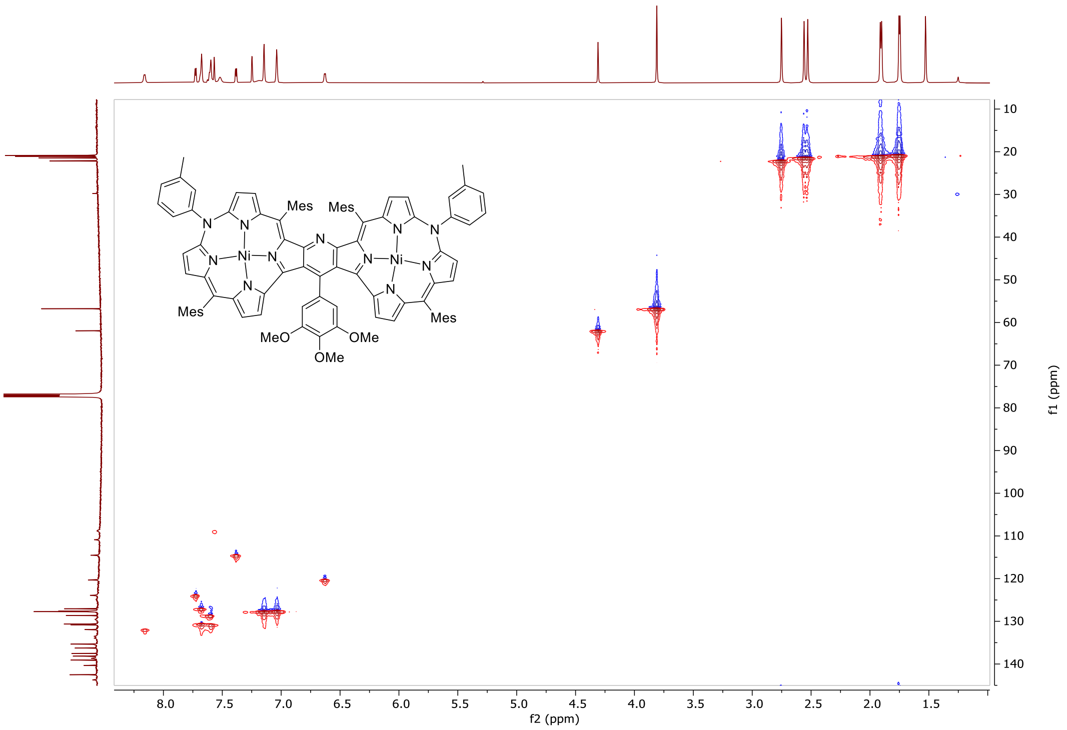

Figure S81. $^{1}H,^{13}C$ HSQC spectrum (500/126 MHz, $CDCl_3$, 300 K) of **5bb-3**.

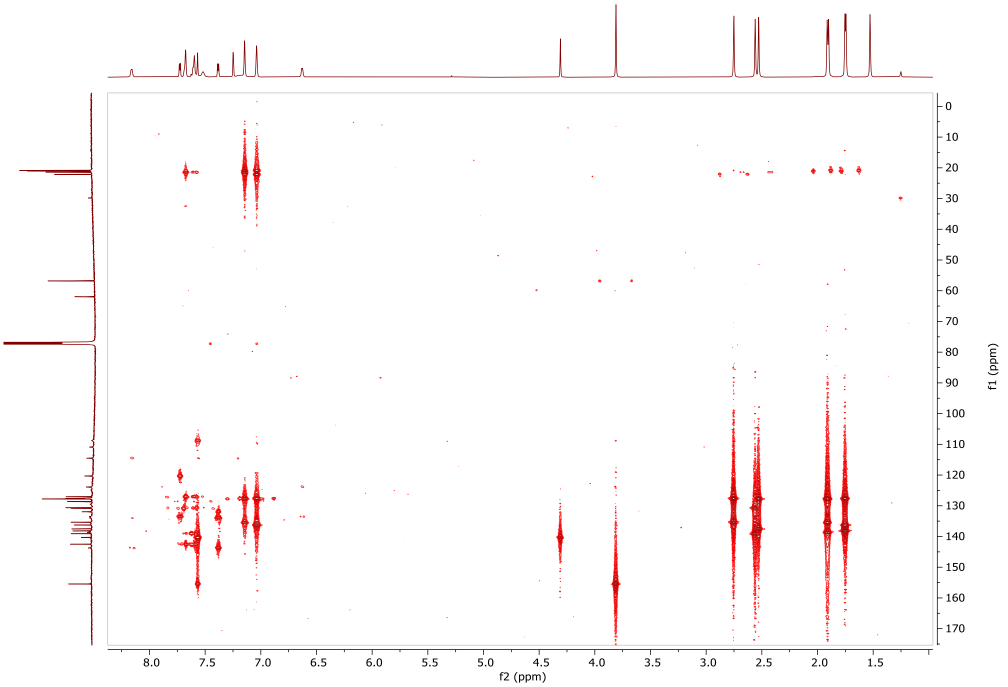

Figure S82. $^{1}H,^{13}C$ HMBC spectrum (500/126 MHz, $CDCl_3$, 300 K) of **5bb-3**.

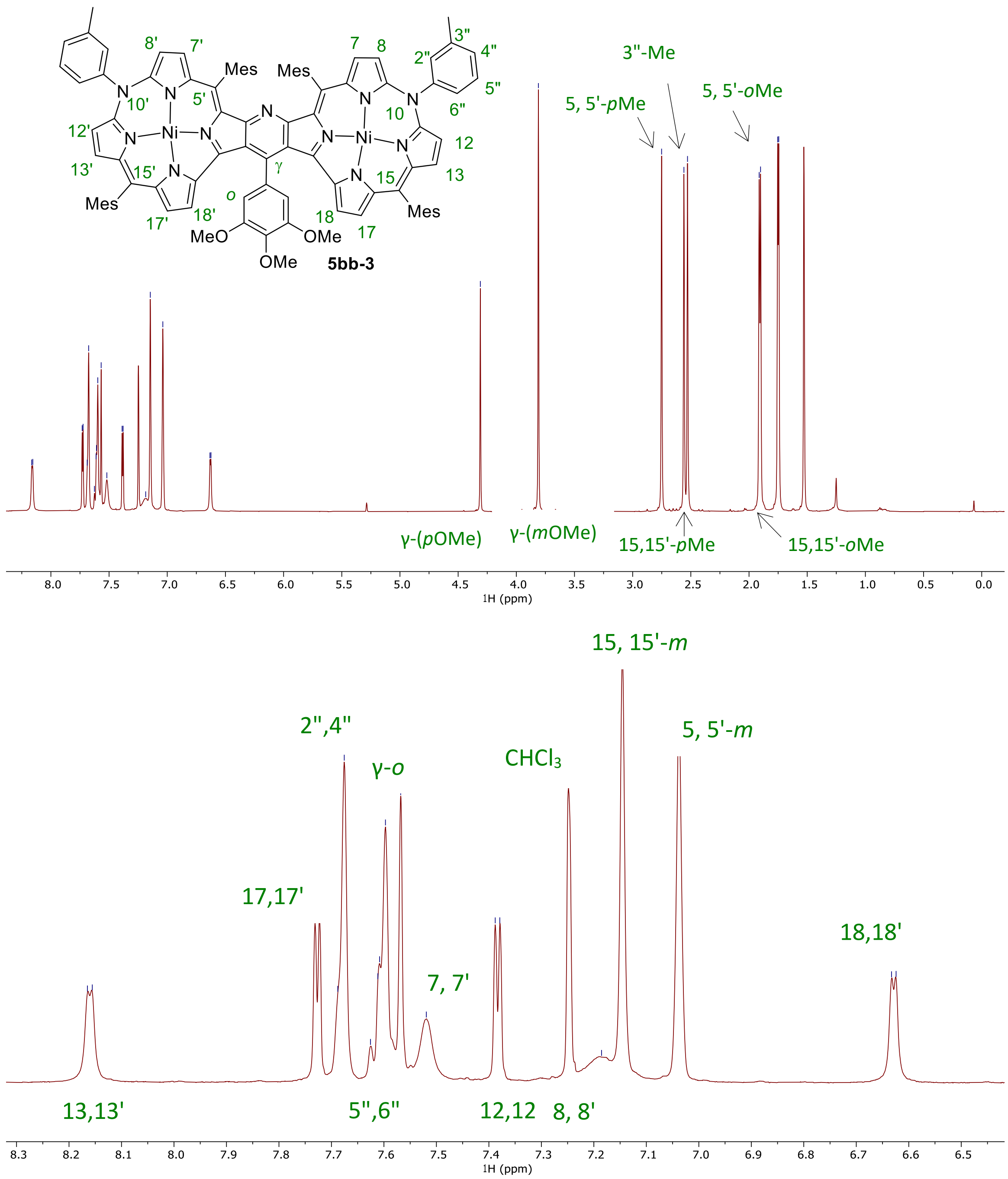

Figure S83. ^{1}H NMR signal assignments (500 MHz, $CDCl_3$, 300 K) for **5bb-3**.

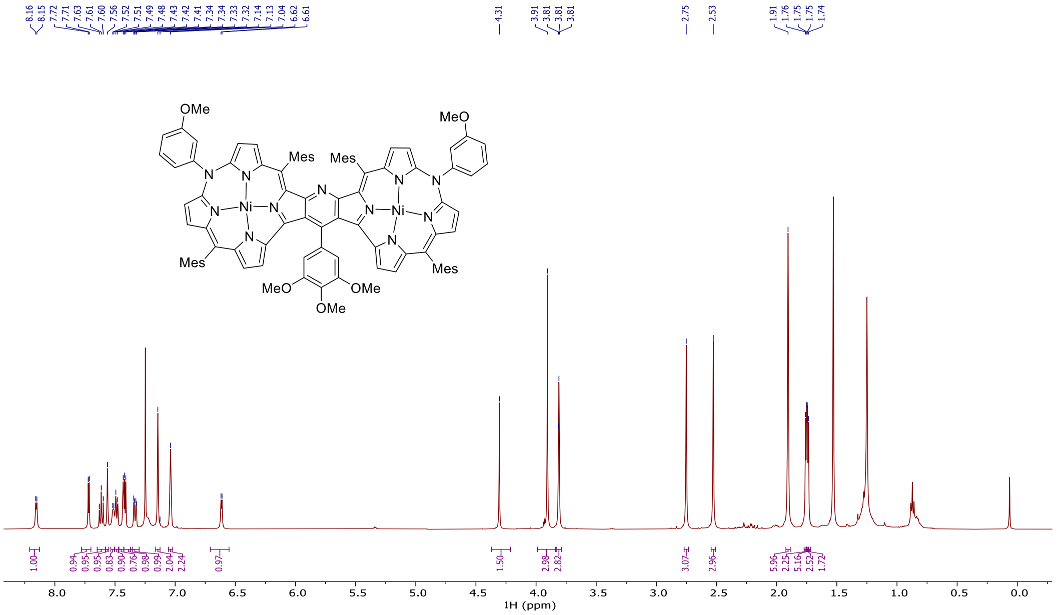

Figure S84. ^{1}H NMR spectrum (500 MHz, $CDCl_3$, 300 K) of **5bb-4**.

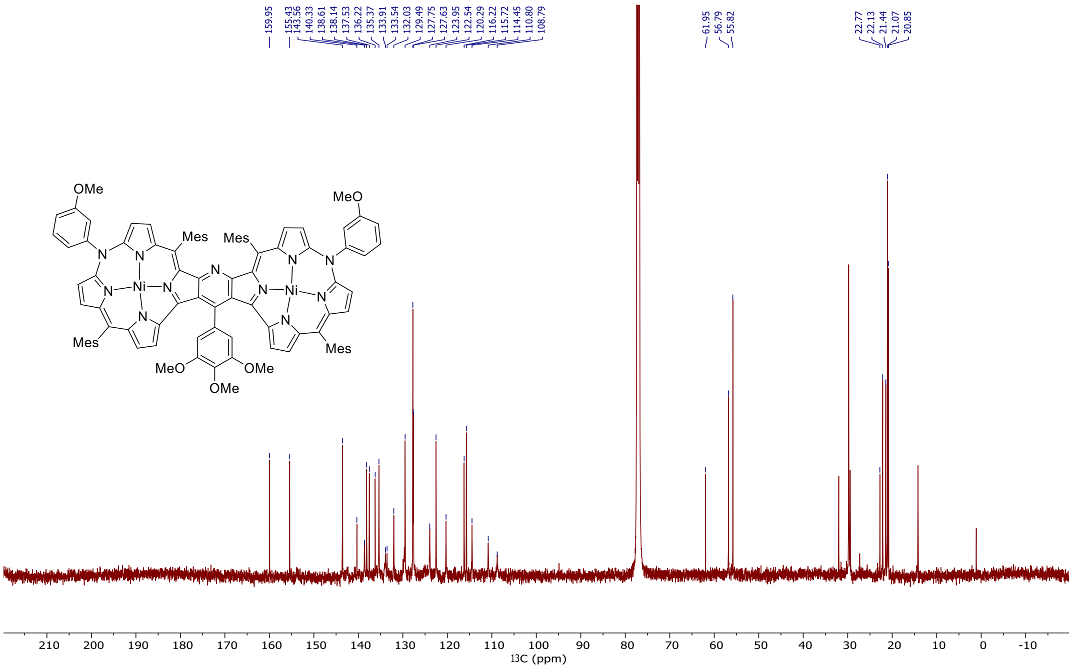

Figure S85. ^{13}C NMR spectrum (126 MHz, $CDCl_3$, 300 K) of **5bb-4**.

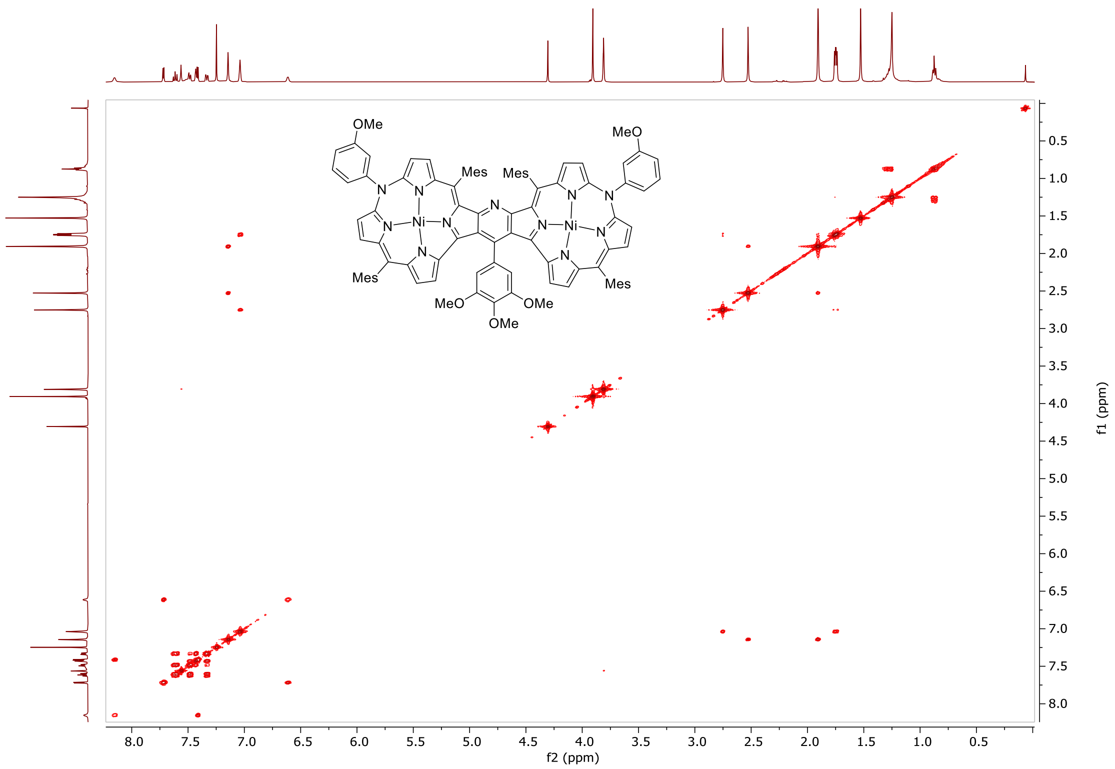

Figure S86. $^{1}H,^{1}H$ COSY spectrum (500 MHz, $CDCl_3$, 300 K) of **5bb-4**.

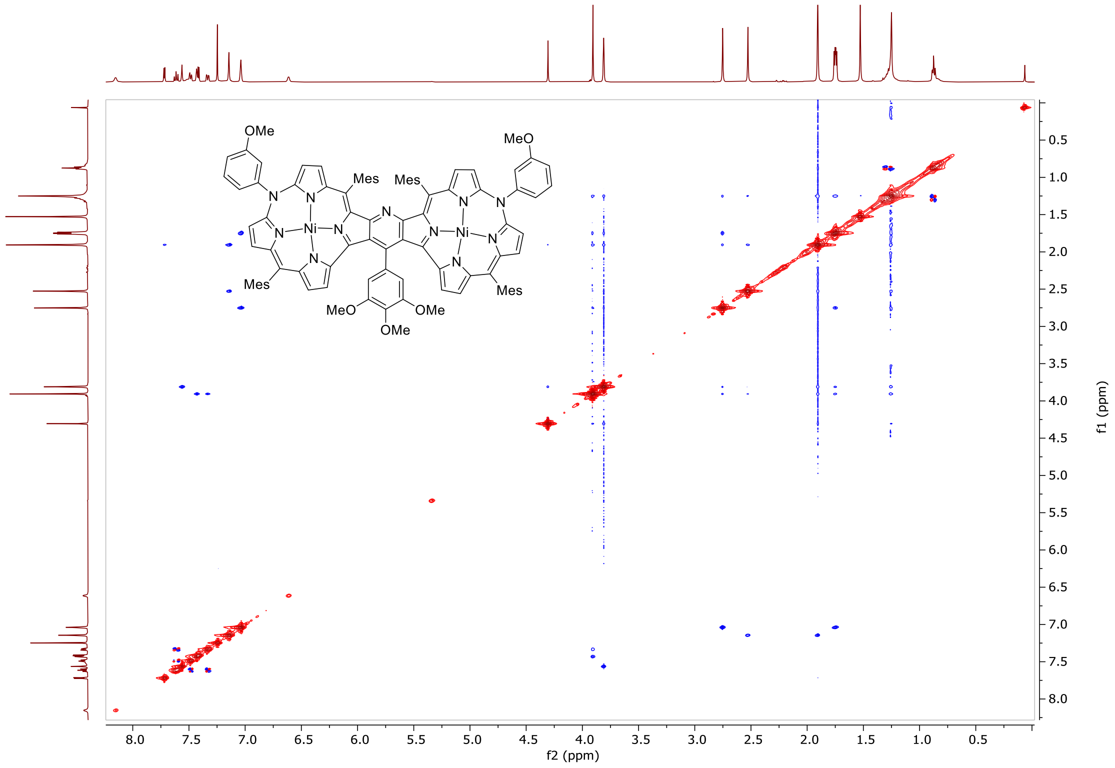

Figure S87. $^{1}H,^{1}H$ ROESY spectrum (500 MHz, $CDCl_3$, 300 K) of **5bb-4**.

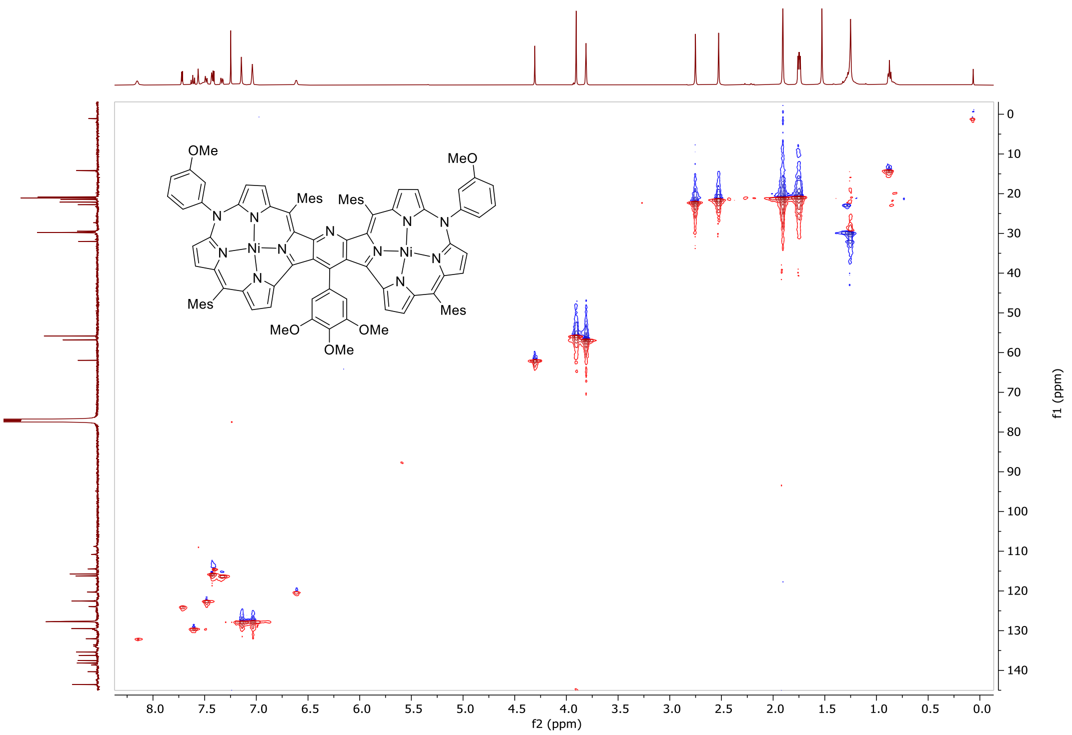

Figure S88. $^{1}H,^{13}C$ HSQC spectrum (500/126 MHz, $CDCl_3$, 300 K) of **5bb-4**.

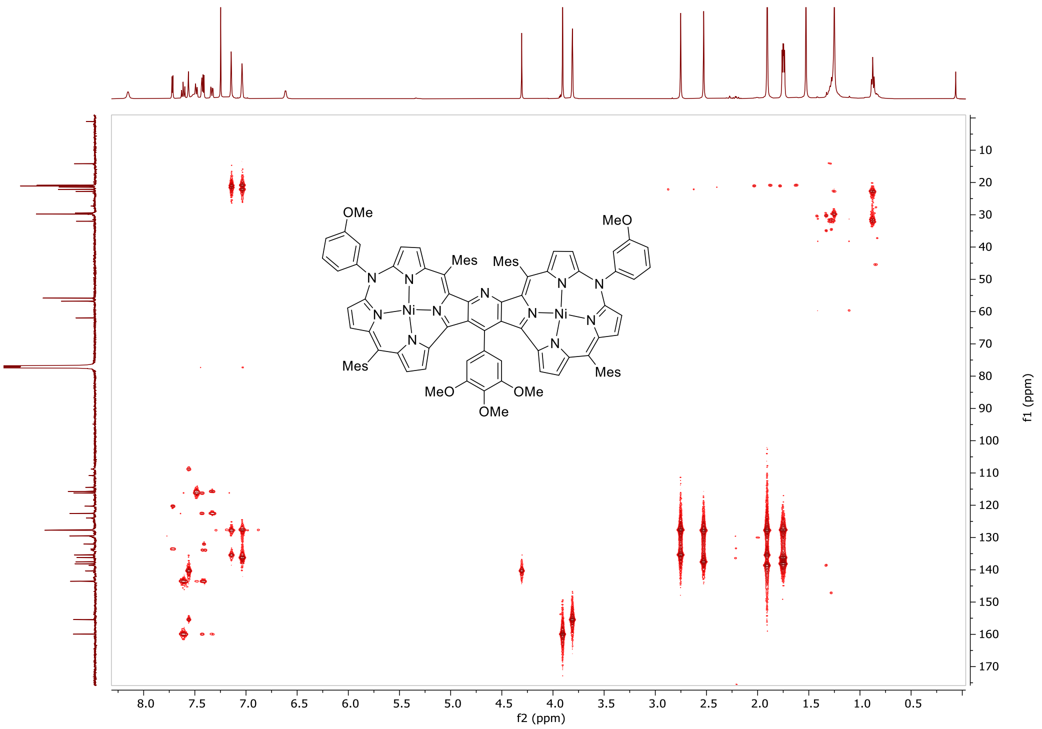

Figure S89. $^{1}H,^{13}C$ HMBC spectrum (500/126 MHz, $CDCl_3$, 300 K) of **5bb-4**.

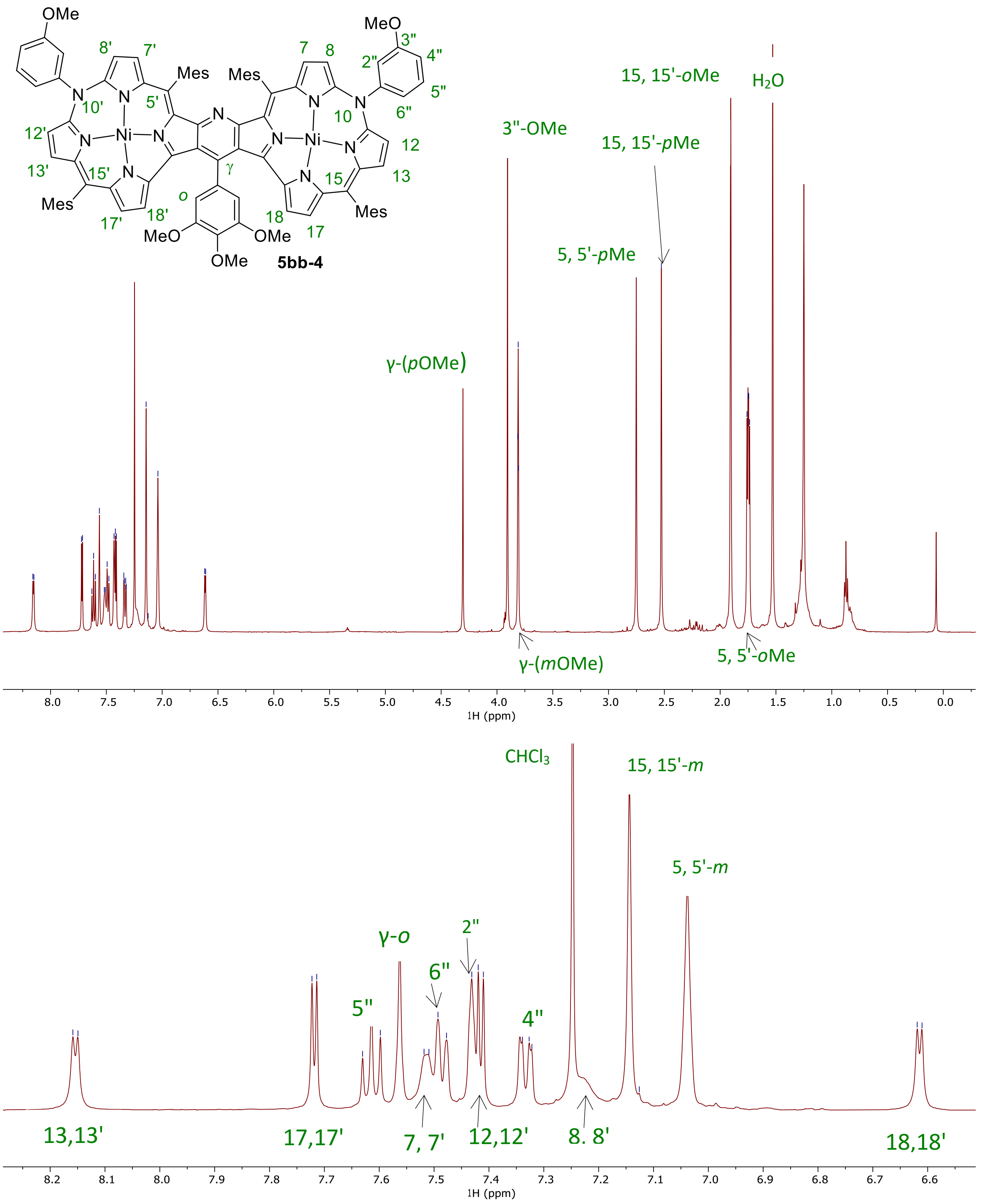

Figure S90. ^{1}H NMR signal assignments (500 MHz, $CDCl_3$, 300 K) for **5bb-4**.

11. Mass spectra for **5bb** and precursors

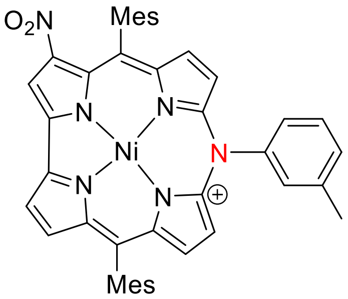

Chemical Formula:
$C_{43}H_{36}N_6NiO_2^+$
Exact Mass: 726.2248

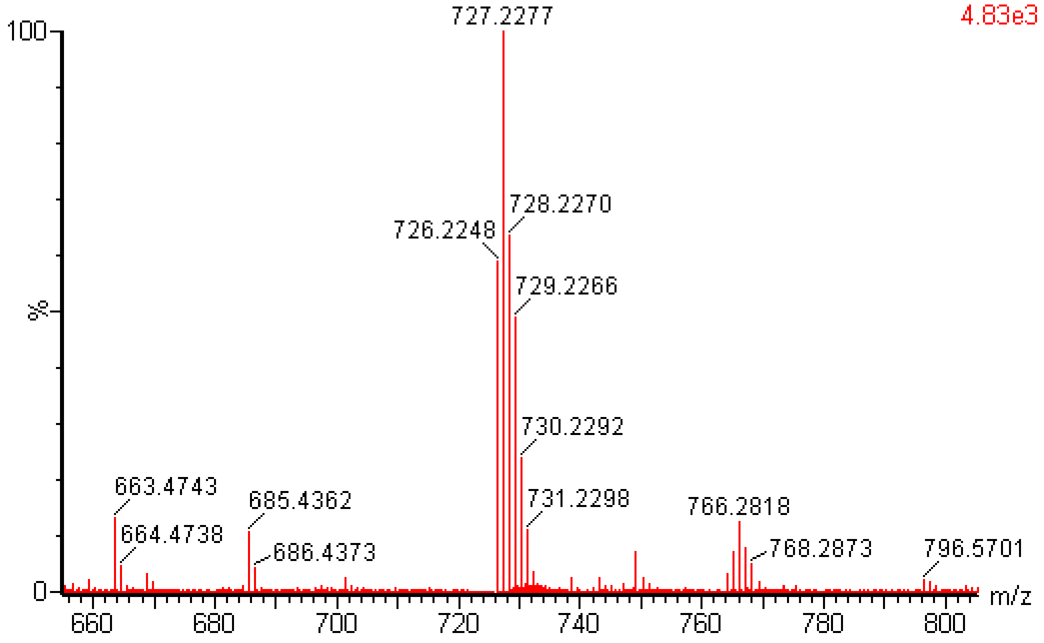

Figure S91. ESI(+) HRMS spectrum of **2-NO₂**(3-MePh).

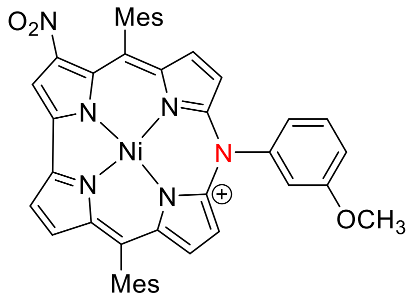

Chemical Formula:
$C_{43}H_{36}N_6NiO_3^+$
Exact Mass: 742.2197

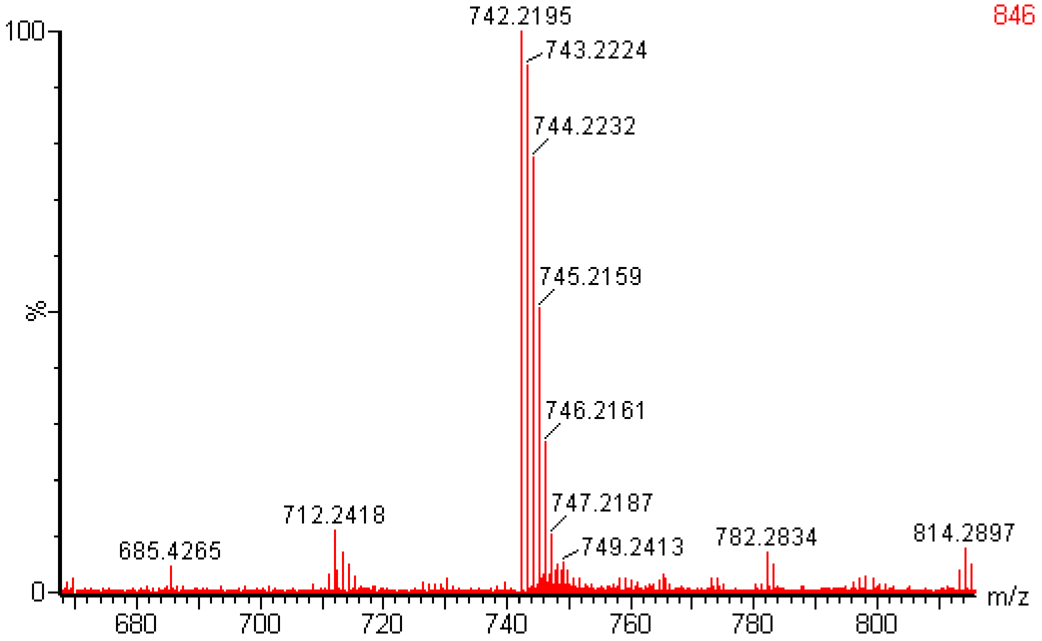

Figure S92. ESI(+) HRMS spectrum of **2-NO$_2$**(3-MeOPh).

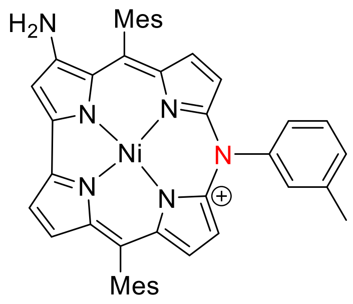

Chemical Formula: $C_{43}H_{38}N_6Ni^+$
Exact Mass: 696.2506

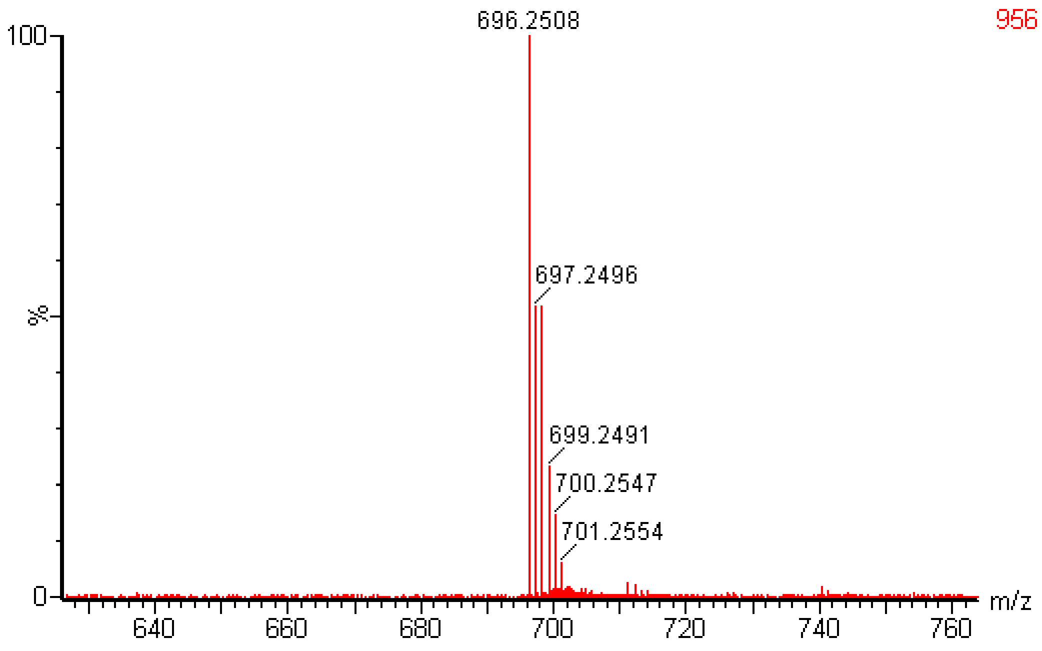

Figure S93. ESI(+) HRMS spectrum of **2-NH$_2$**(3-MePh).

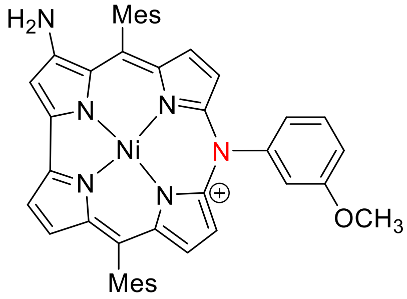

Chemical Formula: $C_{43}H_{38}N_6NiO^+$
Exact Mass: 712.2455

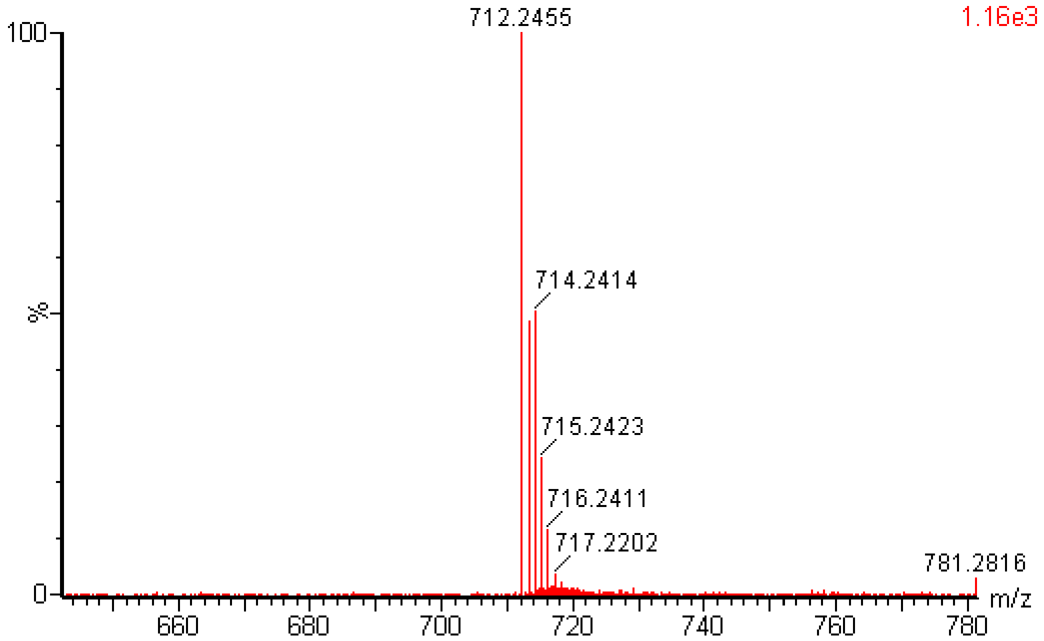

Figure S94. ESI(+) HRMS spectrum of **2-NH$_2$**(3-MeOPh).

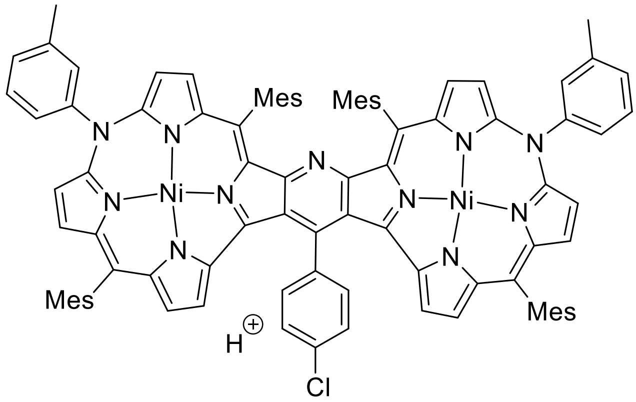

Chemical Formula: $C_{93}H_{75}ClN_{11}Ni_2^+$
Exact Mass: 1496.4597

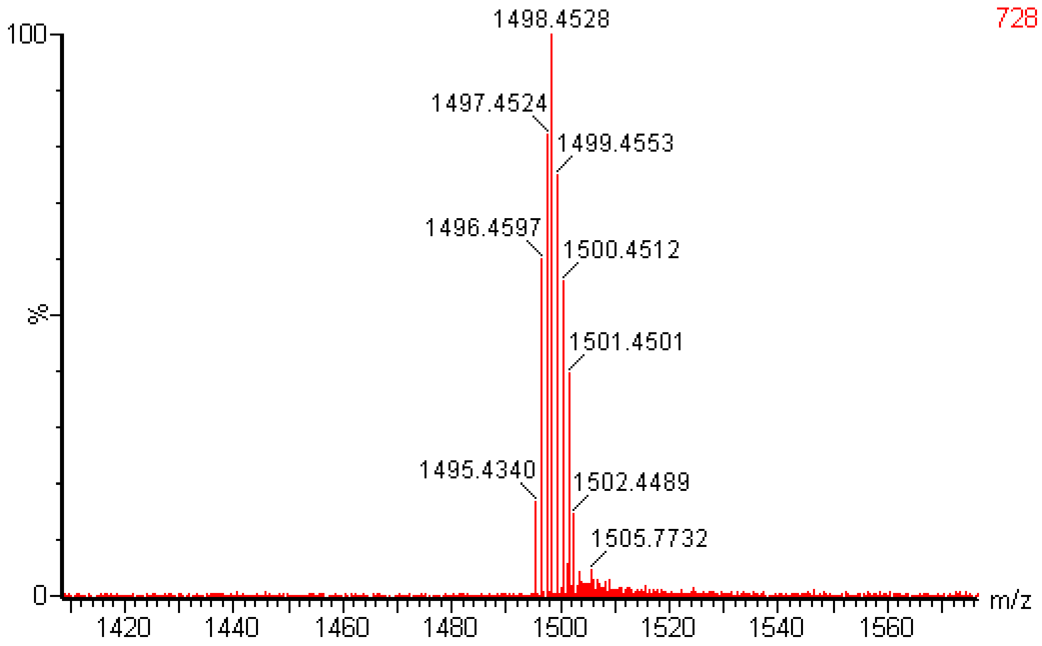

Figure S95. ESI(+) HRMS spectrum of **5bb-1**.

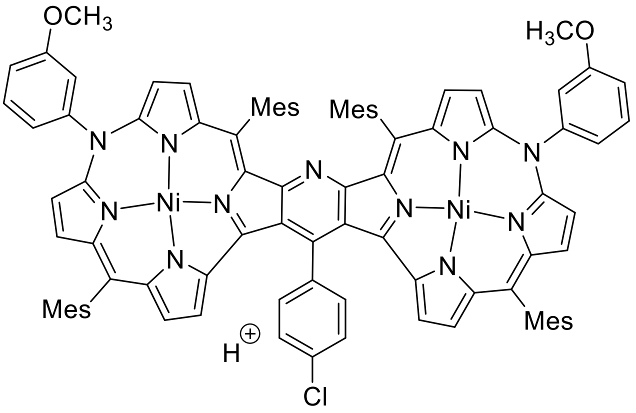

Chemical Formula: $C_{93}H_{75}ClN_{11}Ni_2O_2^+$
Exact Mass: 1528.4495

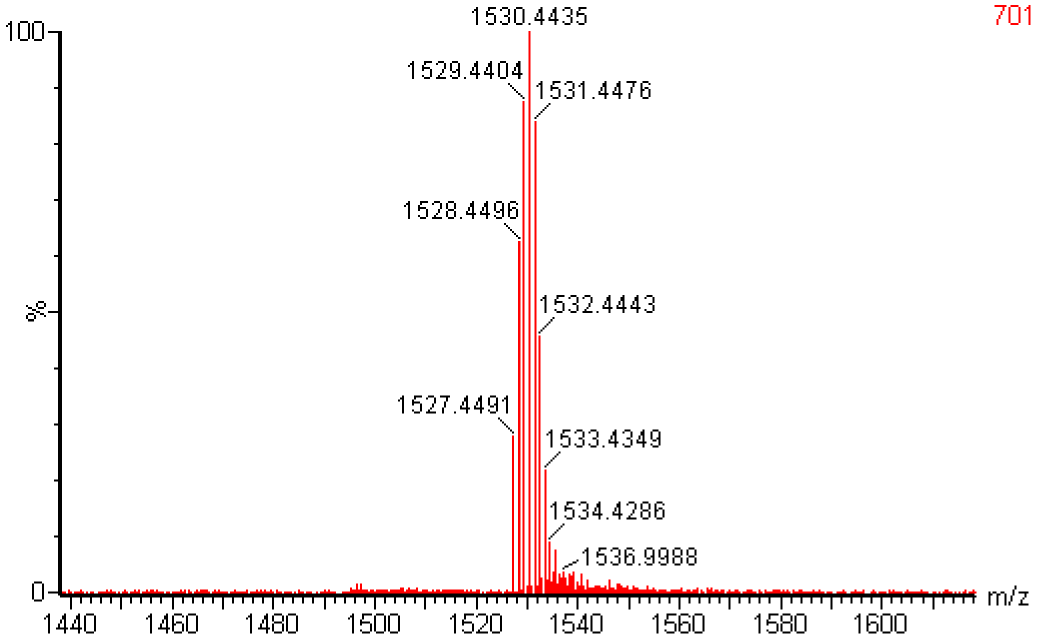

Figure S96. ESI(+) HRMS spectrum of **5bb-2**.

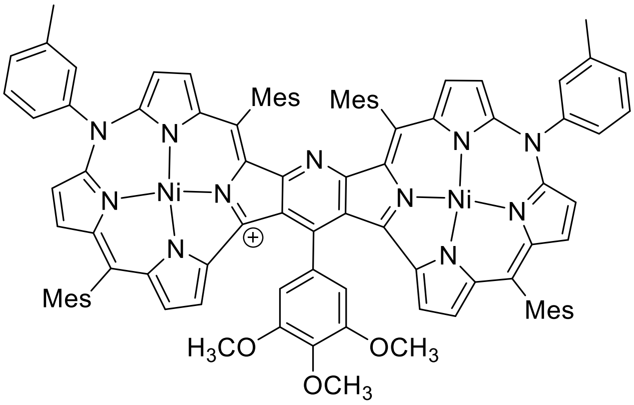

Chemical Formula: $C_{96}H_{81}N_{11}Ni_2O_3^+$
Exact Mass: 1551.5225

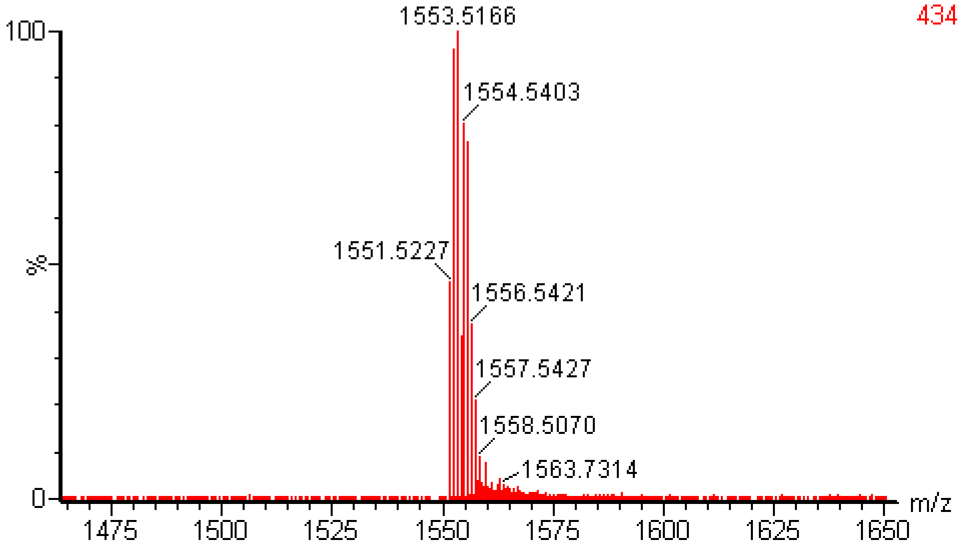

Figure S97. ESI(+) HRMS spectrum of **5bb-3**.

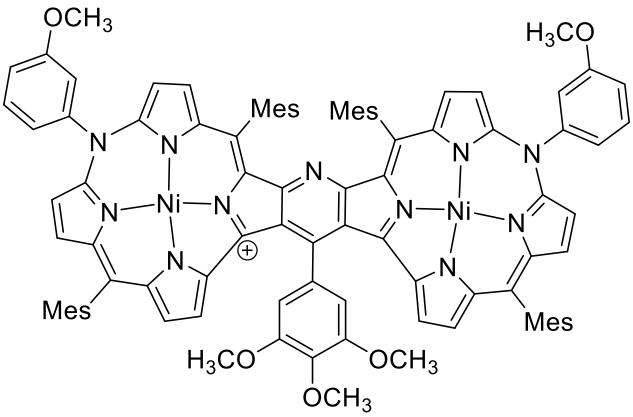

Chemical Formula: $C_{96}H_{81}N_{11}Ni_2O_5^+$
Exact Mass: 1583.5124

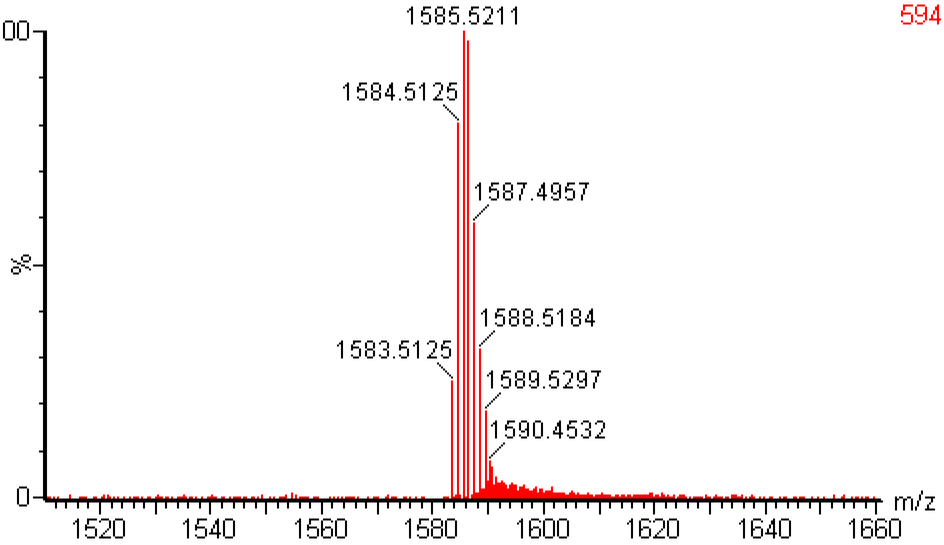

Figure S98. ESI(+) HRMS spectrum of **5bb-4**.

12. UV-vis spectra for **5bb** and precursors

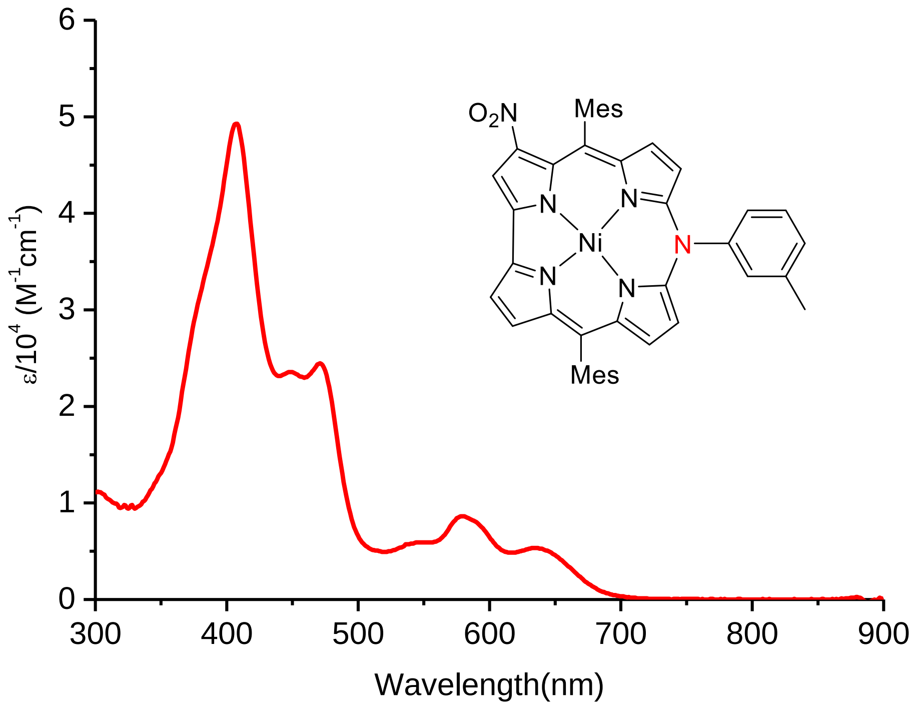

Figure S99. UV-vis spectrum of **2-NO$_2$**(3MePh) in dichloromethane.

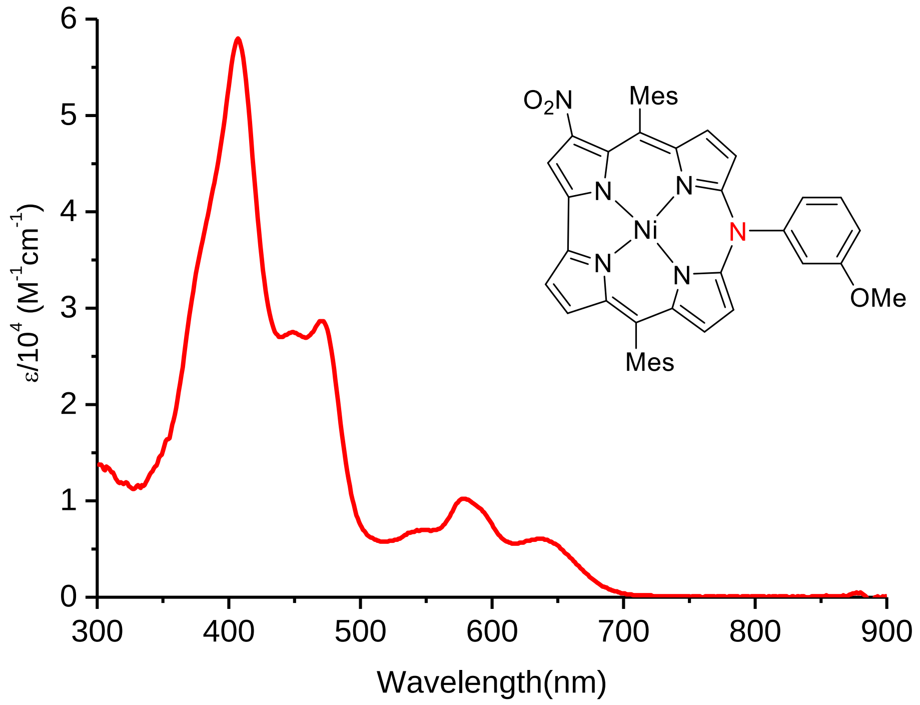

Figure S100. UV-vis spectrum of **2-NO$_2$**(3MeOPh) in dichloromethane.

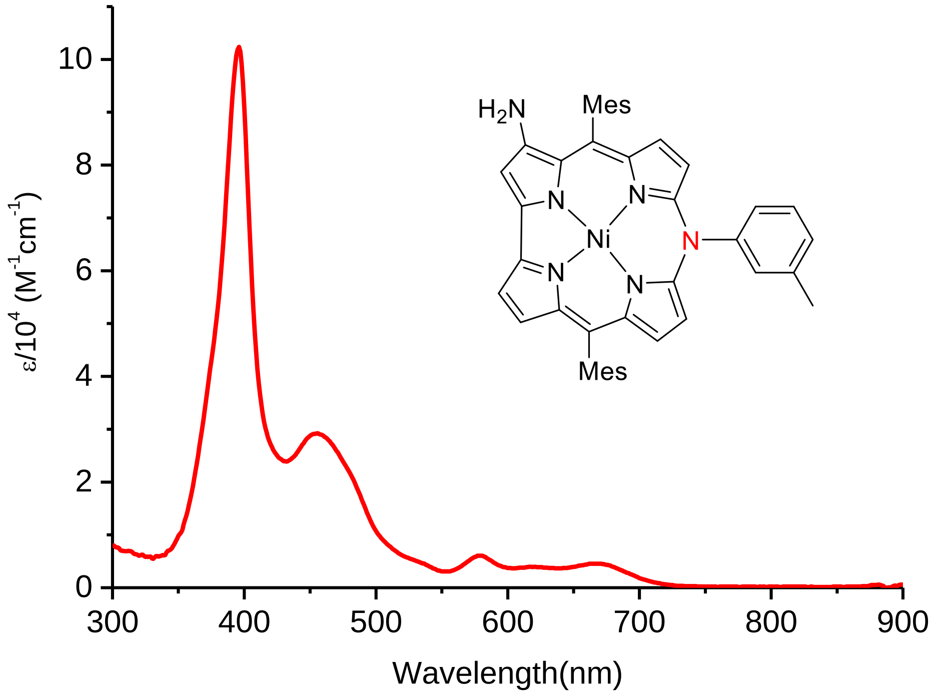

Figure S101. UV-vis spectrum of **2-NH$_2$**(3MePh) in dichloromethane.

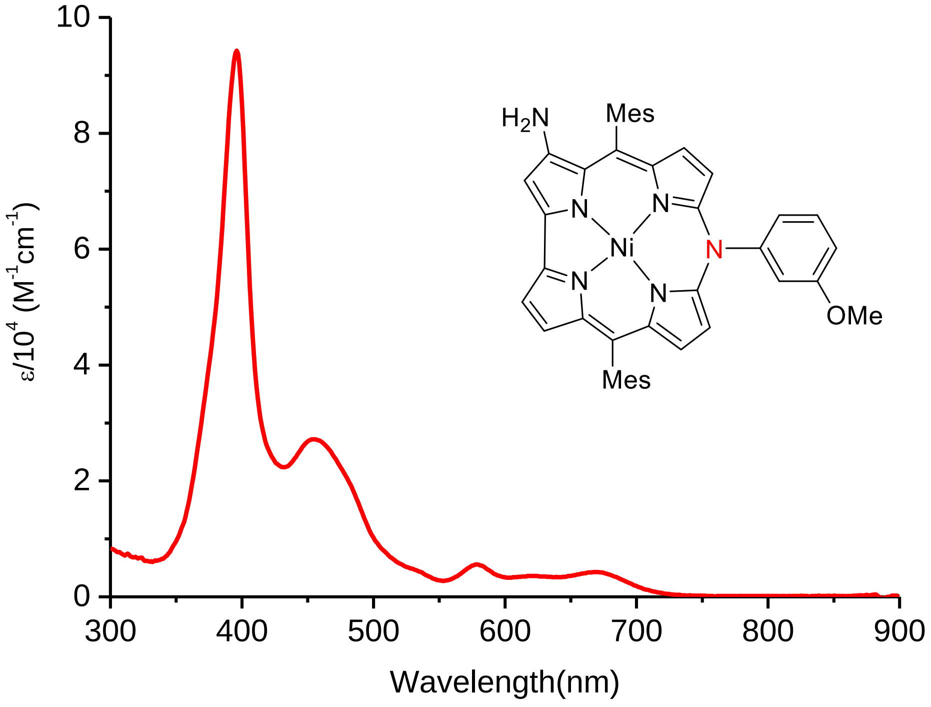

Figure S102. UV-vis spectrum of **2-NH$_2$**(3MeOPh) in dichloromethane.

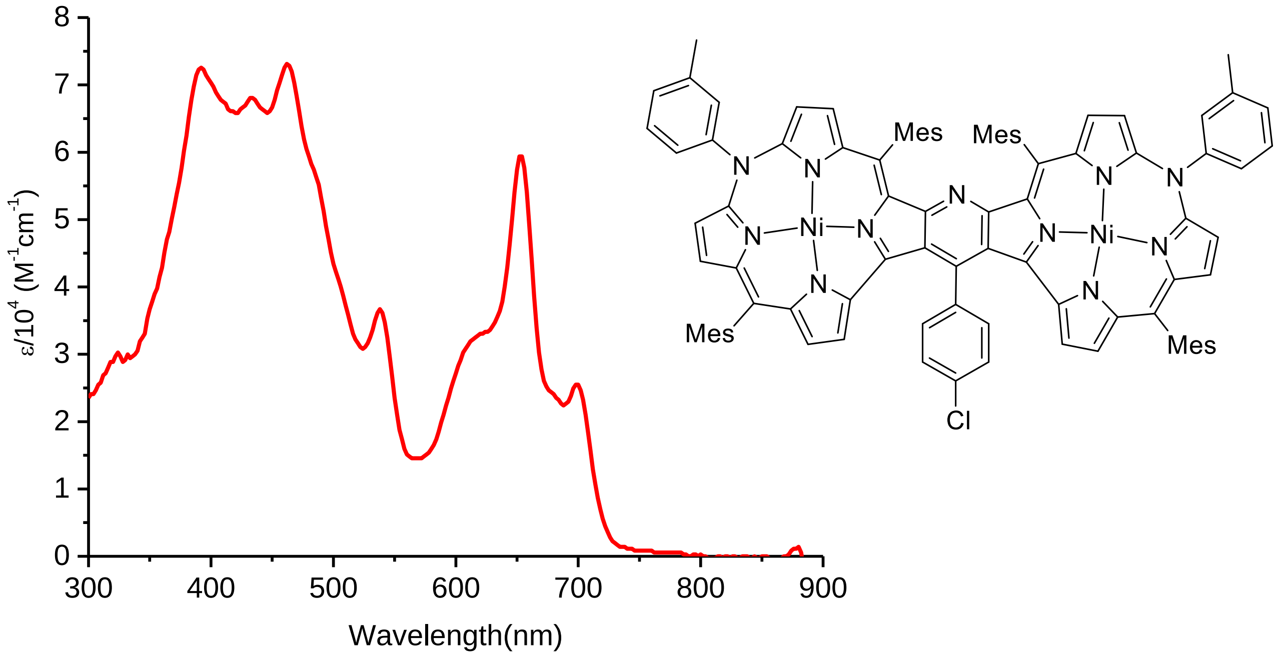

Figure S103. UV-vis spectrum of **5bb-1** in dichloromethane.

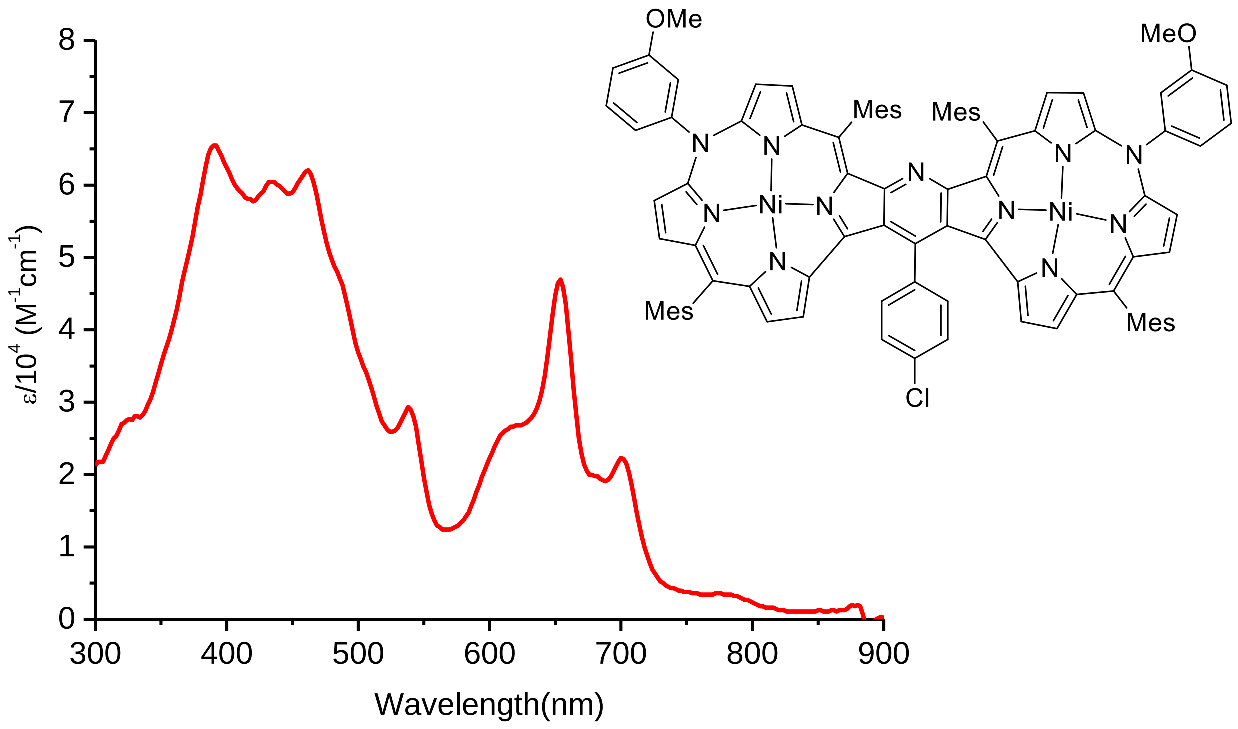

Figure S104. UV-vis spectrum of **5bb-2** in dichloromethane.

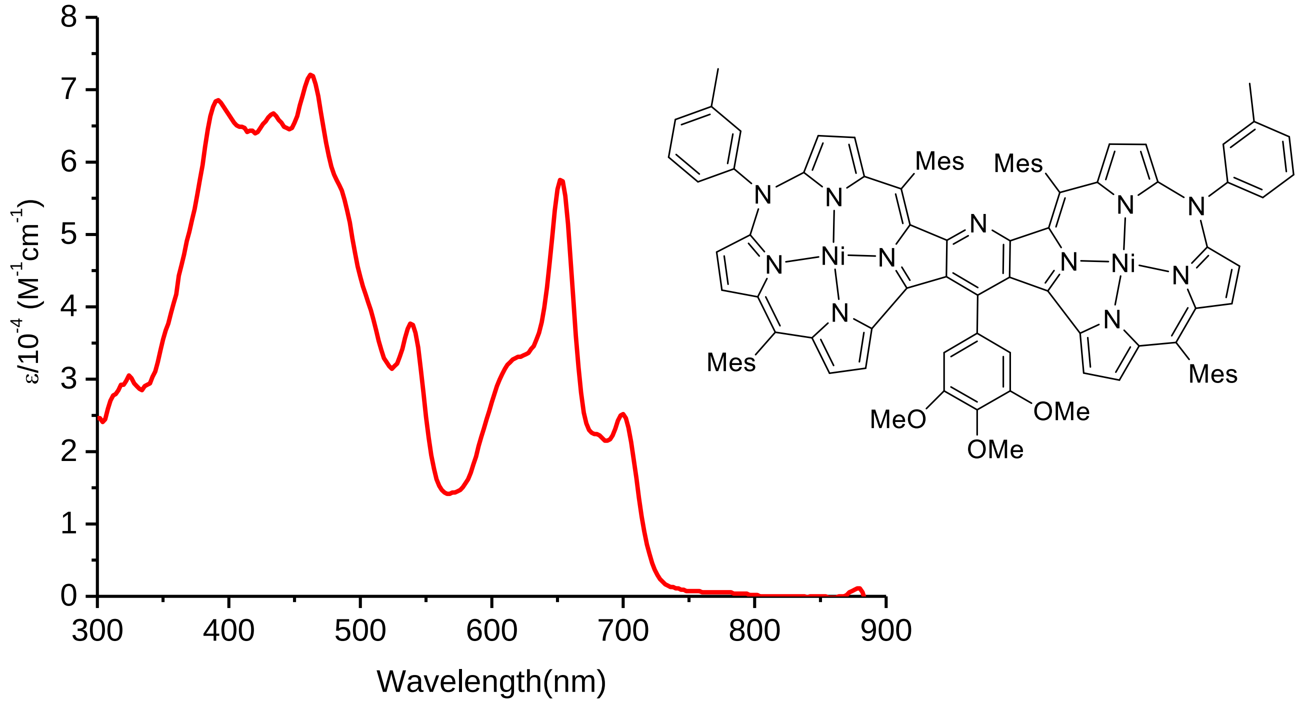

Figure S105. UV-vis spectrum of **5bb-3** in dichloromethane.

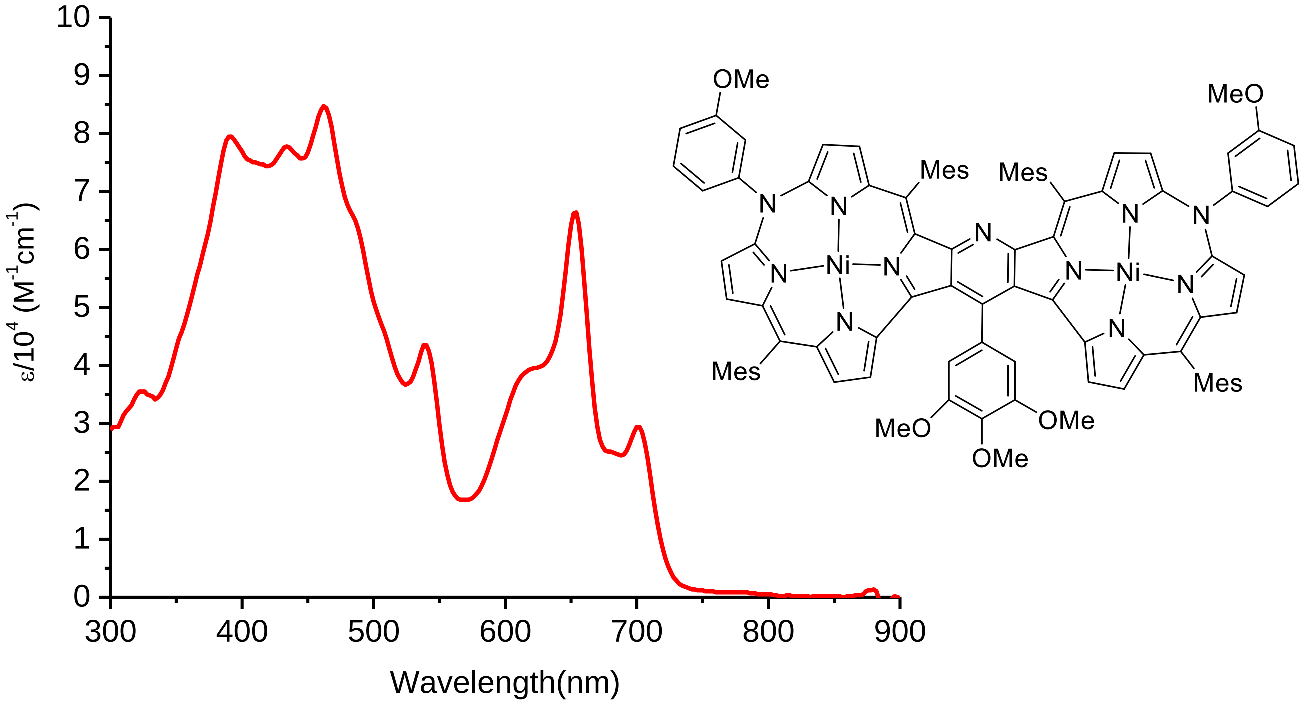

Figure S106. UV-vis spectrum of **5bb-4** in dichloromethane.

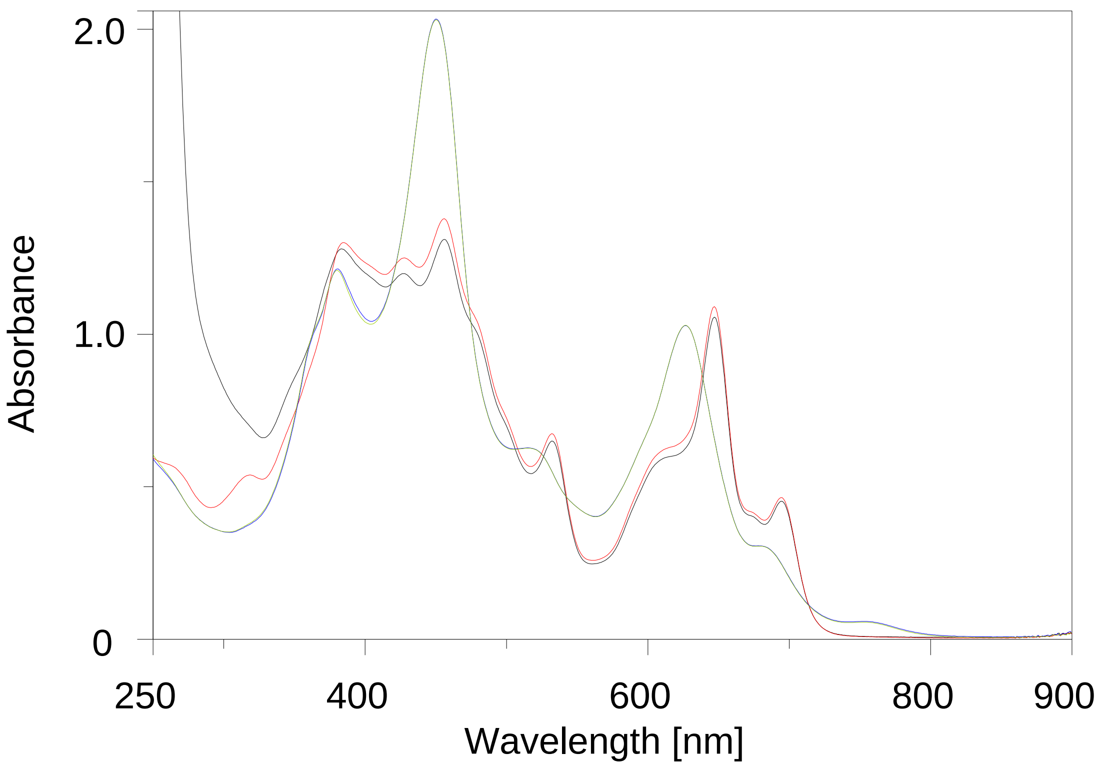

Figure S107. UV-vis spectrum of **5bb-2** in dichloromethane (red), after addition 1 equiv. (blue) and 25 equiv. (green) of trifluoroacetic acid (TFA). The black trace is a spectrum of the sample after addition of 30 equiv. of *N*-ethyl-*N,N*-diisopropylamine to the sample acidified with 25 equiv. of TFA.

13. Electrochemical data

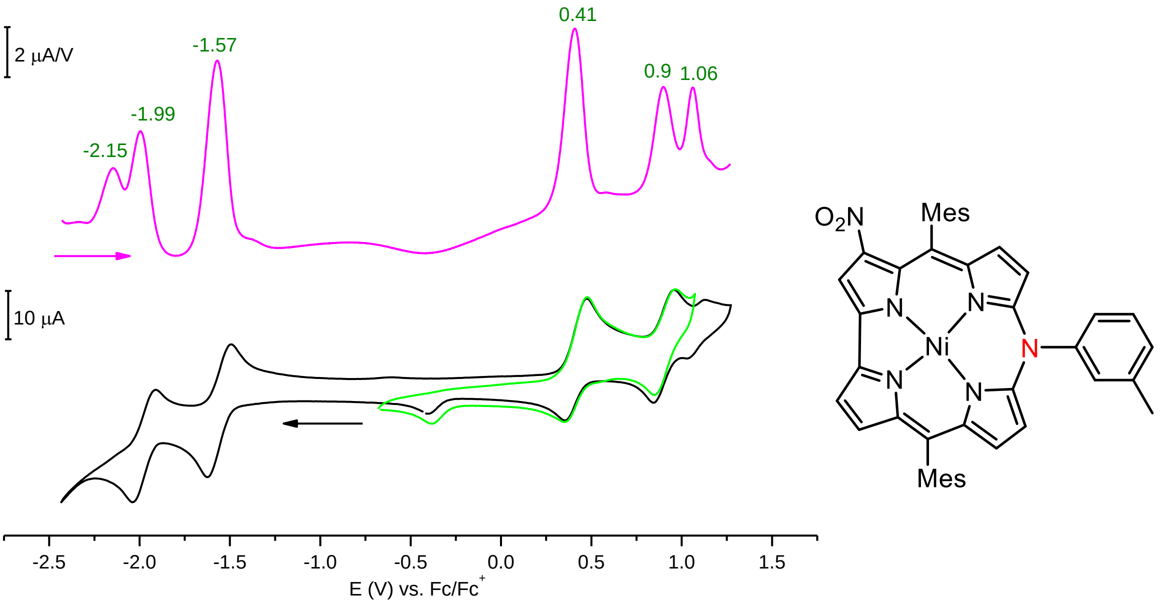

Figure S108. Differential pulse (DP) and cyclic (CV) voltammograms recorded for **2-NO$_2$**(3MePh) in DCM with [Bu$_4$N]PF$_6$ as supporting electrolyte.

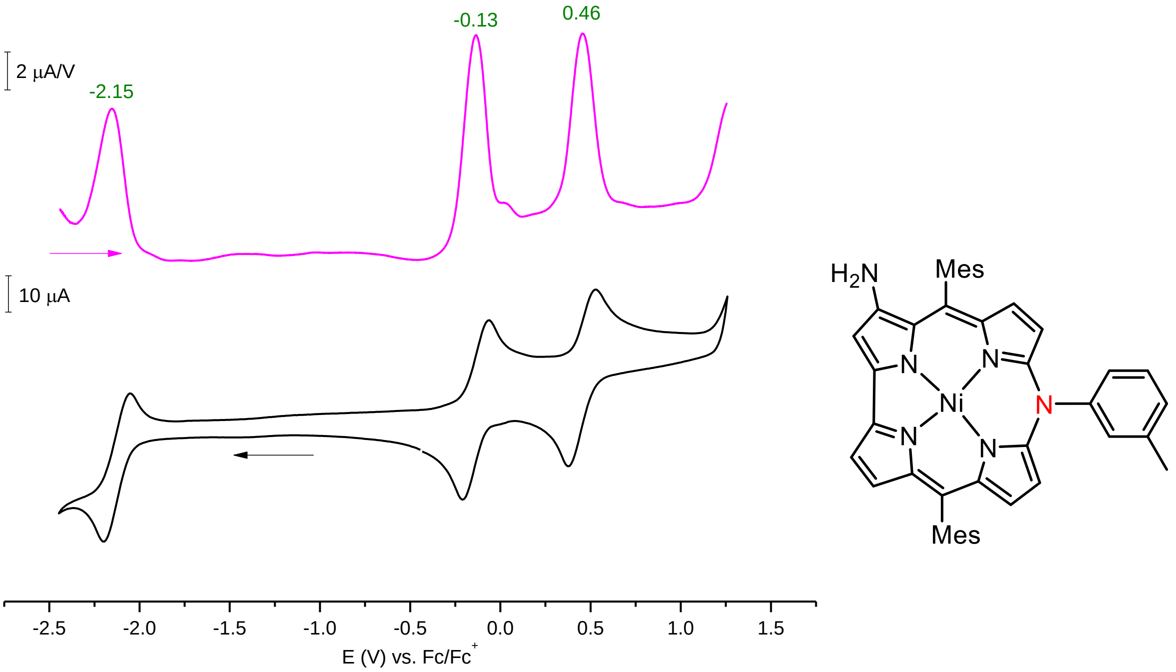

Figure S109. Differential pulse (DP) and cyclic (CV) voltammograms recorded for **2-NH$_2$**(3MePh) in DCM with [Bu$_4$N]PF$_6$ as supporting electrolyte.

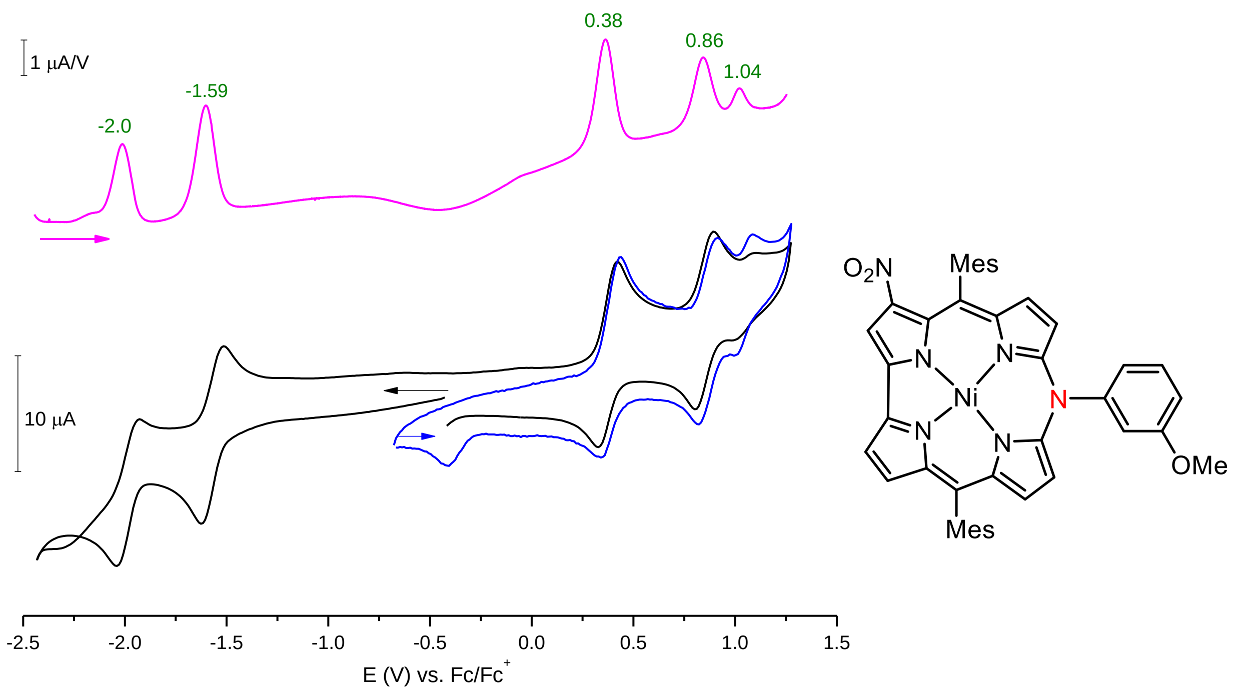

Figure S110. Differential pulse (DP) and cyclic (CV) voltammograms recorded for **2-NO₂**(3MeOPh) in DCM with $[Bu_4N]PF_6$ as supporting electrolyte.

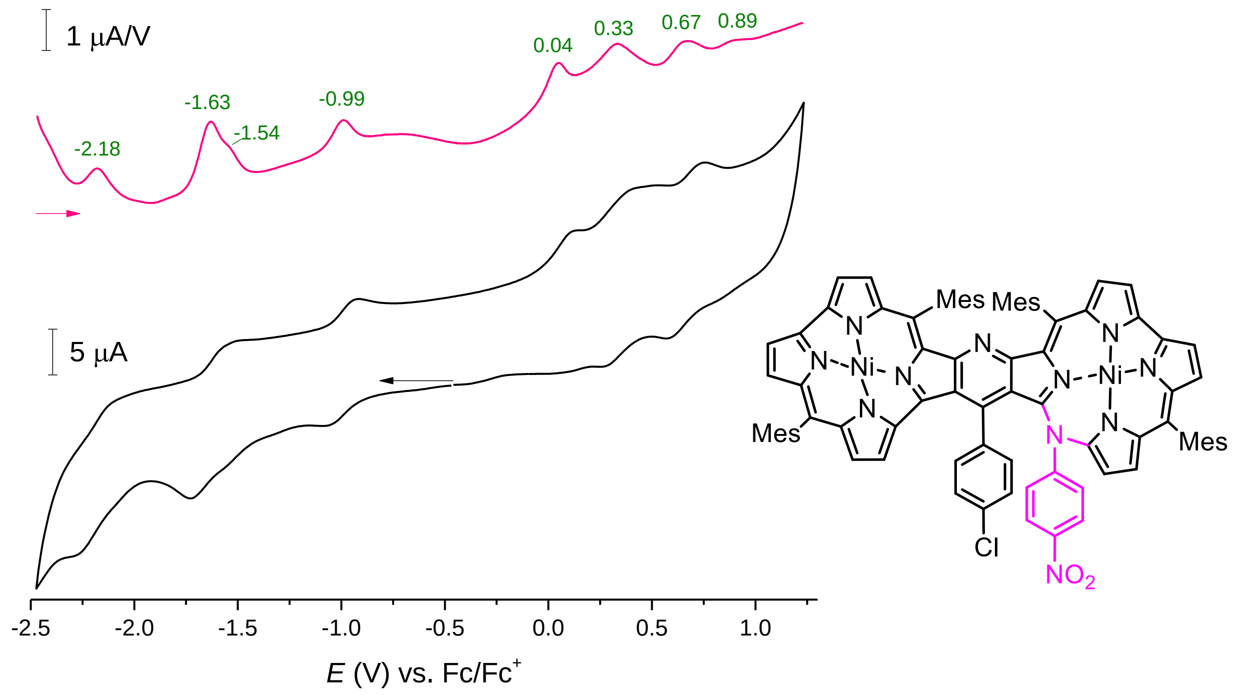

Figure S111. Differential pulse (DP) and cyclic (CV) voltammograms recorded for **4a** in DCM with $[Bu_4N]PF_6$ as supporting electrolyte.

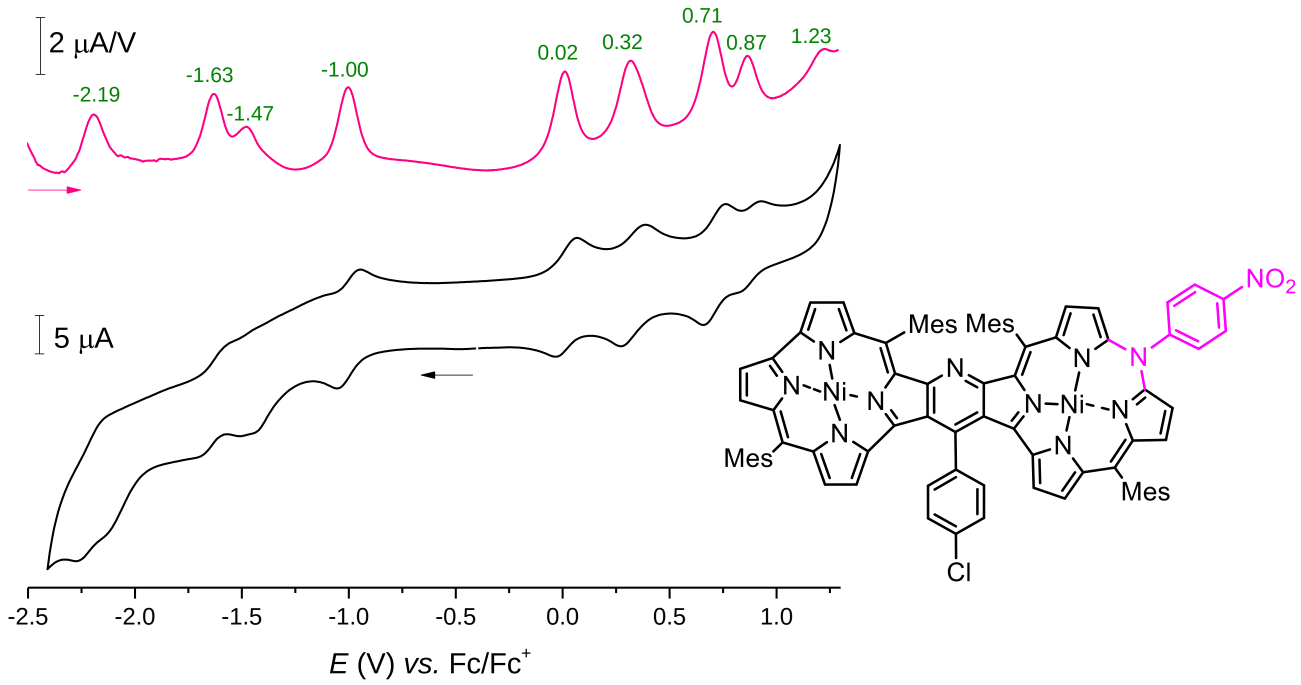

Figure S112. Differential pulse (DP) and cyclic (CV) voltammograms recorded for **4b** in DCM with $[Bu_4N]PF_6$ as supporting electrolyte.

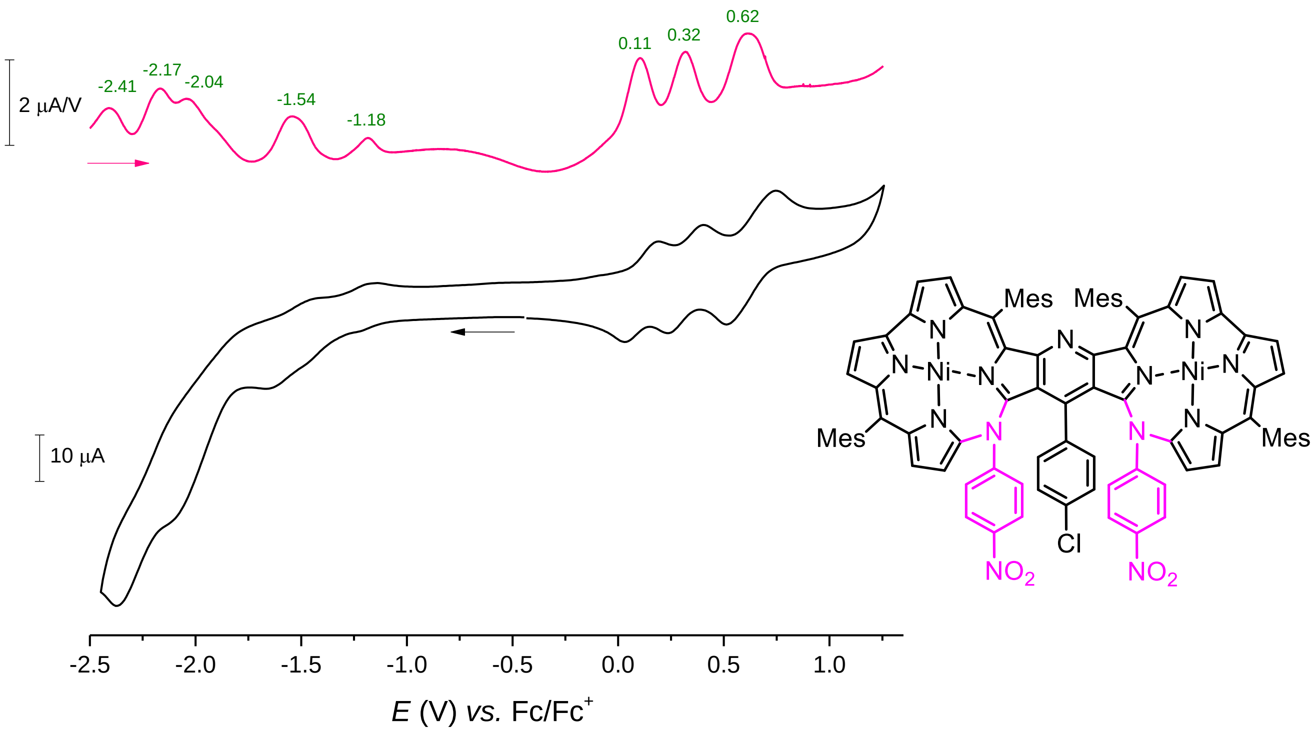

Figure S113. Differential pulse (DP) and cyclic (CV) voltammograms recorded for **5aa** in DCM with $[Bu_4N]PF_6$ as supporting electrolyte.

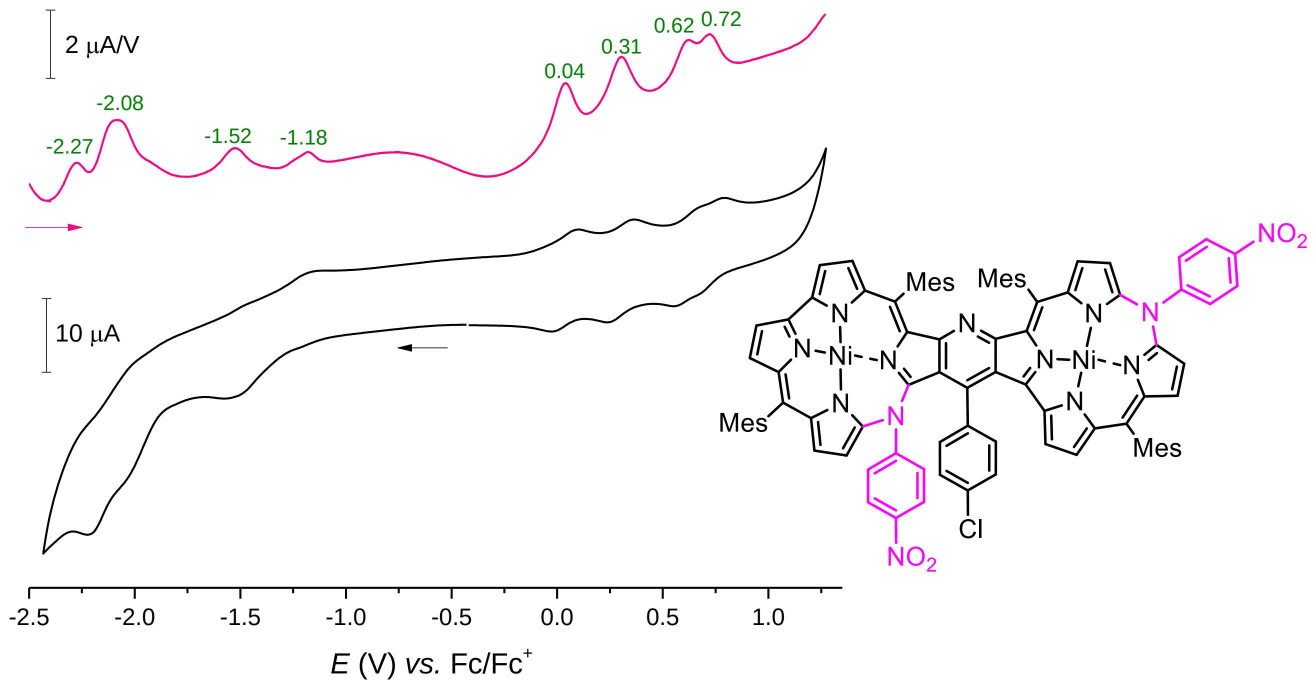

Figure S114. Differential pulse (DP) and cyclic (CV) voltammograms recorded for **5ab** in DCM with $[Bu_4N]PF_6$ as supporting electrolyte.

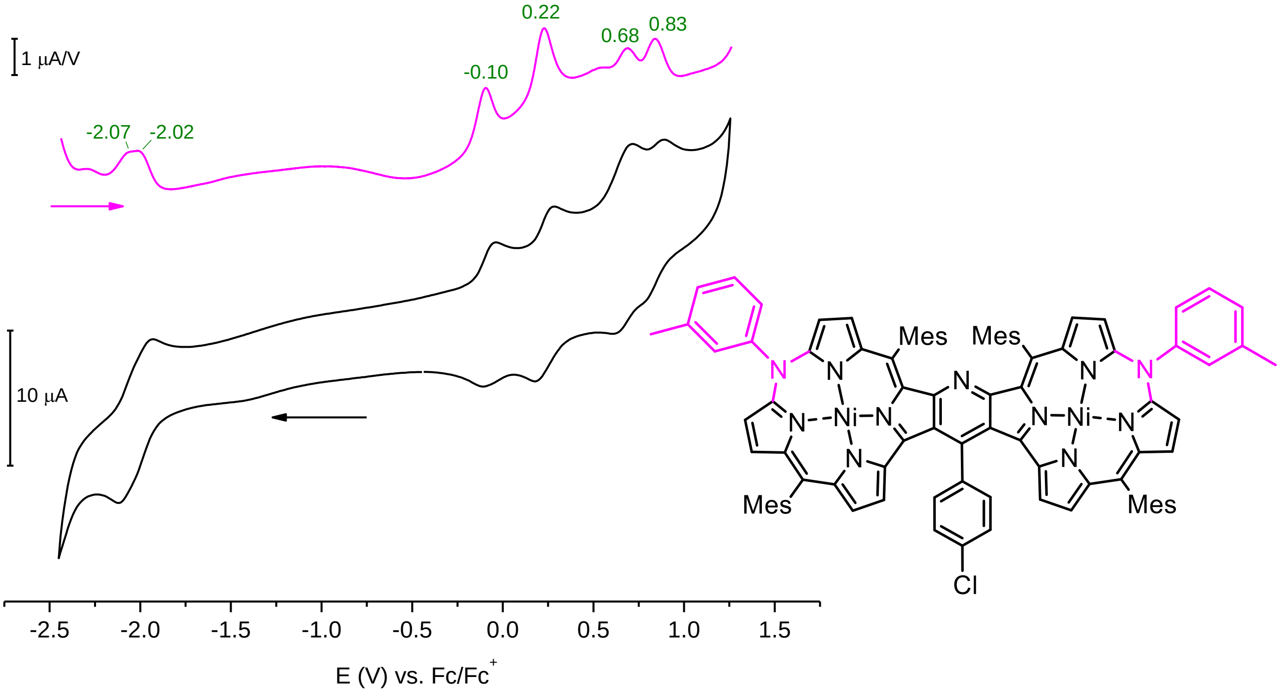

Figure S115. Differential pulse (DP) and cyclic (CV) voltammograms recorded for **5bb-1** in DCM with $[Bu_4N]PF_6$ as supporting electrolyte.

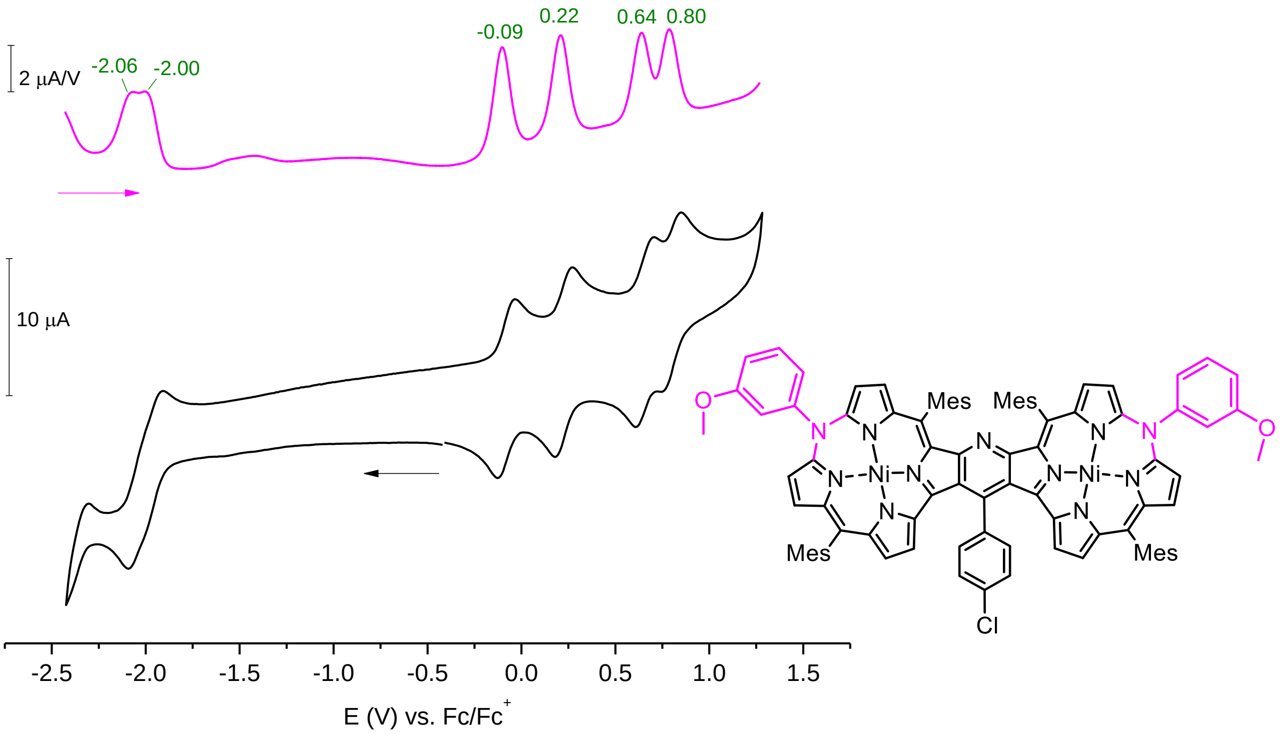

Figure S116. Differential pulse (DP) and cyclic (CV) voltammograms recorded for **5bb-2** in DCM with $[Bu_4N]PF_6$ as supporting electrolyte.

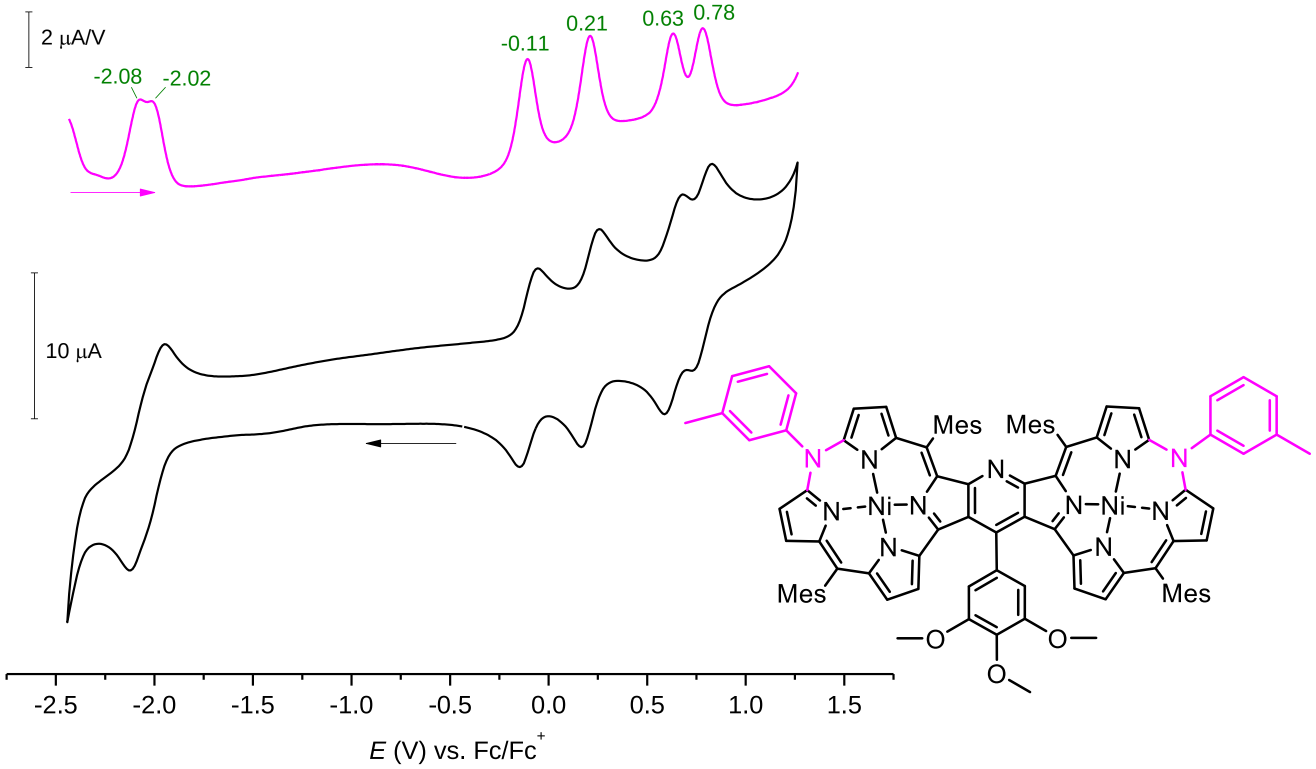

Figure S117. Differential pulse (DP) and cyclic (CV) voltammograms recorded for **5bb-3** in DCM with $[Bu_4N]PF_6$ as supporting electrolyte.

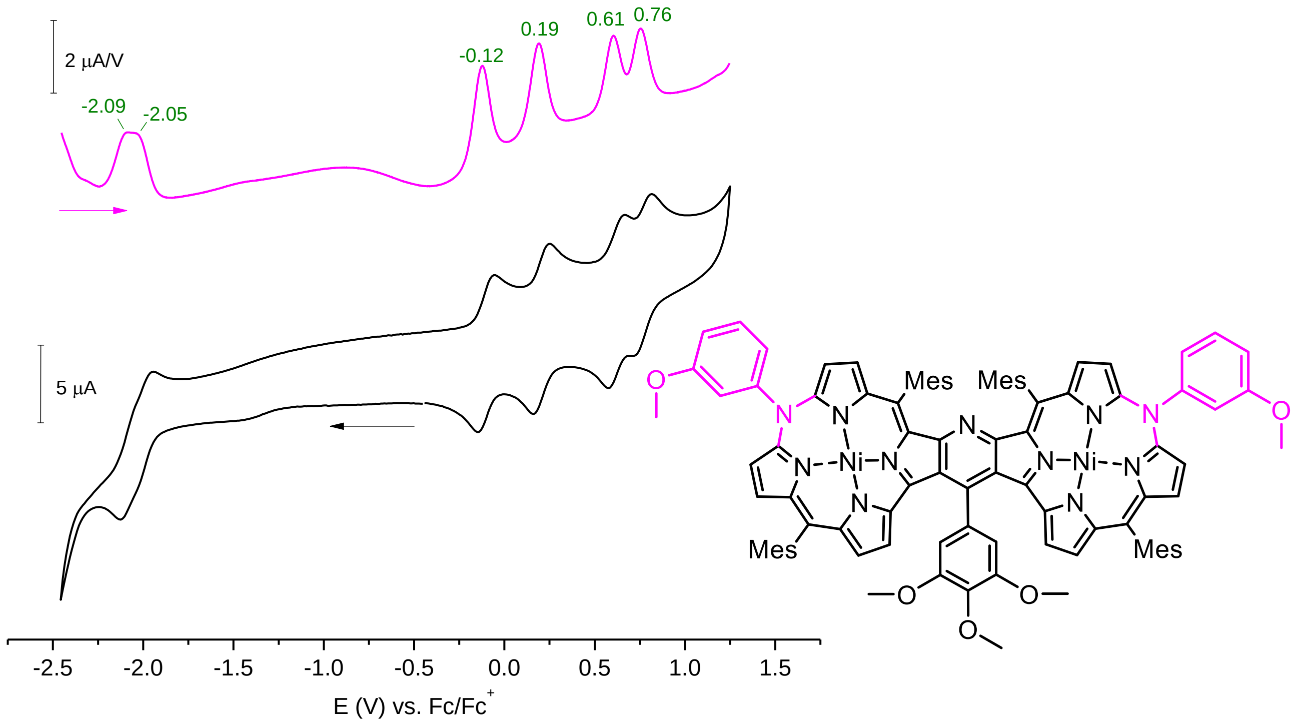

Figure S118. Differential pulse (DP) and cyclic (CV) voltammograms recorded for **5bb-4** in DCM with $[Bu_4N]PF_6$ as supporting electrolyte.

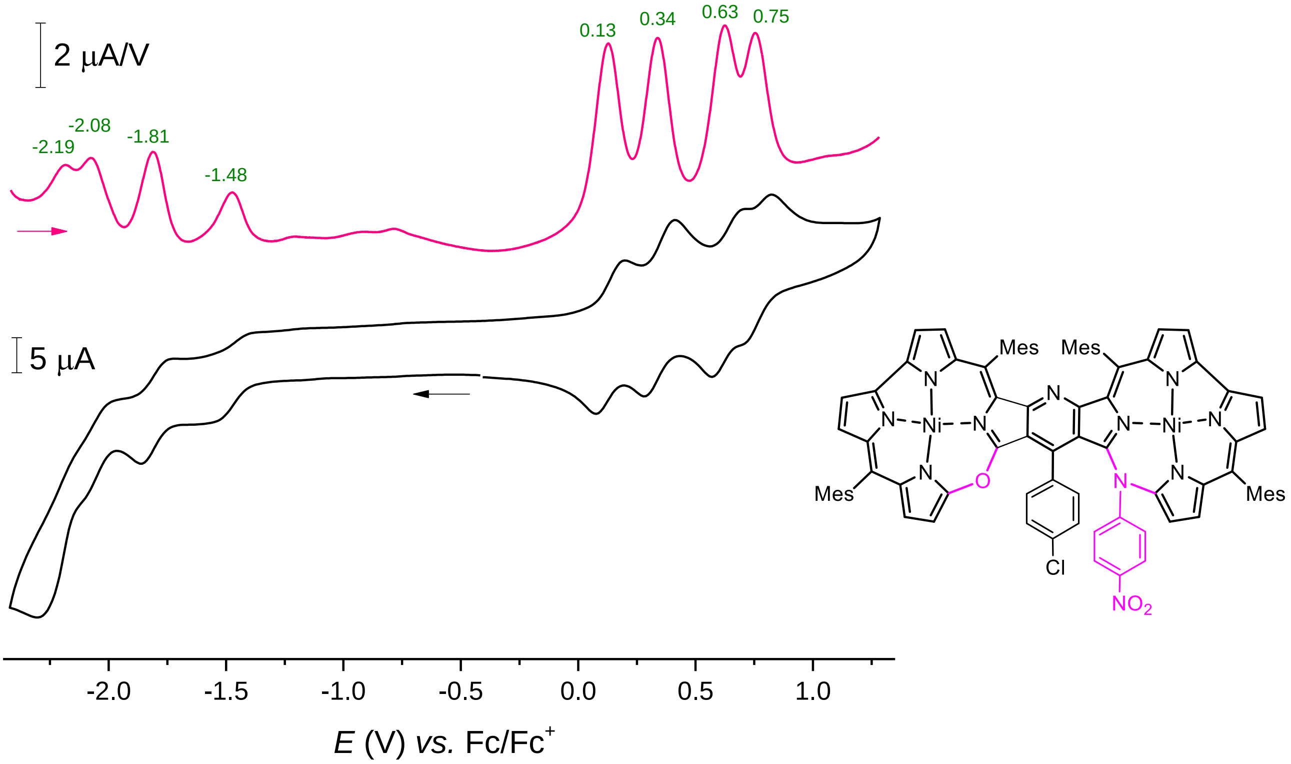

Figure S119. Differential pulse (DP) and cyclic (CV) voltammograms recorded for **6a** in DCM with $[Bu_4N]PF_6$ as supporting electrolyte.

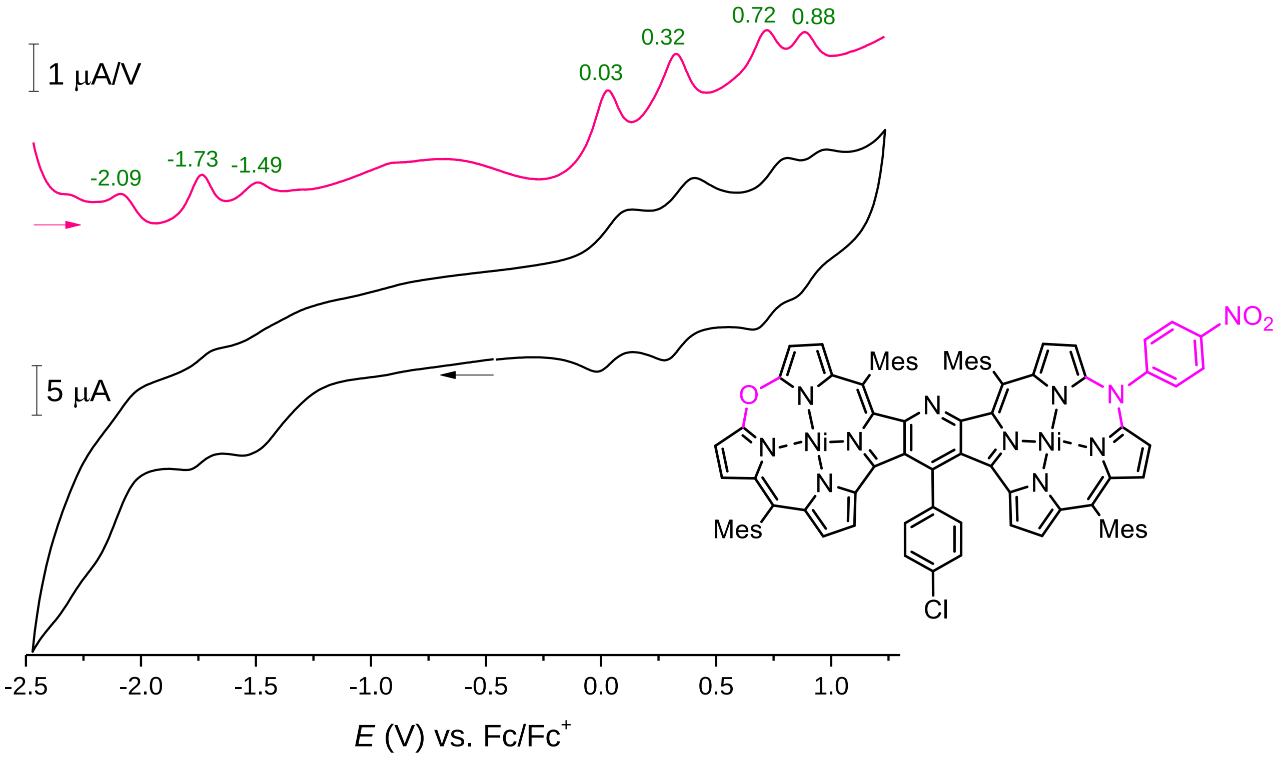

Figure S120. Differential pulse (DP) and cyclic (CV) voltammograms recorded for **6b** in DCM with $[Bu_4N]PF_6$ as supporting electrolyte.

Table S1. Collected electrochemical data (potential *E* vs. $Fc^{0/+}$ couple in volts).

System	E_{red1}	E_{red2}	E_{red3}	E_{red4}	E_{ox1}	E_{ox2}	E_{ox3}	E_{ox4}	E_{ox5}	$\Delta E_{ox1\text{-}red1}$
**5aa**	(−1.18,<br>−1.54)[a]<br>−2.04	−2.17	−2.41		0.11	0.32	0.58	0.64		2.15
**5ab**	(−1.18,<br>−1.52)[a]<br>−2.08	−2.27			0.04	0.31	0.62	0.72		2.12
**5bb-1**	−2.02	−2.07			−0.10	0.22	0.68	0.83		1.92
**5bb-2**	−2.00	−2.06			−0.09	0.22	0.64	0.80		1.91
**5bb-3**	−2.08	−2.02			−0.11	0.21	0.63	0.78		1.97
**5bb-4**	−2.09	−2.05			−0.12	0.19	0.61	0.76		1.93
**4a**	−0.99	−1.54[a]	−1.63	−2.18	0.04	0.33	0.67	0.89		1.03
**4b**	−1.00	−1.47[a]	−1.63	−2.19	0.02	0.32	0.71	0.87	1.23	1.02
**3**[c]	−0.87	−1.12	−1.66	−1.93	0.03	0.28	0.74	0.88	1.24	0.90
**6a**	(−1.48)[a]<br>−1.81	−2.08	−2.19		0.13	0.34	0.63	0.75		1.94
**6b**	(−1.49)[a]<br>−1.73	−2.09	−2.30		0.03	0.32	0.72	0.88		1.76
**2**-NO_2<br>(3MePh)	−1.57	−1.99	−2.15		0.41	0.90	1.06			1.98
**2**-NO_2<br>(3MeOPh)	−1.59	−2.00			0.38	0.86	1.04			1.97
**2**-NH_2<br>(3MePh)	−2.15				−0.13	0.46				2.02
**2**-H<br>(*p*-NO_2Ph)[d]	(−1.11,<br>−1.47)[a]<br>−2.15				0.21	0.68				2.36
**2**-H<br>(3MePh)[d]	−2.09				0.16	0.68				2.25
**2**-H<br>(3MeOPh)[d]	−2.10				0.15	0.66				2.25

[a] Irreversible waves of the NO_2 group reductions. [b] Irreversible wave. [c] Data from ref. [18] [d] Data from ref. [19]

14. Calculation results, part 1

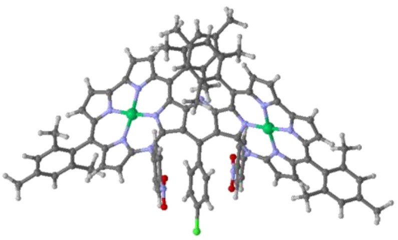

5aa
33.8 kcal/mol

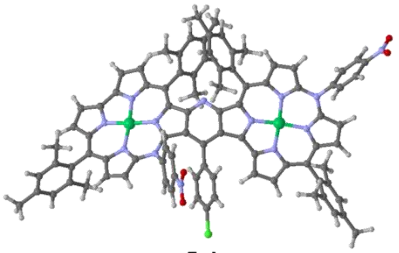

5ab
18.7 kcal/mol

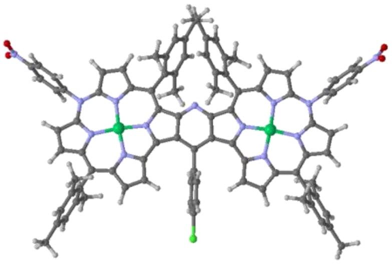

5bb-5
0 kcal/mol

Figure S121. Calculated relative Gibbs free energies and optimized structures of isomeric pyridine-fused bis(azacorroles).

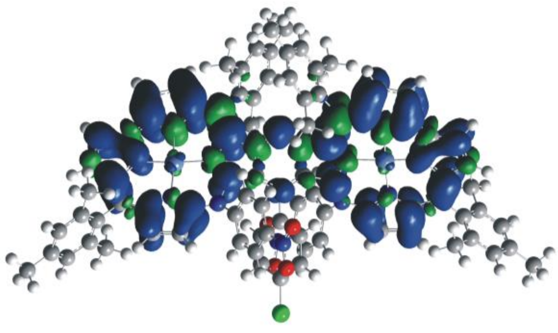

[**5aa**]$^{+\bullet}$

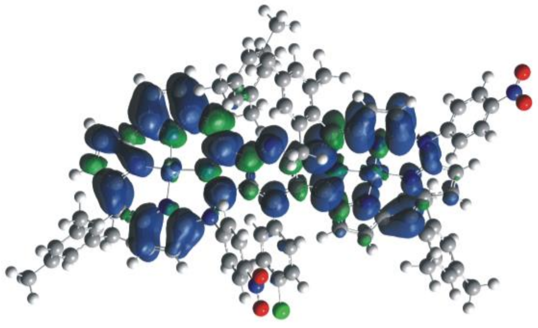

[**5ab**]$^{+\bullet}$

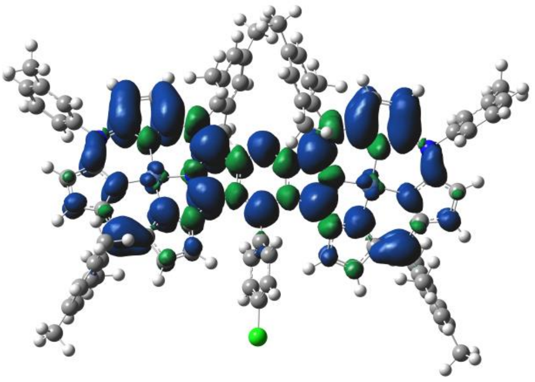

[**5bb-1**]$^{+\bullet}$

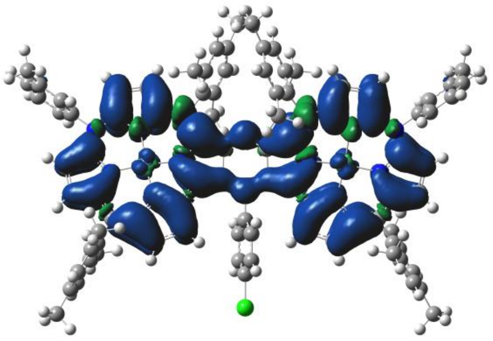

t-[**5bb-1**]$^{2+}$

Figure S122. Calculated spin density distribution (isovalue 0.0004) in the oxidation products of **5aa**, **5ab**, and **5bb-1**.

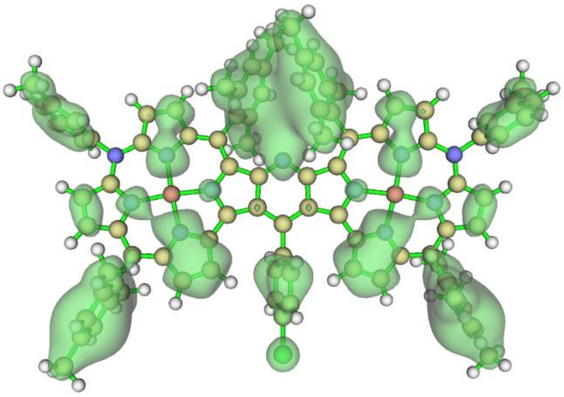

[5bb-1]$^{\bullet+}$

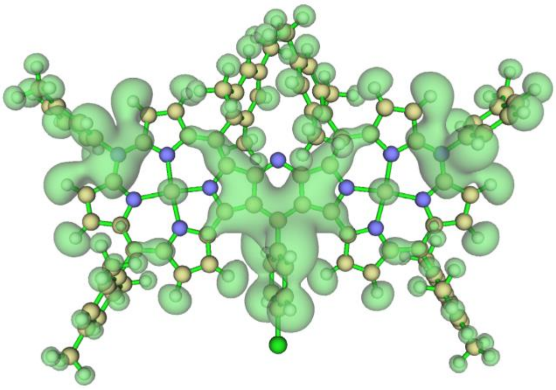

s-[**5bb-1**]$^{2+}$

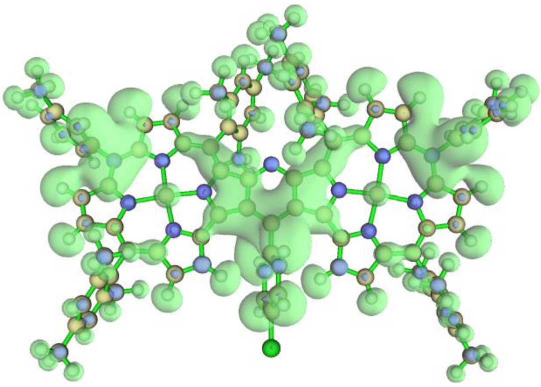

t-[**5bb-1**]$^{2+}$

Figure S123. Positive charge distributions (green surface, isovalue 0.15) over the **[5bb-1]**$^{\bullet+}$, *s*-[**5bb-1**]$^{2+}$, and *t*-[**5bb-1**]$^{2+}$ molecular skeletons derived from the NBO approach applied to DFT-optimized structures.

15. ESR spectra of the oxidized species and VT experiments

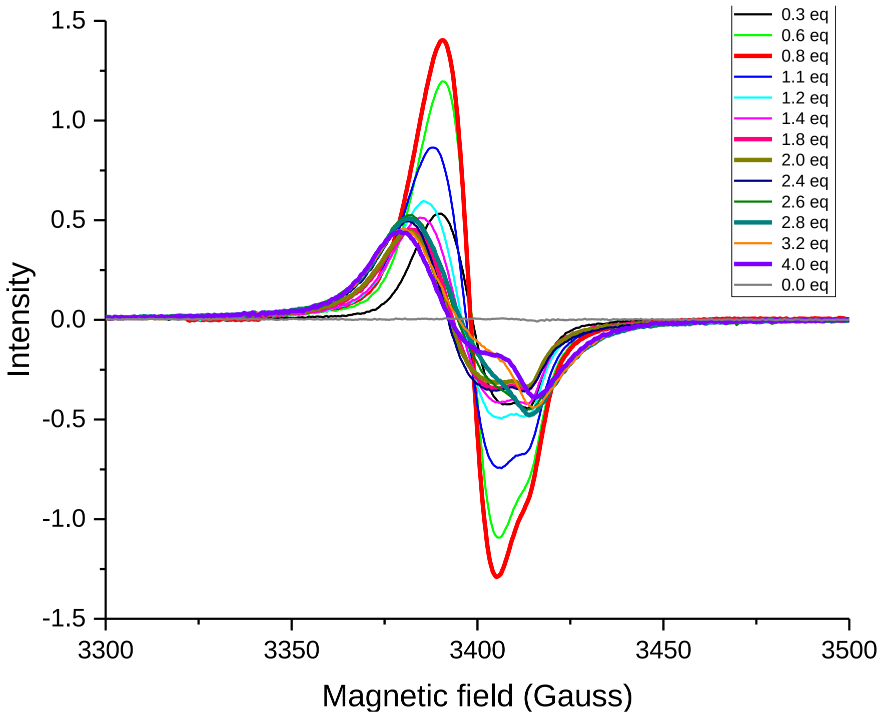

Figure S124. Frozen-solution ESR spectra recorded upon titration of **5aa** with BAHA in DCM at 140 K.

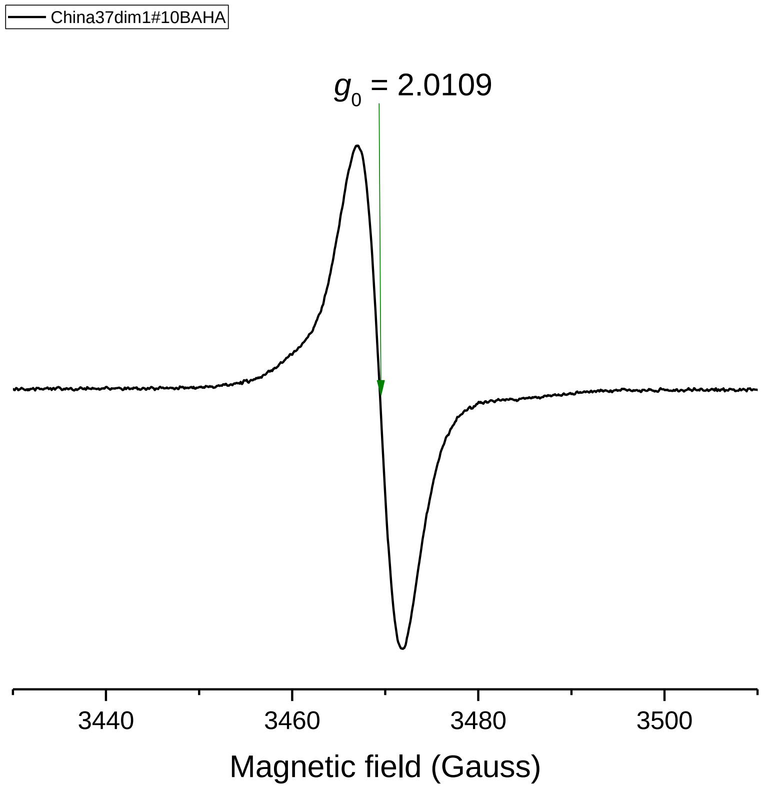

Figure S125. Liquid-solution ESR spectrum of [**5aa**]$^{\bullet+}$ recorded upon titration of **5aa** with BAHA in DCM at 300 K.

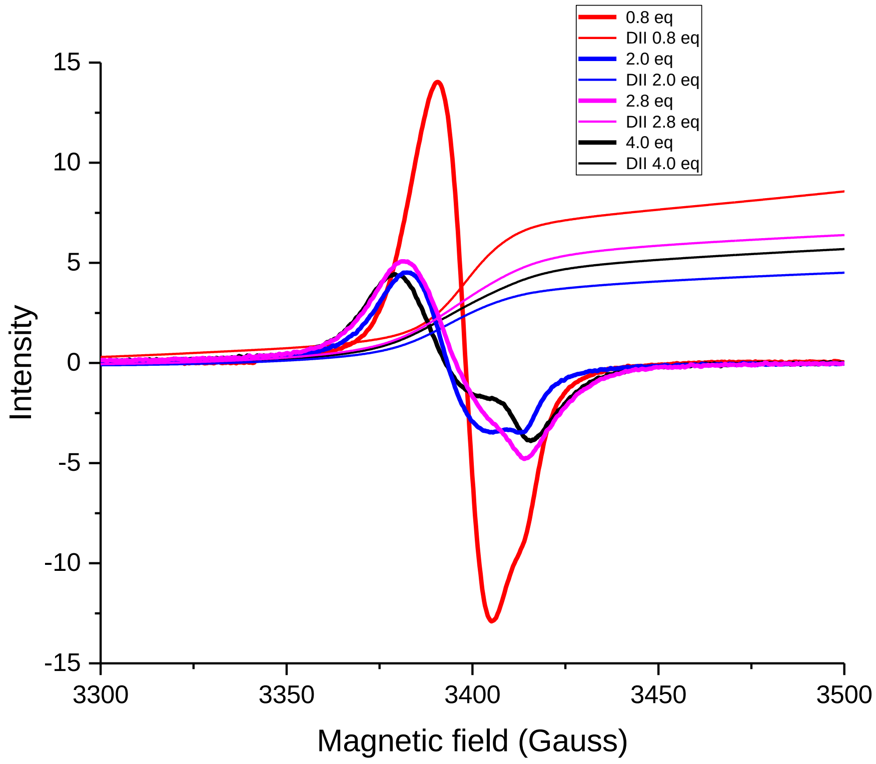

Figure S126. Selected frozen-solution 1-derivative ESR spectra recorded upon titration of **5aa** with BAHA in DCM at 140 K along with doubly integrated ESR spectra.

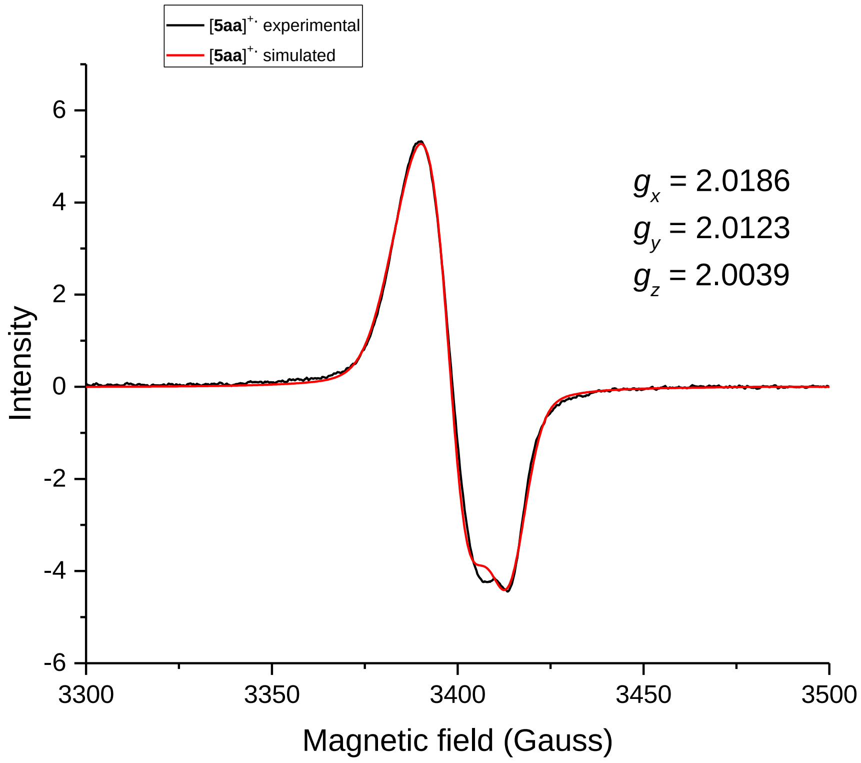

Figure S127. Experimental (black trace) and simulated (red trace) ESR spectra of frozen-solution of [**5aa**]$^{+\bullet}$ in DCM at 140 K.

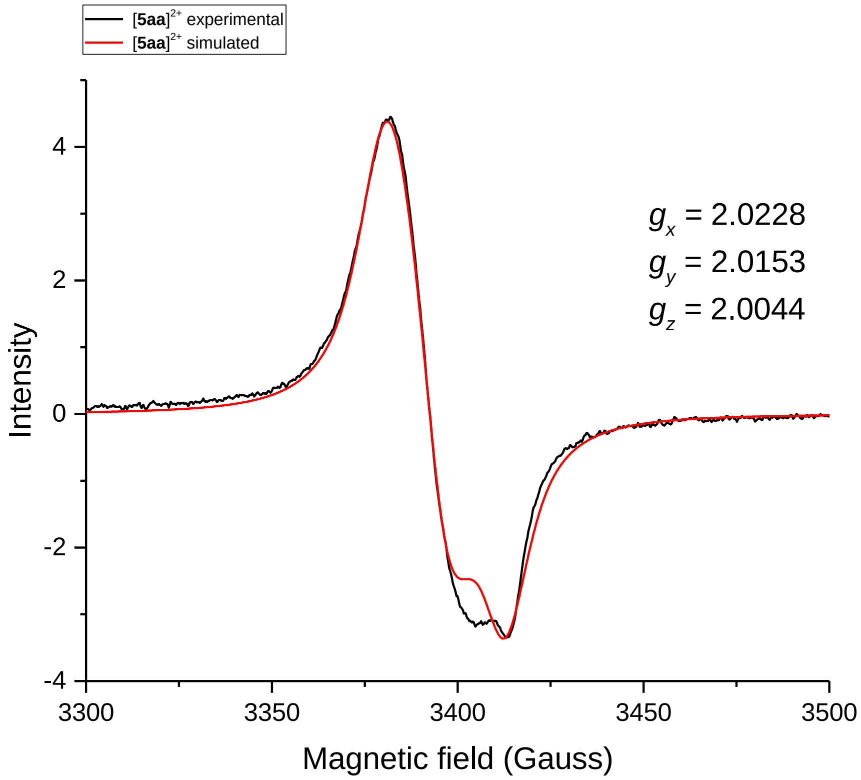

Figure S128. Experimental (black trace) and simulated (red trace) ESR spectra of frozen-solution of **5aa** upon addition of 2 equiv. of BAHA (DCM, 140 K).

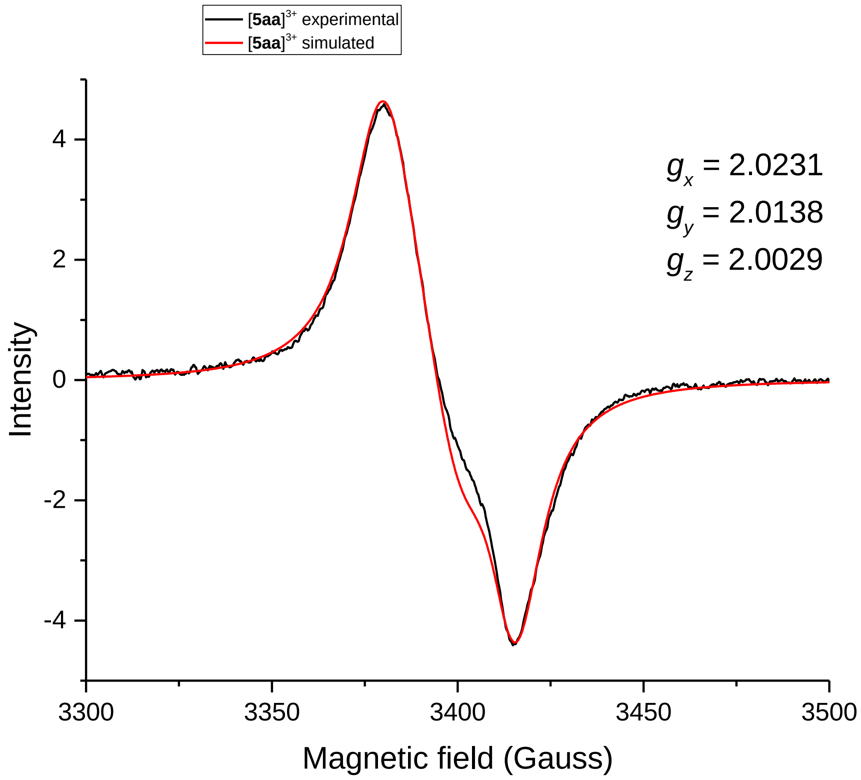

Figure S129. Experimental (black trace) and simulated (red trace) ESR spectra of frozen-solution of **5aa** upon addition of 2 equiv. of BAHA (DCM, 140 K).

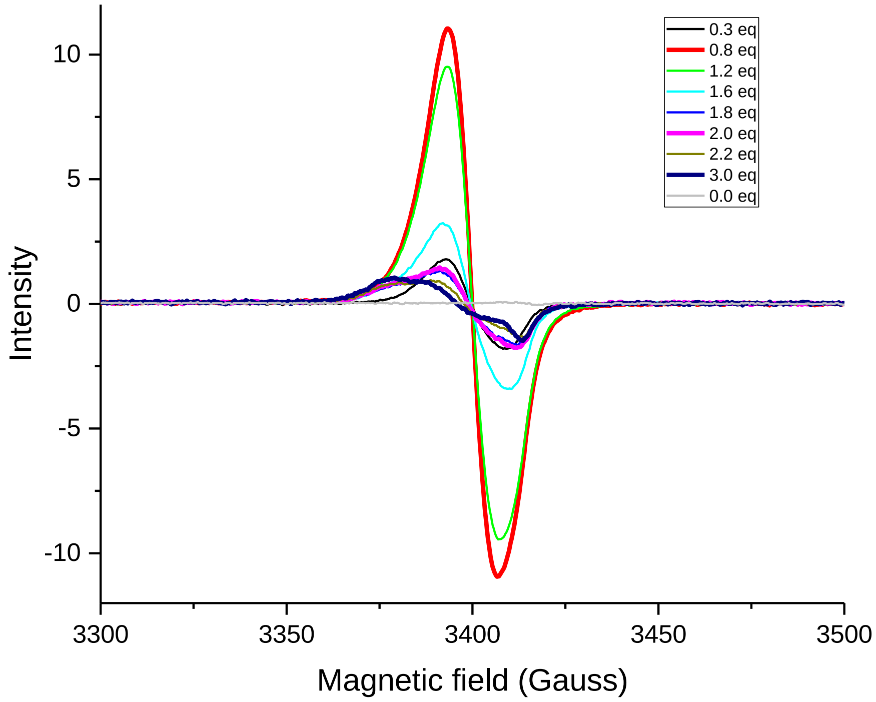

Figure S130. Selected frozen-solution ESR spectra recorded upon titration of **5ab** with BAHA in DCM at 140 K.

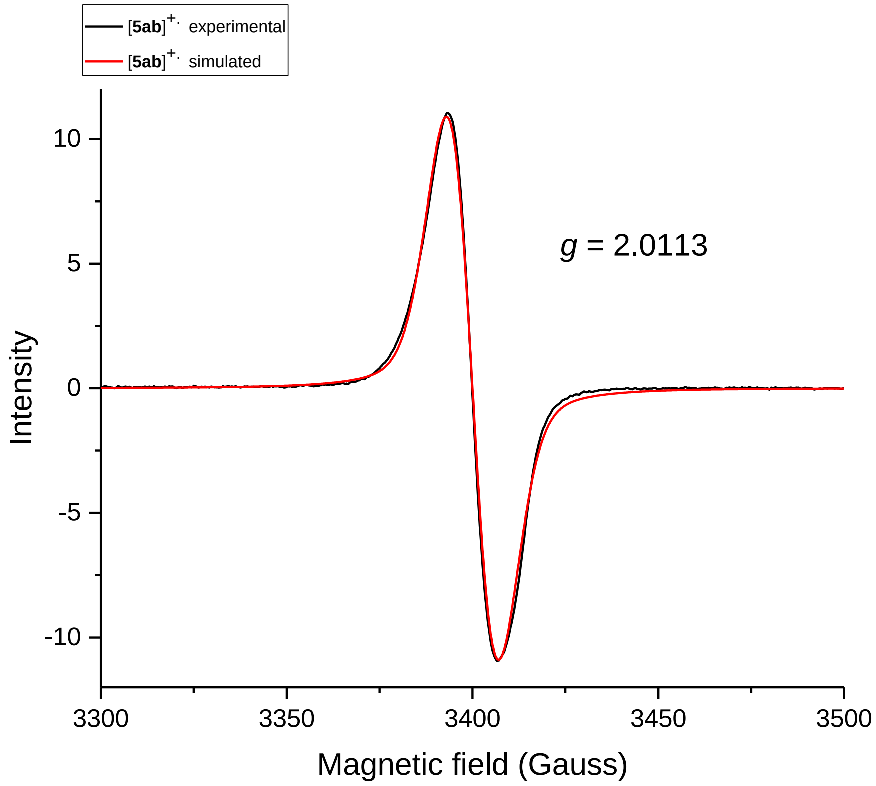

Figure S131. Experimental (black trace) and simulated (red trace) ESR spectra of frozen-solution of [**5ab**]$^{\bullet+}$ (DCM, 140 K).

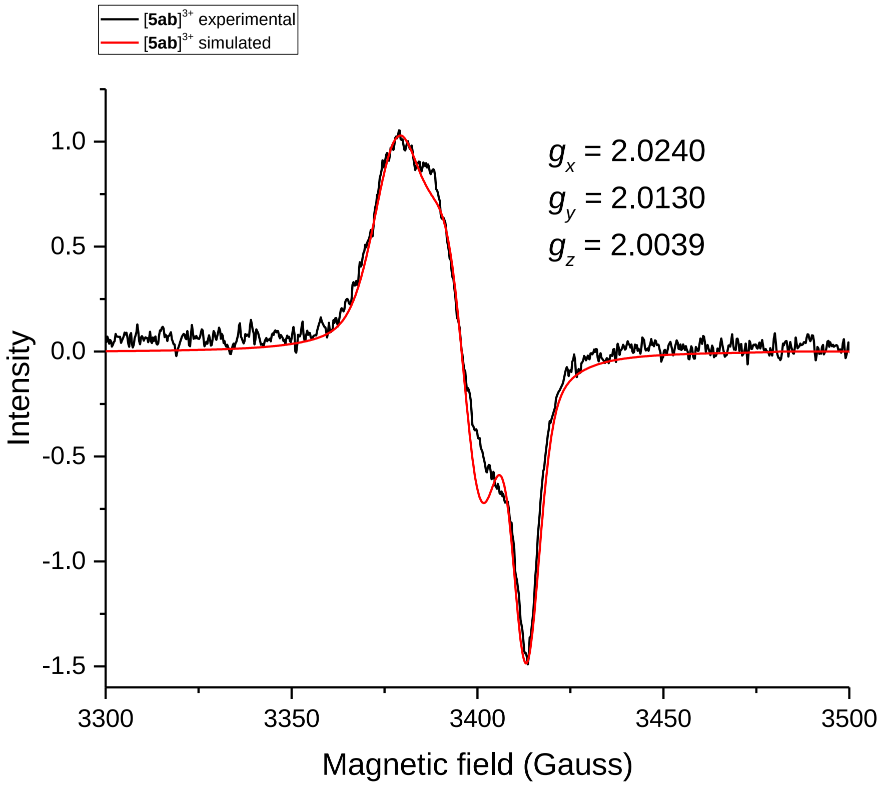

Figure S132. Experimental (black trace) and simulated (red trace) ESR spectra of frozen-solution of **5ab** upon addition of 3 equiv. of BAHA (DCM, 140 K).

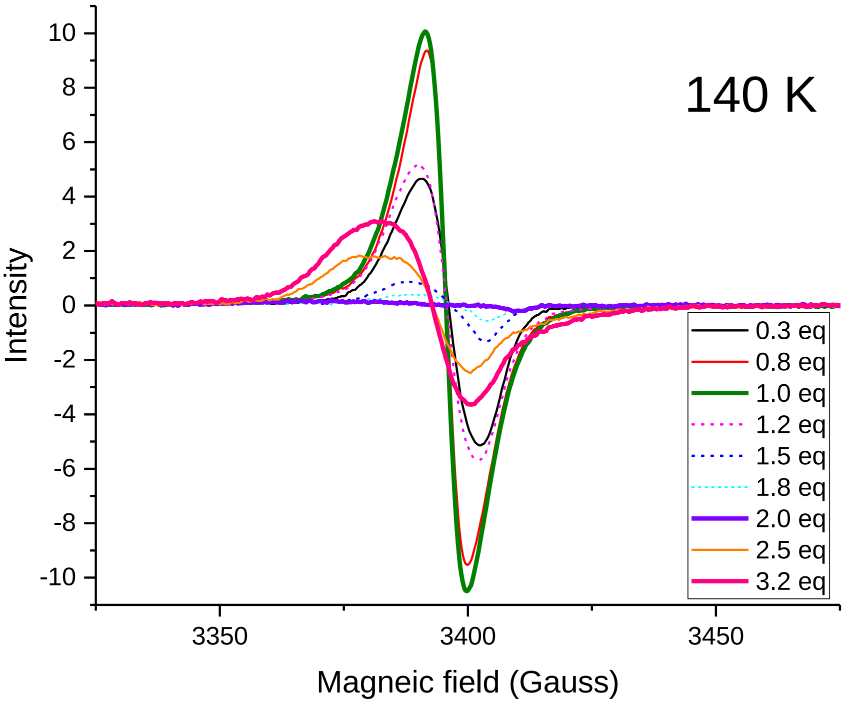

Figure S133. Frozen-solution ESR spectra recorded upon titration of **5bb-2** with BAHA in DCM at 140 K.

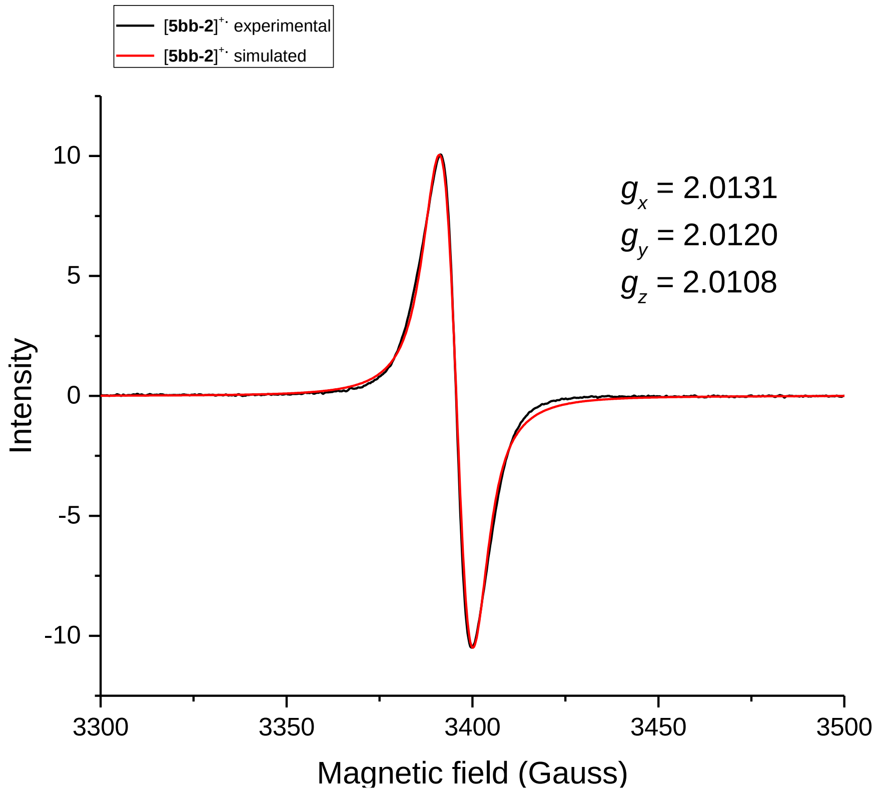

Figure S134. Experimental (black trace) and simulated (red trace) ESR spectra of frozen-solution of [**5bb-2**]$^{•+}$ (DCM, 140 K).

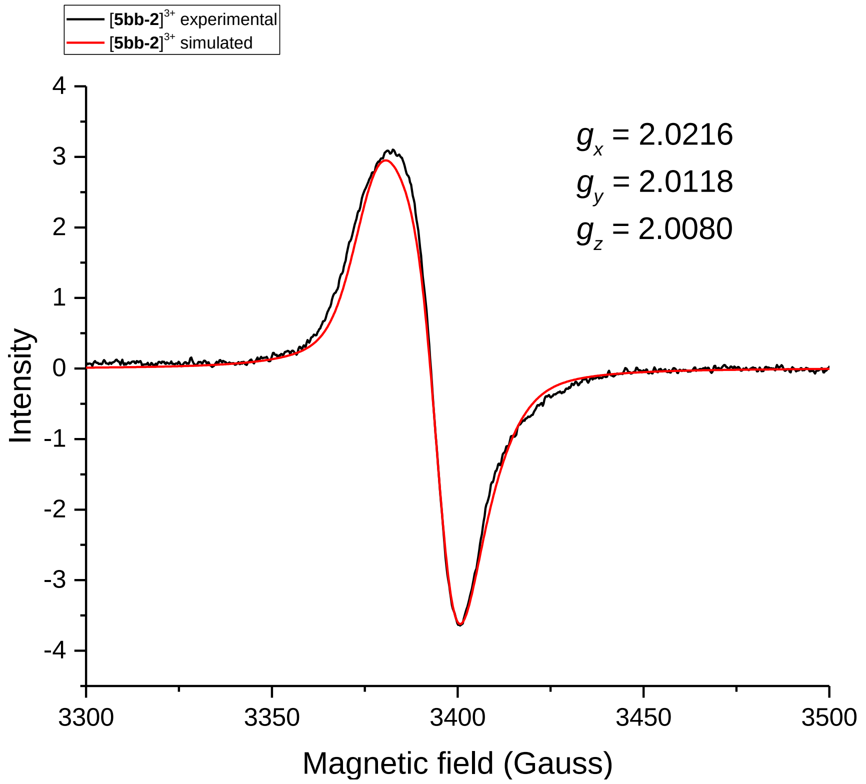

Figure S135. Experimental (black trace) and simulated (red trace) ESR spectra of frozen-solution of **5bb-2** upon addition of 3.2 equiv. of BAHA (DCM, 140 K).

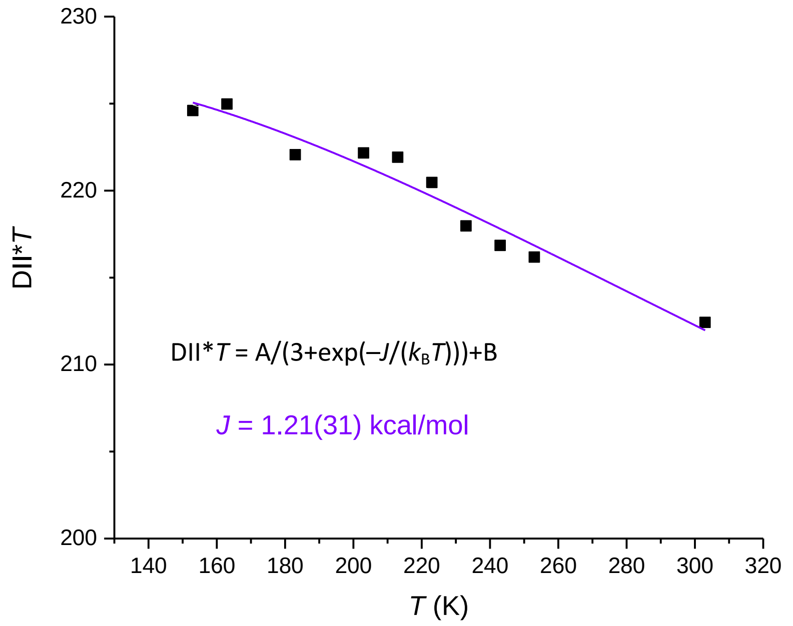

Figure S136. Temperature dependence of the product of doubly integrated ESR signal intensity (DII) of solid [**5ab**]$(SbCl_6)_2$ and temperature (*T*) (black squares) and calculated curve of best fit of the Bleaney-Bower's equation[20-22] (purple trace). The solid sample was prepared by addition of 2 equiv. of BAHA to the solution of **5ab** in dichloromethane, precipitation with hexane, decantation of the solvents after sedimentation of the solid, and drying of the solid *in vacuo.*

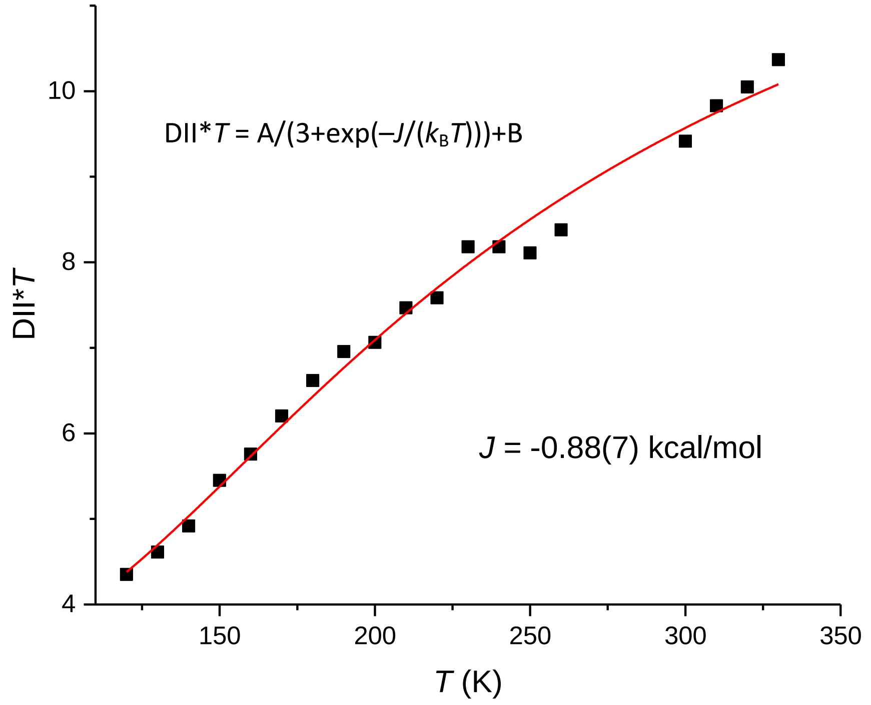

Figure S137. Temperature dependence of the product of doubly integrated ESR signal intensity (DII) of solid [**5bb-2**]$(SbCl_6)_2$ and temperature (*T*) (black squares) and calculated curve of best fit of the Bleaney-Bower's equation[20-22] (red trace). The solid sample was prepared by addition of 2 equiv. of BAHA to the solution of **5bb-2** in dichloromethane, precipitation with hexane, decantation of the solvents after sedimentation of the solid, and drying of the solid *in vacuo.*

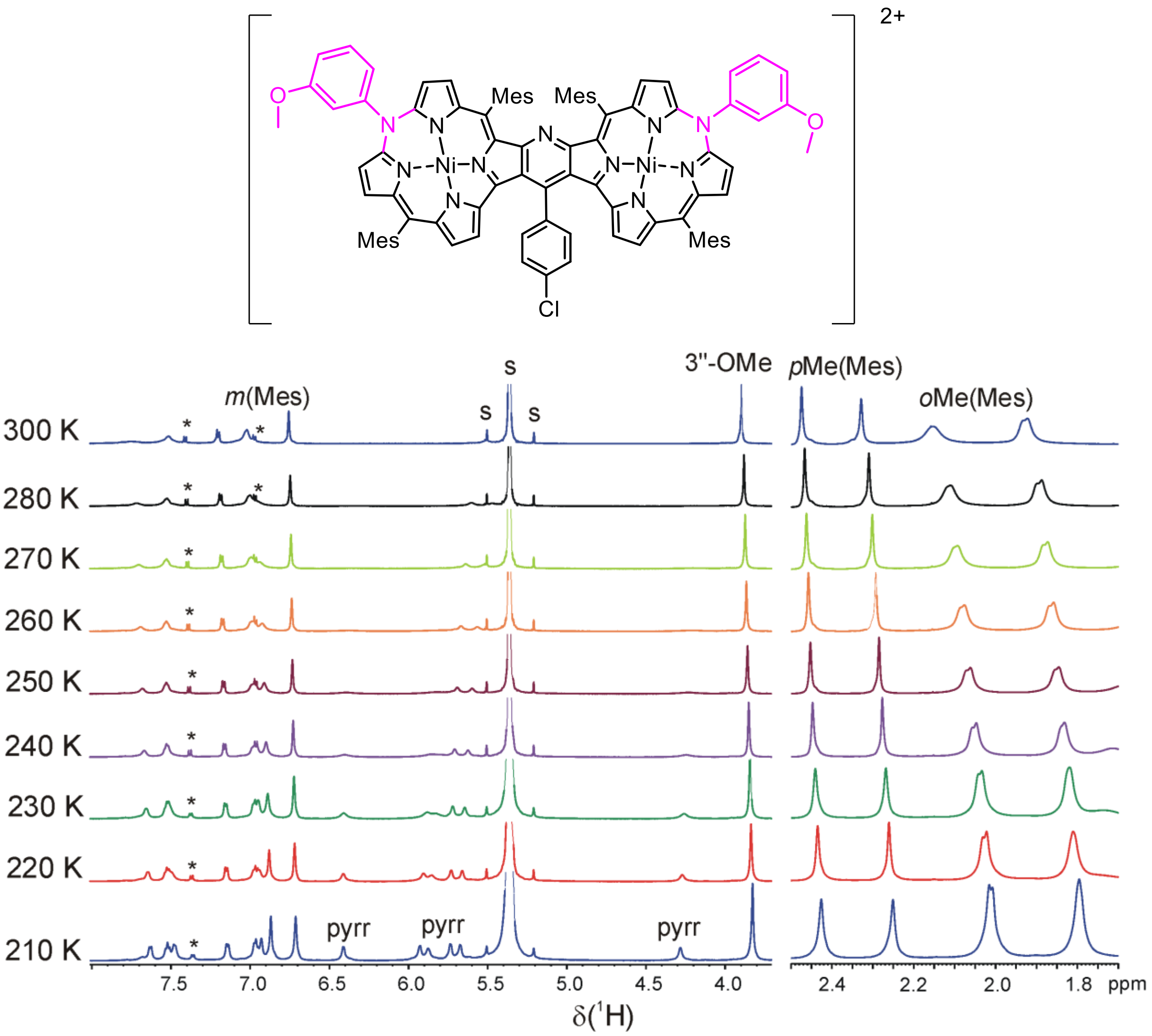

Figure S138. ^{1}H NMR spectra recorded for the solution of [**5bb-2**]$^{2+}$ in CD_2Cl_2 at specified temperatures. The signals of residual tris(bromophenyl)amine, i.e. reduced form of BAHA are marked with asterisks; s, $CHDCl_2$ signal.

16. Chirality of **5aa** and [**5aa**]$^{\bullet+}$

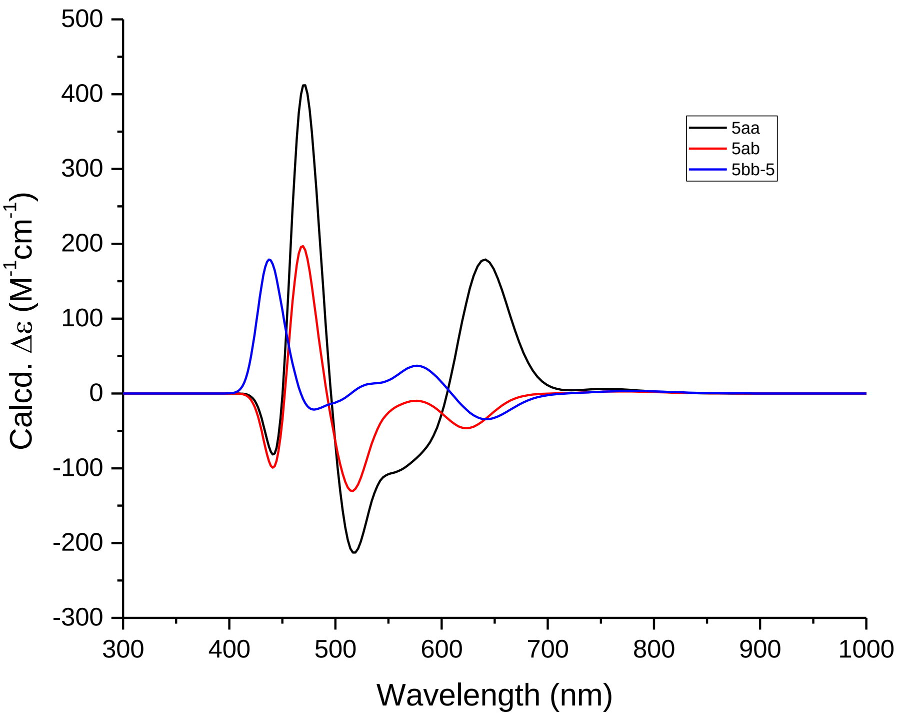

Figure S139. TD DFT calculated CD spectra of one of the enantiomers of **5aa** (black), **5ab** (red), and **5bb-5** (blue).

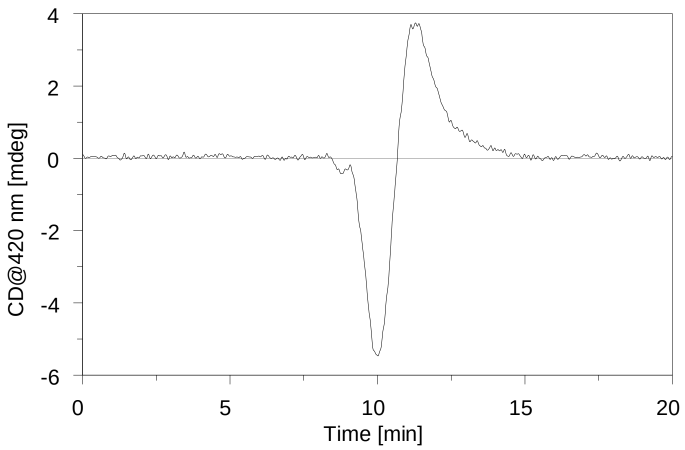

Figure S140. Chiral stationary phase HPLC profile (Chirex 3014, DCM/hexane 15/85 v/v, 2 mL/min) of **5aa** recorded by means of CD intensity monitoring at 420 nm.

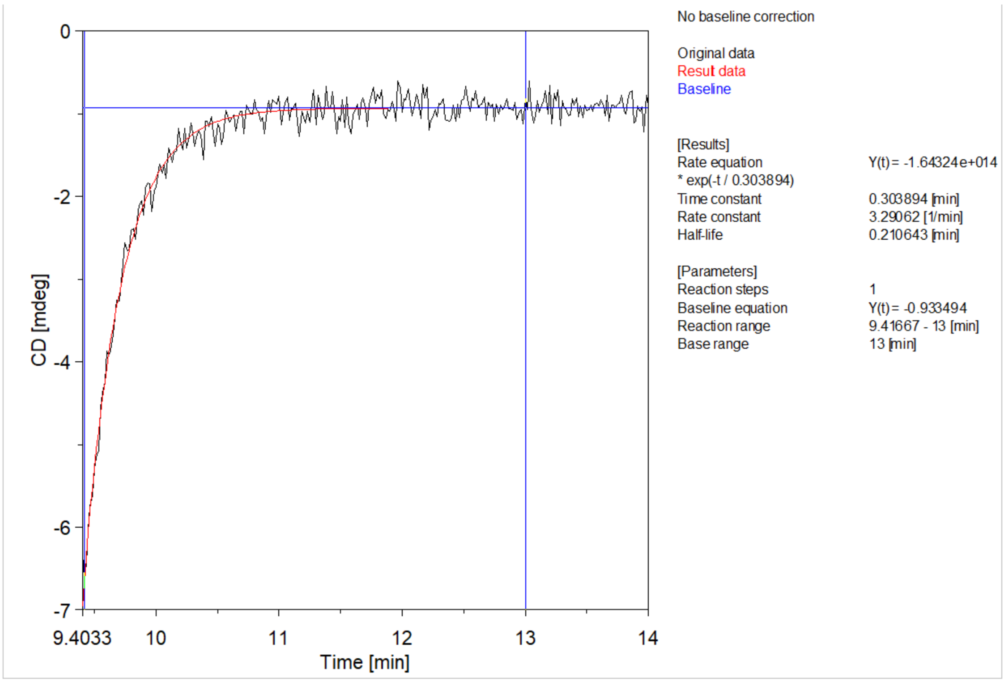

Figure S141. Changes of the CD intensity of the first peak over time for the stopped-flow HPLC of **5aa** (Chirex 3014, DCM/hexane 15/85 v/v, 2 mL/min). The red trace is a best-fit first-order kinetic curve drawn with the parameters given on the right margin of the plot.

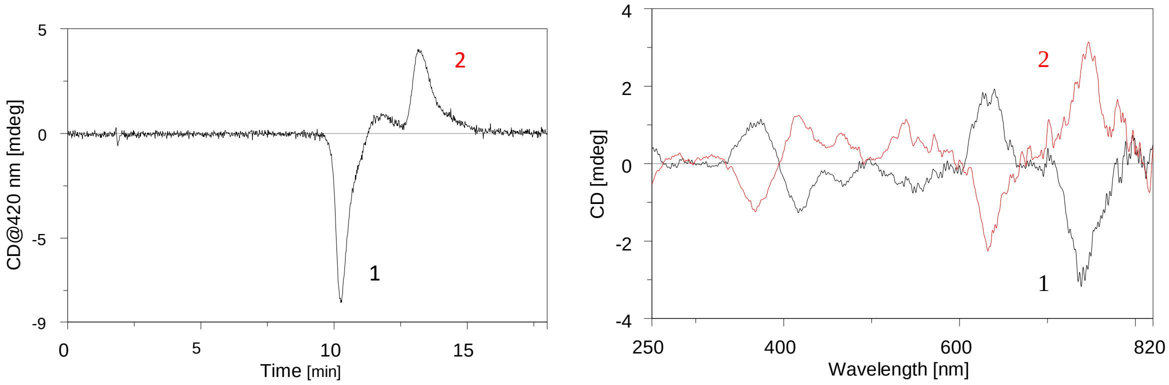

Figure S142. Left: Chiral stationary phase HPLC profile (Chirex 3014, DCM/hexane 15/85 v/v, 2 mL/min) of [**5aa**]$^{\bullet+}$ recorded by means of CD intensity monitoring at 420 nm. The radical cation [**5aa**]$^{\bullet+}$ was obtained by the addition of 1 equiv. of BAHA to the solution of **5aa** in DCM. Right: CD spectra of the fractions entrapped in the flow cell upon the HPLC resolution under the above conditions with the flow stopped near the extrema of the first (1, black trace) and second (2, red trace) peak of the profile.

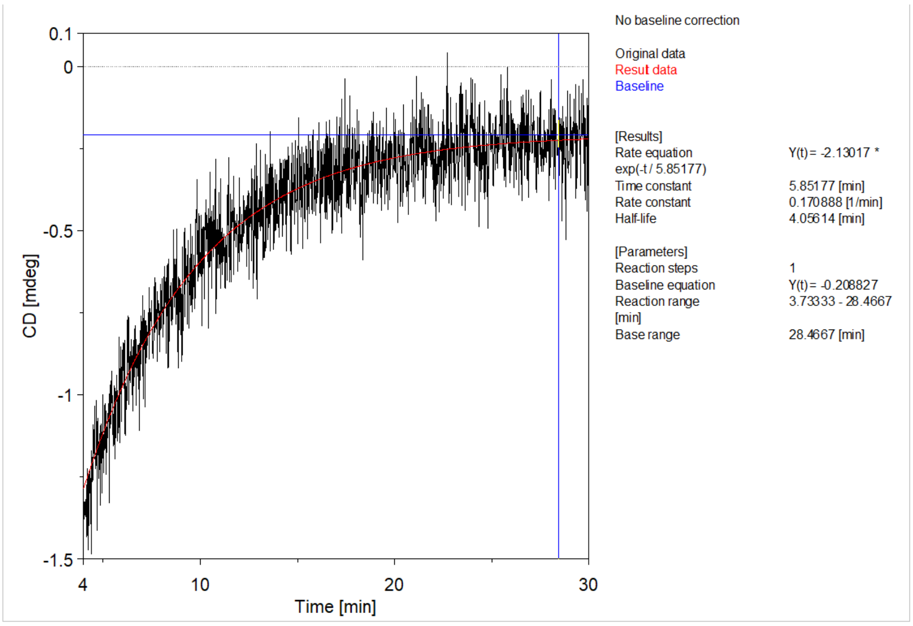

Figure S143. Changes of the CD intensity of the first peak over time for the stopped-flow HPLC of [**5aa**]$^{•+}$ (Chirex 3014, DCM/hexane 15/85 v/v, 2 mL/min). The red trace is a best-fit first-order kinetic curve drawn with the parameters given on the right margin of the plot.

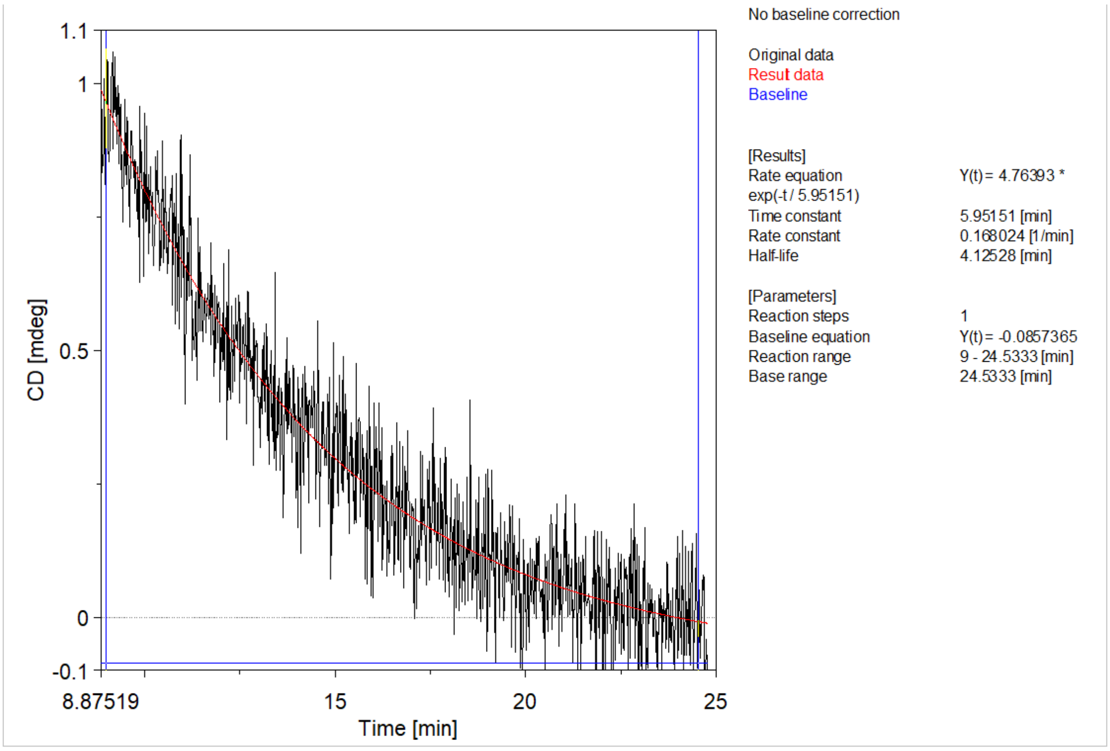

Figure S144. Changes of the CD intensity of the second peak over time for the stopped-flow HPLC of [**5aa**]$^{•+}$ (Chirex 3014, DCM/hexane 15/85 v/v, 2 mL/min). The red trace is a best-fit first order kinetic curve drawn with the parameters given on the right margin of the plot.

17. Spectrophotometric Titrations of Dimers with Oxidants

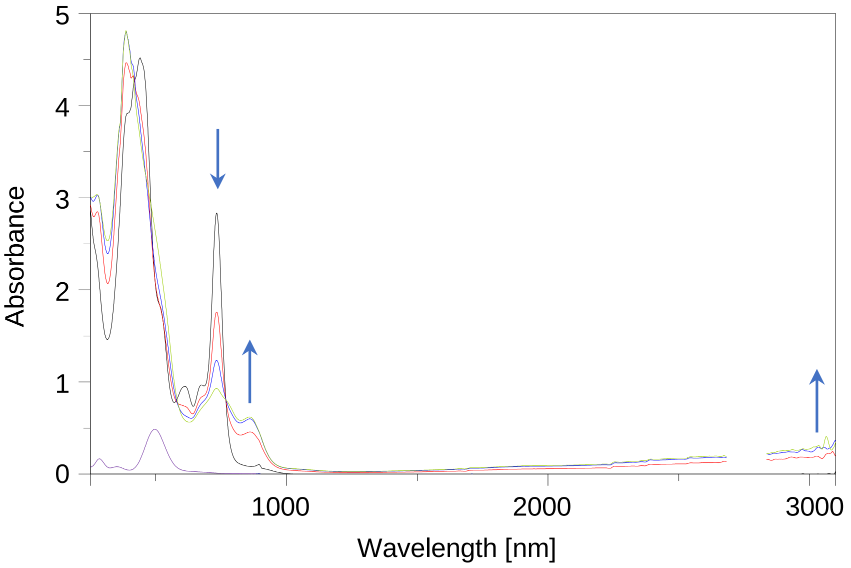

Figure S145. UV-vis-NIR spectrum of 5**aa** in DCM upon addition of I_2: 0 equiv. (black), 1.0 equiv. (red), 2.0 equiv. (blue), 2.5 equiv. (green). The spectrum of I_2 in DCM (purple trace) is shown for comparison.

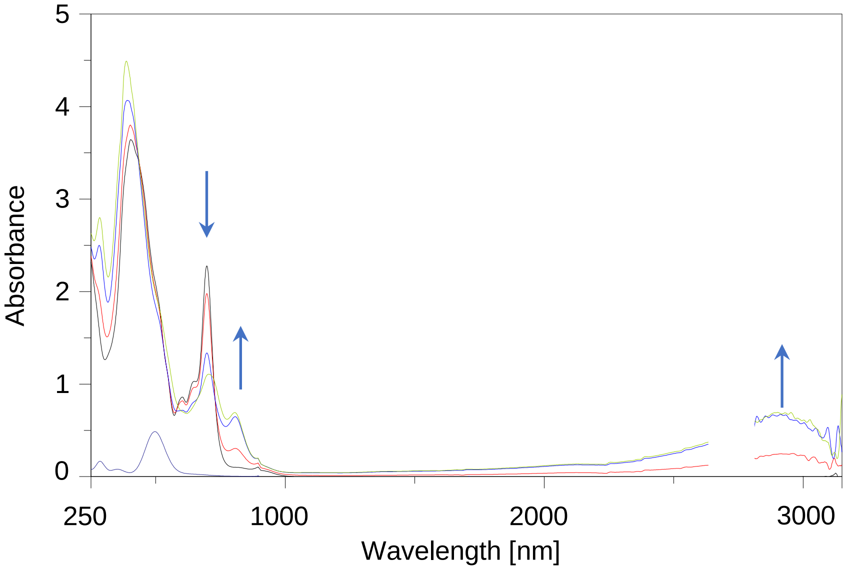

Figure S146. UV-vis-NIR spectra of 5**ab** in DCM recorded upon addition of I_2: 0 equiv. (black), 0.5 equiv. (red), 1.0 equiv. (blue), 1.5 equiv. (green). The spectrum of I_2 in DCM (purple trace) is shown for comparison.

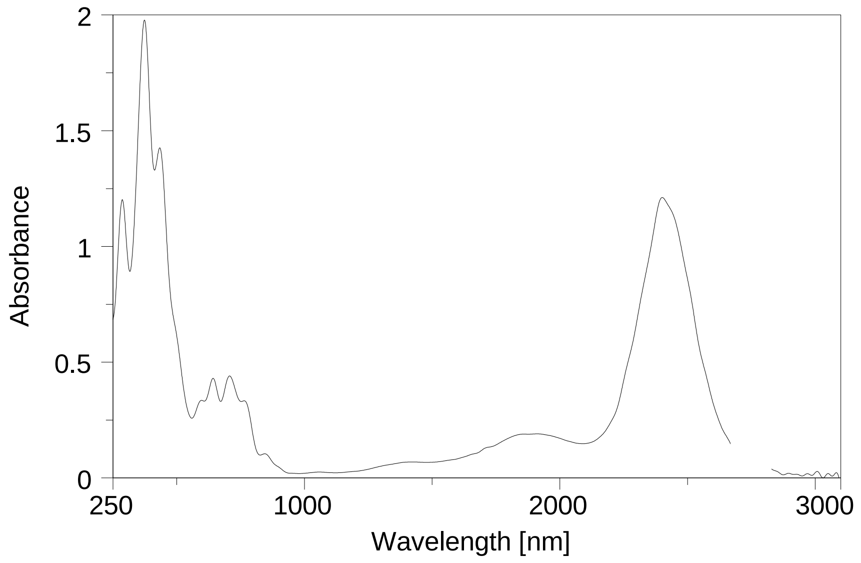

Figure S147. UV-vis-NIR spectrum of **5bb-1** in DCM upon addition of I_2 (2 equiv.).

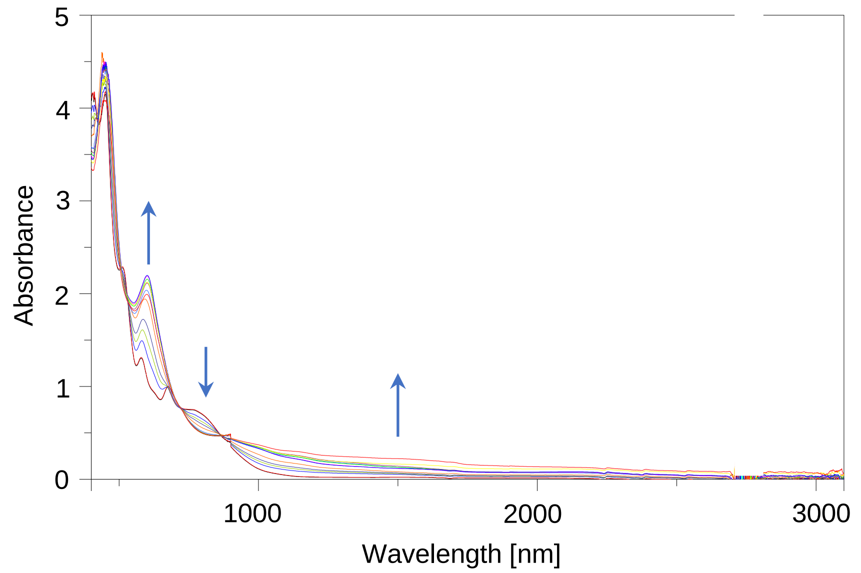

Figure S148. Electronic spectra (DCM) recorded upon titration of **4b** with BAHA (0-3 equiv.).

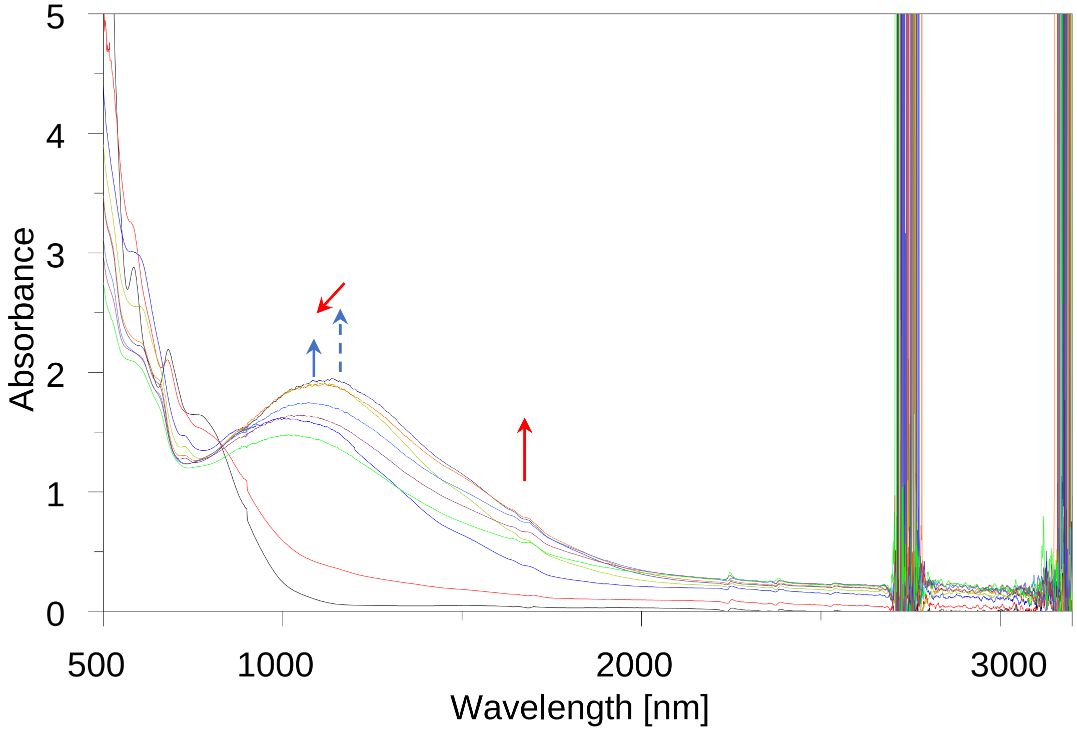

Figure S149. Electronic spectra (DCM) recorded upon titration of **4b** with $AgBF_4$ (0-4 equiv.).

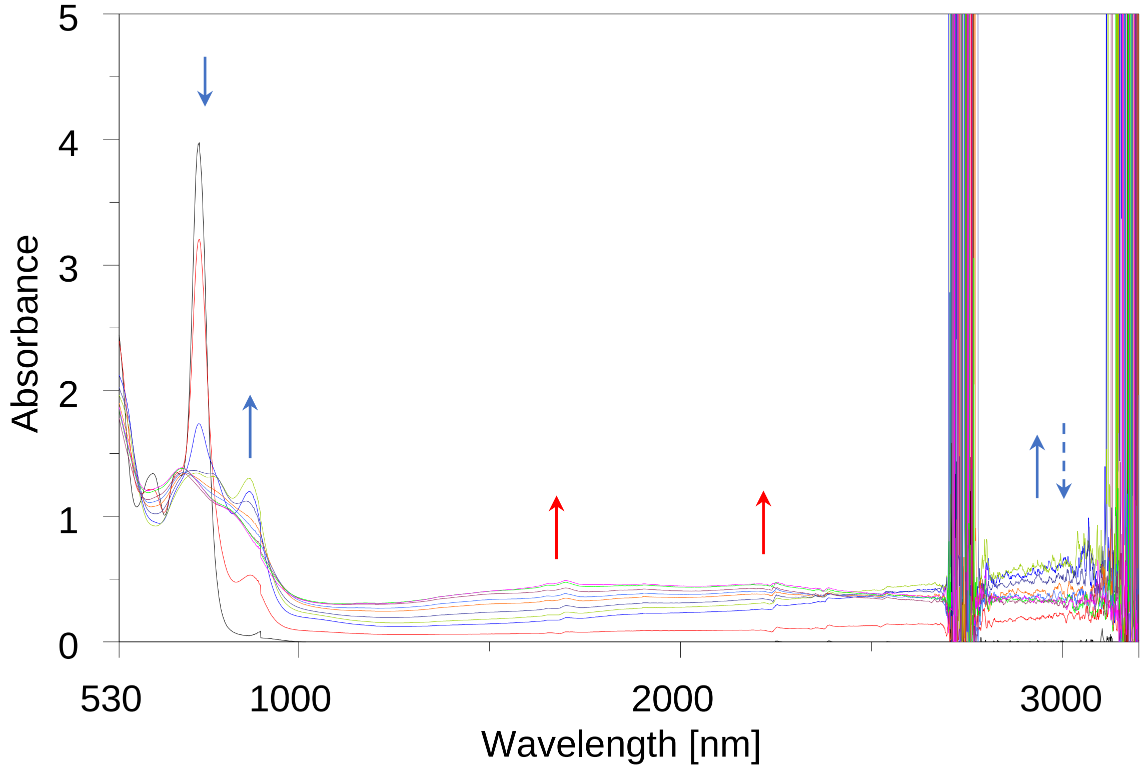

Figure S150. Electronic spectra (DCM) recorded upon titration of **5aa** with $AgBF_4$ in DCM (0-3 equiv.).

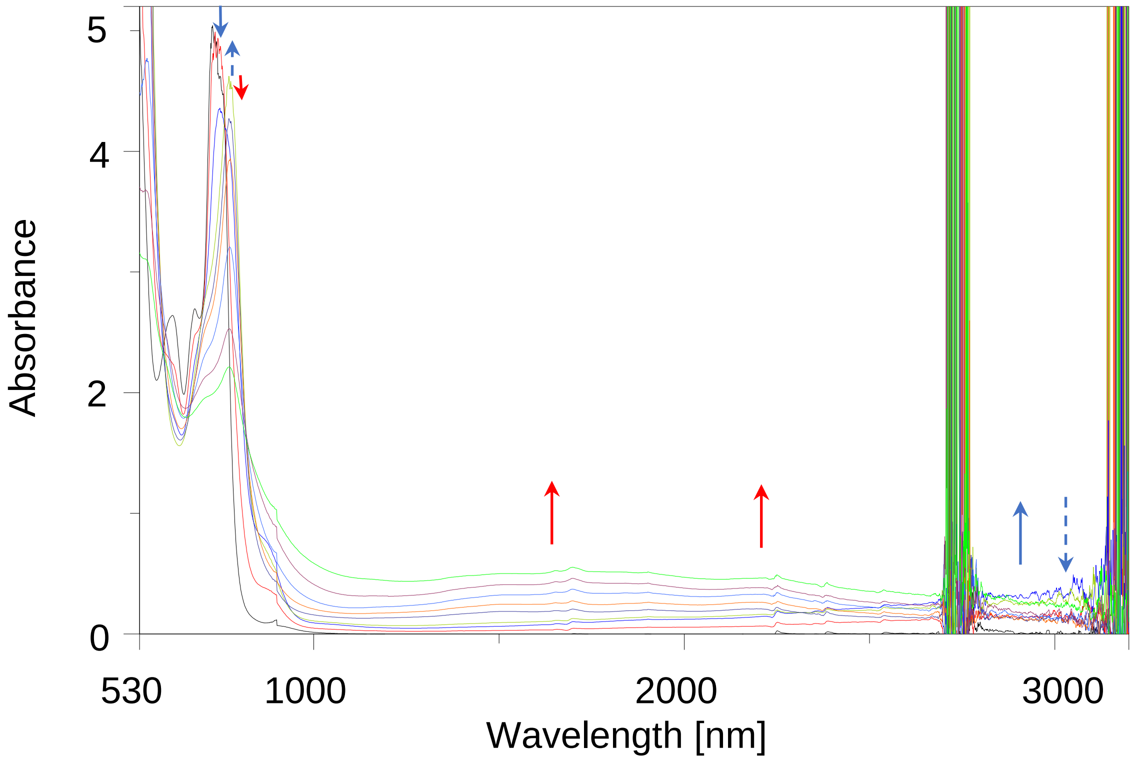

Figure S151. Electronic spectra (DCM) recorded upon titration of **5aa** with BAHA in DCM (0-4 equiv.).

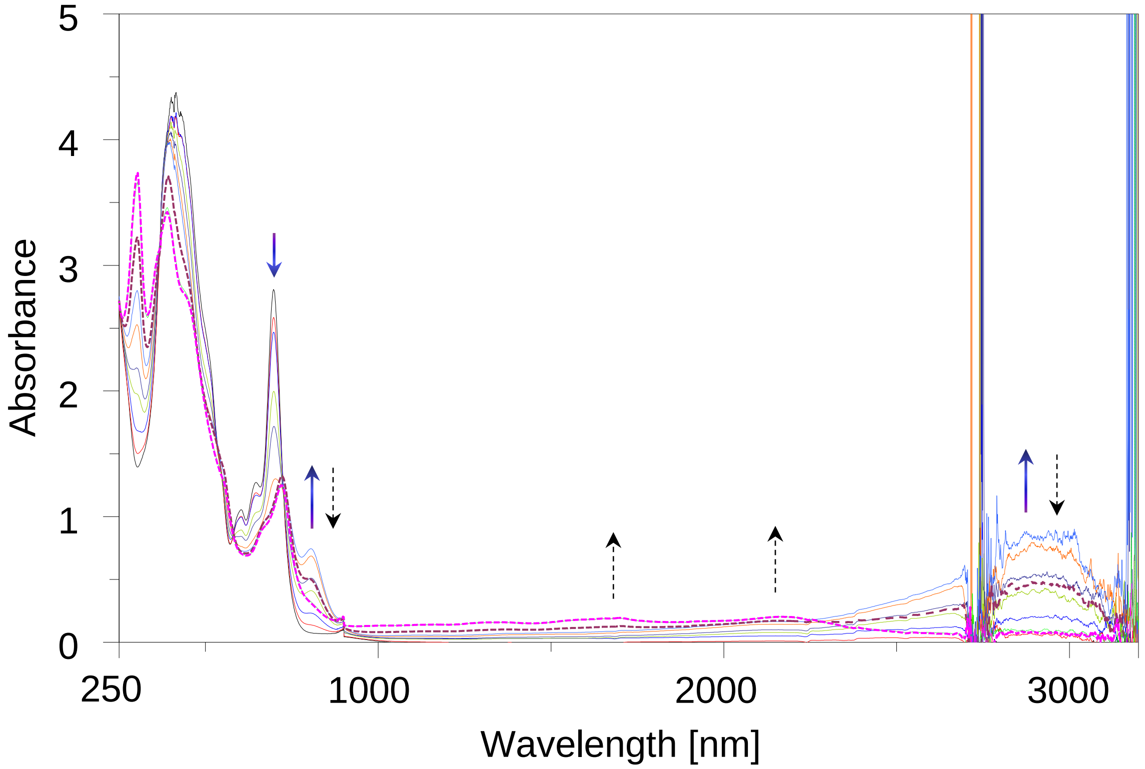

Figure S152. Electronic spectra (DCM) recorded upon titration of **5ab** with BAHA (0-2 equiv.).

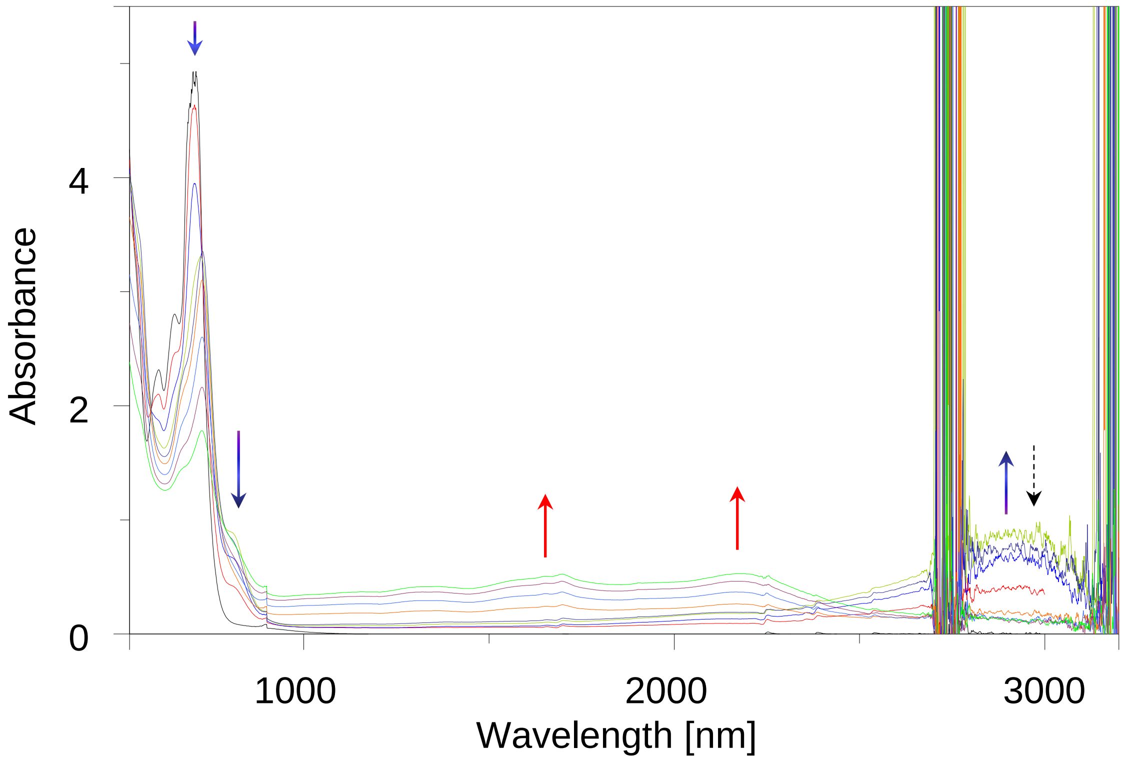

Figure S153. Electronic spectra (DCM) recorded upon titration of **5ab** with BAHA (0-4 equiv.).

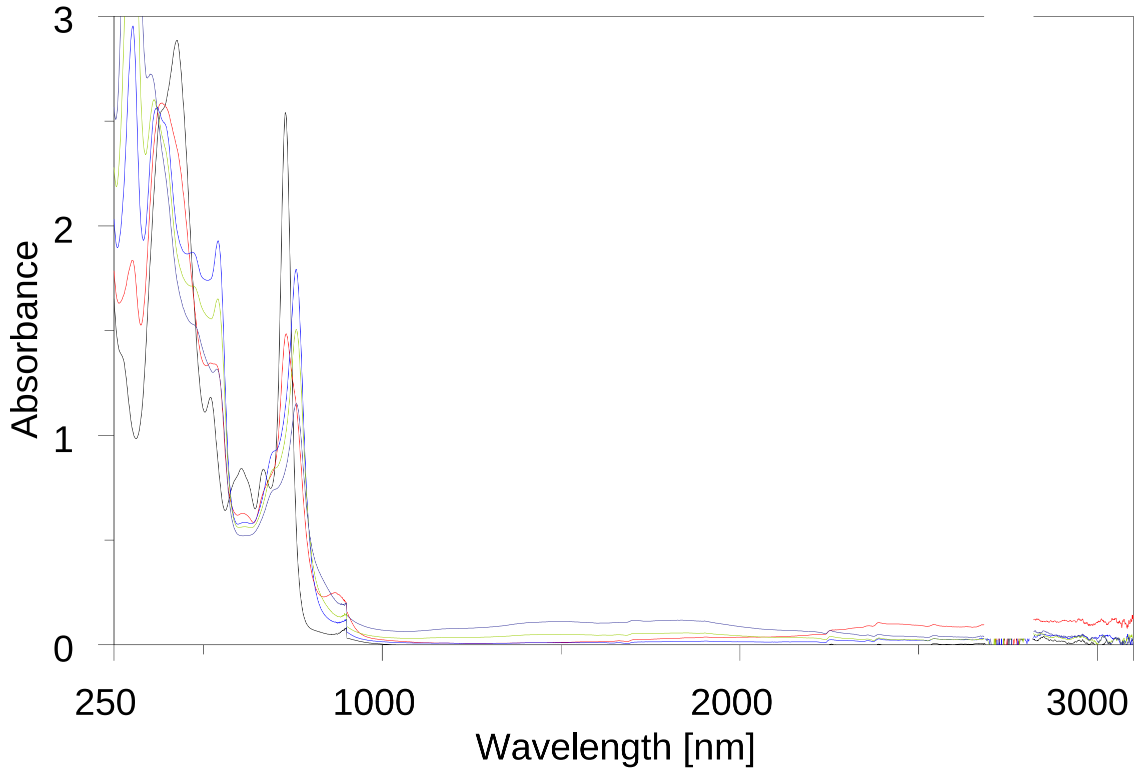

Figure S154. Selected electronic spectra (DCM) recorded upon titration of **6a** with BAHA: black, 0 equiv.; red, 1 equiv.; blue, 2 equiv.; green, 3 equiv.; navy, 4 equiv.

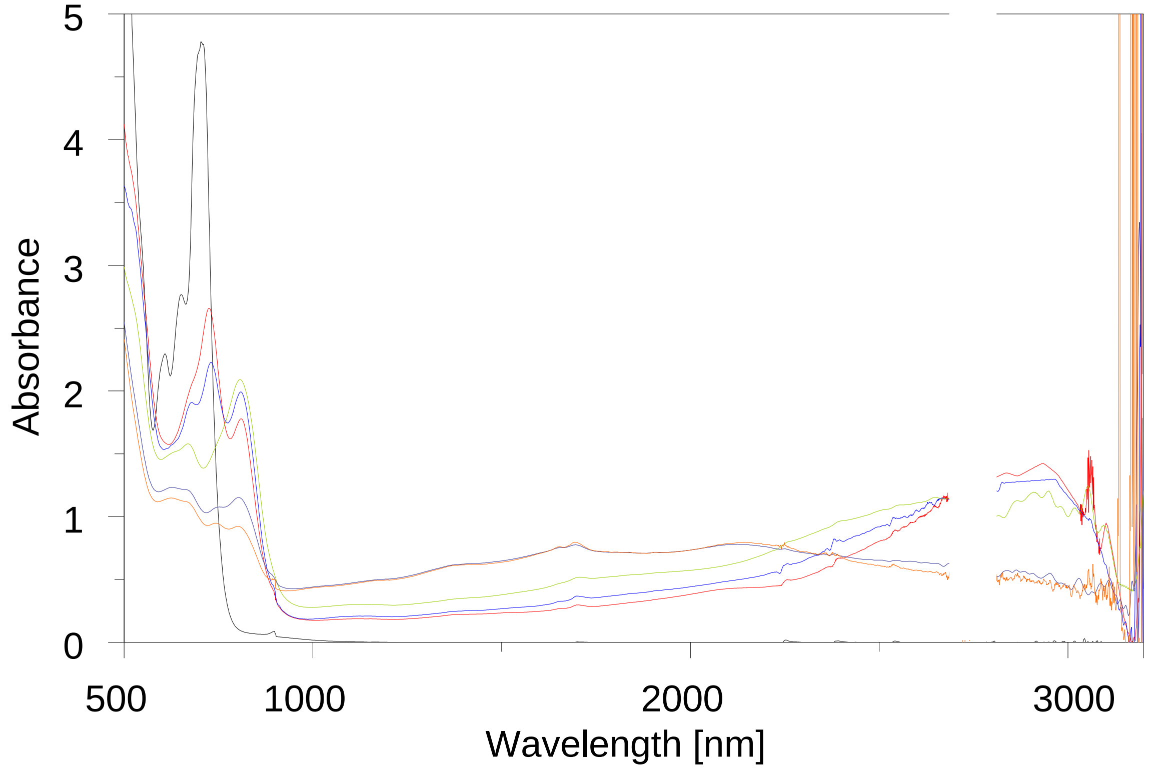

Figure S155. Selected electronic spectra (DCM) recorded upon titration of **5ab** with $AgBF_4$ (black, 0 equiv.; red, 1 equiv.; blue 1.5 equiv.; green, 2 equiv; navy, 3 equiv.; orange, 4 equiv.).

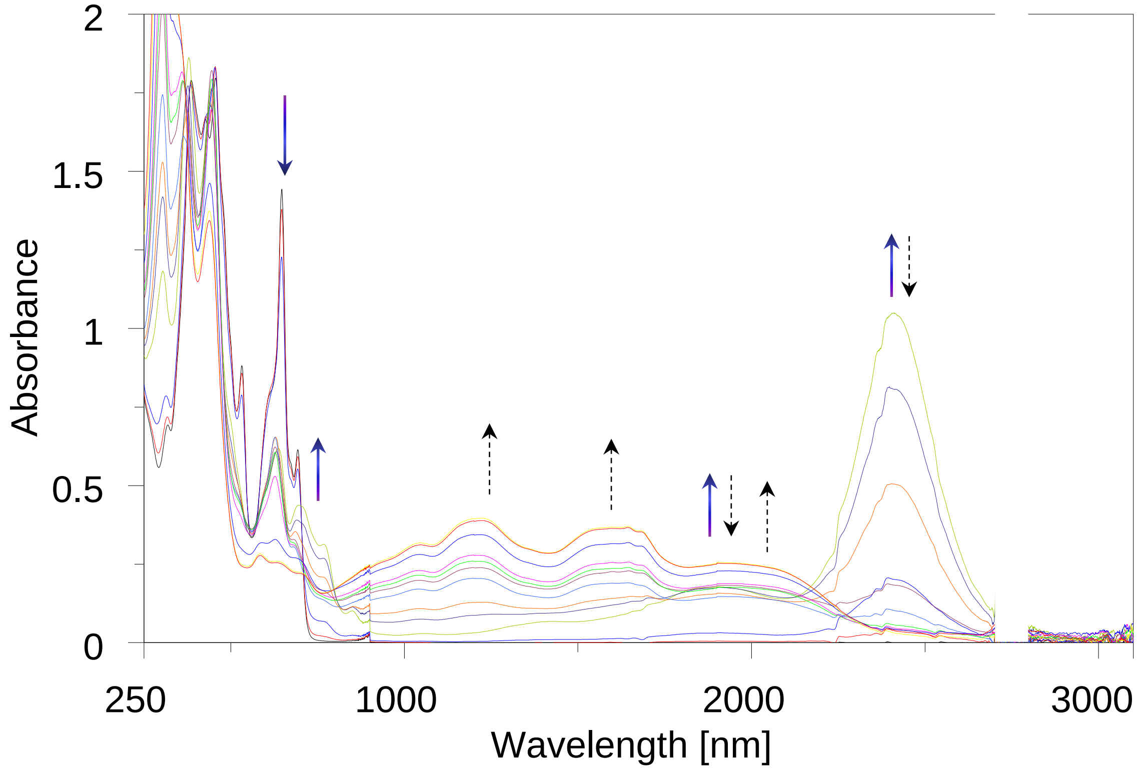

Figure S156. Electronic spectra (DCM) recorded upon titration of **5bb-1** with BAHA (0-4 equiv.).

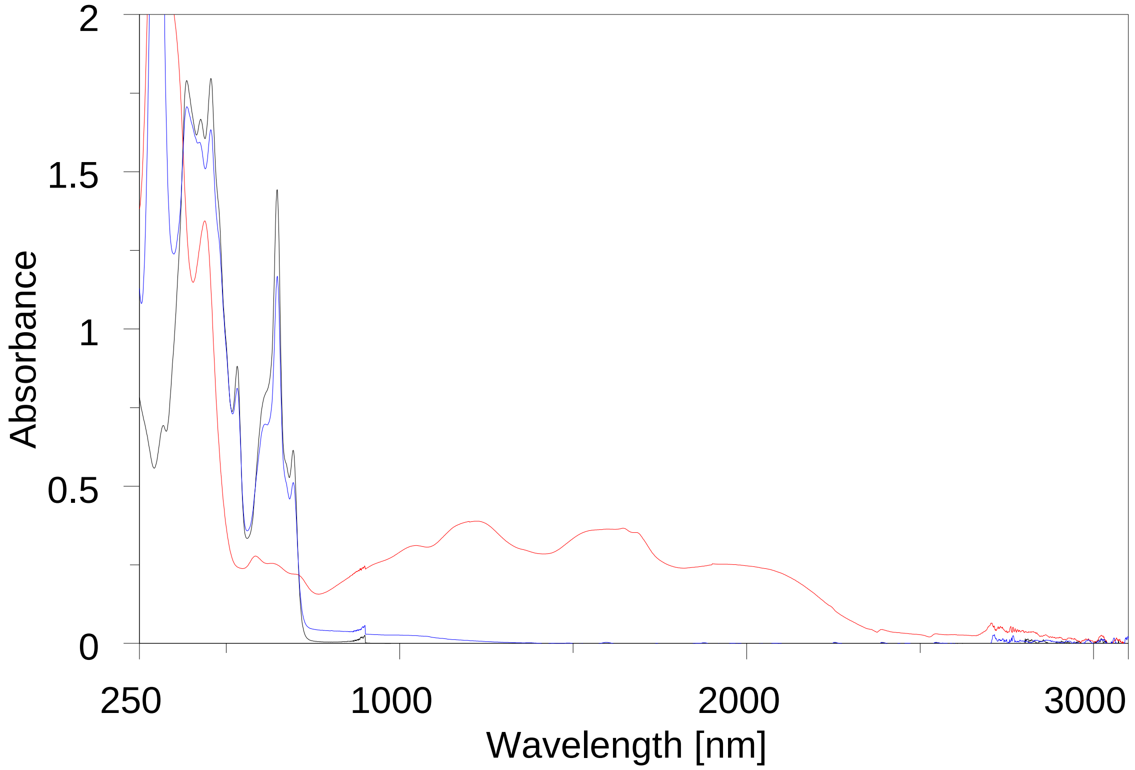

Figure S157. Electronic spectra (DCM) of **5bb-1** (black trace), after addition of 4 equiv. of BAHA oxidant (red trace) and after 3 h at room temperature and addition of solid KO_2 as a reductant (blue trace).

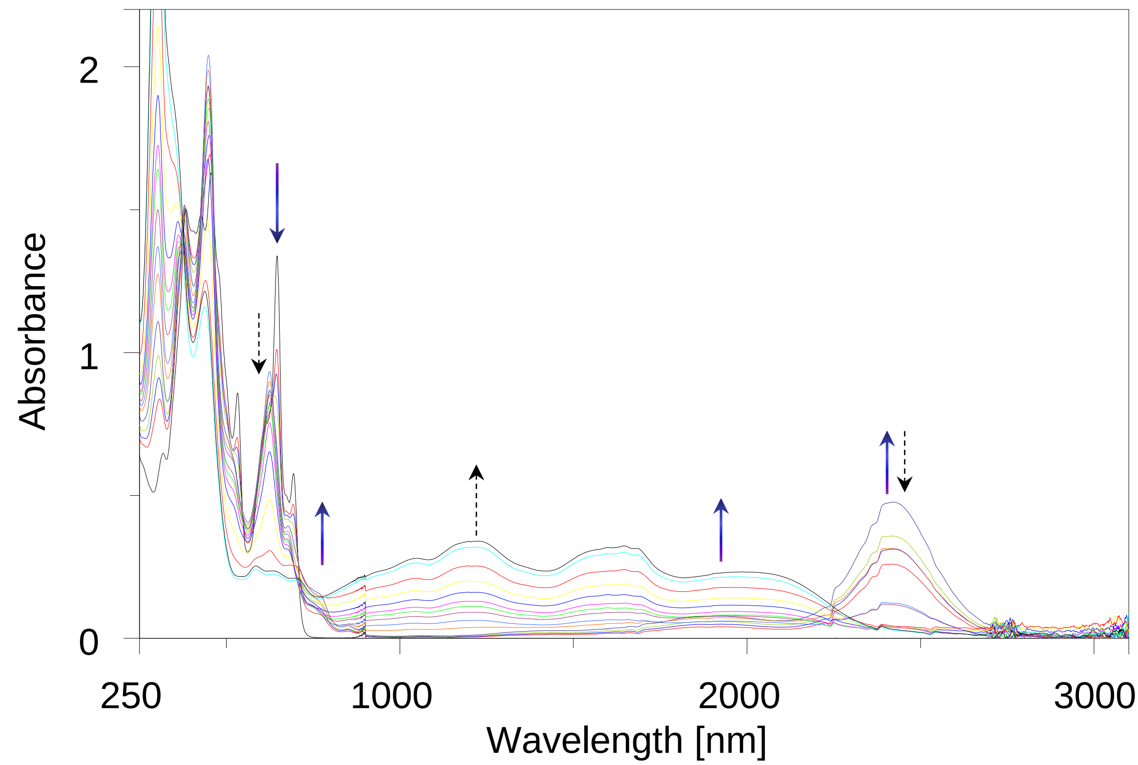

Figure S158. Electronic spectra (DCM) recorded upon titration of **5bb-3** with BAHA (0-3.5 equiv.).

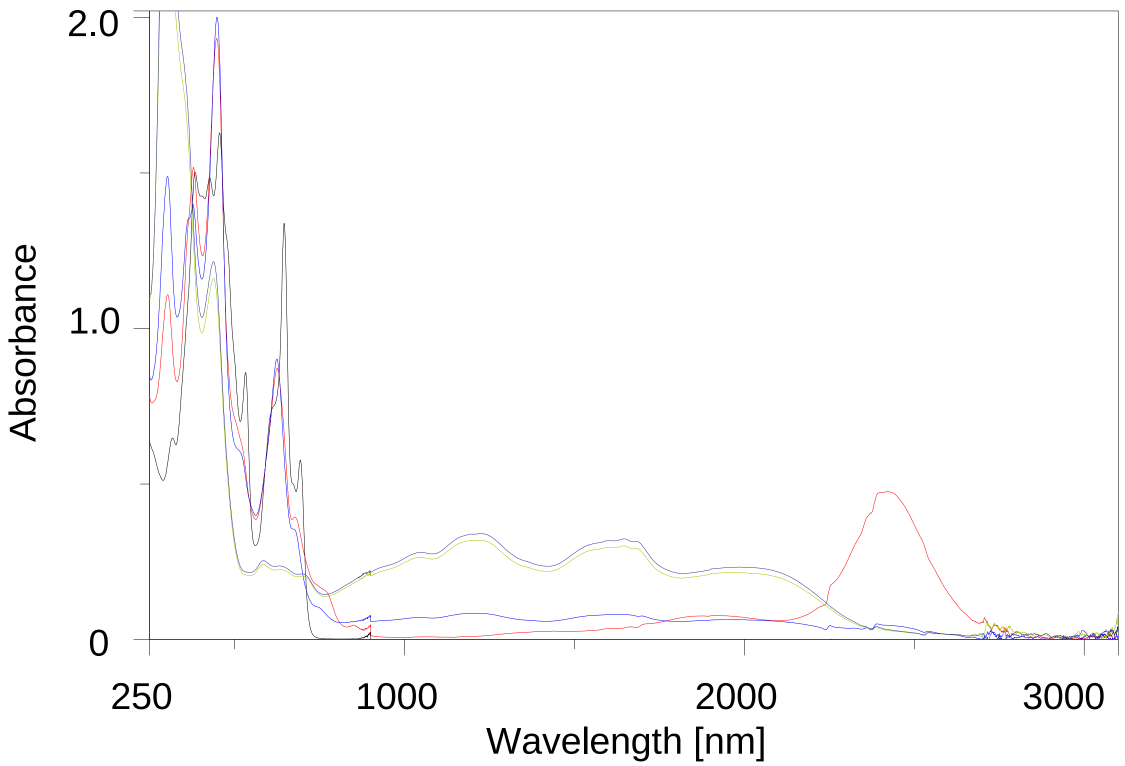

Figure S159. Electronic spectra (DCM) recorded upon titration of **5bb-3** with BAHA: 0 equiv. (black), 1 equiv. (red), 2 equiv (blue), 3.5 equiv (green), 3.5 equiv. after 16 h in solution at room temperature (navy).

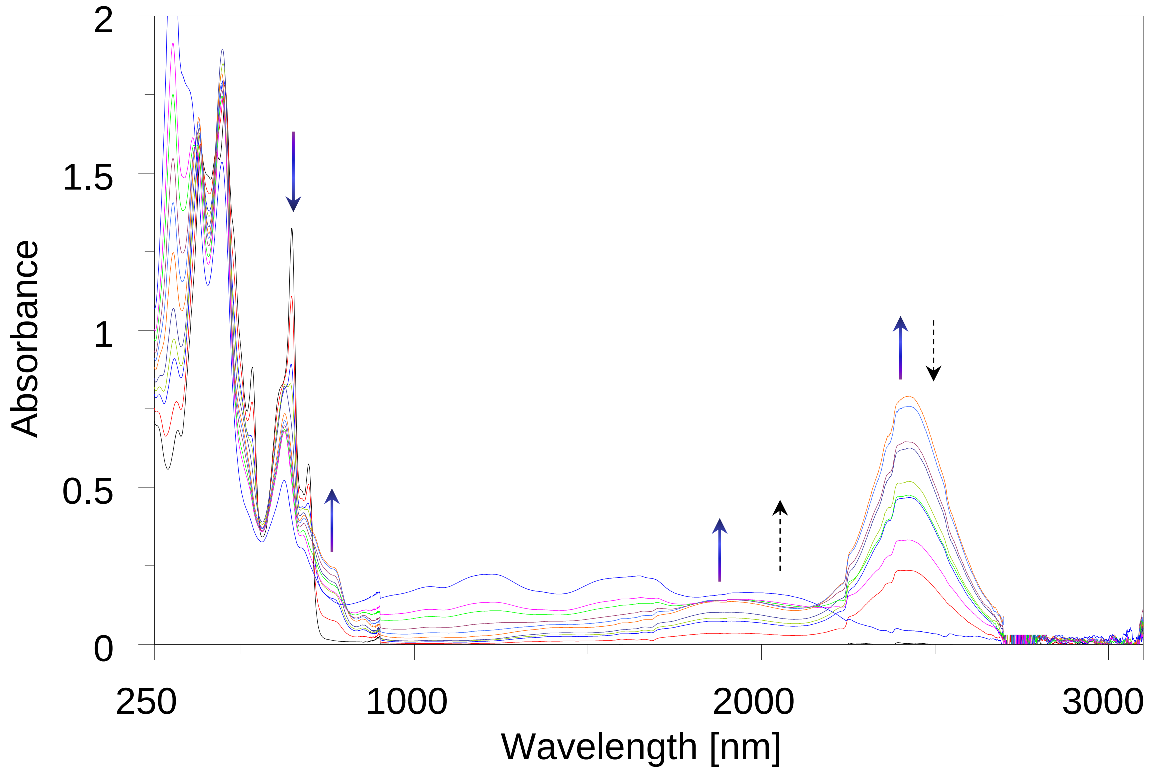

Figure S160. Electronic spectra (DCM) recorded upon titration of **5bb-4** with BAHA (0-2.5 equiv.).

18. DFT and TD DFT Calculation Results, part 2

Table S2. Computational details for the optimized structures of compounds

Structure / Name[a]	SCF E	ZPV[b]	lowest freq.	E	ΔH	ΔG[c]	ΔG_{rel}	–(HOMO)	–(LUMO)	HLG
	a.u.	a.u.	cm^{-1}	a.u.	a.u.	a.u.	kcal/ mol	eV	eV	eV
**5bb-5** /PJC_5_002	-5320.263957	-5318.877532	6.16	-5318.781390	-5318.780446	-5319.025439	0	4.72	2.82	1.90
**6b** /PJC_5_003	-4904.568585	-4903.277766	6.63	-4903.189250	-4903.188305	-4903.413609		4.77	2.83	1.94
**4b** /PJC_5_004	-4829.307775	-4828.023218	5.80	-4827.936235	-4827.935291	-4828.157710	0	4.67	3.30	1.37
**4a** /PJC_5_007	-4829.280735	-4827.996292	7.24	-4827.909612	-4827.908667	-4828.128756	18.2	4.75	3.37	1.38
**5ab** /PJC_5_001_1	-5320.237741	-5318.851501	4.44	-5318.756588	-5318.755644	-5318.995707	18.7	4.79	2.84	1.95
**2** /PJC_5_008	-2269.277584	-2268.610306	12.91	-2268.565929	-2268.564985	-2268.693210		5.03	2.82	2.21
**5aa** /PJC_5_005_UV	-5320.215084	-5318.828628	4.81	-5318.733265	-5318.732321	-5318.971626	33.8	4.86	2.86	2.00
**[5aa]**$^{+}$ /PJC_6_001_p_1_UV	-5320.036700	-5318.650942	6.85	-5318.555545	-5318.554601	-5318.793843		α 5.68<br>β 5.54	3.42<br>4.85	2.26<br>0.69
**s-[5aa]**$^{2+}$ /PJC_6_001_p_2_UV	-5319.828308	-5318.437110	7.27	-5318.343832	-5318.342887	-5318.571583	3.3	6.23	5.6	0.58
**[5ab]**$^{+}$ /PJC_6_002_p_1_UV	-5320.062203	-5318.675751	6.28	-5318.580152	-5318.579208	-5318.819544		α 5.62<br>β 5.58	3.32<br>4.77	2.30<br>0.81
**s-[5ab]**$^{2+}$ /PJC_6_002_p_2_UV	-5319.856276	-5318.466107	7.71	-5318.371965	-5318.371021	-5318.602022	2.4	6.24	5.66	0.58
**[4a]**$^{+}$ /PJC_6_003_p_1	-4829.106952	-4827.822048	7.82	-4827.734834	-4827.733890	-4827.954328		α 5.56<br>β 5.54	4.32<br>4.70	1.24<br>0.84
**s-[4a]**$^{2+}$ /PJC_6_003_p_2	-4828.901313	-4827.614212	7.39	-4827.527714	-4827.526770	-4827.743115	1.3	6.25	5.53	0.72
**d-[4a]**$^{3+}$ /PJC_6_003_p_3	-4828.673158	-4827.382778	12.50	-4827.298845	-4827.297901	-4827.503545		α 7.02<br>β 7.49	6.24<br>6.21	0.78<br>1.28
**t-[5aa]**$^{2+}$ /PJC_6_001_p_2_t_UV	-5319.834922	-5318.442812	7.96	-5318.349776	-5318.348831	-5318.576811	0	α 6.39<br>β 6.90	4.13<br>5.46	2.26<br>1.44
**t-[5ab]**$^{2+}$ /PJC_6_002_p_2_t_UV	-5319.858810	-5318.469063	7.87	-5318.374830	-5318.373886	-5318.605912	0	α 6.34<br>β 6.81	3.99<br>5.50	2.35<br>1.31
**t-[4a]**$^{2+}$ /PJC_6_003_p_2_t	-4828.904980	-4827.617569	10.20	-4827.531419	-4827.530474	-4827.745280	0	α 6.35<br>β 6.95	4.90<br>5.45	2.45<br>1.50
**t-[5bb-1]**$^{2+}$ /PJC_6_004_p_2_t	-4989.544756	-4988.105914	11.21	-4988.012426	-4988.011482	-4988.243198	0.2	α 6.16<br>β 6.67	3.82<br>5.43	2.34<br>1.24
**s-[5bb-1]**$^{2+}$ /PJC_6_004_p_2_UV	-4989.546640	-4988.106949	10.84	-4988.013522	-4988.012578	-4988.243467	0	6.21	5.39	0.82
**[5bb-1]**$^{+}$ /PJC_6_004_p_1	-4989.745703	-4988.308706	6.76	-4988.214433	-4988.213489	-4988.451389		α 5.44<br>β 5.50	3.08<br>4.57	2.36<br>0.93
**5bb-1** /PJC_6_004_0_UV	-4989.914067	-4988.477567	8.55	-4988.384821	-4988.383877	-4988.617208		4.59	2.30	2.29

[a] Data set name (Cartesian coordinated available as *.pdb files). [b] Zero-point vibrational energy. [c] Gibbs free energy

Table S3. NICS(*n*) indices (n = –1, 0, or 1) for **4a**. Blue numbers associate with the schematic structure denote GIAO-calculated chemical shifts of the pyrrole β-H

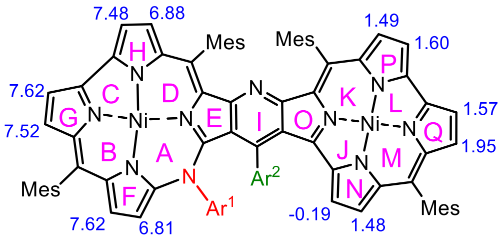

Ar[1] = 4-NO_2Ph, Ar[2] = 4-ClPh

**Ring**	NICS(1)	NICS(0)	NICS(-1)
**A**	-9.89	-14.09	-10.21
**B**	-8.24	-10.37	-7.95
**C**	-7.54	-11.83	-7.93
**D**	-8.27	-11.09	-8.84
**E**	-4.45	-2.19	-6.53
**F**	-9.88	-7.59	-8.19
**G**	-8.15	-7.49	-8.53
**I**	-6.89	-5.05	-8.10
**J**	35.61	40.68	33.66
**K**	33.59	39.13	30.83
**L**	35.88	39.45	31.77
**M**	32.56	40.07	33.01
**N**	0.64	2.97	3.80
**O**	-2.01	-2.15	-4.16
**P**	6.35	5.00	0.66
**Q**	5.00	6.17	3.90

Table S4. NICS(n) indices (n = –1, 0, or 1) for **4b**. Blue numbers associate with the schematic structure denote GIAO-calculated chemical shifts of the pyrrole β-H

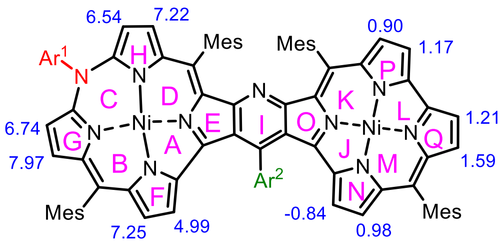

Ar^1 = 4-NO_2Ph, Ar^2 = 4-ClPh

Ring	NICS(1)	NICS(0)	NICS(-1)
**A**	-11.44	-15.24	-11.02
**B**	-11.16	-13.38	-10.83
**C**	-12.47	-16.76	-12.73
**D**	-10.62	-13.12	-11.19
**E**	-6.35	-3.39	-6.11
**F**	-10.28	-8.74	-9.78
**G**	-9.01	-7.10	-8.53
**H**	-9.25	-8.33	-11.06
**I**	-6.92	-4.51	-7.30
**J**	40.35	44.33	36.49
**K**	40.01	42.75	32.50
**L**	41.47	42.88	32.15
**M**	36.84	42.88	34.82
**N**	3.89	3.25	1.92
**O**	-1.79	-2.20	-4.46
**P**	5.44	6.83	-1.68
**Q**	6.62	7.39	2.65

Table S5. NICS(n) indices (n = –1, 0, or 1) for **5aa**. Blue numbers associate with the schematic structure denote GIAO-calculated chemical shifts of the pyrrole β-H

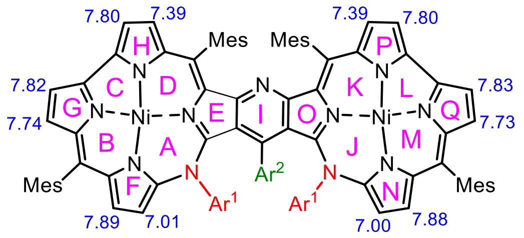

Ar^1 = 4-NO_2Ph, Ar^2 = 4-ClPh

Ring	NICS(1)	NICS(0)	NICS(-1)
**A**	-9.22	-14.08	-9.51
**B**	-7.91	-10.73	-8.37
**C**	-7.67	-11.94	-8.35
**D**	-7.88	-11.05	-8.82
**E**	-3.46	-0.98	-5.61
**F**	-10.16	-7.63	-8.16
**G**	-7.82	-7.10	-8.23
**H**	-8.25	-7.66	-9.26
**I**	-8.75	-6.46	-8.74
**J**	-9.36	-14.10	-9.40
**K**	-8.64	-11.06	-7.65
**L**	-7.37	-11.98	-7.08
**M**	-8.04	-10.77	-7.89
**N**	-7.87	-7.70	-9.93
**O**	-5.65	-1.02	-3.50
**P**	-8.84	-7.65	-7.86
**Q**	-8.18	-7.11	-7.79

Table S6. NICS(n) indices (n = −1, 0, or 1) for **5ab**. Blue numbers associate with the schematic structure denote GIAO-calculated chemical shifts of the pyrrole β-H

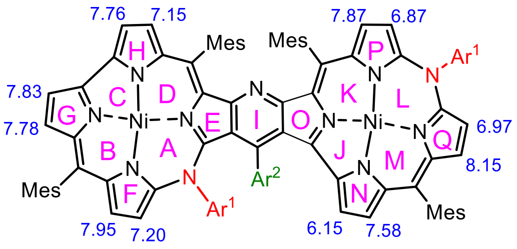

Ar^1 = 4-NO_2Ph, Ar^2 = 4-ClPh

**Ring**	NICS(1)	NICS(0)	NICS(-1)
**A**	-9.32	-14.15	-9.32
**B**	-8.18	-10.88	-7.94
**C**	-7.67	-12.18	-8.28
**D**	-7.79	-11.06	-8.59
**E**	-3.35	-1.24	-5.73
**F**	-9.10	-7.66	-7.45
**G**	-7.96	-7.12	-8.48
**H**	-8.02	-7.74	-8.92
**I**	-8.57	-6.78	-9.61
**J**	-9.40	-14.65	-10.92
**K**	-10.28	-12.67	-9.81
**L**	-12.12	-16.84	-12.08
**M**	-10.32	-13.42	-10.92
**N**	-8.43	-8.19	-9.95
**O**	-6.13	-3.22	-6.74
**P**	-9.98	-7.98	-8.28
**Q**	-7.91	-6.98	-8.65

Table S7. NICS(n) indices (n = –1, 0, or 1) for **5bb-5**. Blue numbers associate with the schematic structure denote GIAO-calculated chemical shifts of the pyrrole β-H

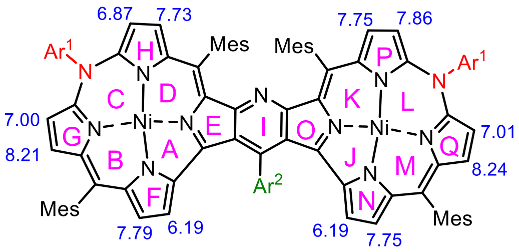

Ar[1] = 4-NO_2Ph, Ar[2] = 4-ClPh

Ring	NICS(1)	NICS(0)	NICS(-1)
**A**	-11.61	-14.56	-10.33
**B**	-11.89	-13.62	-11.02
**C**	-13.02	-17.10	-13.33
**D**	-10.35	-12.82	-11.44
**E**	-6.62	-3.30	-6.17
**F**	-10.55	-7.97	-9.34
**G**	-9.62	-6.94	-8.56
**H**	-9.51	-8.14	-12.03
**I**	-9.59	-7.43	-9.59
**J**	-10.39	-14.58	-11.59
**K**	-11.56	-12.87	-10.39
**L**	-13.40	-17.13	-13.02
**M**	-11.04	-13.65	-11.88
**N**	-9.34	-7.96	-10.48
**O**	-6.18	-3.29	-6.61
**P**	-12.22	-8.12	-9.53
**Q**	-8.59	-6.97	-9.63

Table S8. NICS(*n*) indices (n = –1, 0, or 1) for **6b**. Blue numbers associate with the schematic structure denote GIAO-calculated chemical shifts of the pyrrole β-H

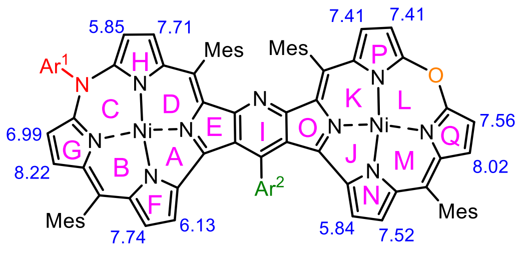

**Ring**	NICS(1)	NICS(0)	NICS(-1)
**A**	-11.41	-14.61	-10.22
**B**	-11.63	-13.59	-10.92
**C**	-12.90	-17.19	-13.22
**D**	-10.30	-12.84	-11.20
**E**	-6.54	-3.37	-6.17
**F**	-10.30	-8.02	-9.13
**G**	-9.10	-6.97	-8.58
**H**	-9.23	-8.18	-11.49
**I**	-9.59	-7.35	-9.54
**J**	-8.06	-11.49	-9.33
**K**	-9.38	-10.37	-8.51
**L**	-12.06	-15.59	-11.71
**M**	-9.11	-11.07	-9.77
**N**	-9.16	-7.55	-10.42
**O**	-6.40	-3.44	-6.64
**P**	-11.32	-7.90	-9.13
**Q**	-8.21	-6.59	-8.70

Table S9. NICS(*n*) indices (n = –1, 0, or 1) for **2**. Blue numbers associate with the schematic structure denote GIAO-calculated chemical shifts of the pyrrole β-H

2

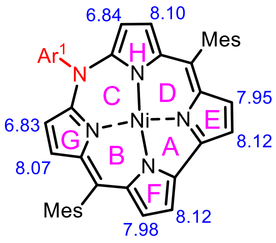

Ar^1 = 4-NO_2Ph

**Ring**	NICS(1)	NICS(0)	NICS(-1)
**A**	-9.73	-13.66	-9.73
**B**	-10.27	-12.73	-10.27
**C**	-12.19	-16.54	-12.19
**D**	-10.27	-12.73	-10.27
**E**	-8.58	-7.55	-8.58
**F**	-8.59	-7.56	-8.58
**G**	-8.34	-7.13	-8.34
**H**	-8.35	-7.14	-8.35

Table S10. NICS(n) indices (n = –1, 0, or 1) for s-[**5bb-1**]$^{2+}$. Blue numbers associate with the schematic structure denote GIAO-calculated chemical shifts of the pyrrole β-H

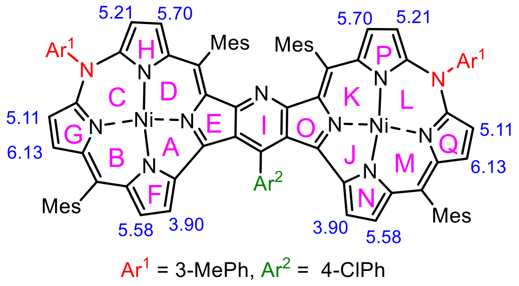

Ring	NICS(1)	NICS(0)	NICS(-1)
**A**	7.95	8.63	8.10
**B**	5.96	6.63	5.14
**C**	1.63	1.34	2.26
**D**	5.23	6.74	6.02
**E**	-1.91	1.92	-1.38
**F**	-9.35	-6.94	-8.38
**G**	-5.25	-2.28	-4.90
**H**	-4.46	-0.34	-5.80
**I**	-4.54	-1.62	-4.54
**J**	8.10	8.63	7.95
**K**	6.02	6.74	5.23
**L**	2.26	1.34	1.63
**M**	5.14	6.63	5.96
**N**	-8.38	-6.94	-9.35
**O**	-1.38	1.92	-1.91
**P**	-5.80	-0.34	-4.46
**Q**	-4.90	-2.28	-5.25

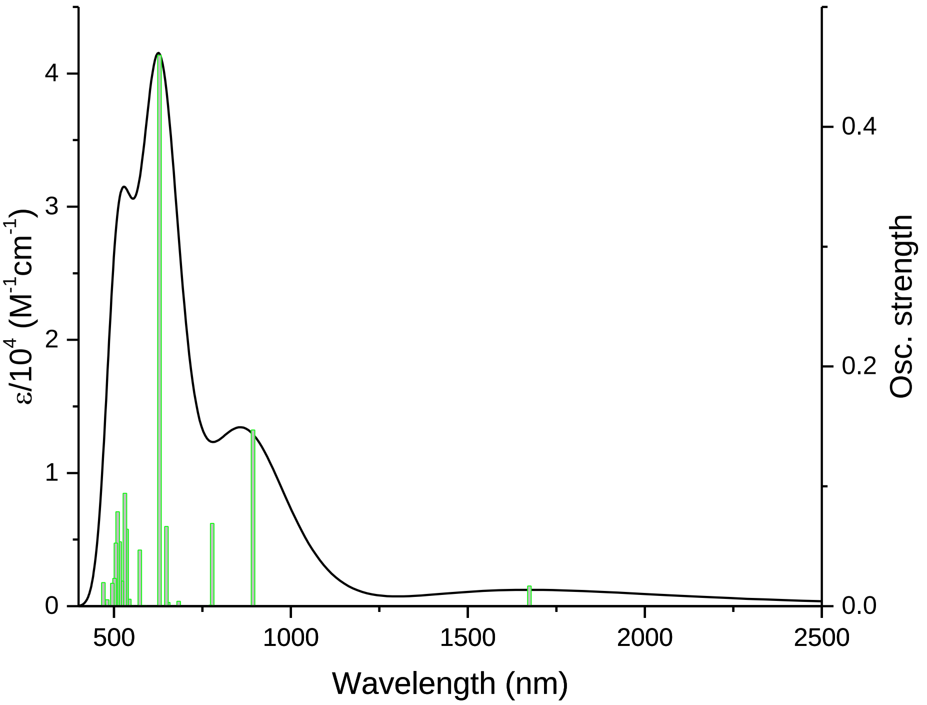

Figure S161. Calculated electronic transitions and vis-NIR spectrum for **4a**.

Table S11. Electronic transitions calculated for **4a**

No.	Energy (cm^{-1})	Wavelength (nm)	Osc. Strength	Symmetry	Major contributions
**1**	5974.15	1673.9	0.0169	Singlet-A	HOMO->LUMO (92%)
**2**	11201.43	892.7	0.147	Singlet-A	H-1->LUMO (90%)
**3**	12862.93	777.4	0.069	Singlet-A	HOMO->L+1 (97%)
**4**	12940.36	772.8	9E-4	Singlet-A	H-3->LUMO (75%), H-2->LUMO (20%)
**5**	14643.00	682.9	0.0041	Singlet-A	H-4->LUMO (45%), H-3->LUMO (10%), H-2->LUMO (36%)
**6**	15304.37	653.4	0.0031	Singlet-A	H-1->L+1 (81%)
**7**	15430.19	648.1	0.0665	Singlet-A	H-4->LUMO (40%), H-2->LUMO (31%), H-1->L+1 (17%)
**8**	15905.25	628.7	0.4597	Singlet-A	HOMO->L+2 (88%)
**9**	17069.11	585.9	7E-4	Singlet-A	H-11->LUMO (98%)
**10**	17442.55	573.3	0.0468	Singlet-A	HOMO->L+3 (78%)
**11**	18176.51	550.2	7E-4	Singlet-A	H-2->L+1 (15%), H-1->L+2 (74%)
**12**	18400.73	543.5	0.0058	Singlet-A	H-5->LUMO (80%)
**13**	18673.35	535.5	0.0642	Singlet-A	H-2->L+1 (53%)
**14**	18841.11	530.8	0.0942	Singlet-A	H-9->LUMO (13%), H-8->LUMO (27%), H-7->LUMO (19%), H-6->LUMO (22%)
**15**	19145.18	522.3	0.021	Singlet-A	H-9->LUMO (10%), H-8->LUMO (25%), H-7->LUMO (54%)
**16**	19375.05	516.1	0.0538	Singlet-A	HOMO->L+4 (54%)
**17**	19595.24	510.3	0.0788	Singlet-A	H-6->LUMO (24%), H-1->L+3 (34%)
**18**	19777.52	505.6	0.0526	Singlet-A	H-6->LUMO (25%), H-1->L+6 (11%)
**19**	19921.89	502.0	0.0232	Singlet-A	H-1->L+3 (26%), H-1->L+6 (13%), HOMO->L+4 (10%)
**20**	20178.38	495.6	0.0191	Singlet-A	H-12->LUMO (94%)
**21**	20307.43	492.4	4E-4	Singlet-A	H-16->LUMO (30%), H-9->LUMO (41%), H-8->LUMO (13%)
**22**	20794.59	480.9	0.005	Singlet-A	H-17->L+6 (78%)
**23**	20813.14	480.5	0.0054	Singlet-A	H-3->L+1 (95%)
**24**	21205.93	471.6	0.0022	Singlet-A	H-15->LUMO (95%)
**25**	21276.91	470.0	0.0197	Singlet-A	H-16->LUMO (46%), H-9->LUMO (21%), H-8->LUMO (13%)

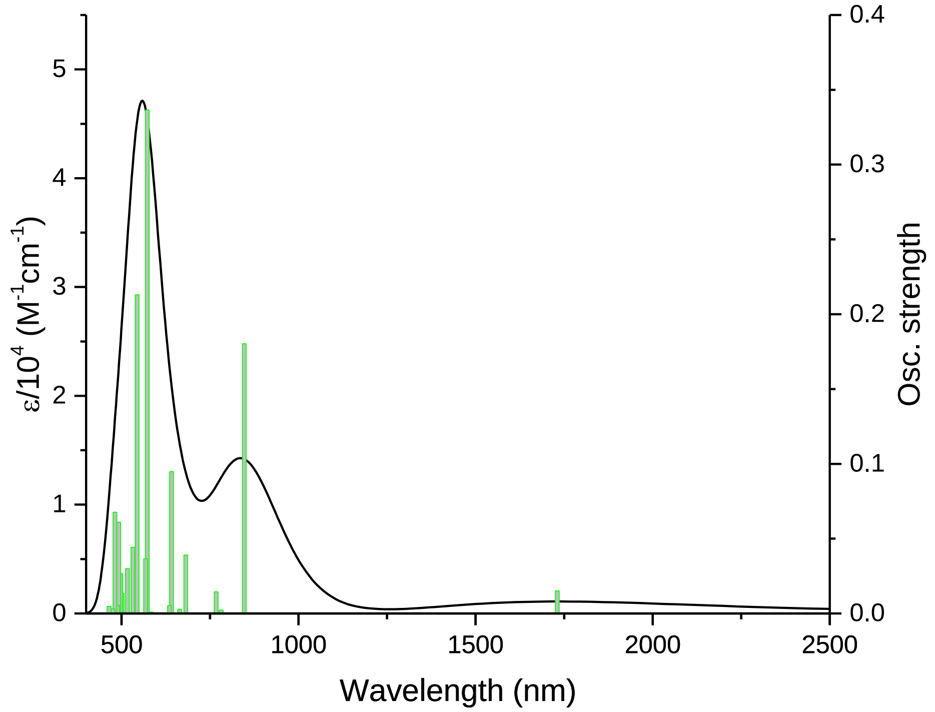

Figure S162. Calculated electronic transitions and vis-NIR spectrum for **4b**.

Table S12. Electronic transitions calculated for **4b**

No.	Energy (cm^{-1})	Wavelength (nm)	Osc. Strength	Symmetry	Major contributions
**1**	5778.16	1730.7	0.0152	Singlet-A	HOMO->LUMO (95%)
**2**	11811.18	846.7	0.1803	Singlet-A	H-1->LUMO (87%)
**3**	12798.41	781.3	0.0023	Singlet-A	HOMO->L+1 (95%)
**4**	13035.53	767.1	0.0145	Singlet-A	H-3->LUMO (86%)
**5**	14676.87	681.3	0.0391	Singlet-A	H-4->LUMO (22%), H-2->LUMO (70%)
**6**	15056.76	664.2	0.0028	Singlet-A	H-4->LUMO (71%), H-2->LUMO (23%)
**7**	15594.73	641.2	0.0949	Singlet-A	HOMO->L+2 (90%)
**8**	15733.46	635.6	0.0053	Singlet-A	H-1->L+1 (89%)
**9**	17101.37	584.7	9E-4	Singlet-A	H-10->LUMO (82%), H-9->LUMO (17%)
**10**	17461.10	572.7	0.3365	Singlet-A	HOMO->L+3 (86%)
**11**	17599.02	568.2	0.0366	Singlet-A	H-2->L+1 (95%)
**12**	18378.96	544.1	0.213	Singlet-A	H-1->L+2 (87%)
**13**	18789.49	532.2	0.0442	Singlet-A	H-7->LUMO (28%), H-6->LUMO (10%), H-5->LUMO (48%)
**14**	19342.79	517.0	0.03	Singlet-A	H-8->LUMO (27%), H-7->LUMO (32%), H-5->LUMO (39%)
**15**	19824.30	504.4	9E-4	Singlet-A	H-6->L+7 (13%), H-1->L+7 (19%), HOMO->L+7 (10%)
**16**	19918.67	502.0	0.0133	Singlet-A	H-8->LUMO (11%), H-2->L+2 (20%), H-1->L+3 (32%)
**17**	20100.14	497.5	0.0267	Singlet-A	H-8->LUMO (42%), H-7->LUMO (18%), H-1->L+3 (11%)
**18**	20328.40	491.9	0.061	Singlet-A	H-16->LUMO (14%), H-10->LUMO (11%), H-9->LUMO (50%)
**19**	20402.60	490.1	1E-4	Singlet-A	H-12->LUMO (13%), H-6->LUMO (58%)
**20**	20429.22	489.5	0.0053	Singlet-A	H-12->LUMO (77%)
**21**	20781.68	481.2	0.0677	Singlet-A	HOMO->L+4 (76%)
**22**	21013.16	475.9	0.003	Singlet-A	H-17->L+6 (21%), H-17->L+7 (57%)
**23**	21221.25	471.2	1E-4	Singlet-A	H-3->L+1 (99%)
**24**	21512.42	464.8	0.0047	Singlet-A	H-15->LUMO (98%)
**25**	21832.62	458.0	2E-4	Singlet-A	H-13->LUMO (87%)

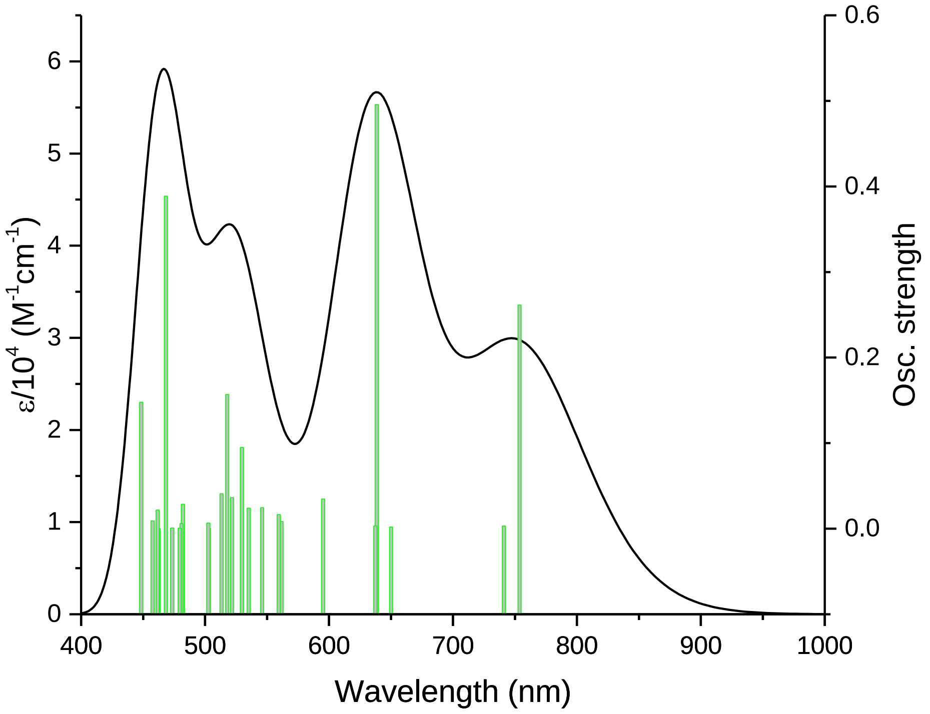

Figure S163. Calculated electronic transitions and vis-NIR spectrum for **5aa**.

Table S13. Electronic transitions calculated for **5aa**

No.	Energy (cm^{-1})	Wavelength (nm)	Osc. Strength	Symmetry	Major contributions
**1**	13267.01	753.7	0.2612	Singlet-A	HOMO->LUMO (98%)
**2**	13493.66	741.1	0.0031	Singlet-A	HOMO->L+1 (99%)
**3**	15382.61	650.1	0.0021	Singlet-A	H-1->LUMO (97%)
**4**	15662.48	638.5	0.4957	Singlet-A	HOMO->L+2 (96%)
**5**	15687.48	637.5	0.0032	Singlet-A	H-1->L+1 (97%)
**6**	16798.92	595.3	0.0346	Singlet-A	HOMO->L+3 (85%)
**7**	17804.69	561.6	0.0084	Singlet-A	H-3->LUMO (10%), H-2->L+1 (14%), H-1->L+2 (59%)
**8**	17874.05	559.5	0.0164	Singlet-A	H-2->LUMO (67%), HOMO->L+5 (16%)
**9**	18311.21	546.1	0.0245	Singlet-A	H-2->L+1 (38%), H-1->L+2 (33%), HOMO->L+4 (22%)
**10**	18683.83	535.2	0.0239	Singlet-A	H-3->LUMO (11%), H-2->L+1 (46%), HOMO->L+4 (34%)
**11**	18876.60	529.8	0.0949	Singlet-A	H-2->LUMO (20%), H-1->L+3 (50%)
**12**	19164.54	521.8	0.0363	Singlet-A	H-3->L+1 (87%)
**13**	19308.91	517.9	0.1569	Singlet-A	H-3->LUMO (71%), HOMO->L+4 (16%)
**14**	19482.32	513.3	0.0408	Singlet-A	H-2->L+2 (10%), H-1->L+3 (21%), HOMO->L+5 (49%)
**15**	19875.92	503.1	4E-4	Singlet-A	H-6->L+7 (13%), H-4->L+8 (10%), H-1->L+7 (19%), HOMO->L+8 (10%)
**16**	19895.28	502.6	0.0066	Singlet-A	H-4->L+7 (12%), H-1->L+8 (11%), HOMO->L+7 (13%)
**17**	20738.93	482.2	0.0286	Singlet-A	H-17->L+8 (24%), H-17->L+9 (10%), H-16->L+7 (33%), H-1->L+4 (11%)
**18**	20790.55	481.0	0.0059	Singlet-A	H-2->L+2 (17%), H-1->L+4 (60%)
**19**	20851.85	479.6	6E-4	Singlet-A	H-17->L+7 (44%), H-16->L+8 (31%), H-16->L+9 (13%)
**20**	21118.82	473.5	9E-4	Singlet-A	H-3->L+2 (26%), H-1->L+5 (60%)
**21**	21349.50	468.4	0.3887	Singlet-A	H-2->L+2 (47%)
**22**	21620.50	462.5	3E-4	Singlet-A	H-10->L+8 (16%), H-9->L+7 (12%), H-5->L+7 (11%)
**23**	21656.79	461.7	0.0219	Singlet-A	H-10->L+7 (24%)
**24**	21848.75	457.7	0.0092	Singlet-A	H-3->L+2 (13%), H-2->L+3 (55%)
**25**	22294.78	448.5	0.1478	Singlet-A	H-6->LUMO (10%), H-3->L+2 (35%), H-2->L+3 (14%), H-1->L+5 (19%)

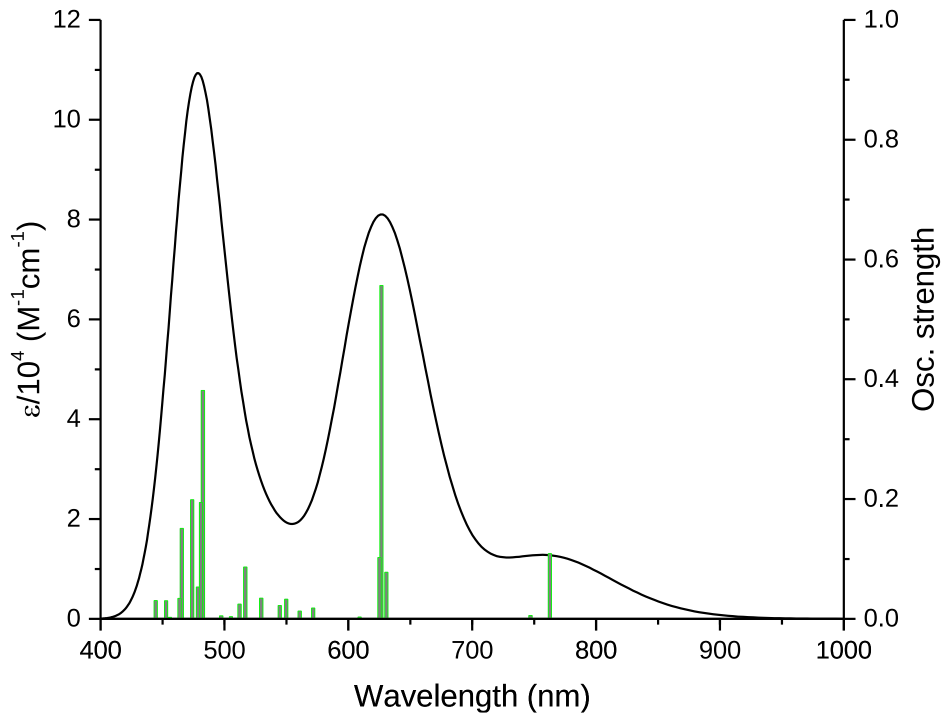

Figure S164. Calculated electronic transitions and vis-NIR spectrum for **5ab**.

Table S14. Electronic transitions calculated for **5ab**

No.	Energy (cm^{-1})	Wavelength (nm)	Osc. Strength	Symmetry	Major contributions
**1**	13110.54	762.7	0.109	Singlet-A	HOMO->L+1 (99%)
**2**	13385.58	747.1	0.0057	Singlet-A	HOMO->LUMO (93%)
**3**	15855.25	630.7	0.0779	Singlet-A	H-1->L+1 (72%), HOMO->L+2 (24%)
**4**	15955.26	626.8	0.5565	Singlet-A	H-1->L+1 (15%), HOMO->L+2 (29%), HOMO->L+3 (51%)
**5**	15995.59	625.2	0.1024	Singlet-A	H-1->L+1 (11%), HOMO->L+2 (37%), HOMO->L+3 (43%)
**6**	16418.22	609.1	0.0032	Singlet-A	H-1->LUMO (86%)
**7**	17494.97	571.6	0.0182	Singlet-A	H-2->LUMO (83%)
**8**	17833.72	560.7	0.0129	Singlet-A	H-2->L+1 (61%), HOMO->L+4 (22%)
**9**	18187.00	549.8	0.0327	Singlet-A	H-2->L+1 (27%), H-1->L+2 (23%), H-1->L+3 (10%), HOMO->L+4 (27%)
**10**	18361.21	544.6	0.0224	Singlet-A	H-1->L+2 (50%), H-1->L+3 (11%), HOMO->L+4 (14%)
**11**	18878.21	529.7	0.0346	Singlet-A	H-3->L+1 (26%), H-1->L+3 (41%)
**12**	19348.43	516.8	0.0866	Singlet-A	H-3->L+1 (52%), H-2->L+2 (11%), HOMO->L+4 (13%), HOMO->L+5 (11%)
**13**	19524.26	512.2	0.0246	Singlet-A	H-1->L+3 (20%), HOMO->L+5 (50%)
**14**	19791.23	505.3	0.0037	Singlet-A	--
**15**	19816.24	504.6	5E-4	Singlet-A	H-8->L+7 (10%), H-8->L+9 (10%), HOMO->L+7 (12%), HOMO->L+9 (10%)
**16**	20104.98	497.4	0.0052	Singlet-A	H-3->LUMO (89%)
**17**	20719.58	482.6	0.3815	Singlet-A	H-16->L+7 (13%), H-16->L+9 (11%), H-2->L+2 (23%), H-1->L+4 (15%)
**18**	20786.52	481.1	0.1943	Singlet-A	H-16->L+7 (17%), H-16->L+9 (15%), H-16->L+10 (10%), H-2->L+3 (17%), H-1->L+4 (18%)
**19**	20890.57	478.7	0.0533	Singlet-A	H-17->L+7 (24%), H-17->L+9 (22%), H-2->L+2 (10%), H-2->L+3 (12%)
**20**	21099.46	473.9	0.1989	Singlet-A	H-17->L+7 (18%), H-17->L+9 (17%), H-2->L+2 (24%), H-1->L+4 (16%)
**21**	21476.93	465.6	0.151	Singlet-A	H-2->L+3 (43%), H-1->L+4 (19%)
**22**	21564.04	463.7	0.0342	Singlet-A	--
**23**	21926.99	456.1	0.0026	Singlet-A	H-7->L+7 (14%), H-7->L+9 (14%)
**24**	22081.04	452.9	0.0303	Singlet-A	H-3->L+2 (59%), H-1->L+5 (12%), HOMO->L+6 (10%)
**25**	22494.80	444.5	0.0307	Singlet-A	H-4->L+1 (14%), H-3->L+3 (42%), H-1->L+5 (16%)

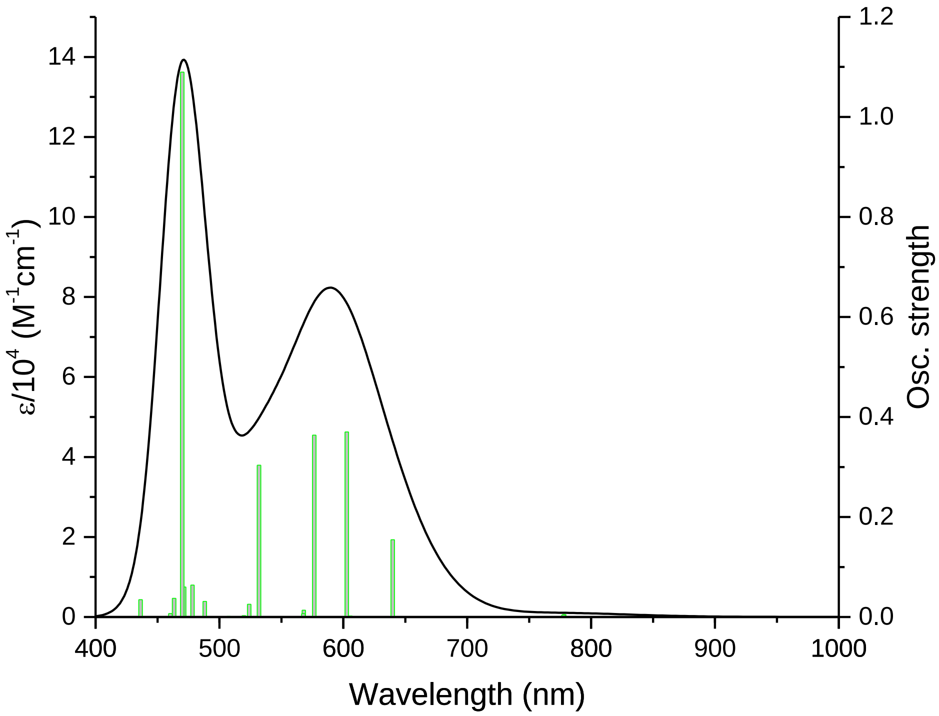

Figure S165. Calculated electronic transitions and UV-vis spectrum for **5bb-5**.

Table S15. Electronic transitions calculated for **5bb-5**

No.	Energy (cm^{-1})	Wavelength (nm)	Osc. Strength	Symmetry	Major contribs
**1**	12848.41	778.3	0.0048	Singlet-A	HOMO->LUMO (92%)
**2**	12857.28	777.8	0.0041	Singlet-A	HOMO->L+1 (92%)
**3**	15627.80	639.9	0.1545	Singlet-A	HOMO->L+2 (94%)
**4**	16505.33	605.9	0.0019	Singlet-A	H-2->L+1 (15%), H-1->LUMO (63%)
**5**	16519.85	605.3	0	Singlet-A	H-2->LUMO (17%), H-1->L+1 (68%)
**6**	16590.02	602.8	0.3702	Singlet-A	HOMO->L+3 (78%)
**7**	17344.95	576.5	0.3636	Singlet-A	HOMO->L+4 (84%)
**8**	17601.44	568.1	0.0136	Singlet-A	H-3->LUMO (19%), H-2->L+1 (51%), H-1->LUMO (12%)
**9**	17611.92	567.8	0.0069	Singlet-A	H-3->L+1 (19%), H-2->LUMO (50%), H-1->L+1 (13%)
**10**	18799.17	531.9	0.3037	Singlet-A	H-1->L+2 (88%)
**11**	19083.88	524.0	0.0254	Singlet-A	H-2->L+2 (28%), H-1->L+3 (44%), HOMO->L+5 (11%)
**12**	19238.74	519.8	0.0026	Singlet-A	H-3->LUMO (66%), H-2->L+1 (24%)
**13**	19254.87	519.3	0.0018	Singlet-A	H-3->L+1 (61%), H-2->LUMO (22%)
**14**	19704.12	507.5	0.0014	Singlet-A	H-1->L+10 (13%), HOMO->L+9 (22%)
**15**	19754.13	506.2	0	Singlet-A	H-1->L+9 (11%), HOMO->L+10 (25%)
**16**	20486.48	488.1	0.0312	Singlet-A	H-2->L+2 (17%), H-1->L+3 (38%), H-1->L+4 (15%), HOMO->L+5 (19%)
**17**	20909.92	478.2	0.0637	Singlet-A	H-17->L+10 (38%), H-16->L+9 (31%), H-2->L+3 (11%)
**18**	20973.64	476.8	6E-4	Singlet-A	H-17->L+9 (37%), H-16->L+10 (46%)
**19**	21212.38	471.4	0.0602	Singlet-A	H-1->L+4 (58%), HOMO->L+5 (24%)
**20**	21275.29	470.0	1.0895	Singlet-A	H-3->L+2 (36%), H-2->L+3 (35%)
**21**	21580.17	463.4	0.0373	Singlet-A	H-3->L+3 (25%), H-2->L+2 (39%), HOMO->L+5 (28%)
**22**	21718.90	460.4	0.0068	Singlet-A	H-3->L+2 (50%), H-2->L+3 (43%)
**23**	21910.86	456.4	1E-4	Singlet-A	H-15->L+10 (12%), H-6->L+10 (12%), H-5->L+9 (17%)
**24**	21931.83	456.0	9E-4	Singlet-A	H-15->L+9 (10%), H-7->L+10 (10%), H-5->L+10 (20%)
**25**	22919.05	436.3	0.0347	Singlet-A	H-2->L+4 (60%)

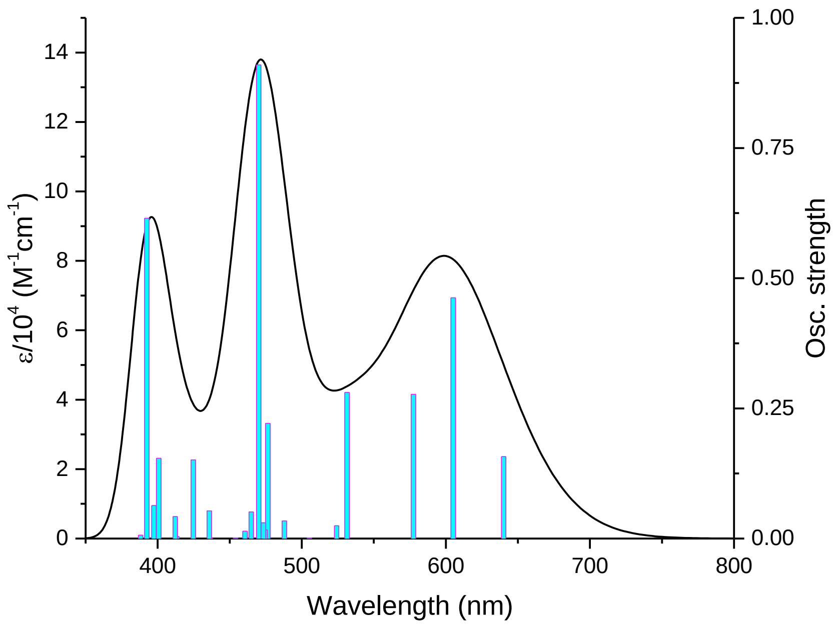

Figure S166. Calculated electronic transitions and UV-vis spectrum for **5bb-1**.

Table S16. Electronic transitions calculated for **5bb-1**

No.	Energy (cm^{-1})	Wavelength (nm)	Osc. Strength	Symmetry	Major contributions
**1**	15621.35	640.1	0.1572	Singlet-A	HOMO->LUMO (94%)
**2**	16526.30	605.1	0.4624	Singlet-A	HOMO->L+1 (80%), HOMO->L+2 (10%)
**3**	17315.92	577.5	0.2771	Singlet-A	HOMO->L+1 (10%), HOMO->L+2 (82%)
**4**	18815.30	531.5	0.2806	Singlet-A	H-1->LUMO (89%)
**5**	19075.82	524.2	0.0247	Singlet-A	H-2->LUMO (30%), H-1->L+1 (47%), HOMO->L+3 (14%)
**6**	19790.43	505.3	0.0012	Singlet-A	H-1->L+6 (13%), HOMO->L+5 (29%)
**7**	19838.82	504.1	0	Singlet-A	H-5->L+5 (10%), H-1->L+5 (14%), HOMO->L+6 (26%)
**8**	20492.93	488.0	0.034	Singlet-A	H-2->LUMO (12%), H-1->L+1 (38%), H-1->L+2 (13%), HOMO->L+3 (24%)
**9**	20988.16	476.5	0.2214	Singlet-A	H-17->L+6 (29%), H-16->L+5 (31%), H-2->L+1 (22%)
**10**	21072.85	474.5	0.0168	Singlet-A	H-17->L+5 (26%), H-16->L+6 (26%), H-1->L+2 (25%), HOMO->L+3 (12%)
**11**	21123.66	473.4	0.0302	Singlet-A	H-17->L+5 (21%), H-16->L+6 (20%), H-1->L+2 (36%), HOMO->L+3 (12%)
**12**	21270.45	470.1	0.9098	Singlet-A	H-17->L+6 (15%), H-16->L+5 (16%), H-3->LUMO (35%), H-2->L+1 (22%)
**13**	21509.19	464.9	0.0513	Singlet-A	H-3->L+1 (23%), H-2->LUMO (43%), HOMO->L+3 (22%)
**14**	21709.22	460.6	0.0142	Singlet-A	H-3->LUMO (46%), H-2->L+1 (46%)
**15**	21999.58	454.6	0	Singlet-A	H-35->L+5 (10%), H-12->L+6 (12%), H-6->L+5 (14%), H-5->L+5 (22%), H-4->L+6 (11%)
**16**	22020.55	454.1	0.0011	Singlet-A	H-12->L+5 (12%), H-6->L+6 (13%), H-5->L+6 (21%), H-4->L+5 (12%)
**17**	22894.05	436.8	3E-4	Singlet-A	H-3->L+1 (34%), HOMO->L+4 (48%)
**18**	22944.05	435.8	0.0531	Singlet-A	H-2->L+2 (80%)
**19**	23541.71	424.8	0.1511	Singlet-A	H-3->L+2 (76%)
**20**	24199.86	413.2	0.0039	Singlet-A	H-5->L+1 (23%), H-4->LUMO (20%), H-3->L+1 (10%), HOMO->L+4 (18%)
**21**	24261.96	412.2	0.0421	Singlet-A	H-5->LUMO (35%), H-4->L+1 (13%), H-1->L+3 (32%)
**22**	24954.79	400.7	0.1542	Singlet-A	H-4->LUMO (18%), H-3->L+2 (10%), H-2->L+3 (15%), HOMO->L+4 (19%)
**23**	25162.89	397.4	0.0634	Singlet-A	H-5->LUMO (21%), H-1->L+3 (38%), H-1->L+4 (11%)
**24**	25479.05	392.5	0.6151	Singlet-A	H-6->LUMO (74%)
**25**	25762.96	388.2	0.0066	Singlet-A	HOMO->L+5 (48%)

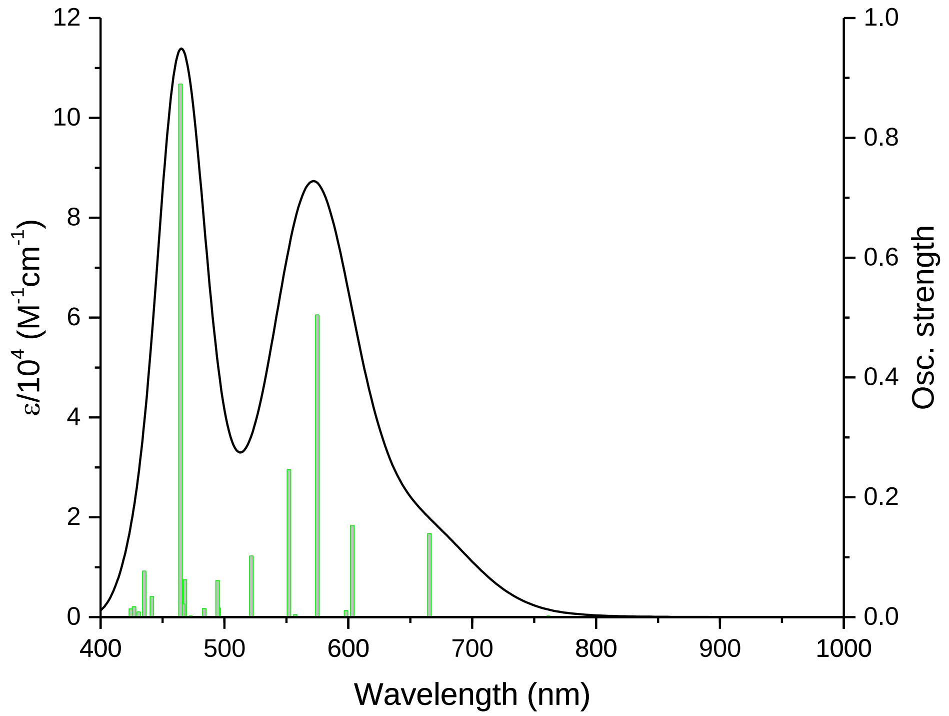

Figure S167. Calculated electronic transitions and UV-vis spectrum for **6b**.

Table S17. Electronic transitions calculated for **6b**

No.	Energy (cm^{-1})	Wavelength (nm)	Osc. Strength	Symmetry	Major contributions
**1**	13133.13	761.4	0.002	Singlet-A	HOMO->LUMO (97%)
**2**	15026.11	665.5	0.1398	Singlet-A	HOMO->L+1 (95%)
**3**	16577.11	603.2	0.1534	Singlet-A	HOMO->L+2 (86%)
**4**	16719.07	598.1	0.0109	Singlet-A	H-2->LUMO (23%), H-1->LUMO (74%)
**5**	17393.35	574.9	0.5044	Singlet-A	HOMO->L+3 (91%)
**6**	17947.45	557.2	0.0044	Singlet-A	H-2->LUMO (69%), H-1->LUMO (21%)
**7**	18113.60	552.1	0.2464	Singlet-A	H-1->L+1 (82%)
**8**	19170.19	521.6	0.1022	Singlet-A	H-2->L+1 (21%), H-1->L+2 (53%)
**9**	19729.13	506.9	7E-4	Singlet-A	H-5->L+8 (10%), HOMO->L+8 (15%)
**10**	20182.41	495.5	0.0152	Singlet-A	H-2->L+1 (14%)
**11**	20223.55	494.5	0.0612	Singlet-A	H-2->L+1 (38%), H-1->L+2 (26%)
**12**	20677.64	483.6	0.0145	Singlet-A	H-2->L+1 (14%), H-2->L+2 (29%), H-1->L+3 (18%), HOMO->L+4 (19%)
**13**	20946.22	477.4	0.0012	Singlet-A	H-16->L+6 (22%), H-16->L+7 (15%), H-16->L+8 (46%)
**14**	21151.89	472.8	0.002	Singlet-A	H-3->LUMO (97%)
**15**	21364.01	468.1	0.0626	Singlet-A	H-1->L+3 (41%), HOMO->L+4 (30%)
**16**	21424.51	466.8	0.0221	Singlet-A	H-17->L+6 (20%), H-17->L+7 (19%), H-17->L+8 (28%), HOMO->L+4 (11%)
**17**	21528.55	464.5	0.8898	Singlet-A	H-3->L+1 (39%), H-2->L+2 (29%)
**18**	21901.99	456.6	0.0014	Singlet-A	H-5->L+8 (16%)
**19**	22387.53	446.7	9E-4	Singlet-A	--
**20**	22653.69	441.4	0.0346	Singlet-A	H-3->L+1 (18%), H-2->L+2 (13%), HOMO->L+5 (11%)
**21**	22978.74	435.2	0.0771	Singlet-A	H-2->L+3 (51%)
**22**	23217.48	430.7	0.0089	Singlet-A	H-5->LUMO (47%), H-4->LUMO (35%)
**23**	23419.11	427.0	0.0175	Singlet-A	H-7->L+1 (18%), H-2->L+3 (19%), HOMO->L+5 (18%)
**24**	23559.45	424.5	0.0139	Singlet-A	H-3->L+2 (70%)
**25**	23937.73	417.8	9E-4	Singlet-A	H-5->LUMO (27%), H-4->LUMO (54%)

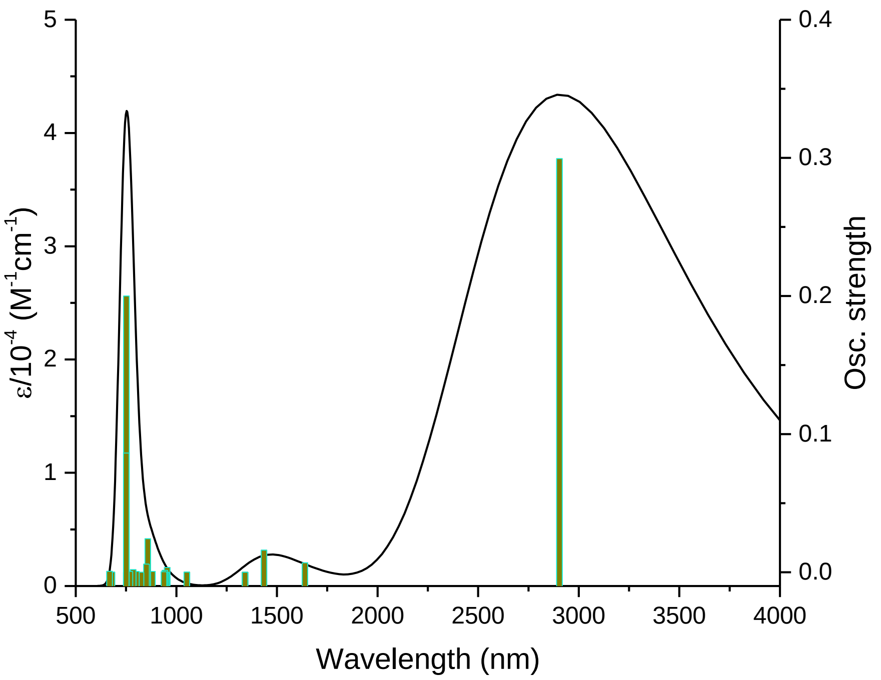

Figure S168. Calculated electronic transitions and vis-NIR spectrum for [**5aa**]$^{+\bullet}$.

Table S18. Electronic transitions calculated for [**5aa**]$^{+\bullet}$

No.	Energy (cm^{-1})	Wavelength (nm)	Osc. Strength	Symmetry	Major contributions
**1**	3443.18	2904.3	0.2994	2.006-A	HOMO(B)->LUMO(B) (99%)
**2**	6101.58	1638.9	0.0068	2.042-A	H-1(B)->LUMO(B) (99%)
**3**	6964.60	1435.8	0.016	2.053-A	H-2(B)->LUMO(B) (98%)
**4**	7455.79	1341.2	0	3.481-A	H-17(A)->L+8(A) (22%), H-16(A)->L+7(A) (23%), H-16(B)->L+9(B) (22%), H-15(B)->L+8(B) (23%)
**5**	7455.79	1341.2	0	3.481-A	H-17(A)->L+7(A) (23%), H-16(A)->L+8(A) (22%), H-16(B)->L+8(B) (23%), H-15(B)->L+9(B) (22%)
**6**	9497.18	1052.9	0	3.458-A	H-14(A)->L+7(A) (11%), H-13(A)->L+8(A) (11%), H-10(B)->L+9(B) (11%)
**7**	9510.08	1051.5	1E-4	3.457-A	H-14(A)->L+8(A) (10%), H-13(A)->L+7(A) (12%), H-10(B)->L+8(B) (11%)
**8**	10481.17	954.1	0.0034	2.058-A	H-4(B)->LUMO(B) (67%), H-3(B)->LUMO(B) (23%)
**9**	10535.21	949.2	0	3.371-A	H-15(A)->L+7(A) (12%), H-12(A)->L+8(A) (12%), H-14(B)->L+8(B) (13%)
**10**	10536.02	949.1	0	3.477-A	H-15(A)->L+8(A) (13%), H-12(A)->L+7(A) (14%), H-14(B)->L+9(B) (14%)
**11**	10598.93	943.5	0.0014	2.205-A	H-5(B)->LUMO(B) (89%)
**12**	10654.58	938.6	3E-4	2.042-A	H-4(B)->LUMO(B) (18%), H-3(B)->LUMO(B) (76%)
**13**	11350.64	881.0	5E-4	2.082-A	H-10(B)->LUMO(B) (77%), H-4(B)->LUMO(B) (14%)
**14**	11657.13	857.8	0.0242	2.074-A	H-11(B)->LUMO(B) (85%)
**15**	11743.43	851.5	0.0059	2.082-A	H-13(B)->LUMO(B) (83%), H-12(B)->LUMO(B) (11%)
**16**	12134.61	824.1	0	3.254-A	H-1(A)->LUMO(A) (16%), HOMO(B)->L+1(B) (64%)
**17**	12479.82	801.3	6E-4	2.087-A	H-14(B)->LUMO(B) (92%)
**18**	12736.30	785.2	2E-4	2.044-A	H-6(B)->LUMO(B) (98%)
**19**	12739.53	785.0	0.0018	2.044-A	H-7(B)->LUMO(B) (98%)
**20**	12991.98	769.7	5E-4	2.034-A	H-12(B)->LUMO(B) (83%)
**21**	13301.70	751.8	0.2	2.328-A	HOMO(A)->LUMO(A) (48%), H-8(B)->LUMO(B) (30%)
**22**	13309.76	751.3	0	2.039-A	H-9(B)->LUMO(B) (99%)
**23**	13312.18	751.2	0.0863	2.169-A	HOMO(A)->LUMO(A) (21%), H-8(B)->LUMO(B) (69%)
**24**	14714.78	679.6	1E-4	3.139-A	H-1(A)->L+3(A) (12%), HOMO(A)->L+5(A) (12%), HOMO(B)->L+2(B) (28%), HOMO(B)->L+4(B) (36%)
**25**	14974.49	667.8	7E-4	2.077-A	H-15(B)->LUMO(B) (96%)

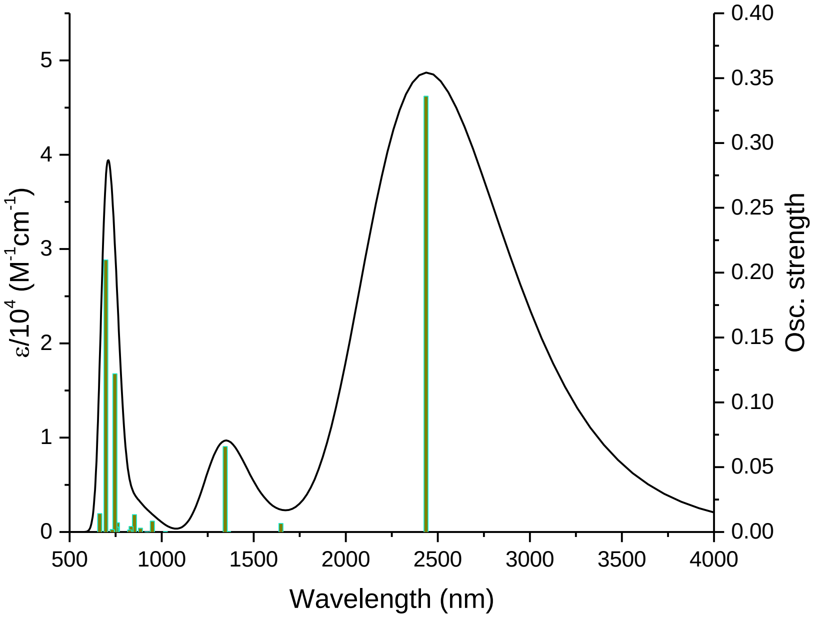

Figure S169. Calculated electronic transitions and vis-NIR spectrum for [**5ab**]$^{+•}$.

Table S19. Electronic transitions calculated for [**5ab**]$^{+•}$

No.	Energy (cm^{-1})	Wavelength (nm)	Osc. Strength	Symmetry	Major contributions
1	4105.36	2435.8	0.3361	2.010-A	HOMO(B)->LUMO(B) (98%)
2	6070.13	1647.4	0.0066	2.045-A	H-1(B)->LUMO(B) (97%)
3	7335.61	1363.2	4E-4	3.472-A	H-17(A)->L+7(A) (51%), H-16(B)->L+8(B) (51%)
4	7434.01	1345.2	0.0659	2.057-A	H-2(B)->LUMO(B) (94%)
5	7721.95	1295.0	0	3.474-A	H-16(A)->L+8(A) (51%), H-15(B)->L+9(B) (52%)
6	9468.14	1056.2	0	3.451-A	H-14(A)->L+7(A) (17%), H-13(B)->L+8(B) (11%), H-11(B)->L+8(B) (11%)
7	9802.06	1020.2	3E-4	3.423-A	H-11(A)->L+8(A) (16%), H-9(B)->L+9(B) (10%), H-8(B)->L+9(B) (10%)
8	10453.75	956.6	0	3.473-A	H-13(B)->L+8(B) (10%)
9	10537.63	949.0	0.0084	2.037-A	H-4(B)->LUMO(B) (35%), H-3(B)->LUMO(B) (62%)
10	10800.57	925.9	1E-4	3.365-A	H-14(B)->L+9(B) (12%)
11	10886.87	918.5	1E-4	2.110-A	H-4(B)->LUMO(B) (58%), H-3(B)->LUMO(B) (34%)
12	11304.67	884.6	0.0029	2.098-A	H-11(B)->LUMO(B) (20%), H-5(B)->LUMO(B) (68%)
13	11740.21	851.8	0.0134	2.088-A	H-11(B)->LUMO(B) (33%), H-9(B)->LUMO(B) (20%), H-8(B)->LUMO(B) (19%), H-5(B)->LUMO(B) (16%)
14	11990.24	834.0	0.0043	2.081-A	H-13(B)->LUMO(B) (59%), H-9(B)->LUMO(B) (11%), H-8(B)->LUMO(B) (12%)
15	12102.35	826.3	0.0016	2.084-A	H-13(B)->LUMO(B) (12%), H-11(B)->LUMO(B) (38%), H-9(B)->LUMO(B) (15%), H-8(B)->LUMO(B) (18%)
16	13163.77	759.7	0.0071	3.015-A	HOMO(A)->L+1(A) (29%), HOMO(B)->L+1(B) (11%), HOMO(B)->L+3(B) (17%)
17	13234.75	755.6	0.0038	2.058-A	H-7(B)->LUMO(B) (96%)
18	13342.02	749.5	0	2.038-A	H-6(B)->LUMO(B) (99%)
19	13404.93	746.0	0.122	2.928-A	H-1(A)->LUMO(A) (12%), HOMO(A)->LUMO(A) (21%), HOMO(B)->L+1(B) (25%), HOMO(B)->L+4(B) (10%)
20	13621.09	734.2	0.0014	2.128-A	H-14(B)->LUMO(B) (39%), H-13(B)->LUMO(B) (10%), H-12(B)->LUMO(B) (41%)
21	13669.48	731.6	0.0019	2.125-A	H-14(B)->LUMO(B) (43%), H-12(B)->LUMO(B) (44%)
22	13834.02	722.9	0	2.036-A	H-10(B)->LUMO(B) (97%)
23	13925.16	718.1	0	2.035-A	H-9(B)->LUMO(B) (51%), H-8(B)->LUMO(B) (48%)
24	14354.25	696.7	0.2099	2.680-A	HOMO(A)->LUMO(A) (51%), HOMO(B)->L+1(B) (20%)
25	15075.31	663.3	0.0142	2.927-A	H-2(A)->L+1(A) (14%), HOMO(A)->L+1(A) (12%)

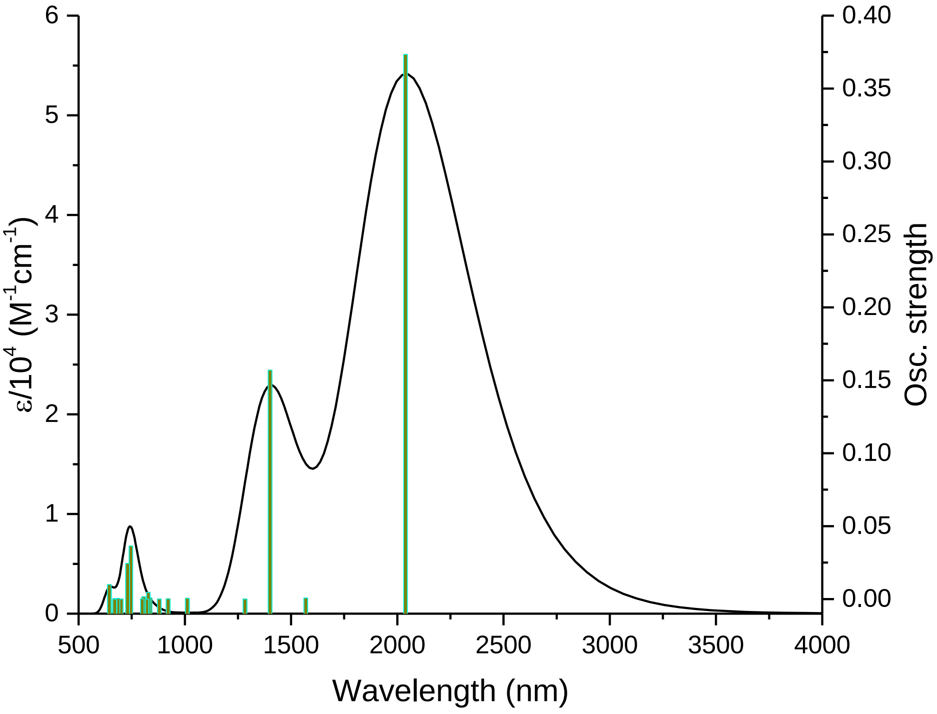

Figure S170. Calculated electronic transitions and vis-NIR spectrum for [**5bb-1**]$^{+•}$.

Table S20. Electronic transitions calculated for [**5bb-1**]$^{+•}$

No.	Energy (cm^{-1})	Wavelength (nm)	Osc. Strength	Symmetry	Major contributions
**1**	4905.46	2038.5	0.3734	2.016-A	HOMO(B)->LUMO(B) (96%)
**2**	6373.39	1569.0	8E-4	2.041-A	H-1(B)->LUMO(B) (99%)
**3**	7139.62	1400.6	0.157	2.041-A	H-2(B)->LUMO(B) (95%)
**4**	7793.74	1283.1	1E-4	3.470-A	H-17(A)->L+6(A) (27%), H-16(A)->L+5(A) (28%), H-16(B)->L+7(B) (27%), H-15(B)->L+6(B) (28%)
**5**	7793.74	1283.1	0	3.471-A	H-17(A)->L+5(A) (28%), H-16(A)->L+6(A) (27%), H-16(B)->L+6(B) (28%), H-15(B)->L+7(B) (27%)
**6**	9878.68	1012.3	0	3.423-A	H-9(B)->L+7(B) (10%), H-6(B)->L+6(B) (11%)
**7**	9887.55	1011.4	6E-4	3.420-A	H-9(B)->L+6(B) (10%), H-6(B)->L+7(B) (10%)
**8**	10849.77	921.7	0	3.455-A	H-12(B)->L+6(B) (15%)
**9**	10857.03	921.1	2E-4	3.442-A	H-12(B)->L+7(B) (15%)
**10**	11366.77	879.8	1E-4	2.039-A	H-3(B)->LUMO(B) (94%)
**11**	11965.23	835.8	9E-4	2.072-A	H-6(B)->LUMO(B) (36%), H-5(B)->LUMO(B) (60%)
**12**	12071.70	828.4	0.0046	2.062-A	H-12(B)->LUMO(B) (58%), H-6(B)->LUMO(B) (20%), H-5(B)->LUMO(B) (14%)
**13**	12183.00	820.8	0	2.068-A	H-9(B)->LUMO(B) (48%), H-4(B)->LUMO(B) (42%)
**14**	12401.58	806.3	0.0016	2.048-A	H-12(B)->LUMO(B) (35%), H-6(B)->LUMO(B) (38%), H-5(B)->LUMO(B) (23%)
**15**	12506.43	799.6	2E-4	2.032-A	H-9(B)->LUMO(B) (45%), H-4(B)->LUMO(B) (53%)
**16**	13400.90	746.2	0.0364	2.512-A	HOMO(A)->LUMO(A) (69%)
**17**	13680.78	731.0	0.0244	3.053-A	H-1(A)->LUMO(A) (26%), HOMO(A)->L+1(A) (31%), HOMO(B)->L+1(B) (28%)
**18**	14280.85	700.2	0	2.031-A	H-8(B)->LUMO(B) (97%)
**19**	14283.27	700.1	0	2.031-A	H-7(B)->LUMO(B) (99%)
**20**	14601.05	684.9	4E-4	2.022-A	H-13(B)->LUMO(B) (98%)
**21**	14891.41	671.5	4E-4	3.140-A	H-2(A)->LUMO(A) (29%), H-14(B)->LUMO(B) (14%), H-1(B)->L+1(B) (15%)
**22**	14926.10	670.0	0	2.029-A	H-11(B)->LUMO(B) (98%)
**23**	14926.10	670.0	0	2.027-A	H-10(B)->LUMO(B) (97%)
**24**	15489.07	645.6	0.0076	2.287-A	H-14(B)->LUMO(B) (77%)
**25**	15510.85	644.7	0.01	3.087-A	H-3(A)->LUMO(A) (17%), H-2(A)->L+1(A) (16%), H-2(B)->L+1(B) (15%)

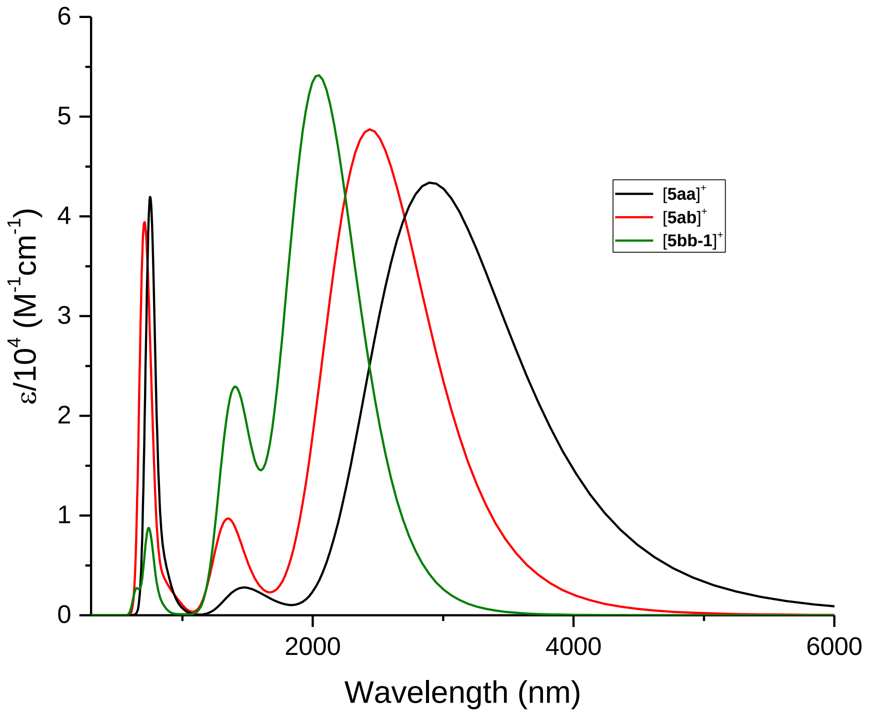

Figure S171. Comparison of calculated vis-NIR spectra of [**5aa**]$^{+\bullet}$ (black), of [**5ab**]$^{+\bullet}$ (red), and [**5bb-1**]$^{+\bullet}$ (green).

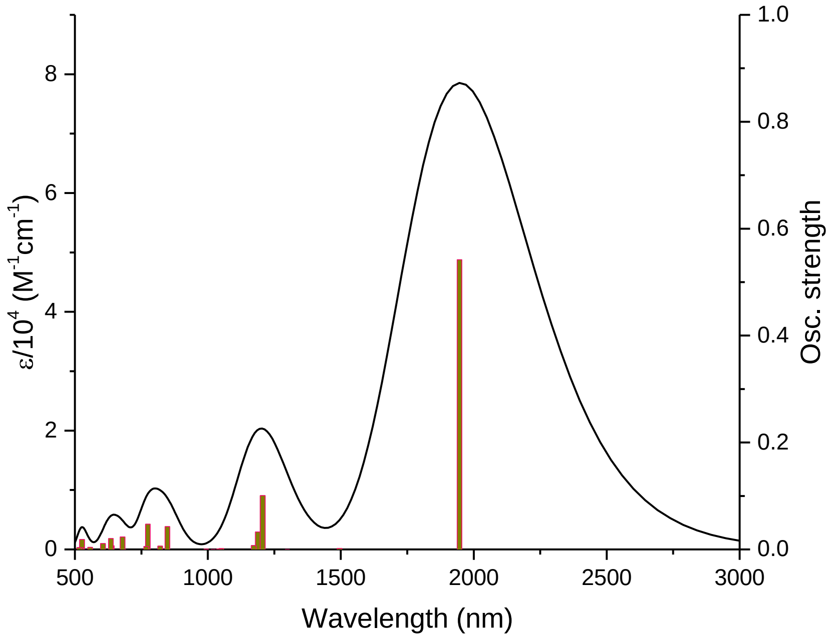

Figure S172. Calculated electronic transitions and vis-NIR spectrum for s-[**5aa**]$^{2+}$.

Table S21. Electronic transitions calculated for s-[**5aa**]$^{2+}$

No.	Energy (cm^{-1})	Wavelength (nm)	Osc. Strength	Symmetry	Major contributions
1	5137.75	1946.4	0.5418	Singlet-A	HOMO->LUMO (115%)
2	6687.95	1495.2	0.0019	Singlet-A	H-1->LUMO (100%)
3	7700.18	1298.7	1E-4	Singlet-A	H-2->LUMO (100%)
4	8289.77	1206.3	0.1006	Singlet-A	H-4->LUMO (48%), H-3->LUMO (47%)
5	8419.62	1187.7	0.0329	Singlet-A	H-4->LUMO (49%), H-3->LUMO (46%)
6	8528.51	1172.5	0.0073	Singlet-A	H-6->LUMO (10%), H-5->LUMO (88%)
7	9514.12	1051.1	0.0016	Singlet-A	H-6->LUMO (87%), H-5->LUMO (11%)
8	9598.00	1041.9	9E-4	Singlet-A	H-9->LUMO (33%), H-7->LUMO (63%)
9	9805.28	1019.9	0	Singlet-A	H-8->LUMO (97%)
10	9806.09	1019.8	1E-4	Singlet-A	H-9->LUMO (65%), H-7->LUMO (35%)
11	10060.96	993.9	7E-4	Singlet-A	H-10->LUMO (100%)
12	11791.02	848.1	0.0428	Singlet-A	H-12->LUMO (90%)
13	12186.23	820.6	0.0061	Singlet-A	H-14->LUMO (13%), H-11->LUMO (85%)
14	12911.32	774.5	0.0472	Singlet-A	H-13->LUMO (90%)
15	13016.18	768.3	0.0056	Singlet-A	H-14->LUMO (85%), H-11->LUMO (12%)
16	14620.41	684.0	1E-4	Singlet-A	H-16->LUMO (99%)
17	14643.80	682.9	1E-4	Singlet-A	H-17->LUMO (98%)
18	14725.26	679.1	0.0236	Singlet-A	HOMO->L+1 (95%)
19	15650.38	639.0	0.0069	Singlet-A	H-15->LUMO (97%)
20	15734.26	635.6	0.0205	Singlet-A	H-18->LUMO (96%)
21	16516.62	605.5	0.0111	Singlet-A	HOMO->L+2 (92%)
22	17943.42	557.3	0.004	Singlet-A	HOMO->L+3 (92%)
23	18981.45	526.8	0.0186	Singlet-A	H-20->LUMO (25%), HOMO->L+4 (66%)
24	19113.73	523.2	0.0034	Singlet-A	H-20->LUMO (69%), HOMO->L+4 (28%)
25	19367.79	516.3	0.0035	Singlet-A	H-19->LUMO (97%)

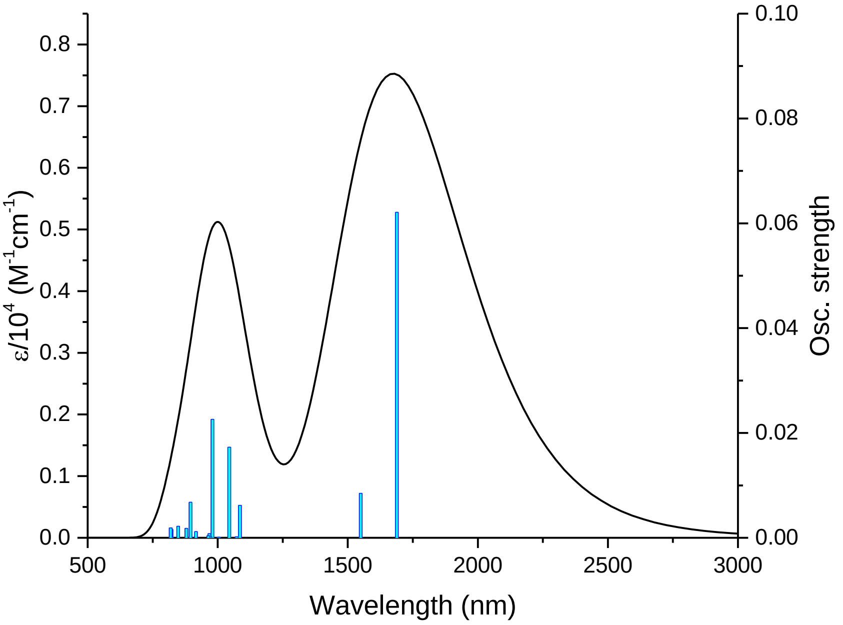

Figure S173. Calculated electronic transitions and vis-NIR spectrum for t-[**5aa**]$^{2+}$.

Table 22. Electronic transitions calculated for t-[**5aa**]$^{2+}$

No.	Energy (cm^{-1})	Wavelength (nm)	Osc. Strength	Symmetry	Major contributions
1	4887.72	2045.9	0.0417	3.025-A	HOMO(B)->LUMO(B) (78%)
2	6220.15	1607.7	0.0292	3.031-A	H-1(B)->LUMO(B) (42%), HOMO(B)->L+1(B) (24%)
3	7461.44	1340.2	0	4.130-A	H-17(A)->L+7(A) (41%), H-17(A)->L+8(A) (15%), H-15(B)->L+9(B) (41%), H-15(B)->L+10(B) (13%)
4	7809.06	1280.6	0	4.122-A	H-18(A)->L+7(A) (15%), H-18(A)->L+8(A) (40%), H-17(B)->L+9(B) (12%), H-17(B)->L+10(B) (36%)
5	8555.93	1168.8	0.0082	3.024-A	H-3(B)->LUMO(B) (10%), H-2(B)->LUMO(B) (54%), H-1(B)->LUMO(B) (31%)
6	8887.42	1125.2	0.0089	3.027-A	H-3(B)->LUMO(B) (64%), H-2(B)->LUMO(B) (27%)
7	9281.83	1077.4	0.0087	3.027-A	H-8(B)->LUMO(B) (38%), H-5(B)->LUMO(B) (17%), H-1(B)->LUMO(B) (10%), HOMO(B)->L+1(B) (10%)
8	9920.62	1008.0	0.0087	3.046-A	H-10(B)->LUMO(B) (14%), H-5(B)->LUMO(B) (15%), HOMO(B)->L+1(B) (29%)
9	9950.46	1005.0	4E-4	3.024-A	H-4(B)->LUMO(B) (81%)
10	10137.58	986.4	5E-4	3.400-A	H-11(B)->LUMO(B) (39%), H-11(B)->L+1(B) (13%)
11	10194.85	980.9	0	4.052-A	H-13(A)->L+7(A) (16%), H-10(B)->L+9(B) (13%)
12	10250.50	975.6	0.0043	3.023-A	H-8(B)->LUMO(B) (28%), H-5(B)->LUMO(B) (42%), H-5(B)->L+1(B) (14%)
13	10454.56	956.5	0	3.052-A	H-6(B)->LUMO(B) (86%)
14	10466.66	955.4	0.0166	3.075-A	H-10(B)->LUMO(B) (21%), H-1(B)->L+1(B) (12%), HOMO(B)->L+1(B) (10%)
15	10659.42	938.1	0.0016	3.666-A	H-11(B)->LUMO(B) (19%), H-11(B)->L+10(B) (10%)
16	10672.33	937.0	0	3.077-A	H-7(B)->LUMO(B) (76%), H-7(B)->L+1(B) (14%)
17	10694.11	935.1	0	4.097-A	H-14(A)->L+7(A) (15%), H-12(B)->L+9(B) (14%)
18	10815.89	924.6	0.0136	3.139-A	H-9(B)->LUMO(B) (10%), H-1(B)->L+1(B) (38%)
19	10958.66	912.5	4E-4	3.111-A	H-12(B)->LUMO(B) (11%), H-10(B)->LUMO(B) (10%), H-9(B)->LUMO(B) (29%), H-2(B)->L+1(B) (31%)
20	11117.55	899.5	0.0044	3.869-A	--
21	11169.17	895.3	0.0101	3.115-A	H-12(B)->LUMO(B) (42%), H-12(B)->L+1(B) (17%)
22	11698.27	854.8	0.0027	3.027-A	H-9(B)->LUMO(B) (10%), H-3(B)->L+1(B) (63%)
23	11981.37	834.6	5E-4	3.018-A	H-9(B)->LUMO(B) (39%), H-3(B)->L+1(B) (22%), H-2(B)->L+1(B) (34%)
24	12287.86	813.8	0.0022	3.087-A	H-14(B)->LUMO(B) (10%), H-13(B)->LUMO(B) (41%), H-12(B)->L+1(B) (15%)
25	12949.23	772.2	0.0045	3.032-A	H-8(B)->L+1(B) (54%), H-5(B)->L+1(B) (24%)

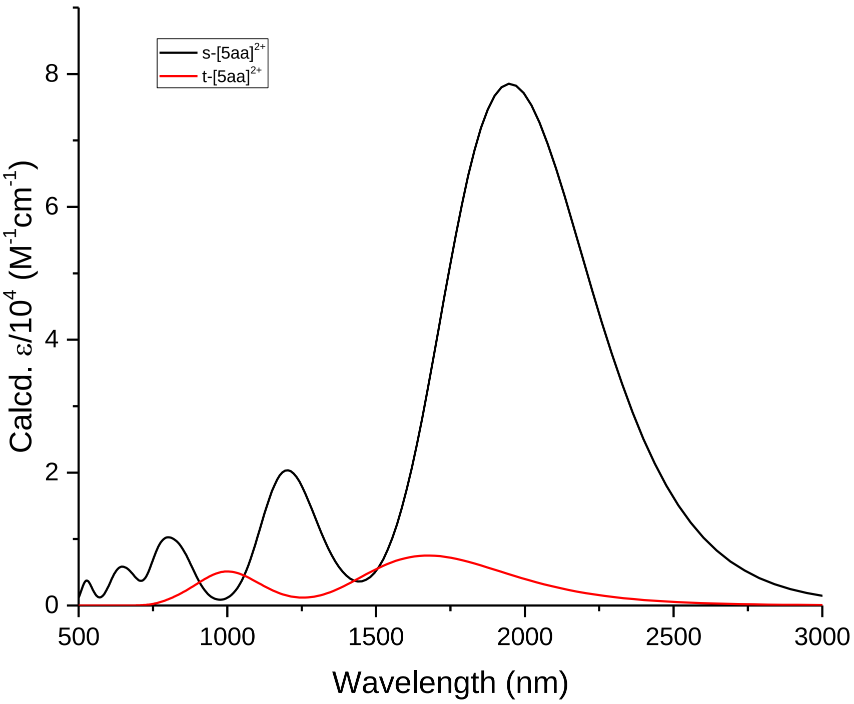

Figure S174. Comparison of the calculated vis-NIR spectra for s-[**5aa**]$^{2+}$ (black) and t-[**5aa**]$^{2+}$ (red).

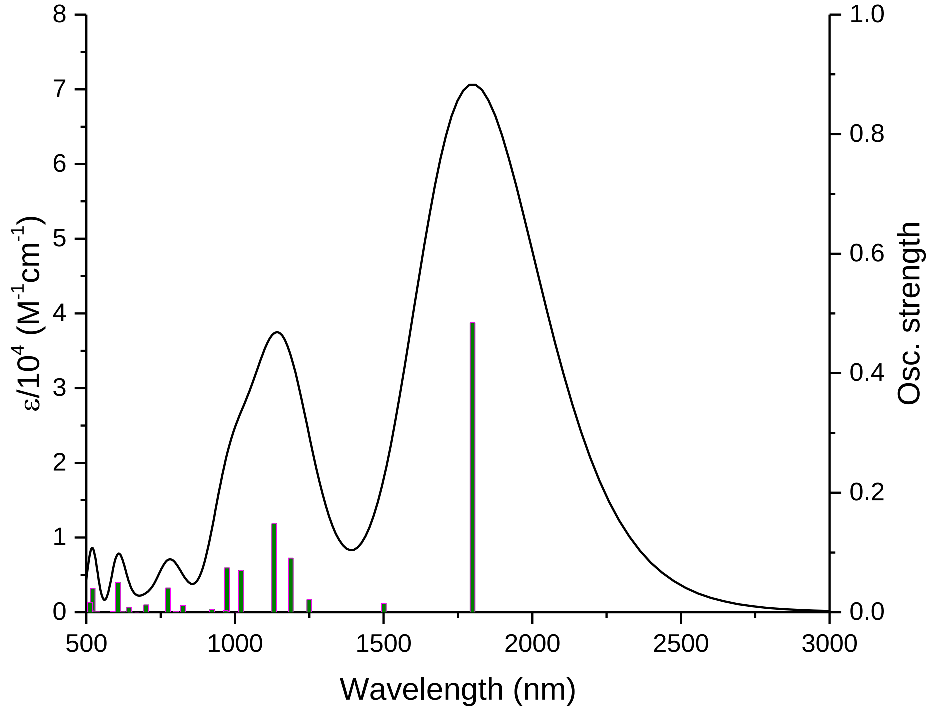

Figure S175. Calculated electronic transitions and vis-NIR spectrum for s-[**5ab**]$^{2+}$.

Table 23. Electronic transitions calculated for s-[**5ab**]$^{2+}$

No.	Energy (cm^{-1})	Wavelength (nm)	Osc. Strength	Symmetry	Major contributions
**1**	5558.77	1799.0	0.4845	Singlet-A	HOMO->LUMO (99%)
**2**	6665.37	1500.3	0.0146	Singlet-A	H-1->LUMO (99%)
**3**	7999.41	1250.1	0.0209	Singlet-A	H-3->LUMO (13%), H-2->LUMO (85%)
**4**	8420.43	1187.6	0.0906	Singlet-A	H-4->LUMO (44%), H-3->LUMO (47%)
**5**	8834.19	1132.0	0.1481	Singlet-A	H-4->LUMO (47%), H-3->LUMO (30%)
**6**	9806.90	1019.7	0.0697	Singlet-A	H-9->LUMO (27%), H-7->LUMO (48%), H-6->LUMO (12%)
**7**	9929.49	1007.1	1E-4	Singlet-A	H-5->LUMO (99%)
**8**	10277.12	973.0	0.0741	Singlet-A	H-9->LUMO (60%), H-7->LUMO (35%)
**9**	10314.22	969.5	0.0019	Singlet-A	H-7->LUMO (11%), H-6->LUMO (84%)
**10**	10323.90	968.6	0.0029	Singlet-A	H-8->LUMO (96%)
**11**	10836.06	922.8	0.0042	Singlet-A	H-10->LUMO (99%)
**12**	12111.22	825.7	0.0117	Singlet-A	H-13->LUMO (15%), H-12->LUMO (65%), H-11->LUMO (17%)
**13**	12470.94	801.9	0.0018	Singlet-A	H-12->LUMO (12%), H-11->LUMO (80%)
**14**	12911.32	774.5	0.0405	Singlet-A	H-13->LUMO (75%), H-12->LUMO (18%)
**15**	14260.69	701.2	0.0123	Singlet-A	H-14->LUMO (86%)
**16**	14916.42	670.4	1E-4	Singlet-A	H-16->LUMO (99%)
**17**	15426.97	648.2	2E-4	Singlet-A	H-17->LUMO (90%)
**18**	15521.33	644.3	0.0085	Singlet-A	H-15->LUMO (88%)
**19**	16032.69	623.7	6E-4	Singlet-A	HOMO->L+1 (83%)
**20**	16498.88	606.1	0.0498	Singlet-A	HOMO->L+1 (10%), HOMO->L+2 (85%)
**21**	16987.65	588.7	0.0015	Singlet-A	H-18->LUMO (88%)
**22**	18608.82	537.4	7E-4	Singlet-A	H-19->LUMO (93%)
**23**	19183.09	521.3	0.0402	Singlet-A	HOMO->L+3 (61%), HOMO->L+4 (15%)
**24**	19432.32	514.6	0.0038	Singlet-A	H-20->LUMO (64%), HOMO->L+3 (19%)
**25**	19497.65	512.9	0.0166	Singlet-A	H-20->LUMO (15%), HOMO->L+3 (10%), HOMO->L+4 (62%)

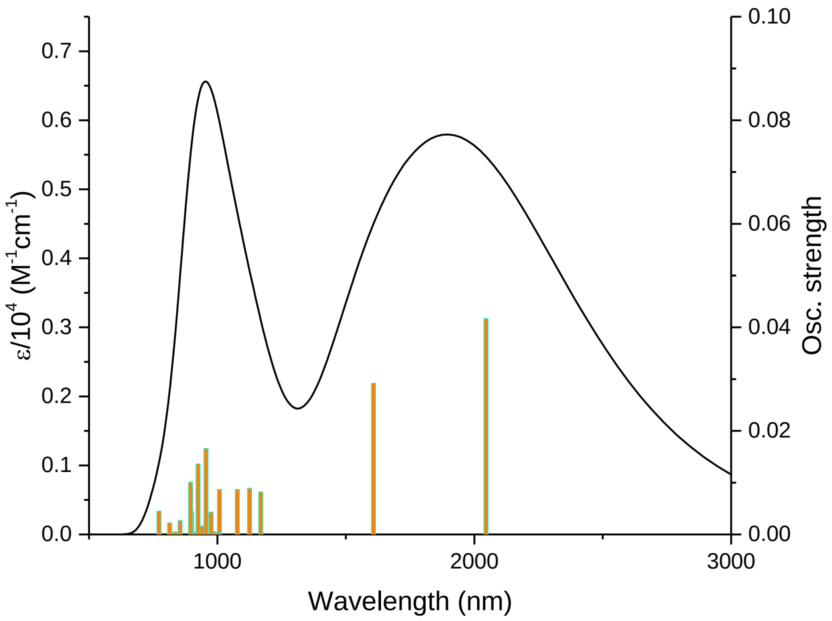

Figure S176. Calculated electronic transitions and vis-NIR spectrum for t-[**5ab**]$^{2+}$.

Table 24. Electronic transitions calculated for t-[**5ab**]$^{2+}$

No.	Energy (cm^{-1})	Wavelength (nm)	Osc. Strength	Symmetry	Major contributions
**1**	4887.72	2045.9	0.0417	3.025-A	HOMO(B)->LUMO(B) (78%)
**2**	6220.15	1607.7	0.0292	3.031-A	H-1(B)->LUMO(B) (42%), HOMO(B)->L+1(B) (24%)
**3**	7461.44	1340.2	0	4.130-A	H-17(A)->L+7(A) (41%), H-17(A)->L+8(A) (15%), H-15(B)->L+9(B) (41%), H-15(B)->L+10(B) (13%)
**4**	7809.06	1280.6	0	4.122-A	H-18(A)->L+7(A) (15%), H-18(A)->L+8(A) (40%), H-17(B)->L+9(B) (12%), H-17(B)->L+10(B) (36%)
**5**	8555.93	1168.8	0.0082	3.024-A	H-3(B)->LUMO(B) (10%), H-2(B)->LUMO(B) (54%), H-1(B)->LUMO(B) (31%)
**6**	8887.42	1125.2	0.0089	3.027-A	H-3(B)->LUMO(B) (64%), H-2(B)->LUMO(B) (27%)
**7**	9281.83	1077.4	0.0087	3.027-A	H-8(B)->LUMO(B) (38%), H-5(B)->LUMO(B) (17%), H-1(B)->LUMO(B) (10%), HOMO(B)->L+1(B) (10%)
**8**	9920.62	1008.0	0.0087	3.046-A	H-10(B)->LUMO(B) (14%), H-5(B)->LUMO(B) (15%), HOMO(B)->L+1(B) (29%)
**9**	9950.46	1005.0	4E-4	3.024-A	H-4(B)->LUMO(B) (81%)
**10**	10137.58	986.4	5E-4	3.400-A	H-11(B)->LUMO(B) (39%), H-11(B)->L+1(B) (13%)
**11**	10194.85	980.9	0	4.052-A	H-13(A)->L+7(A) (16%), H-10(B)->L+9(B) (13%)
**12**	10250.50	975.6	0.0043	3.023-A	H-8(B)->LUMO(B) (28%), H-5(B)->LUMO(B) (42%), H-5(B)->L+1(B) (14%)
**13**	10454.56	956.5	0	3.052-A	H-6(B)->LUMO(B) (86%)
**14**	10466.66	955.4	0.0166	3.075-A	H-10(B)->LUMO(B) (21%), H-1(B)->L+1(B) (12%), HOMO(B)->L+1(B) (10%)
**15**	10659.42	938.1	0.0016	3.666-A	H-11(B)->LUMO(B) (19%), H-11(B)->L+10(B) (10%)
**16**	10672.33	937.0	0	3.077-A	H-7(B)->LUMO(B) (76%), H-7(B)->L+1(B) (14%)
**17**	10694.11	935.1	0	4.097-A	H-14(A)->L+7(A) (15%), H-12(B)->L+9(B) (14%)
**18**	10815.89	924.6	0.0136	3.139-A	H-9(B)->LUMO(B) (10%), H-1(B)->L+1(B) (38%)
**19**	10958.66	912.5	4E-4	3.111-A	H-12(B)->LUMO(B) (11%), H-10(B)->LUMO(B) (10%), H-9(B)->LUMO(B) (29%), H-2(B)->L+1(B) (31%)
**20**	11117.55	899.5	0.0044	3.869-A	--
**21**	11169.17	895.3	0.0101	3.115-A	H-12(B)->LUMO(B) (42%), H-12(B)->L+1(B) (17%)
**22**	11698.27	854.8	0.0027	3.027-A	H-9(B)->LUMO(B) (10%), H-3(B)->L+1(B) (63%)
**23**	11981.37	834.6	5E-4	3.018-A	H-9(B)->LUMO(B) (39%), H-3(B)->L+1(B) (22%), H-2(B)->L+1(B) (34%)
**24**	12287.86	813.8	0.0022	3.087-A	H-14(B)->LUMO(B) (10%), H-13(B)->LUMO(B) (41%), H-12(B)->L+1(B) (15%)
**25**	12949.23	772.2	0.0045	3.032-A	H-8(B)->L+1(B) (54%), H-5(B)->L+1(B) (24%)

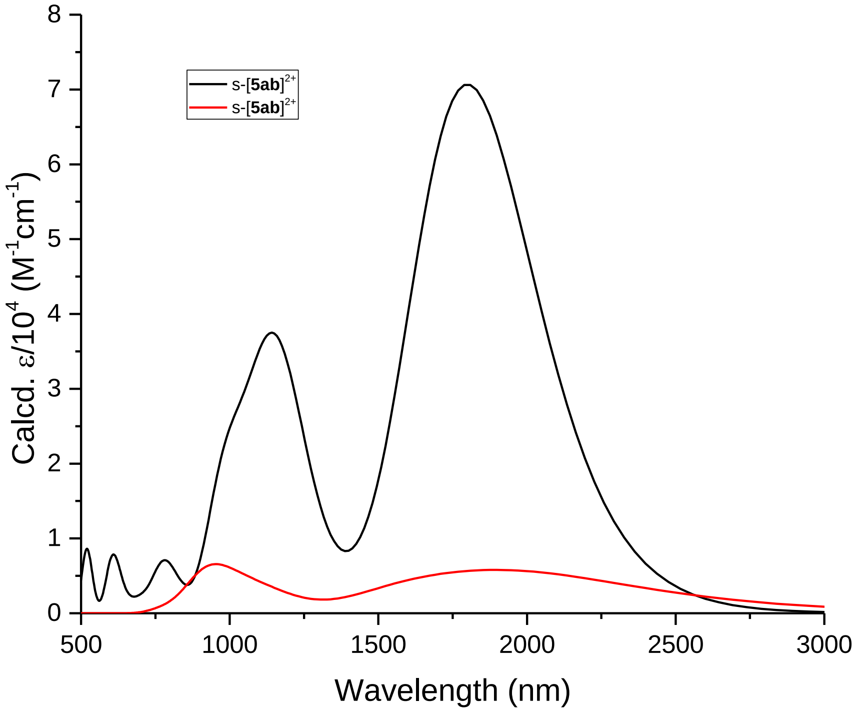

Figure S177. Comparison of the calculated vis-NIR spectra for s-[**5ab**]$^{2+}$(black) and t-[**5ab**]$^{2+}$ (red).

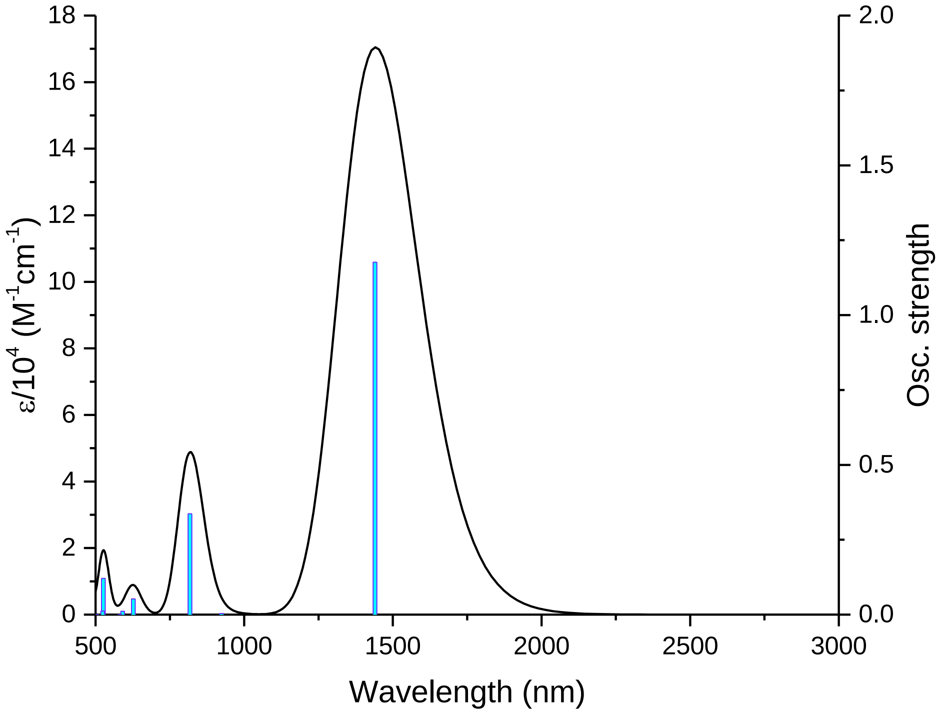

Figure S178. Calculated electronic transitions and vis-NIR spectrum for s-[**5bb-1**]$^{2+}$.

Table S25. Electronic transitions calculated for s-[**5bb-1**]$^{2+}$.

No	Energy (cm^{-1})	Wavelength (nm)	Osc. Strength	Symmetry	Major contributions
**1**	6944.43	1440.0	1.1763	Singlet-A	HOMO->LUMO (97%)
**2**	10825.57	923.7	0.0041	Singlet-A	H-1->LUMO (96%)
**3**	12230.59	817.6	0.3369	Singlet-A	H-2->LUMO (90%)
**4**	15943.97	627.2	0.0052	Singlet-A	H-3->LUMO (84%)
**5**	15944.77	627.2	0.0525	Singlet-A	H-13->LUMO (67%)
**6**	16913.45	591.2	0.0114	Singlet-A	H-9->LUMO (86%)
**7**	17176.38	582.2	3E-4	Singlet-A	H-6->LUMO (86%)
**8**	19006.46	526.1	0.1212	Singlet-A	H-11->LUMO (11%), HOMO->L+1 (72%)
**9**	19090.34	523.8	0.012	Singlet-A	H-11->LUMO (61%), HOMO->L+1 (13%)
**10**	19244.39	519.6	0	Singlet-A	H-12->LUMO (84%)
**11**	19851.72	503.7	4E-4	Singlet-A	H-10->LUMO (83%)
**12**	20431.64	489.4	0	Singlet-A	H-4->LUMO (92%)
**13**	20434.86	489.4	0	Singlet-A	H-5->LUMO (92%)
**14**	20738.13	482.2	7E-4	Singlet-A	H-24->L+6 (42%), H-23->L+7 (41%)
**15**	20805.88	480.6	0.0052	Singlet-A	H-24->L+7 (44%), H-23->L+6 (43%)
**16**	21493.06	465.3	2E-4	Singlet-A	H-8->LUMO (91%)
**17**	21493.06	465.3	0	Singlet-A	H-7->LUMO (94%)
**18**	21510.00	464.9	0.1147	Singlet-A	HOMO->L+2 (74%)
**19**	21748.74	459.8	1E-4	Singlet-A	H-34->L+6 (14%), H-12->L+6 (19%), H-11->L+7 (19%)
**20**	21763.26	459.5	0.0144	Singlet-A	H-34->L+7 (12%), H-12->L+7 (17%), H-11->L+6 (17%)
**21**	22318.17	448.1	0.0079	Singlet-A	H-33->L+6 (10%), H-31->L+7 (14%), H-17->L+7 (13%), H-13->L+6 (21%)
**22**	22389.95	446.6	0	Singlet-A	H-33->L+7 (11%), H-31->L+6 (16%), H-17->L+6 (14%), H-13->L+7 (23%)
**23**	22569.81	443.1	0.0153	Singlet-A	H-16->LUMO (78%)
**24**	23167.47	431.6	0.0207	Singlet-A	HOMO->L+3 (63%)
**25**	24151.47	414.1	0.0138	Singlet-A	H-23->LUMO (56%), H-17->LUMO (23%)

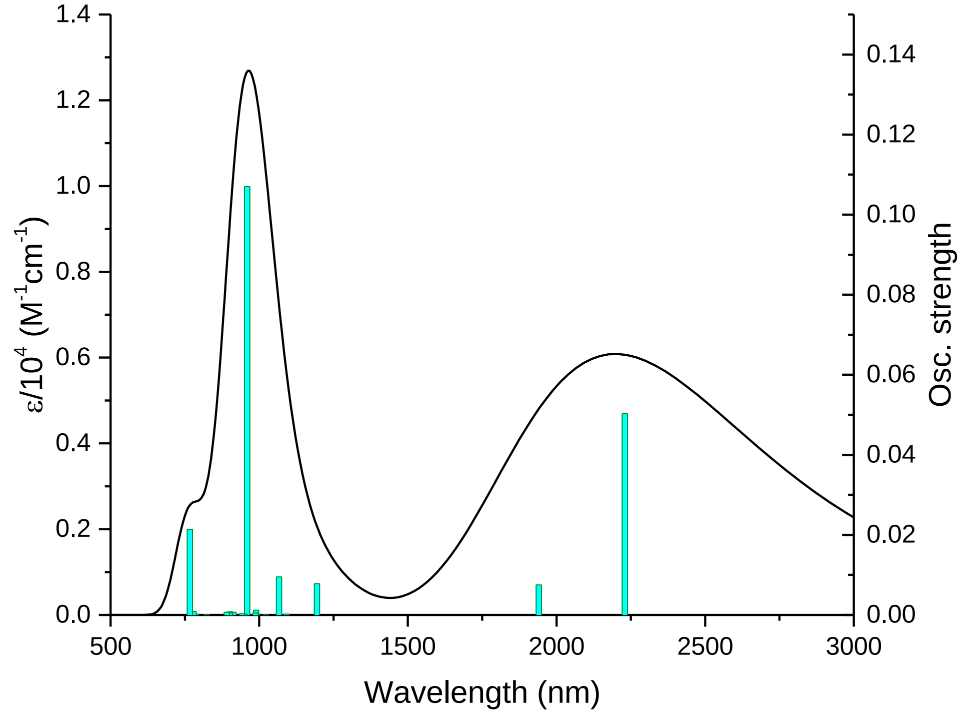

Figure S179. Calculated electronic transitions and vis-NIR spectrum for t-[**5bb-1**]$^{2+}$.

Table 26. Electronic transitions calculated for t-[**5bb-1**]$^{2+}$.

No.	Energy (cm^{-1})	Wavelength (nm)	Osc. Strength	Symmetry	Major contributions
**1**	4484.40	2229.9	0.0503	3.022-A	H-1(B)->L+1(B) (15%), HOMO(B)->LUMO(B) (82%)
**2**	5153.10	1940.6	0.0075	3.025-A	H-1(B)->LUMO(B) (70%), HOMO(B)->L+1(B) (25%)
**3**	7809.06	1280.6	0	4.127-A	H-22(A)->L+5(A) (27%), H-21(A)->L+6(A) (17%), H-19(B)->L+7(B) (26%), H-18(B)->L+8(B) (26%)
**4**	7809.06	1280.6	0	4.127-A	H-22(A)->L+6(A) (26%), H-21(A)->L+5(A) (18%), H-19(B)->L+8(B) (26%), H-18(B)->L+7(B) (27%)
**5**	8374.45	1194.1	0.0078	3.018-A	H-2(B)->LUMO(B) (93%)
**6**	9160.85	1091.6	2E-4	3.024-A	H-6(B)->LUMO(B) (34%), H-4(B)->LUMO(B) (12%), H-1(B)->LUMO(B) (21%), HOMO(B)->L+1(B) (24%)
**7**	9377.81	1066.3	0.0095	3.022-A	H-3(B)->LUMO(B) (89%)
**8**	9781.89	1022.3	1E-4	3.064-A	H-10(B)->LUMO(B) (26%), H-6(B)->LUMO(B) (14%), HOMO(B)->L+1(B) (33%)
**9**	10077.09	992.3	1E-4	3.027-A	H-6(B)->LUMO(B) (11%), H-4(B)->LUMO(B) (74%)
**10**	10082.74	991.8	1E-4	3.024-A	H-5(B)->LUMO(B) (91%)
**11**	10102.90	989.8	0.0012	3.232-A	H-11(B)->LUMO(B) (60%), H-10(B)->L+1(B) (14%)
**12**	10127.90	987.4	5E-4	3.141-A	H-10(B)->LUMO(B) (30%), H-6(B)->LUMO(B) (28%), HOMO(B)->L+1(B) (11%)
**13**	10425.52	959.2	0.107	3.040-A	H-1(B)->L+1(B) (81%), HOMO(B)->LUMO(B) (15%)
**14**	10501.34	952.3	1E-4	3.943-A	H-11(B)->L+8(B) (11%), H-10(B)->L+7(B) (12%)
**15**	10510.21	951.5	2E-4	3.890-A	H-11(B)->LUMO(B) (12%), H-11(B)->L+7(B) (11%), H-10(B)->L+8(B) (11%)
**16**	10607.80	942.7	1E-4	3.033-A	H-8(B)->LUMO(B) (90%)
**17**	10611.03	942.4	3E-4	3.042-A	H-7(B)->LUMO(B) (89%)
**18**	10950.59	913.2	7E-4	3.515-A	H-12(B)->LUMO(B) (37%)
**19**	11090.12	901.7	8E-4	3.183-A	H-9(B)->LUMO(B) (43%), H-2(B)->L+1(B) (34%)
**20**	11092.54	901.5	5E-4	4.092-A	H-12(B)->L+8(B) (16%)
**21**	11225.62	890.8	7E-4	3.568-A	H-12(B)->LUMO(B) (39%)
**22**	12158.81	822.4	1E-4	3.021-A	H-9(B)->LUMO(B) (39%), H-2(B)->L+1(B) (55%)
**23**	12703.23	787.2	2E-4	3.077-A	H-16(B)->LUMO(B) (33%), H-15(B)->LUMO(B) (19%), H-12(B)->L+1(B) (30%)
**24**	12843.57	778.6	9E-4	3.025-A	H-3(B)->L+1(B) (91%)
**25**	13041.99	766.8	0.0214	3.027-A	H-6(B)->L+1(B) (67%), H-4(B)->L+1(B) (22%)

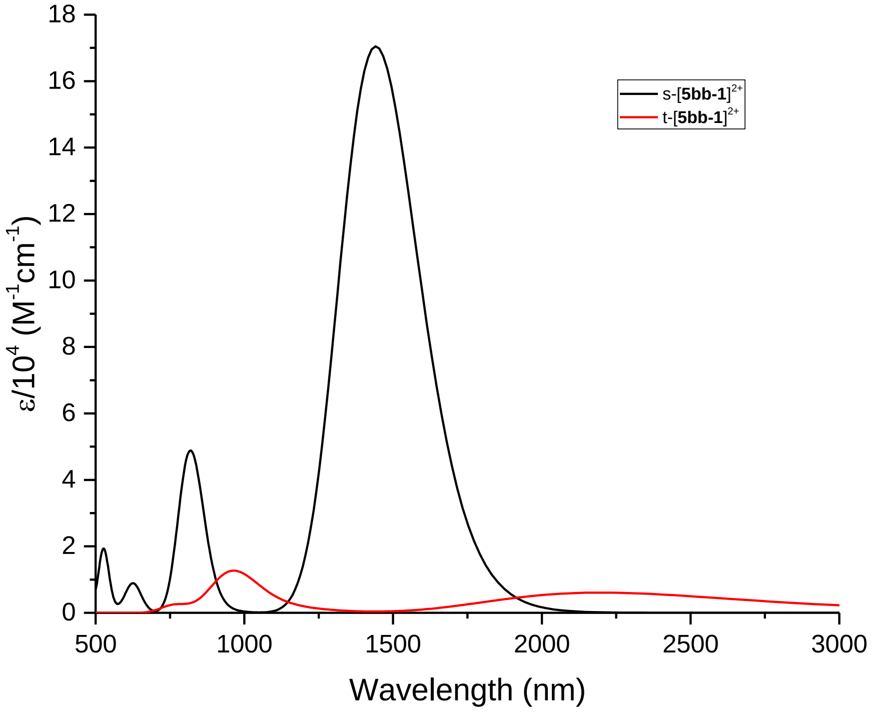

Figure S180. Comparison of the calculated vis-NIR spectra for s-[**5bb-1**]$^{2+}$(black) and t-[**5bb-1**]$^{2+}$ (red).

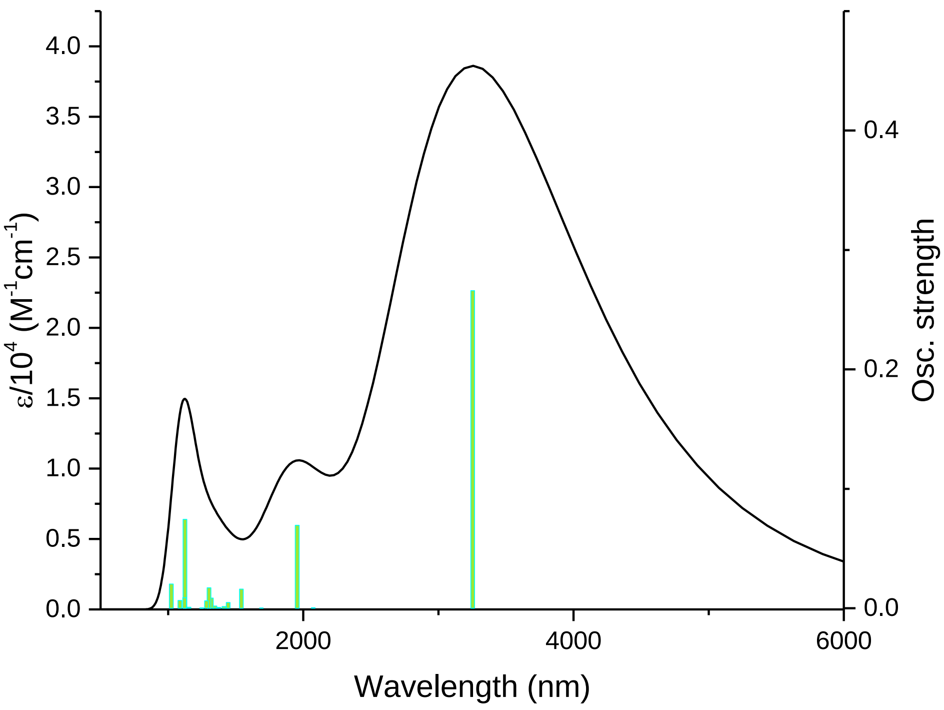

Figure S181. Calculated electronic transitions and vis-NIR spectrum for d-[**5aa**]$^{3+}$.

Table 27. Electronic transitions calculated for d-[**5aa**]$^{3+}$

No	Energy (cm^{-1})	Wavelength (nm)	Osc. Strength	Symmetry	Major contributions
**1**	3073.78	3253.3	0.266	2.003-A	HOMO(A)->LUMO(A) (100%)
**2**	4822.39	2073.7	4E-4	3.085-A	H-9(A)->LUMO(A) (18%), H-2(A)->LUMO(A) (20%), H-1(A)->LUMO(A) (24%), H-9(B)->LUMO(B) (14%), H-8(B)->L+1(B) (10%)
**3**	5114.36	1955.3	0.0694	2.861-A	H-10(A)->LUMO(A) (21%), H-9(B)->L+1(B) (16%), H-8(B)->LUMO(B) (20%), HOMO(B)->LUMO(B) (22%)
**4**	5918.50	1689.6	1E-4	2.833-A	H-2(A)->LUMO(A) (38%), H-1(A)->LUMO(A) (60%)
**5**	6197.56	1613.5	0	2.701-A	H-4(A)->LUMO(A) (13%), H-2(A)->LUMO(A) (34%), H-1(A)->LUMO(A) (13%)
**6**	6483.89	1542.3	0.016	2.790-A	H-7(A)->LUMO(A) (62%), H-3(A)->LUMO(A) (31%)
**7**	6925.88	1443.9	0.0048	2.702-A	H-7(A)->LUMO(A) (28%), H-3(A)->LUMO(A) (32%), HOMO(B)->LUMO(B) (26%)
**8**	7074.29	1413.6	0.0014	2.798-A	H-4(A)->LUMO(A) (78%)
**9**	7268.67	1375.8	7E-4	2.178-A	H-1(B)->LUMO(B) (69%), HOMO(B)->LUMO(B) (17%)
**10**	7292.87	1371.2	0	2.814-A	H-5(A)->LUMO(A) (90%)
**11**	7295.28	1370.7	0	2.853-A	H-6(A)->LUMO(A) (92%)
**12**	7333.19	1363.7	0	3.494-A	H-18(A)->L+9(A) (21%), H-17(A)->L+8(A) (24%), H-17(B)->L+10(B) (20%), H-16(B)->L+9(B) (24%)
**13**	7334.00	1363.5	0	3.498-A	H-18(A)->L+8(A) (24%), H-17(A)->L+9(A) (22%), H-17(B)->L+9(B) (23%), H-16(B)->L+10(B) (21%)
**14**	7426.75	1346.5	0.0019	2.463-A	H-3(A)->LUMO(A) (20%), H-3(B)->LUMO(B) (14%), H-1(B)->LUMO(B) (12%), HOMO(B)->LUMO(B) (15%)
**15**	7573.55	1320.4	0.0085	2.510-A	H-9(A)->LUMO(A) (15%), H-6(B)->LUMO(B) (16%), H-2(B)->LUMO(B) (47%)
**16**	7679.20	1302.2	0.0171	2.453-A	H-3(B)->LUMO(B) (53%), H-2(B)->L+1(B) (20%)
**17**	7788.09	1284.0	0.0063	2.182-A	H-6(B)->LUMO(B) (64%), H-3(B)->L+1(B) (18%), H-2(B)->LUMO(B) (10%)
**18**	7999.41	1250.1	1E-4	2.827-A	H-8(A)->LUMO(A) (98%)
**19**	8017.96	1247.2	0	2.275-A	H-5(B)->LUMO(B) (15%), H-5(B)->L+1(B) (23%), H-4(B)->LUMO(B) (59%)
**20**	8018.76	1247.1	1E-4	2.288-A	H-5(B)->LUMO(B) (57%), H-4(B)->LUMO(B) (15%), H-4(B)->L+1(B) (23%)
**21**	8661.59	1154.5	9E-4	2.599-A	H-7(B)->LUMO(B) (28%), H-1(B)->L+1(B) (30%), HOMO(B)->L+1(B) (31%)
**22**	8889.84	1124.9	0.0744	2.329-A	H-10(A)->LUMO(A) (41%), H-8(B)->LUMO(B) (39%)
**23**	8909.20	1122.4	0.0088	2.438-A	H-9(A)->LUMO(A) (33%), H-1(B)->L+1(B) (21%)
**24**	9199.56	1087.0	0.0065	2.540-A	H-9(A)->LUMO(A) (14%), H-9(B)->LUMO(B) (27%), HOMO(B)->L+1(B) (24%)
**25**	9768.99	1023.6	0.0203	2.685-A	H-6(B)->L+1(B) (66%), H-2(B)->L+1(B) (15%)

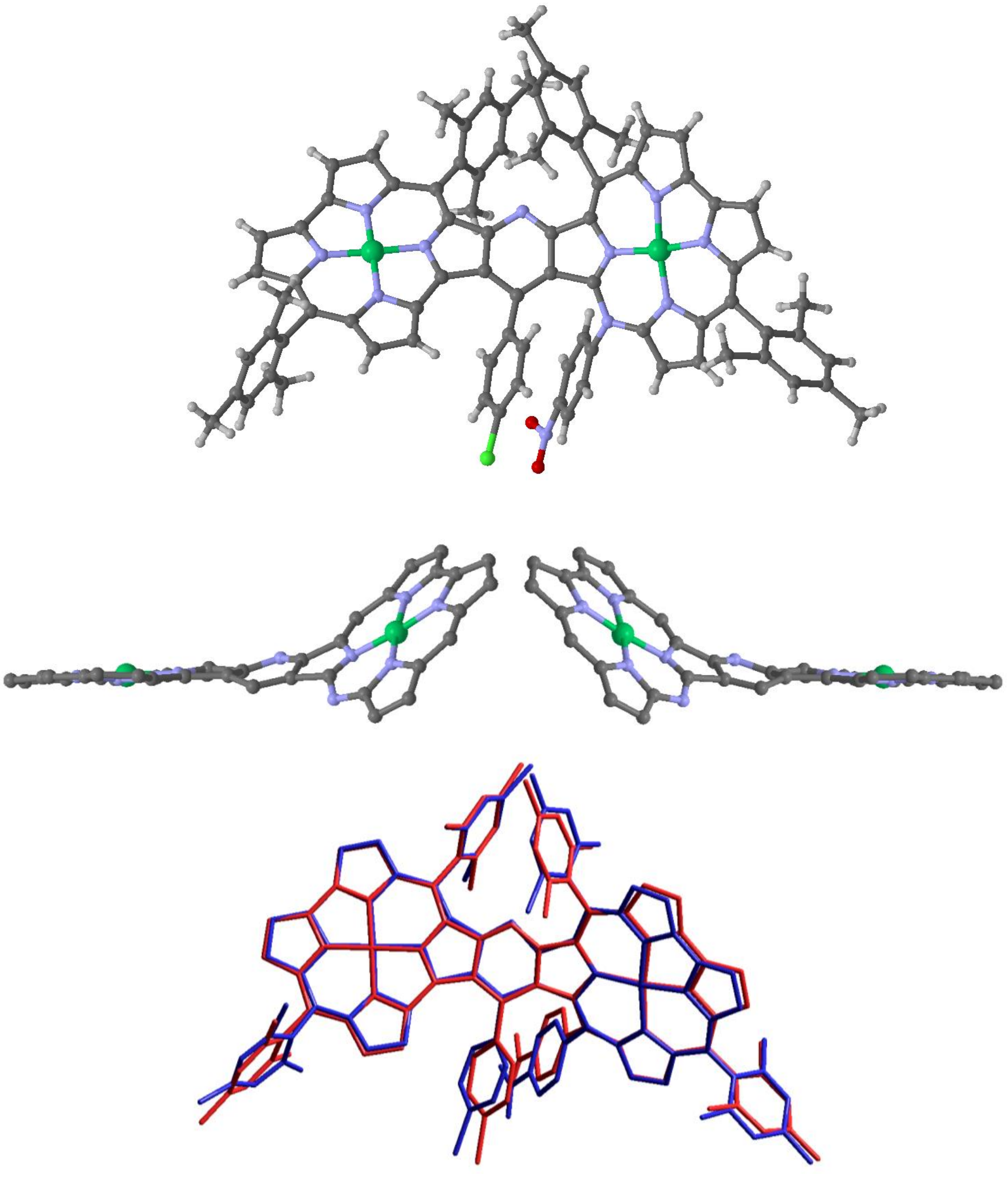

Figure S182. Molecular structure of **4a** (balls-and-sticks representation) based on the preliminary single crystal X-ray diffraction analysis (top; only one out of two symmetry-independent molecules is shown), side views of the enantiomers present in the unit cells with all hydrogens and all aryl substituents omitted (mid), and superimposed stick representations of the X-ray molecular structure (blue) and DFT-calculated model of **4a** (red) with all hydrogens omitted.

19. Frontier Orbitals of Cation Radicals [**5aa**], [**5ab**], and [**5bb-1**]

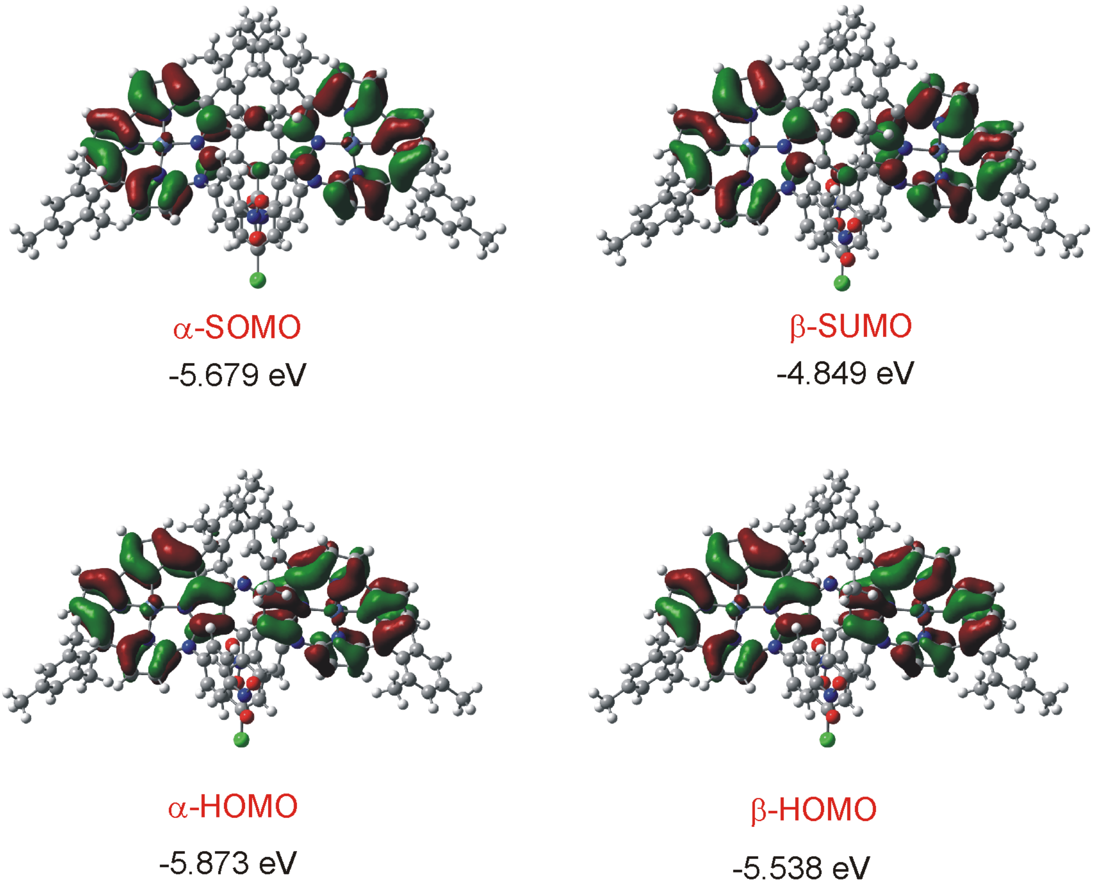

Figure S183. Frontier spinorbitals (isovalue 0.02) for [**5aa**]$^{+•}$.

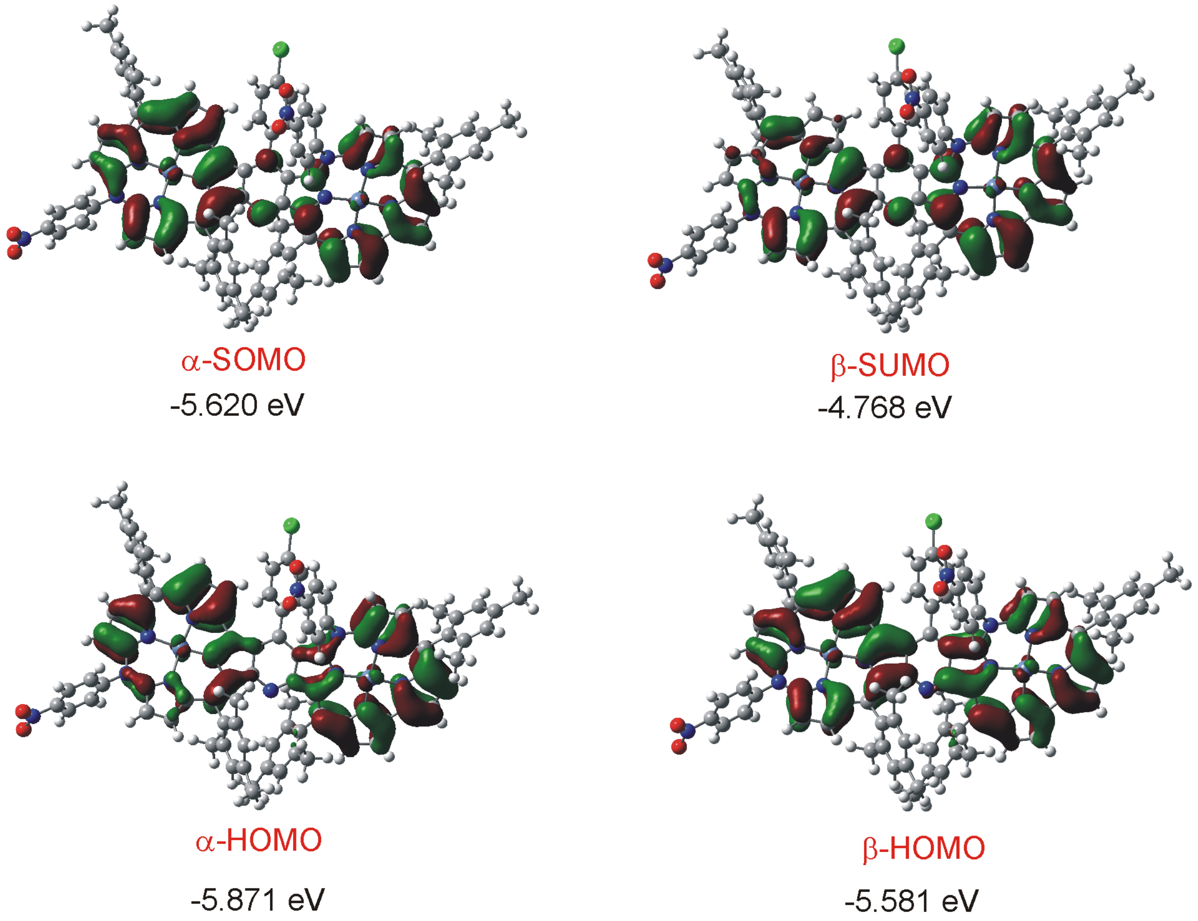

Figure S184. Frontier spinorbitals (isovalue 0.02) for [**5ab**]$^{+•}$.

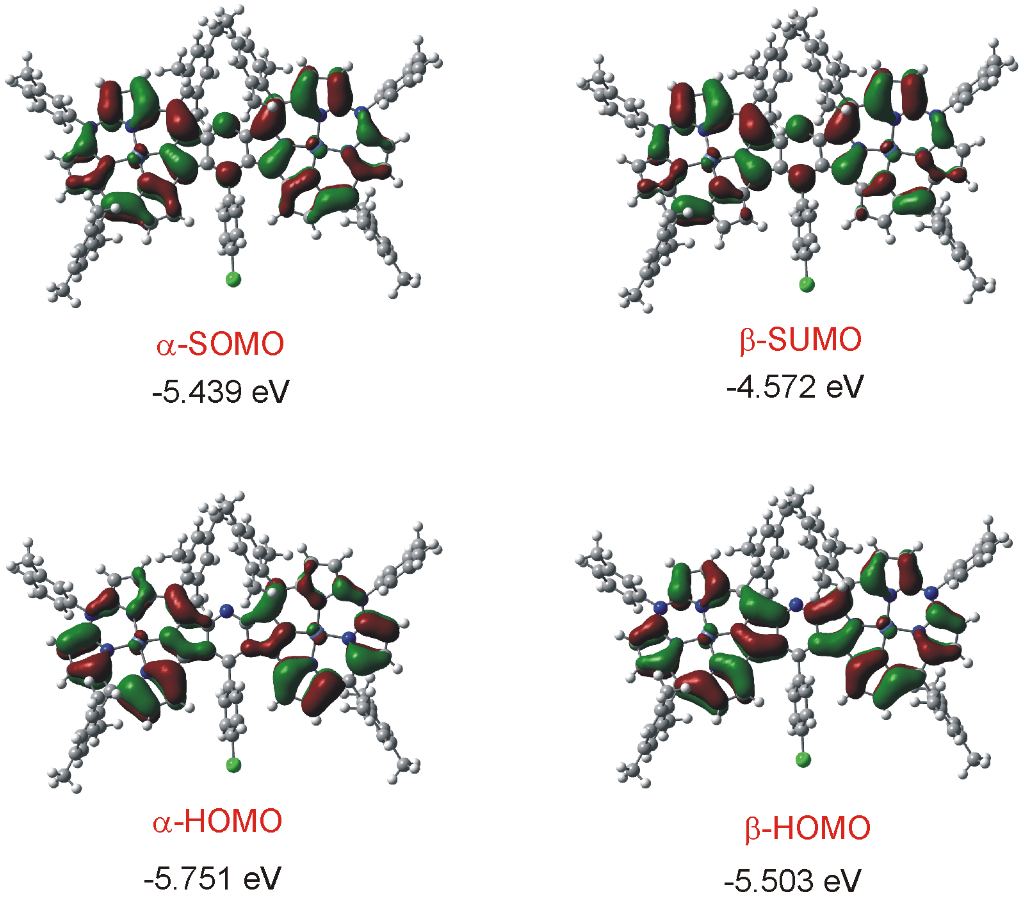

Figure S185. Frontier spinorbitals (isovalue 0.02) for [**5bb-1**]$^{+\bullet}$.

20. Crystallographic Data

Table S28 Crystal data and structure refinement for **5aa**.

**Identification code**	tx5543a_sq
**Empirical formula**	$C_{97}H_{74}ClN_{13}Ni_2O_4$
**Formula weight**	1638.56
**Temperature/K**	170.15
**Crystal system**	triclinic
**Space group**	P-1
**a/Å**	9.7866(2)
**b/Å**	20.9926(3)
**c/Å**	24.6984(4)
**α/°**	69.250(2)
**β/°**	79.525(2)
**γ/°**	79.622(2)
**Volume/Å³**	4628.88(15)
**Z**	2
**ρ_{calc}g/cm³**	1.176
**μ/mm⁻¹**	1.210
**F(000)**	1704.0
**Crystal size/mm³**	0.21 × 0.15 × 0.01
**Radiation**	CuKα (λ = 1.54184)
**2Θ range for data collection/°**	4.538 to 151.198
**Index ranges**	-12 ≤ h ≤ 12, -26 ≤ k ≤ 26, -30 ≤ l ≤ 30
**Reflections collected**	68088
**Independent reflections**	18338 [R_{int} = 0.0417, R_{sigma} = 0.0357]
**Data/restraints/parameters**	18338/6/1066
**Goodness-of-fit on F²**	1.061
**Final R indexes [I>=2σ (I)]**	R_1 = 0.0575, wR_2 = 0.1436
**Final R indexes [all data]**	R_1 = 0.0693, wR_2 = 0.1502
**Largest diff. peak/hole / e Å⁻³**	0.42/-0.52

Table S29 Crystal data and structure refinement for **5ab**.

**Identification code**	tx3924_sq
**Empirical formula**	$C_{91}H_{68}ClN_{13}Ni_2O_4$
**Formula weight**	1560.45
**Temperature/K**	110.15
**Crystal system**	monoclinic
**Space group**	$P2_1/n$
**a/Å**	22.8347(4)
**b/Å**	31.7760(3)
**c/Å**	27.9926(4)
**α/°**	90
**β/°**	105.2578(16)
**γ/°**	90
**Volume/Å³**	19595.3(5)
**Z**	8
**ρ_{calc}g/cm³**	1.058
**μ/mm^{-1}**	1.121
**F(000)**	6480.0
**Crystal size/mm³**	0.18 × 0.07 × 0.01
**Radiation**	CuKα (λ = 1.54184)
**2Θ range for data collection/°**	4.294 to 130
**Index ranges**	-26 ≤ h ≤ 26, -37 ≤ k ≤ 37, -32 ≤ l ≤ 30
**Reflections collected**	163902
**Independent reflections**	33293 [R_{int} = 0.0976, R_{sigma} = 0.0700]
**Data/restraints/parameters**	33293/0/2023
**Goodness-of-fit on F²**	1.037
**Final R indexes [I>=2σ (I)]**	R_1 = 0.0740, wR_2 = 0.2000
**Final R indexes [all data]**	R_1 = 0.1144, wR_2 = 0.2284
**Largest diff. peak/hole / e Å^{-3}**	1.79/-0.59

Table S30. Crystal data and structure refinement for **5bb_1**.

**Identification code**	LS_3
**Empirical formula**	$C_{93}H_{74}ClN_{11}Ni_2$
**Formula weight**	1498.50
**Temperature/K**	170.15
**Crystal system**	monoclinic
**Space group**	I2/a
**a/Å**	15.0235(2)
**b/Å**	17.1243(2)
**c/Å**	36.2360(4)
**α/°**	90
**β/°**	95.1330(10)
**γ/°**	90
**Volume/Å³**	9284.94(19)
**Z**	4
**ρ_{calc}g/cm³**	1.072
**μ/mm⁻¹**	1.123
**F(000)**	3128.0
**Crystal size/mm³**	0.48 × 0.31 × 0.02
**Radiation**	CuKα (λ = 1.54184)
**2Θ range for data collection/°**	4.896 to 151.078
**Index ranges**	-18 ≤ h ≤ 14, -21 ≤ k ≤ 20, -45 ≤ l ≤ 45
**Reflections collected**	36454
**Independent reflections**	9316 [R_{int} = 0.0520, R_{sigma} = 0.0342]
**Data/restraints/parameters**	9316/1/493
**Goodness-of-fit on F²**	1.053
**Final R indexes [I>=2σ (I)]**	R_1 = 0.0701, wR_2 = 0.2229
**Final R indexes [all data]**	R_1 = 0.0783, wR_2 = 0.2309
**Largest diff. peak/hole / e Å⁻³**	0.82/-0.49

Table S31. Crystal data and structure refinement for **5bb_1_I2**.

**Identification code**	LS_3_I2
**Empirical formula**	$C_{94}H_{75}Cl_3I_{1.95}N_{11}Ni_2$
**Formula weight**	1829.87
**Temperature/K**	100.15
**Crystal system**	monoclinic
**Space group**	I2/a
**a/Å**	14.94269(16)
**b/Å**	16.94000(17)
**c/Å**	36.5712(4)
**α/°**	90
**β/°**	95.3560(11)
**γ/°**	90
**Volume/Å^3**	9216.81(18)
**Z**	4
**ρ_{calc}g/cm^3**	1.319
**μ/mm^{-1}**	6.814
**F(000)**	3705.0
**Crystal size/mm^3**	0.191 × 0.122 × 0.010
**Radiation**	CuKα (λ = 1.54184)
**2Θ range for data collection/°**	4.854 to 146.366
**Index ranges**	-17 ≤ h ≤ 18, -11 ≤ k ≤ 20, -43 ≤ l ≤ 45
**Reflections collected**	35115
**Independent reflections**	8848 [R_{int} = 0.0244, R_{sigma} = 0.0191]
**Data/restraints/parameters**	8848/0/595
**Goodness-of-fit on F^2**	1.090
**Final R indexes [I>=2σ (I)]**	R_1 = 0.0976, wR_2 = 0.2688
**Final R indexes [all data]**	R_1 = 0.1035, wR_2 = 0.2723
**Largest diff. peak/hole / e Å^{-3}**	1.13/-0.73

21. References

[1] G. M. Sheldrick, Acta Cryst. **2015**, *C71*, 3-8.
[2] CrysAlis PRO 1.171.40.67a (Rigaku OD, 2019).
[3] G. M. Sheldrick, Acta Cryst. **2015**, *A71*, 3.
[4] G. M. Sheldrick, Acta Cryst. **2015**, *C71*, 3.
[5] Gaussian 16, Revision C.01, M. J. Frisch, G. W. Trucks, H. B. Schlegel, G. E. Scuseria, M. A. Robb, J. R. Cheeseman, G. Scalmani, V. Barone, G. A. Petersson, H. Nakatsuji, X. Li, M. Caricato, A. V. Marenich, J. Bloino, B. G. Janesko, R. Gomperts, B. Mennucci, H. P. Hratchian, J. V. Ortiz, A. F. Izmaylov, J. L. Sonnenberg, D. Williams-Young, F. Ding, F. Lipparini, F. Egidi, J. Goings, B. Peng, A. Petrone, T. Henderson, D. Ranasinghe, V. G. Zakrzewski, J. Gao, N. Rega, G. Zheng, W. Liang, M. Hada, M. Ehara, K. Toyota, R. Fukuda, J. Hasegawa, M. Ishida, T. Nakajima, Y. Honda, O. Kitao, H. Nakai, T. Vreven, K. Throssell, J. A. Montgomery, Jr., J. E. Peralta, F. Ogliaro, M. J. Bearpark, J. J. Heyd, E. N. Brothers, K. N. Kudin, V. N. Staroverov, T. A. Keith, R. Kobayashi, J. Normand, K. Raghavachari, A. P. Rendell, J. C. Burant, S. S. Iyengar, J. Tomasi, M. Cossi, J. M. Millam, M. Klene, C. Adamo, R. Cammi, J. W. Ochterski, R. L. Martin, K. Morokuma, O. Farkas, J. B. Foresman, and D. J. Fox, Gaussian, Inc., Wallingford CT, 2016.
[6] A. D. Becke, *Phys.Rev.A* **1988,** *38* 3098-3100.
[7] A. D. Becke, *J.Chem.Phys.* **1993,** *98* 5648.
[8] C. Lee, W. Yang, R. G. Parr, *Phys.Rev.B* **1988,** *37* 785-789.
[9] N. M. O'Boyle, A. L. Tenderholt, K. M. Langner, *J.Comp.Chem.* **2008,** *29* 839-845.
[10] a) D. Geuenich, R. Herges, *J. Phys. Chem. A* **2001**, *105*, 3214-3220; b) D. Geuenich, K. Hess, F. Koehler, R. Herges, *Chem. Rev.* **2005**, *105*, 3758-3772.
[11] T. Lu, F. Chen, Multiwfn: A Multifunctional Wavefunction Analyzer. *J. Comp. Chem.* **2012**, *33*, 580–592. https://doi.org/10.1002/jcc.22885.
[12] T. Lu, F. Chen, *J. Mol Graph. Modelling* **2012**, *38*, 314–323. https://doi.org/10.1016/j.jmgm.2012.07.004.
[13] J. Zhang, T. Lu, *Phys. Chem. Chem. Phys.* **2021**, *23*, 20323–20328. https://doi.org/10.1039/D1CP02805G.
[14] T. Ito, Y. Hayashi, S. Shimizu, J.-Y. Shin, N. Kobayashi, H. Shinokubo, *Angew.Chem.Int.Ed.* **2012,** *51* 8542-8545.
[15] Z. Deng, X. Li, M. Stępień, P. J. Chmielewski, *Chem.Eur.J.* **2016,** *22* 4231-4246.
[16] X. Li, Y. Meng, P. Yi, M. Stępień, P. J. Chmielewski, *Angew.Chem.Int.Ed.* **2017,** *56* 10810-10814.
[17] F. Mandoj, S. Nardis, R. Pudi, L. Lvova, F. R. Fronczek, K. M. Smith, L. Prodi, D. Genovese, R. Paolesse, *Dyes and Pigments* **2013,** *99* 136-143.
[18] X. Li, Y. Meng, P. Yi, M. Stępień, P. J. Chmielewski, *Angew.Chem.Int.Ed.* **2017,** *56* 10810-10814.
[19] S. Li, Y. Zhu, X. Li, S. Zhang, M. J. Białek, P. J. Chmielewski, *Org.Chem.Front.* **2023,** *10* 4342-4.
[20] B. Bleaney, K. D. Bowers, *Proc.Roy.Soc.A* **1952,** *A214* 451-465.
[21] S. K. Kaczmarek, H. Fuks, G. Leniec, T. Skibiński, A. Jasik, E. Tomaszewicz, T. Groń, *Current Topics in Biophysics* **2010,** *33* 103-108.
[22] J. Guo, Y. Yang, C. Dou, Y. Wang, *J.Am.Chem.Soc.* **2021,** *143* 18272-18279.
